# How to select predictive models for decision-making or causal inference

**DOI:** 10.1093/gigascience/giaf016

**Published:** 2025-03-21

**Authors:** Matthieu Doutreligne, Gaël Varoquaux

**Affiliations:** Soda, Inria Saclay, 91120, Palaiseau, France; Mission Data, Haute Autorité de Santé, 93210 Saint-Denis, France; Soda, Inria Saclay, 91120, Palaiseau, France

**Keywords:** Model Selection, Predictive model, Treatment Effect, G-computation, Machine Learning

## Abstract

**Background:**

We investigate which procedure selects the most trustworthy predictive model to explain the effect of an intervention and support decision-making.

**Methods:**

We study a large variety of model selection procedures in practical settings: finite samples settings and without a theoretical assumption of *well-specified* models. Beyond standard cross-validation or internal validation procedures, we also study elaborate causal risks. These build proxies of the causal error using “nuisance” reweighting to compute it on the observed data. We evaluate whether empirically estimated nuisances, which are necessarily noisy, add noise to model selection and compare different metrics for causal model selection in an extensive empirical study based on a simulation and 3 health care datasets based on real covariates.

**Results:**

Among all metrics, the mean squared error, classically used to evaluate predictive modes, is worse. Reweighting it with a propensity score does not bring much improvement in most cases. On average, the $R\text{-risk}$, which uses as nuisances a model of mean outcome and propensity scores, leads to the best performances. Nuisance corrections are best estimated with flexible estimators such as a super learner.

**Conclusions:**

When predictive models are used to explain the effect of an intervention, they must be evaluated with different procedures than standard predictive settings, using the $R\text{-risk}$ from causal inference.

## Introduction

### Extending prediction to prescription needs causality

Prediction models have long been used in biomedical settings, as with risk score or prognostic models [1, 2]. While these have historically been simple models on simple data, this is changing with progress in machine learning and richer medical data [3, 4]. Health predictions can now integrate medical images [5–9], patient records [10–12], or clinical notes [13–15]. Complex data are difficult to control and model, but these models are validated by verifying the accuracy of the prediction on left-out data [16–18]. Crucial to the clinical adoption of a model predicting a health outcome is that it “can support decisions about patient care” [19]. Precision medicine is about guiding decisions: for example, will an individual benefit from an intervention such as surgery [20]? An estimate of the effect of the treatment can be obtained by contrasting model predictions with and without the treatment, but statistical validity requires causal inference [21–23].

Indeed, concluding on the effect of a treatment is a difficult causal inference task, as it can be easily compromised by confounding: spurious associations between treatment allocation and baseline health (e.g., only prescribing a drug for mild cases) [24, 25]. Predictive modeling is linked to causal inference theory by the concept of *outcome models* (or g-computation, g-estimation, g-formula [26], Q-model [21], or conditional mean regression [27]). Medical statistics and epidemiology have mostly used other causal inference methods, modeling treatment assignment with propensity scores [28–31]. Outcome modeling brings the benefit of going beyond average effects, estimating individualized or conditional average treatment effects (CATEs), central to precision medicine. For this purpose, such methods are also invaluable for randomized trials [32–34].

Outcome modeling methods, even when specifically designed for causal inference, are numerous: Bayesian additive regression trees [35], targeted maximum likelihood estimation [36, 37], causal boosting [38], causal multivariate adaptive regression splines [38], random forests [39, 40], meta-learners [41], R-learners [42], and doubly robust estimation [43]. The wide variety of methods raises the problem of selecting between different estimators based on the data at hand. Indeed, estimates of treatment effects can vary markedly across different predictive models [44–47] (illustration in Appendix A.1).

Given complex health data, which predictive model is to be most trusted to yield valid causal estimates needed to motivate individual treatment decisions? As no single machine learning method performs best across all datasets, there is a pressing need for clear guidelines to select outcome models for causal inference.

#### Objectives and structure of the study

The intersection between machine learning and causal inference is growing rapidly [48, 49]. We focus on *model selection procedures* in practical settings, without theoretical assumptions often made in statistical literature such as *infinite* data or *well-specified* models (Appendix A.2). Asymptotic causal inference theory recommends complex risks, but a practical question is whether model selection procedures, which rely on data split, can estimate these risks reliably enough. Indeed, these risks come with more quantities to estimate, which may bring additional variance, leading to worse model selection.

We first illustrate the problem of causal model selection. Then we anchor causal model selection in the *potential outcome* framework and detail the causal risks and model selection procedure. We then rewrite the so-called $R\text{-risk}$ as a reweighted version of mean squared difference between the true and estimated individualized treatment effect. Finally, we conduct a thorough empirical study comparing the different metrics on diverse datasets, using a family of simulations and real health data, going beyond prior work limited to specific simulation settings [50, 51] (Appendix A.2).

### Illustration: the best predictor may not estimate best causal effects

**Figure 1:**
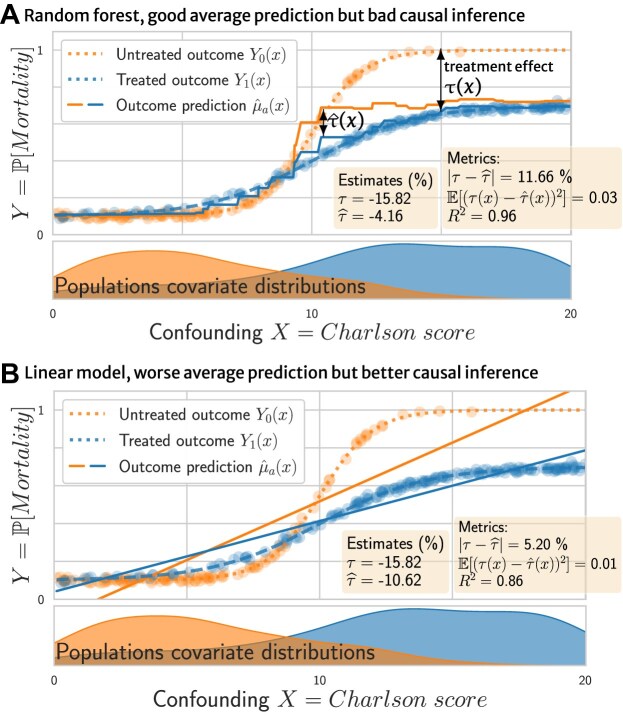
Illustration: (A) a random forest predictor with high performance for standard prediction (high $R^2$) but that yields poor causal estimates (large error between true effect $\tau$ and estimated $\hat{\tau }$) and (B) a linear predictor with smaller prediction performance leading to better causal estimation. Selecting the predictor with the smallest error for the individual treatment effect $\mathbb {E}[(\tau (x) - \hat{\tau }(x))^2]$—the $\tau \text{-risk}$, Eq. (10)—would lead to the best causal estimates; however, computing this error is not feasible: it requires access to unknown quantities: $\tau (x)$. While the random forest fits the data better than the linear model, it gives worse causal inference because its error is inhomogeneous between treated and untreated. The $R^2$ score does not capture this inhomogeneity.

Using a predictor to reason on causal effects relies on contrasting the prediction of the outcome for a given individual with and without the treatment. Given various predictors of the outcome, which one should we use? Standard predictive modeling or machine learning practice selects the predictor that minimizes the expected error on the outcome [17, 18]. However, this predictor may not be the best model to reason about causal effects of an intervention, as Fig. 1 illustrates. Consider the probability *Y* of an undesirable outcome (e.g., death), a binary treatment $A \in \lbrace 0, 1\rbrace$, and a covariate $X \in \mathbb {R}$ summarizing the patient’s health status (e.g., the Charlson index [52]). We simulate a treatment beneficial (decreases mortality) for patients with high Charlson scores (bad health status) but with little effect for patients in good condition (low Charlson scores).

Figure 1A shows a random forest predictor with a counterintuitive behavior: it predicts well on average the outcome (as measured by a regression $R^2$ score) but performs poorly to estimate causal quantities: the average treatment effect $\tau$ (as visible via the error $|\tau - \hat{\tau }|$) or the conditional average treatment effect (the error $\mathbb {E}[(\tau (x) - \hat{\tau }(x))^2]$, called CATE). On the contrary, Fig. 1B shows a linear model with a smaller $R^2$ score but better causal inference.

The problem is that causal estimation requires controlling an error on both treated and nontreated outcome for the same individual: the observed outcome and the nonobserved *counterfactual* one. The linear model is misspecified—the outcome functions are not linear—leading to poor $R^2$, but it interpolates better to regions where there are few untreated individuals—high Charlson score—and thus gives better causal estimates. Conversely, the random forest puts weaker assumptions on the data and thus has a higher $R^2$ score but is biased by the treated population in the poor-overlap region, leading to bad causal estimates.

This toy example illustrates that the classic minimum mean squared error (MSE) criterion is not suited to choosing a model among candidate estimators for causal inference.

## Methods

### Neyman–Rubin potential outcomes framework

We first expose the classic construction of the outcome modeling (or g-computation) estimators of causal effect [21, 24, 53].

#### Settings

The Neyman–Rubin potential outcomes framework [54, 55] enables statistical reasoning on causal treatment effects: given an outcome $Y \in \mathbb {R}$ (e.g., mortality risk or hospitalization length), function of a binary treatment $A \in \mathcal {A} = \lbrace 0, 1\rbrace$ (e.g., a medical procedure), and baseline covariates $X \in \mathcal {X} \subset \mathbb {R}^d$, we observe the factual distribution, $O = (Y(A), X, A) \sim \mathcal {D} = \mathbb {P}(y, x, a)$. However, we want to model the existence of potential observations (unobserved, i.e., counterfactual) that correspond to a different treatment. Thus, we want quantities on the counterfactual distribution $O^{*} = (Y(1), Y(0), X, A) \sim \mathcal {D}^{*} = \mathbb {P}(y(1), y(0), x, a)$.

Popular quantities of interest (estimands) are, at the population level, the average treatment effect


\begin{eqnarray*}
{\rm ATE} \qquad \tau \overset{\rm def}{=} {\mathbb {E}_{Y(1),Y(0) \sim \mathcal {D}^{*}}[Y(1) - Y(0)]};
\end{eqnarray*}

at the individual level, to model heterogeneity, the conditional average treatment effect


\begin{eqnarray*}
\text{CATE} \qquad \tau (x) \overset{\rm def}{=} \mathbb {E}_{Y(1),Y(0) \sim \mathcal {D}^\star }[Y(1) - Y(0) | X=x].
\end{eqnarray*}

#### Causal assumptions

A given data need to meet a few assumptions to enable identifying causal estimands [56]. (i) an individual’s outcome *Y* is solely governed by the corresponding potential outcome:


(1) \begin{eqnarray*}
\mathrm{{\it Consistency}\,\,\,assumption,} \qquad Y = A\, Y(1) + (1 - A)\, Y(0)
\end{eqnarray*}

(ii) unconfoundedness $\lbrace Y(0), Y(1) \rbrace \perp \!\!\! \perp A | X$, (iii) strong overlap (i.e., every patient has a strictly positive probability to receive each treatment), and (iv) generalization—no covariate shift. These classic assumptions, called *strong ignorability*, are formally detailed in Appendix A.3.

#### Identifying treatment effects with outcome models—G-computation [53]

Should we know the 2 potential outcomes for a given *X*, we could compute the difference between them, which gives the causal effect of the treatment. These 2 potential outcomes can be estimated from observed data: the consistency and unconfoundedness A.3 assumptions imply the following equality, linking the target quantity to the observed data:


(2) \begin{eqnarray*}
\mathbb {E}_{Y(a) \sim \mathcal {D^{\star }}} [Y(a)|X=x] = \mathbb {E}_{Y \sim \mathcal {D}} [Y|X=x, A=a]
\end{eqnarray*}

On the left, the expectation is taken on the counterfactual unobserved distribution. On the right, the expectation is taken on the factual observed distribution conditionally on the treatment. For the rest of the article, the expectations will always be taken on the factual observed distribution $\mathcal {D}$. This identification leads to outcome-based estimators (i.e., g-computation estimators [21]):


(3) \begin{eqnarray*}
\tau =& \mathbb {E}_{Y \sim \mathcal {D^{\star }}}[Y(1) - Y(0)] \\
= &\mathbb {E}_{Y \sim \mathcal {D}}[Y|A=1] - \mathbb {E}_{Y \sim \mathcal {D}}[Y| A=0]
\end{eqnarray*}

This equation builds on the conditional expectation of the outcome given the treatment $\mathbb {E}_{\sim \mathcal {D}}[Y|A]$. Outcome-based methods target this quantity conditionally on the covariates, called *response function*:


\begin{eqnarray*}
\text{Response function} \qquad \mu _{a}(x) \overset{\rm def}{=} \mathbb {E}_{Y \sim \mathcal {D}} [Y|X=x, A=a]
\end{eqnarray*}

Given a sample of data and the oracle response functions $\mu _0, \mu _1$, the finite sum version of Eq. (3) leads to an unbiased estimator of the average treatment effect (ATE), written:


(4) \begin{eqnarray*}
\hat{\tau }= \frac{1}{n} \biggl (\sum _{i=1}^n \mu _{1}(x_i) - \mu _{0}(x_i) \biggr )
\end{eqnarray*}

This estimator is an oracle *finite sum* estimator by opposition to the population expression of $\tau$, $\mathbb {E}[\mu _{1}(x_i) - \mu _{0}(x_i)]$, which involves an expectation taken on the full distribution $\mathcal {D}$, which is observable but requires infinite data. For each estimator $\ell$ taking an expectation over $\mathcal {D}$, we use the symbol $\hat{\ell }$ to note its finite sum version. The formulas in Eqs. (2–4) are all partly oracle formulas: they rely on conditional expectations and the response functions, but they give no specific procedures on how to compute or select them. This last point is the topic of our work, described in the next section.

Similarly to the ATE, at the individual level, Eq. (2) links the CATE to statistical quantities:


(5) \begin{eqnarray*}
\tau (x) = \mu _{1}(x) - \mu _{0}(x)
\end{eqnarray*}

#### Robinson decomposition

G-computation is a choice of decomposition of the CATE estimation. Other choices of decomposition exist, such as the R-decomposition [57]. The latter introduces 2 new statistical estimates, the conditional mean outcome and the probability of being treated (known as propensity score [28]):


(6) \begin{eqnarray*}
\text{Conditional mean outcome} \qquad m(x) \overset{\rm def}{=} \mathbb {E}_{Y \sim \mathcal {D}} [Y|X=x]
\end{eqnarray*}

(7) \begin{eqnarray*}
\text{Propensity score} \qquad e(x) \overset{\rm def}{=} \mathbb {P}[A=1|X=x]
\end{eqnarray*}

with these, the outcome (Eq. 1) can be written


(8) \begin{eqnarray*}
& \text{R-decomposition} \qquad y(a) = m(x) + \big ( a - e(x) \big ) \tau (x) + \varepsilon (x; a) \\
& \text{with}\qquad \mathbb {E}[\varepsilon (X; A)|X, A] = 0
\end{eqnarray*}

*m* and *e* are often called *nuisances* [43]. They are unknown and must be estimated from the data.

Both the ATE and CATE formulas (Eqs. 4, 5) and the Robinson decomposition involve conditional expectations—the response functions $\mu _a(x)$ or the nuisances $m(x)$ and $e(x)$. In practice, those are given by statistical models: linear models, random forests, and so on [48, 49].

### Model selection, oracle, and feasible risks

#### Causal model selection

We formalize model selection for causal estimation. Thanks to the outcome model identification (Eq. 2), a given model $f: \mathcal {X} \times \mathcal {A} \rightarrow \mathcal {Y}$—learned from data or built from domain knowledge—induces feasible estimates of the ATE and CATE (Eqs. 4, 5), $\hat{\tau }_{f}$ and $\hat{\tau }_{f}(x)$. However, the g-computation framework presented above is written in terms of “perfect” conditional expectations (oracles), and it does not control an error (e.g., on both populations), as highlighted in Fig. 1. Selection procedures are needed to find the best conditional expectation models.

A selection procedure combines a risk $\ell$, evaluating the quality of a model *f* with observed data *O*, and a splitting strategy of the data to estimate different regressions (nuisances) involved in the risk. Formally, let $\mathcal {F}=\lbrace f: \mathcal {X} \times \mathcal {A} \rightarrow \mathcal {Y}\rbrace$ be a family of such estimators. Our goal is to select the best candidate in this family for the observed dataset *O* using a risk $\ell$:


(9) \begin{eqnarray*}
f^{*}_{\ell } = \mathrm{argmin}_{f \in \mathcal {F}} \ell (f, O)
\end{eqnarray*}

We now detail possible risks $\ell$, risks useful for causal model selection, and how to compute them.

#### The $\tau \text{-risk}$: an oracle error risk

As we would like to target the CATE, the following evaluation risk is natural (also called PEHE [35, 58]):


(10) \begin{eqnarray*}
\tau \text{-risk}(f) \overset{\rm def}{=} \mathbb {E}_{X\sim p(X)}[(\tau (X) - \hat{\tau }_f(X))^2]
\end{eqnarray*}

Given observed data from $p(X)$, the expectation is computed with a finite sum, as in Eq. (4), to give an estimated value $\widehat{\tau \text{-risk}}(f)$. However, this risk is not feasible as the oracles $\tau (x)$ are not accessible with the observed data $(Y, X, A) \sim \mathcal {D}$.

#### Feasible error risks

Table 1 lists *feasible* risks (detailed in Appendix A.4), based on the prediction error of the outcome model and *observable* quantities. These observable quantities, called nuisances, are *e* (propensity score, Eq. 7) and *m* (conditional mean outcome, Eq. 6). We give the definitions as *semi-oracles*, a function of the true unknown nuisances, but later instantiate them with estimated nuisances, noted $\big (\check{e}, \check{m} \big )$. Semi-oracles risks are superscripted with the $^{\star }$ symbol.

**Table 1:** Review of causal risks. The $R\text{-risk}^{*}$ is called $\tau \text{-risk}_R$ in [50].

Risk	Equation	Reference
$\tau \text{-risk}=\text{MSE}(\tau (X), \tau _f(X))$	$\mathbb {E}_{X\sim p(X)}[(\tau (X) - \hat{\tau }_f(X))^2]$	Eq. (10) [35]
$\mu \text{-risk} = \text{MSE}(Y, f(X))$	$\mathbb {E}_{(Y, X, A) \sim \mathcal {D}}\left[(Y-f(X ; A))^2 \right]$	[50]
$\mu \text{-risk}_{IPW}^{*}$	$\mathbb {E}_{(Y, X, A) \sim \mathcal {D}}\left[ \Big ( \frac{A}{e(X)} + \frac{1-A}{1-e(X)} \Big ) (Y-f(X ; A))^2 \right]$	[59]
$\tau \text{-risk}^{\star }_{IPW}$	$\mathbb {E}_{(Y, X, A) \sim \mathcal {D}} \left[ \Big (Y \left( \frac{A}{e(X)} - \frac{1-A}{1-e(X)}\right)-\hat{\tau }_f\left(X\right)\Big )^2 \right]$	[39]
$U\text{-risk}^{*}$	$\mathbb {E}_{(Y, X, A) \sim \mathcal {D}} \big [ \big ( \frac{Y-m\left(X\right)}{A-e\left(X\right)} - \hat{\tau }_f\left(X\right)\big )^{2} \big ]$	[42]
$R\text{-risk}^{*}$	$\mathbb {E}_{(Y, X, A) \sim \mathcal {D}} \big [\big (\left(Y-m\left(X\right)\right) -\left(A-e\left(X\right)\right) \hat{\tau }_f\left(X\right)\big )^{2} \big ]$	[42]

### Model selection procedure

**Figure 2:**
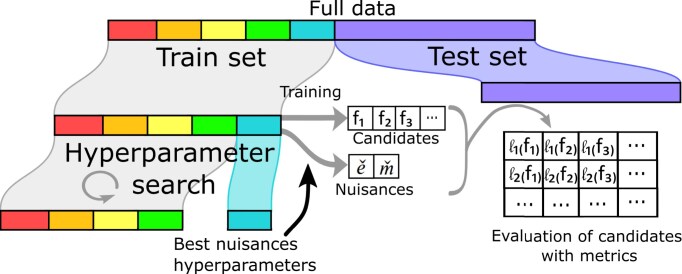
Estimation procedure for causal model selection.

Causal model selection (Eq. 9) may involve estimating various quantities from the observed data: the outcome model *f*, its induced risk as introduced in the previous section, and possibly nuisances required by the risk. Given a dataset with *N* samples, we split train and test sets $(\mathcal {T}, \mathcal {S})$. We fit each candidate estimator $f \in \mathcal {F}$ on $\mathcal {T}$. We also fit the nuisance models $(\check{e}, \check{m})$ on the train set $\mathcal {T}$, setting hyperparameters by a nested cross-validation before fitting the nuisance estimators with these parameters on the full train set. Causal quantities are then computed by applying the fitted candidate estimators $f \in \mathcal {F}$ on the test set $\mathcal {S}$. Finally, we compute the model selection metrics for each candidate model on the test set. This procedure is described in Algorithm 1 and Fig. 2.

**Algorithm 1:** Model selection procedure

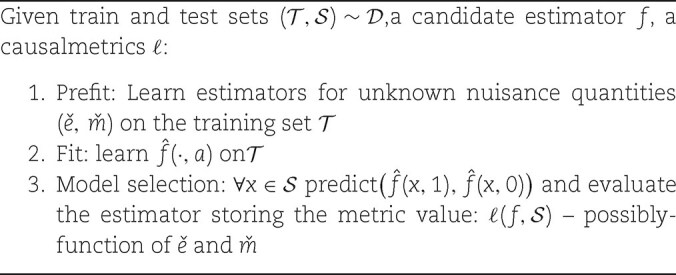

### R-risk as reweighted oracle metric

The $R\text{-risk}$ can be rewritten as a rebalanced $\tau \text{-risk}$.

This rewriting involves reweighted residuals: for each potential outcome, $a \in \lbrace 0; 1\rbrace$, the variance conditionally on *x* is [60]:


\begin{eqnarray*}
\sigma _{y}^{2}(x ; a) \overset{\text{def}}{=} \int _{y}\left(y-\mu _{a}(x)\right)^{2} p(y \mid x=x ; A=a) \, d y
\end{eqnarray*}

Integrating over the population, we get the Bayes squared error, $\sigma ^2_{B}(a) = \int _{\mathcal {X}} \sigma _y^2(x;a) p(x)dx$, and its propensity weighted version, $\tilde{\sigma }^2_{B}(a) = \int _{\mathcal {X}}\sigma _y^2(x;a)\, p(x; a)\, dx$. In case of a purely deterministic link between the covariates, the treatment, and the outcome, these residual terms are null.

**Proposition 1 (*R-*risk as reweighted *τ*-risk)** Given an outcome model *f*, its $R\text{-risk}$ appears as weighted version of its $\tau \text{-risk}$ (proof in Appendix A.5):


(11) \begin{eqnarray*}
R\text{-risk}^{*}(f) & = \int _{x} e(x)\big (1-e(x)\big )\big (\tau (x)-\tau _ {f}(x)\big )^{2} p(x) d x \\
& \quad \,\, + \tilde{\sigma }_B^2(1) + \tilde{\sigma }_B^{2}(0)
\end{eqnarray*}

The $R \text{-risk}$ targets the oracle at the cost of an overlap reweighting and the addition of the reweighted Bayes residuals, which are independent of *f*. In good overlap regions, the weights $e(x) \big (1-e(x) \big )$ are close to $\frac{1}{4}$; hence, the $R \text{-risk}$ is close to the desired gold-standard $\tau \text{-risk}$. For randomized control trials, this weight is constant, making the $R\text{-risk}$ particularly suited for exploring heterogeneity (Appendix A.5).

## Empirical study

We evaluate the following causal metrics, oracle and feasible versions, presented in Table 1:

$\widehat{\mu \text{-risk}}_{IPW}^{*}$ , $\widehat{R\text{-risk}}^{*}$, $\widehat{U\text{-risk}}^{*}$, $\widehat{\tau \text{-risk}_{IPW}}^{*}$, $\widehat{\mu \text{-risk}}$, $\widehat{\mu \text{-risk}}_{IPW}$, $\widehat{R\text{-risk}}$, $\widehat{U\text{-risk}}$, $\widehat{\tau \text{-risk}_{IPW}}$. We benchmark the metrics in a variety of settings: many different simulated data generation processes and 3 semi-simulated datasets.We provide scripts for the simulations [61] and the selection procedure [62].

The simulations, designed to evaluate the effect of the overlap parameter, also explore more diverse and noisy covariate distributions. They cover a diversity of causal settings, such as different ratios of causal effect to background responses, and functional links between covariates, outcome, and treatment.

### Caussim: extensive simulation settings

#### Data generation

We use simulated data, on which the ground-truth causal effect is known. Going beyond prior empirical studies of causal model selection [50, 51], we use many generative processes, which are needed to reach general conclusions (Appendix A.7).

We generate the response functions using the random bases extension, a common method in biostatistics (e.g., functional regression with splines) [63, 64]. By allowing the function to vary at specific knots, we control the complexity of the nonlinear outcome models. We use random approximation of radial basis function (RBF) kernels [65] to generate the outcome and treatment functions. RBF uses the same process as polynomial splines but replaces polynomials with Gaussian kernels. Unlike polynomials, Gaussian kernels have decreasing influences in the input space. This avoids unrealistic divergences of the functions at the ends of the feature space. We generated 1,000 datasets based on these functions, with random overlap parameters. An example shown is in Appendix A.7, Supplementary Fig. S6, and details are in Appendix A.7.

#### Family of candidate estimators

We test model selection across different candidate estimators that approximate imperfectly the data-generating process. To build such estimators, we first use an RBF expansion similar to that used for data generation. We choose 2 random knots and transform the raw data features with a Gaussian kernel. This step is referred to as the featurization. Then, we fit a linear regression on these transformed features. We consider 2 ways of combining these steps for an outcome model; we use common nomenclature [41, 66] to refer to these different meta-learners that differ on how they model, jointly or not, the treated and the nontreated:

- SLearner: A single learner for both populations, taking the treatment as a supplementary covariate.
- SftLearner: A single set of basis functions is sampled at random for both populations, leading to a given feature space used to model both the treated and controls, and then 2 separate different regressors are fitted on this shared representation.
- TLearner: Two completely different learners for each population, hence separate feature representations and regressors.

For the regression step, we fit a ridge regression on the transformed features with 6 different choices of the regularization parameter $\lambda \in [10^{-3}, 10^{-2}, 10^{-1}, 1, 10^{1}, 10^{2}]$, coupled with a TLearner or a SftLearner. We sample 10 different random bases for learning and featurization, yielding a family $\mathcal {F}$ of 120 candidate estimators.

### Semi-simulated datasets

#### Datasets

We also use 3 semi-simulated datasets, adding a known synthetic causal effect to a real—nonsynthetic—health care covariate. ACIC 2016 [45] is based on the Collaborative Perinatal Project [67], a randomized controlled trial studying infants’ developmental disorders containing 4,802 indivduals and 55 features. We used 770 dataset instances: 10 random seeds for each of the 77 simulated settings for the treatment and outcomes. ACIC 2018 [68] simulated treatment and outcomes for the Linked Births and Infant Deaths Database (LBIDD) [69] with $D=177$ covariates. We used all 432 datasets of size $N=5\, 000$. Twins [70] is an augmentation of real data on twin births and mortality rates [71]. There are $N=11\, 984$ samples, and $D=50$ covariates for which we simulated 1,000 different treatment allocations. Appendix A.7 gives dataset details.

#### Family of candidate estimators

For these 3 datasets, the family of candidate estimators includes gradient boosting trees for both the response surfaces and the treatment (scikit-learn HistGradientBoostingRegressor HistGradientBoostingClassifier [72]) with SLearner, learning rate in $\lbrace 0.01, 0.1, 1\rbrace$, and maximum number of leaf nodes in $\lbrace 25, 27, 30, 32, 35, 40\rbrace$, resulting in a family of size 18.

#### Nuisance estimators

Drawing from the Targetde Maximum Likelihood Estimation literature that uses a combination of flexible machine learning methods [37], we model the nuisances $\check{e}$ (respectivley $\check{m}$) with a meta-learner: a stacked estimator of ridge and boosting classifiers (respectively regressions) (hyperparameter selection in Appendix A.7).

### Measuring overlap between treated and non treated

Good overlap between treated and control populations is crucial for causal inference (Appendix A.3, assumption 3). We introduce the normalized total variation (NTV), a divergence based on the propensity score summarizing the overlap between both populations (Appendix A.6).

## Results: Factors Driving Good Model Selection

### The $R\text{-risk}$ is the best metric on average

**Figure 3:**
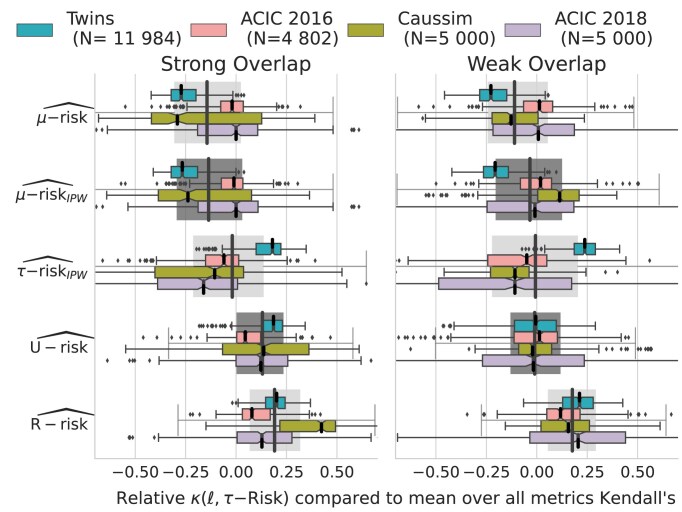
The *R*-risk is the best metric: relative Kendall’s $\tau$ agreement with $\tau \text{-risk}$. Strong and weak overlap correspond to the first and last tertiles of the overlap distribution measured with normalized total variation (Appendix A.6). Appendix A.7 presents the same results by adding semi-oracle risks in Supplementary Fig. S7, measured with absolute Kendall in Supplementary Fig. S7 and with $\tau \mathrm{-risk}$ gains in Supplementary Fig. S9. Supplementary Table S3 gives the median and interquartile range of the relative Kendall.

Figure 3 shows the agreement between the ideal ranking of outcome models given the oracle $\tau \text{-risk}$ and the different feasible causal metrics. We measure this agreement with relative Kendall tau $\kappa$ (see Appendix A.7, Eq. 20) [73]. To remove the variance across datasets (some datasets lead to easier model selection than others), we report values for 1 metric relative to the mean of all metrics for a given dataset instance: $\text{Relative} \, \kappa (\ell ,\tau \mathrm{{-risk}})= \kappa (\ell ,\tau \mathrm{{-risk}}) - mean_{\ell }\big (\kappa (\ell ,\tau \mathrm{{-risk}})\big )$. Given the importance of overlap in how well metrics approximate the oracle $\tau \text{-risk}$, we separate strong and weak overlap.

Among all metrics, the classical MSE (i.e., factual $\mu \text{-risk}$) is worse, and reweighting it with a propensity score ($\mu \text{-risk}_{IPW}$) does not bring much improvement. The $R\text{-risk}$, which includes a model of mean outcome and propensity scores, leads to the best performances. Interestingly, the $U\text{-risk}$, which uses the same nuisances, deteriorates in weak overlap, probably due to variance inflation when dividing by extreme propensity scores.

Beyond rankings, the differences in terms of absolute ability to select the best model are large: the R-risk selects a model with a $\tau \text{-risk}$ only 1% higher than the best possible candidate for strong overlap on Caussim, but selecting with the $\mu \text{-risk}$ or $\mu \text{-risk}_{IPW}$—as per machine learning practice—leads to 10% excess risk, and using $\tau \text{-risk}_{IPW}$—as in some causal inference methods [74, 75]—leads to 100% excess risk (Appendix A.7, Supplementary Fig. S9). Across datasets, the $R\text{-risk}$ consistently decreases the risk compared to the $\mu \text{-risk}$: from 0.1% to 1% on ACIC 2016, 1% from to 20% on ACIC 2018, and 0.05% from to 1% on Twins.

### Model selection is harder for low population overlap

**Figure 4:**
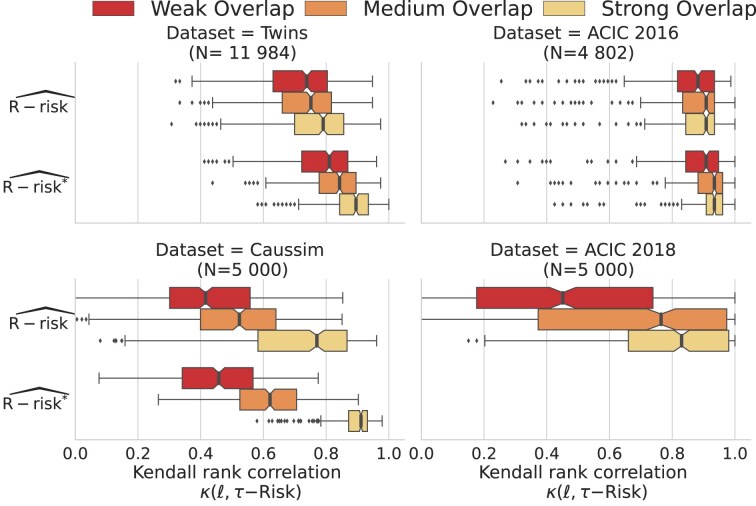
Model selection is harder for low population overlap: Kendall’s $\tau$ agreement with $\tau \text{-risk}$. Strong, medium, and weak overlap are the tertiles of the overlap measured with NTV (Appendix A.6, Eq. 17). Appendix A.7 presents results for all metrics in Supplementary Fig. S8 in absolute Kendall and continuous overlap values in Supplementary Fig. S8.

Model selection for causal inference becomes more and more difficult with increasingly differently treated and control populations (Fig. 4). The absolute Kendall’s coefficient correlation with $\tau \text{-risk}$ drops from 0.9 (excellent agreement with oracle selection) to 0.6 on both Caussim and ACIC 2018 (Appendix A.7, Supplementary Fig. S8).

### Nuisances can be estimated on the same data as outcome models

**Figure 5:**
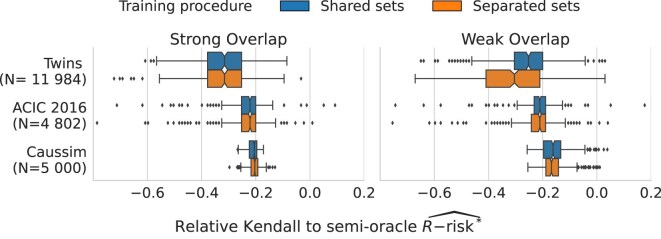
Nuisances can be estimated on the same data as outcome models: Results for the R-risk are similar between the shared nuisances/candidate set and the separated nuisances set procedures. Appendix A.7, Supplementary Fig. S8 details results for all metrics.

Using the train set $\mathcal {T}$ both to fit the candidate estimator and the nuisance estimates is a form of double dipping that can lead errors in nuisances correlated to that of outcome models [42]. In theory, these correlations can bias model selection and, strictly speaking, push to split a third separated dataset—a “nuisance set”—to fit the nuisance models. The drawback is that it depletes the data available for model estimation and selection. However, Fig. 5 shows no substantial difference between a procedure with a separated nuisance set and the simpler shared nuisance candidate set procedure.

Empirically, the best split is 90 %/10 %: using 90% of the data to estimate both the nuisances and candidates, then computing the risks on the remaining test set for model selection (experiments in Appendix A.8).

### Stacked models are good overall estimators of nuisances

**Figure 6:**
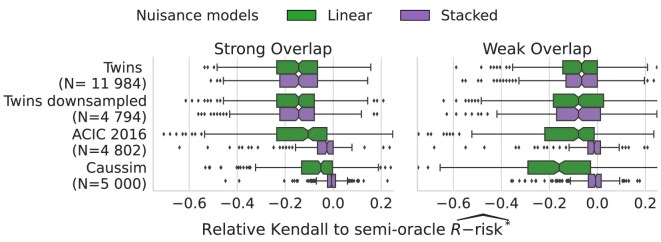
Stacked models are good overall estimators of the nuisances: results are shown only for the R-risk; Appendix A.7, Supplementary Fig. S12 details every metric. For Twins, where the true propensity model is linear, stacked and linear estimations of the nuisances perform equivalently, even for a downsampled version (*N* = 4,794).

Stacked nuisance estimators (boosting and linear) lead to feasible metrics with close performances to the oracle ones: the corresponding estimators recover well enough the true nuisances. One may wonder if simpler models for the nuisance could be useful, particularly in data-poor settings or when the true models are linear. Figure 6 compares causal model selection estimating nuisances with stacked estimators or the linear model. It comprises the Twins data, where the true propensity model is linear, and a downsampled version of these data, to study a situation favorable to linear models. In these settings, stacked and linear estimations of the nuisances perform equivalently. Detailed analysis (Appendix A.7, Supplementary Fig. S13) confirms that using adaptive models, as built by stacking linear models and gradient-boosted trees, suffices to estimate nuisance.

### R-risk is robust to a wide range of effect ratio values

**Figure 7:**
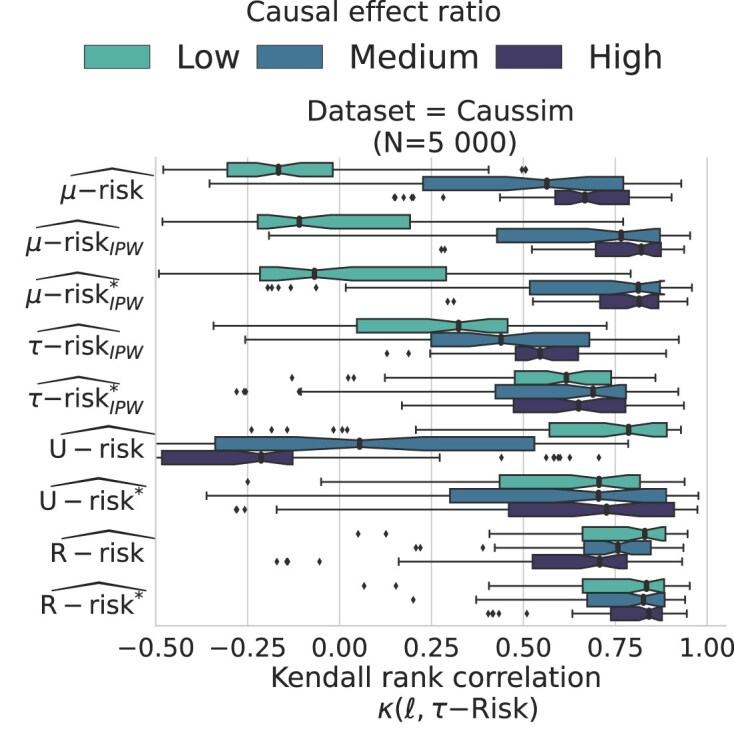
R-risk is robust to a wide range of effect ratios: Kendall’s $\tau$ agreement with $\tau$-risk. Strong, medium, and weak causal effect ratios are the tertiles of the absolute ratio causal effect to baseline response. $\Delta _{\mu }$: low $[0.04;2.86[$, medium $[2.86; 16.65[$, high $[16.65;206.53[$. Appendix A.9 details this simulation.

Beyond overlap, we study for Caussim simulations, the effect on model selection of a different causal effect ratio to baseline. We vary the empirical mean absolute difference between the causal effect and the baseline, $\Delta _{\mu } = \frac{1}{N} \sum _{i=1}^N \big | \frac{\mu _{1}(x_i) - \mu _{0}(x_i)}{\mu _{0}(x_i) + \mu _{1}(x_i) - \frac{1}{N} \sum _{j=1}^N \mu _0(x_j) + \mu _1(x_j)} \big |$, covering a ratio range from 0.04 to 206 ($\text{median}=9.1$). Appendix A.9 details this setup as well as an alternative measure of effect ratio. Figure 7 shows that for high values of the ratio, R-risk is outperformed by the $\mu \text{-risk}_{IPW}$ and the $\tau \text{-risk}_{IPW}$. However, on average, the R-risk is still the better risk.

## Discussion and Conclusion

### Nuisance models: more gain than pain

Predictive models are increasingly used to reason about treatment effects, for instance, in precision medicine to drive individualized decisions. Our results highlight that they should be selected, validated, and tuned using different procedures and error measures than those classically used to assess prediction. Rather, selecting the best outcome model according to the $R\text{-risk}$ (Appendix A.4, Eq. Definition 5) leads to more valid causal estimates on average. Estimating the $R\text{-risk}$ requires a more complex procedure than standard cross-validation used in machine learning, for example: it involves fitting nuisance models necessary for model evaluation. Our results show that these can be learned on the same set of data as the outcome models evaluated. The nuisance models must be well estimated (Fig. 6). Our results show that using a flexible stacking-based family of estimators for nuisance models suffices for good model selection. To select propensity score models, we used the Brier score, minimized by the true individual probability. An easy mistake is to use calibration errors popular in machine learning [76–79] as these select not for the individual posterior probability but for an aggregate error rate [80].

### More $R\text{-risk}$ to select models driving decisions

Increasingly complex prediction models integrating richer medical data have flourished because their predictions can be easily demonstrated and validated on left-out data. But using them to underpin a decision on whether to treat or not requires more careful validation, using a metric accounting for the putative intervention, the $R\text{-risk}$. On average, the $R\text{-risk}$ brings a sizable benefit to select the most adequate model, even when model development is based on treated and untreated populations with few differences, as in randomized controlled trials. Our conclusions are that without prior knowledge, the R-risk is a good default. However, there is much remaining variation, and the R-risk will not be optimal for every situation. We have identified one such specific situation: when the causal effect is large compared to the variation of the baseline effect, the $\mu \text{-risk}_{IPW}$ performs slightly better.

To facilitate better model selection, we provide Python code [62]. This model selection procedure puts no constraints on the models used to build predictive models: it opens the door to evaluating a wide range of models, from gradient boosting to convolutional neural network or language models.

## Availability of Source Code and Requirements

### Source code to replicate the experiments of the study

The following repository is self-contained to allow anyone to replicate all experiments of the study. It contains different codes to cover the full content of the article and thus requires a bit of work to install and run the code.

- Project name: Caussim
- Project homepage: [61]
- Operating system(s): Platform independent
- Programming language: Python
- License: BSD 3-Clause License

### Standalone source code for the selection procedure

Code for cross-validation to select the best predictive model to guide decision on potential intervention. The procedure implemented here is described in detail in Algorithm 1. It serves as a simple plug-and-play code for a user who would want to use our procedure.

- Project name: Causal Model Selection
- Project homepage: [62]
- Operating system(s): Platform independent
- Programming language: Python
- License: BSD 2-Clause License

## Supplementary Material

giaf016_Supplemental_File

giaf016_GIGA-D-24-00240_Original_Submission

giaf016_GIGA-D-24-00240_Revision_1

giaf016_GIGA-D-24-00240_Revision_2

giaf016_Response_to_Reviewer_Comments_Original_Submission

giaf016_Response_to_Reviewer_Comments_Revision_1

giaf016_Reviewer_1_Report_Original_Submission Paola Lecca -- 8/12/2024

giaf016_Reviewer_1_Report_Revision_1 Paola Lecca -- 11/24/2024

giaf016_Reviewer_2_Report_Original_Submission Szymon Jaroszewicz -- 8/16/2024

giaf016_Reviewer_2_Report_Revision_1 Szymon Jaroszewicz -- 12/8/2024

## Data Availability

The semi-simulated and simulated datasets used for the experiments are available at the following URLs. Detailed explanations on how to generate the datasets are available in the readme of the data section of our GitHub [61]. ACIC 2016: dataset provided through R package aciccomp2016 [45] ACIC 2018: scaling subset from the Synapse repository [81] Twins: dataset from [70]; our version is on a Zenodo repository [82] Caussim dataset: generated dataset with the source code [61] The result data generated by the experiments are available at a dedicated Zenodo repository [83]. We provide the data for the simulations and the semi-simulated datasets to allow an easy replication of the main graphic (Fig. 3) in the Results section.

## Additional Files

**A.1 Variability of ATE estimation on ACIC 2016**.

**A.2 Prior work: model selection for outcome modeling (g-computation)**.

**A.3 Causal assumptions**.

**A.4 Definitions of feasible risks**.

**A.5 Proofs: Links between feasible and oracle risks**.

**A.6 Measuring overlap**.

**A.7 Experiments**.

**A.8 Data split choices**.

**A.9 Sensitivity of the results to a different ratio of causal effect to baseline response**.

**Supplementary Fig. S1**. Different outcome models lead to different estimation errors on the average treatment effects on 77 classic simulations with known true causal effect [45]. The different models are ridge regression and random forests with different hyperparameters (details in A.1). The different configurations are plotted as a function of increasing difference between treated and untreated population—see subsection. There is no systematic best performer; data-driven model selection is important.

**Supplementary Fig. S2**. (a) Without calibration, estimation of NTV is not trivial, even for boosting models. (b) Calibrated classifiers are able to recover the true normalized total variation for all datasets where it is available.

**Supplementary Fig. S3**. NTV recovers well the overlap settings described in the ACIC paper [45].

**Supplementary Fig. S4**. Good correlation between overlap measured as normalized total variation and maximum mean discrepancy (200 sampled Caussim datasets).

**Supplementary Fig. S5**. Maximal value of inverse propensity weights increases exponentially with the overlap as measure by normalized total variation.

**Supplementary Fig. S6**. Example of the simulation setup in the input space with 2 knots (i.e., basis functions). The top panel shows the observations in feature space, while the bottom panel displays the 2 response surfaces on a 1-dimensional cut along the black lines drawn on the top panel.

**Supplementary Fig. S7**. The R-risk is the best metric on average: relative Kendall’s $\tau$ agreement with $\tau$-risk. Strong and weak overlap correspond to the first and last tertiles of the overlap distribution measured with normalized total variation (Eq. 17).

**Supplementary Fig. S8**. Agreement with $\tau$-risk ranking of methods function of overlap violation. The lines represent medians, estimated with a lowess. The transparent bands denote the 5% and 95% confidence intervals.

**Supplementary Fig. S9**. Metric performances by normalized tau-risk distance to the best method selected with $\tau$-risk. All nuisances are learned with the same estimator stacking gradient boosting and ridge regression. Dotted and plain lines correspond to 60% lowess quantile estimates. This choice of quantile allows one to see better the oracle metric lines for which outliers with a value of 0 distort the curves.

**Supplementary Fig. S10**. Results are similar between the shared nuisances/candidate set and the separated nuisances set procedure. The experience has not been run on the full metrics for Caussim due to computation costs.

**Supplementary Fig. S11**. Low population overlap hinders causal model selection for all metrics: Kendall’s $\tau$ agreement with $\tau$-risk. Strong, medium, and weak overlap correspond to the tertiles of the overlap distribution measured with normalized total variation (Eq. 17).

**Supplementary Fig. S12**. Learning the nuisances with stacked models (linear and gradient boosting) is important for successful model selection with R-risk. For the Twins dataset, there is no improvement for stacked models compared to linear models because of the linearity of the propensity model.

**Supplementary Fig. S13**. Flexible models are performant in recovering nuisances in the downsampled Twins dataset. The propensity score is linear in this setup, making it particularly challenging for flexible models compared to linear methods.

**Supplementary Fig. S14**. Kendall correlation coefficients for each causal metric. Each (color, shape) pair indicates a different (treatment ratio, seed) of the generation process.

**Supplementary Fig. S15**. (a) For CATE, a train/test ratio of 0.9/0.1 appears to be a good trade-off. (b) For ATE, there is a small signal pointing also to 0.9/0.1 (K = 10) for ATE. Experiences on 10 replications of all 78 instances of the ACIC 2016 data.

**Supplementary Fig. S16**. R-risk is robust to a wide range of effect variance: Kendall’s $\tau$ agreement with $\tau$-risk. Strong, medium, and weak causal effect variance are the tertiles of the ratio of causal effect variance to the mean outcome variance: low [0; 0.04[, medium [0.04; 1.04[, and high [1.04; 11.08[.

**Supplementary Table S1**. Hyperparameter grid used for ACIC 2016 ATE variability.

**Supplementary Table S2**. Hyperparameter grid used for nuisance models.

**Supplementary Table S3**. Values of relative $\kappa (\ell$, $\tau$-risk) compared to the mean over all metrics Kendall’s τ as shown in the boxplots of Figure 3.

**Supplementary Table S4**. Distribution of the causal effect ratio $\Delta _\mu$ for the experiment on the causal effect ratio; results in Figure 7.

**Supplementary Table S5**. Statistical summary of the causal effect variance.

## Abbreviations

ATE: average treatment effect; CATE: conditional average treatment effect; LBIDD: Linked Births and Infant Deaths Database; MSE: mean squared error; NTV: normalized total variation; RBF: radial basis function.
