# Supplementary material for: How to select predictive models for decision-making or causal inference
Source: Gigascience. 2025 Mar 21;14:giaf016. doi: 10.1093/gigascience/giaf016 (PMC11927402; doi:10.1093/gigascience/giaf016)
Supplement: giaf016_Supplemental_File [file giaf016_supplemental_file.pdf]

A Additional files for paper : How to select predictive models for decision-making or causal inference? Doutreligne, Matthieu and Varoquaux, Gaël, 2025

A.1 Variability of ATE estimation on ACIC 2016

Figure 8 shows ATE estimations for six different models used in g-computation estimators on the 76 configurations of the ACIC 2016 dataset. Outcome models are fitted on half of the data and inference is done on the other half –ie. train/test with a split ratio of 0.5. For each configuration, and each model, this train test split was repeated ten times, yielding non parametric variance estimates [84]. Figure 8 shows large variations obtained across different outcome estimators on semi-synthetic datasets [45]. Flexible models such as random forests are doing well in most settings except when treated and untreated populations differ noticeably, in which case a linear model (ridge) is to be preferred. However random forests with different hyper-parameters (max depth= 2) yield poor estimates. A simple rule of thumb such as preferring flexible models does not work in general; model selection is needed.

Outcome models are implemented with scikit-learn [72] and the following hyper-parameters:

Outcome Model	Hyper-parameters grid
Random Forests	Max depth: [2, 10]
Ridge regression without treatment interaction	Ridge regularization: [0.1]
Ridge regression with treatment interaction	Ridge regularization: [0.1]

Table 2. Hyper-parameters grid used for ACIC 2016 ATE variability

A.2 Prior work : model selection for outcome modeling (g-computation)

A natural way to select a predictive model for causal inference would be an error measure between a causal quantity such as the CATE and models' estimate. But such error is not a "feasible" risk: it cannot be computed solely from observed data and requires oracle knowledge.

Simulation studies of causal model selection. Using eight simulations setups from [38], where the oracle CATE is known, [50] compare four causal risks, concluding that for CATE estimation the best model-selection risk is the so-called R-risk [42] –def. 5, below. Their empirical results are clear for randomized treatment allocation but less convincing for observational settings where both simple MSE –μ-risk(f) def. 1– and reweighted MSE –μ-risk$_{IPW}$ def. 2– appear to perform better than R-risk on half of the simulations. Another work [51] studied empirically both MSE and reweighted MSE risks on the semi-synthetic ACIC 2016 datasets [45], but did not include the R-risk.

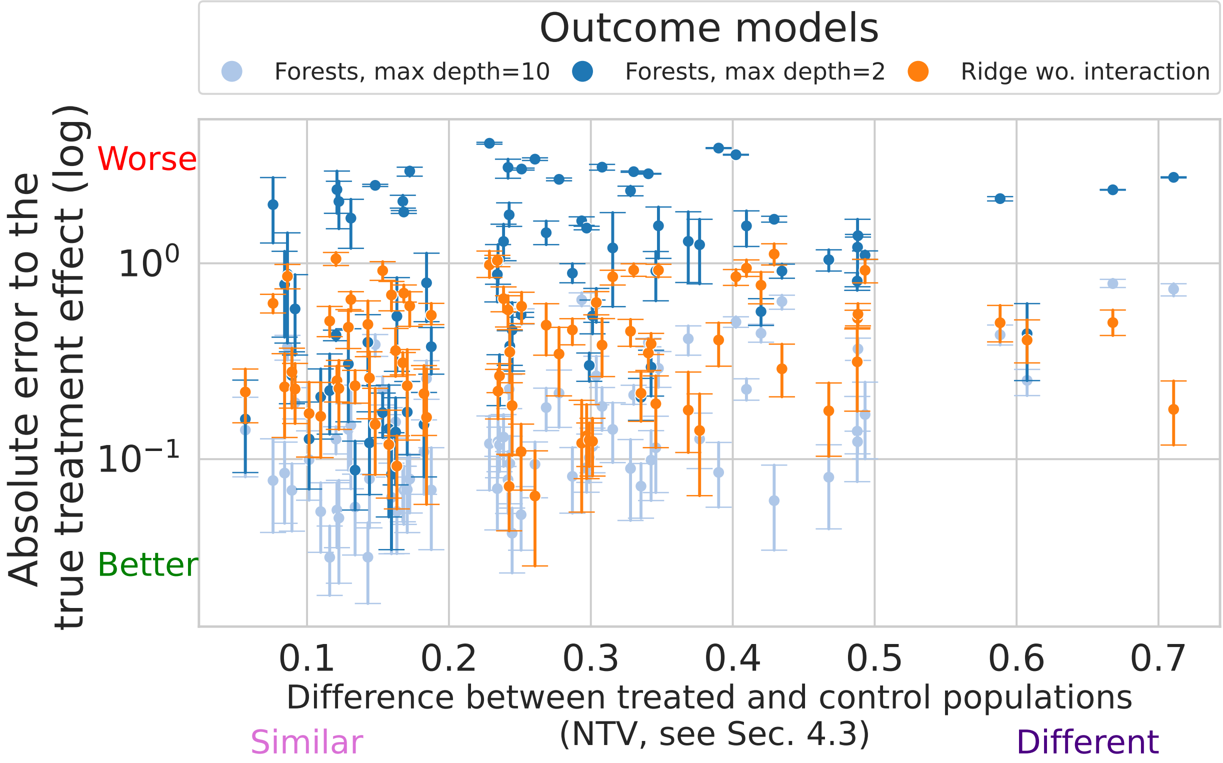

Figure 8. Different outcome models lead to different estimation errors on the Average Treatment Effects, on 77 classic simulations with known true causal effect [45]. The different models are ridge regression and random forests with different hyper-parameters (details A.1). The different configurations are plotted as a function of increasing difference between treated and untreated population –see subsection . There is no systematic best performer; data-driven model selection is important.

We complete these prior empirical work by studying a wider variety of data generative processes and varying the influence of overlap, an important parameter of the data generation process which makes a given causal metric appropriate [85]. We also study how to best adapt cross-validation procedures to causal metrics which themselves come with models to estimate.

Theoretical studies of causal model selection. Several theoretical works have proposed causal model selection procedures that are *consistent*: select the best model in a family given asymptotically large data. These work rely on introducing a CATE estimator in the testing procedure: matching [86], an IPW estimate [75], a doubly robust estimator [87], or debiasing the error with influence functions [51]. However, for theoretical guarantees to hold, the test-set correction needs to converge to the oracle: it needs to be flexible enough –well-posed– and asymptotic data. From a practical perspective, meeting such requirements implies having a good CATE estimate, thus having solved the original problem of causal model selection.

Statistical guarantees on causal estimation procedures. Much work in causal inference has focused on procedures that guarantee asymptotically consistent estimators, such as Targeted Machine Learning Estimation (TMLE) [36, 37] or Double Machine Learning [43]. Here also, theories require asymptotic regimes and models to be *well-specified*.

By contrast, without assuming that estimators are well specified, there exists an upper bound on the oracle error to the CATE (τ-risk) that involves the error on the outcome and the similarity of the distributions of treated and control patients [88]. However, they use this upper bound for model optimization, and do not give insights on model selection. In addition, for hyperparameter selection, they rely on a plugin estimate of the τ-risk built with counterfactual nearest neighbors, which has been shown ineffective [50]. An interesting direction is taken in [89] where the authors derive convergence rates for orthogonal losses such as the R-loss, or the DR-Loss without assuming well-specification of the model for target parameter.

A.3 Causal assumptions

We assume the following four assumptions, referred as strong ignorability and necessary to assure identifiability of the causal estimands with observational data [56]:

Assumption 1 (Consistency) *The observed outcome is the potential outcome of the assigned treatment:*

$$Y = A\,Y(1) + (1 - A)\,Y(0)$$

Here, we assume that the intervention A has been well defined. This assumption focuses on the design of the experiment. It clearly states the link between the observed outcome and the potential outcomes through the intervention [24].

Assumption 2 (Unconfoundedness)

$$\{Y(0), Y(1)\} \perp\!\!\!\perp A|X$$

This condition –also called ignorability– is equivalent to the conditional independence on $e(X)$ [28]: $\{Y(0), Y(1)\} \perp\!\!\!\perp A|e(X)$.

Assumption 3 (Overlap, also known as Positivity))

$$\eta < e(x) < 1 - \eta \quad \forall x \in \mathcal{X} \text{ and some } \eta > 0$$

The treatment is not perfectly predictable. Or with different words, every patient has a chance to be treated and not to be treated. For a given set of covariates, we need examples of both to recover the ATE.

As noted by [85], the choice of covariates X can be viewed as a trade-off between these two central assumptions. A bigger covariates set generally reinforces the ignorability assumption. In the contrary, overlap can be weakened by large $\mathcal{X}$ because of the potential inclusion of instruments: variables only linked to the treatment which could lead to arbitrarily small propensity scores.

Assumption 4 (Generalization) *The training data on which we build the estimator and the test data on which we make the estimation are drawn from the same distribution $\mathcal{D}^*$, also known as the "no covariate shift" assumption [90].*

A.4 Definitions of feasible risks

Definition 1 (Factual μ-risk) *[60] This is the usual Mean Squared Error on the target y. It is what is typically meant by "generalization error" in supervised learning:*

$$\mu\text{-}risk(f) = \mathbb{E}\left[(Y - f(X;A))^2\right]$$

Definition 2 (μ-risk$^{\star}_{IPW}$) *[58] Let the inverse propensity weighting function $w(x,a) = \frac{a}{e(x)} + \frac{1-a}{1-e(x)}$, we define the semi-oracle Inverse Propensity Weighting risk,*

$$\mu\text{-}risk^{\star}_{IPW}(f) = \mathbb{E}\left[\left(\frac{A}{e(X)} + \frac{1-A}{1-e(X)}\right)(Y - f(X;A))^2\right]$$

Definition 3 **(τ-risk$^{\star}_{IPW}$)** *[39] The CATE $\tau(x)$ can be estimated with a regression against inverse propensity weighted outcomes [74, 75, 39], the τ-risk$_{IPW}$.*

$$\tau\text{-risk}^{\star}_{IPW}(f) = \mathbb{E}\left[\left(Y\frac{A - e(X)}{e(X)(1 - e(X))} - \tau_f(X)\right)^2\right]$$

Definition 4 **(U-risk*)** *[41, 42] Based on the Robinson decomposition –eq. 8, the U-learner uses the $A - e(X)$ term in the denominator. The derived risk is:*

$$U\text{-risk}^{\star}(f) = \mathbb{E}\left[\left(\frac{Y - m(X)}{A - e(X)} - \tau_f(X)\right)^2\right]$$

Note that extreme propensity weights in the denominator term might inflate errors in the numerator due to imperfect estimation of the mean outcome m.

Definition 5 **(R-risk*)** *[42, 50] The R-risk also uses two nuisance m and e:*

$$R\text{-risk}^{\star}(f) = \mathbb{E}\big[\big((Y - m(X)) - (A - e(X))\,\tau_f(X)\big)^2\big]$$

It is also based on the Robinson decomposition –eq. 8.

A.5 Proofs: Links between feasible and oracle risks

Reformulation of the R-risk as reweighted τ-risk

Proposition 1 **(R-risk as reweighted τ-risk)** ***Proof 1*** *We consider the R-decomposition: [57],*

$$y(a) = m(x) + (a - e(x))\tau(x) + \varepsilon(x; a) \tag{12}$$

Where $\mathbb{E}[\varepsilon(X; A)|X, A] = 0$ We can use it as plug in the R-risk formula:

$$\begin{aligned}
R\text{-risk}(f) &= \int_{\mathcal{Y}\times\mathcal{X}\times\mathcal{A}} [(y - m(x)) - (a - e(x))\tau_f(x)]^2 p(y; x; a)\,dydxda \\
&= \int_{\mathcal{Y}\times\mathcal{X}\times\mathcal{A}} \left[(a - e(x))\tau(x) + \varepsilon(x; a) - (a - e(x))\tau_f(x)\right]^2 p(y; x; a)\,dydxda \\
&= \int_{\mathcal{X}\times\mathcal{A}} (a - e(x))^2(\tau(x) - \tau_f(x))^2 p(x; a)\,dxda \\
&+ 2\int_{\mathcal{Y}\times\mathcal{X}\times\mathcal{A}} (a - e(x))(\tau(x) - \tau_f(x)) \int_{\mathcal{Y}} \varepsilon(x; a)p(y \mid x; a)\,dy\,p(x; a)\,dxda \\
&+ \int_{\mathcal{X}\times\mathcal{A}} \int_{\mathcal{Y}} \varepsilon^2(x; a)p(y \mid x; a)\,dy\,p(x; a)\,dxda
\end{aligned}$$

The first term can be decomposed on control and treated populations to force $e(x)$ to appear:

$$\begin{aligned}
&\int_{\mathcal{X}} (\tau(x) - \tau_f(x))^2 \left[e(x)^2 p(x; 0) + (1 - e(x))^2 p(x; 1)\right] dx \\
&= \int_{\mathcal{X}} (\tau(x) - \tau_f(x))^2 \left[e(x)^2(1 - e(x))p(x) + (1 - e(x))^2 e(x)p(x)\right] dx \\
&= \int_{\mathcal{X}} (\tau(x) - \tau_f(x))^2(1 - e(x))e(x)[1 - e(x) + e(x)]p(x)\,dx \\
&= \int_{\mathcal{X}} (\tau(x) - \tau_f(x))^2(1 - e(x))e(x)p(x)\,dx.
\end{aligned}$$

The second term is null since, $\mathbb{E}[\varepsilon(x, a)|X, A] = 0$.
The third term corresponds to the modulated residuals : $\tilde{\sigma}^2_B(0) + \tilde{\sigma}^2_B(1)$

Interesting special cases

Randomization special case. If the treatment is randomized as in RCTs, $p(A = 1 \mid X = x) = p(A = 1) = p_A$, thus μ-risk$_{IPW}$ takes a simpler form:

$$\mu\text{-risk}_{IPW} = \mathbb{E}_{(Y,X,A)\sim\mathcal{D}}\left[\left(\frac{A}{p_A} + \frac{1 - A}{1 - p_A}\right)(Y - f(X; A))^2\right]$$

However, we still can have large differences between τ-risk and μ-risk$_{IPW}$ coming from heterogeneous errors between populations as shown experimentally in [50] and our results below.

Concerning the R-risk, replacing $e(x)$ by its randomized value p_A in Proposition 1 yields the oracle τ-risk up to multiplicative and additive constants:

$$R\text{-risk} = p_A\,(1 - p_A)\,\tau\text{-risk} \;+\; (1 - p_A)\,\sigma^2_B(0) \;+\; p_A\,\sigma^2_B(1)$$

Thus, selecting estimators with R-risk* in randomized setting controls the τ-risk. This explains the strong performances of R-risk in randomized setups [50] and is a strong argument to use it to estimate heterogeneity in RCTs.

Oracle Bayes predictor. If we have access to the oracle Bayes predictor for the outcome ie. $f(x, a) = \mu(x, a)$, then all risks are equivalent up to the residual variance:

$$\tau\text{-risk}(\mu) = \mathbb{E}_{X \sim p(X)}[(\tau(X) - \tau_\mu(X))^2] = 0 \tag{13}$$

$$\begin{aligned} \mu\text{-risk}(\mu) &= \mathbb{E}_{(Y,X,A) \sim p(Y;X;A)}[(Y - \mu_A(X))^2] \\ &= \int_{\mathcal{X},\mathcal{A}} \varepsilon(x,a)^2 p(a \mid x)\, p(x)\, dx\, da \leq \sigma_B^2(0) + \sigma_B^2(1) \end{aligned} \tag{14}$$

$$\mu\text{-risk}_{IPW}(\mu) = \sigma_B^2(0) + \sigma_B^2(1) \tag{15}$$

$$R\text{-risk}(\mu) = \tilde{\sigma}_B^2(0) + \tilde{\sigma}_B^2(1) \leq \sigma_B^2(0) + \sigma_B^2(1) \quad \text{from Proposition 1} \tag{16}$$

Thus, differences between causal risks only matter in finite sample regimes. Universally consistent learners converge to the Bayes risk in asymptotic regimes, making all model selection risks equivalent. In practice however, choices must be made in non-asymptotic regimes.

A.6 Measuring overlap

Motivation of the Normalized Total Variation. Overlap is often assessed by comparing visually population distributions as in Figure 1 or computing standardized difference on each feature [91, 29]. While these methods are useful to decide if positivity holds, they do not yield a single measure. Rather, we compute the divergence between the population covariate distributions $\mathbb{P}(X|A = 0)$ and $\mathbb{P}(X|A = 1)$ [85, 88]. Computing overlap when working only on samples of the observed distribution, outside of simulation, requires a sophisticated estimator of discrepancy between distributions, as two data points never have the same exact set of features. Maximum Mean Discrepancy [92] is typically used in the context of causal inference [60, 88]. However it needs a kernel, typically Gaussian, to extrapolate across neighboring observations. We prefer avoiding the need to specify such a kernel, as it must be adapted to the data which is tricky with categorical or non-Gaussian features, a common situation for medical data.

For simulated and some semi-simulated data, we have access to the probability of treatment for each data point, which sample both densities in the same data point. Thus, we can directly use distribution discrepancy measures and rely on the Normalized Total Variation (NTV) distance to measure the overlap between the treated and control propensities. This is the empirical measure of the total variation distance [93] between the distributions, $TV(\mathbb{P}(X|A = 1), \mathbb{P}(X|A = 0))$. As we have both distribution sampled on the same points, we can rewrite it a sole function of the propensity score, a low dimensional score more tractable than the full distribution $\mathbb{P}(X|A)$:

$$\widehat{NTV}(e, 1-e) = \frac{1}{2N} \sum_{i=1}^{N} \Big| \frac{e(x_i)}{p_A} - \frac{1 - e(x_i)}{1 - p_A} \Big| \tag{17}$$

Formally, we can rewrite NTV as the Total Variation distance between the two population distributions. For a population $O = (Y(A), X, A) \sim \mathcal{D}$:

$$\begin{aligned} NTV(O) &= \frac{1}{2N} \sum_{i=1}^{N} \Big| \frac{e(x_i)}{p_A} - \frac{1 - e(x_i)}{1 - p_A} \Big| \\ &= \frac{1}{2N} \sum_{i=1}^{N} \Big| \frac{P(A = 1|X = x_i)}{p_A} - \frac{P(A = 0|X = x_i)}{1 - p_A} \Big| \end{aligned}$$

Thus NTV approximates the following quantity in expectation over the data distribution $\mathcal{D}$:

$$\begin{aligned} NTV(\mathcal{D}) &= \int_{\mathcal{X}} \Big| \frac{p(A = 1|X = x)}{p_A} - \frac{p(A = 0|X = x)}{1 - p_A} \Big| p(x) dx \\ &= \int_{\mathcal{X}} \Big| \frac{p(A = 1, X = x)}{p_A} - \frac{p(A = 0, X = x)}{1 - p_A} \Big| dx \\ &= \int_{\mathcal{X}} |p(X = x|A = 1) - p(X = x|A = 0)| dx \end{aligned}$$

For countable sets, this expression corresponds to the Total Variation distance between treated and control populations covariate distributions : $TV(p_0(x), p_1(x))$.

Measuring overlap without the oracle propensity scores:. For ACIC 2018, or for non-simulated data, the true propensity scores are not known. To measure overlap, we rely on flexible estimations of the Normalized Total Variation, using gradient boosting trees to approximate the propensity score. Empirical arguments for this plug-in approach is given in Figure 9.

Empirical arguments. We show empirically that NTV is an appropriate measure of overlap by :

- Comparing the NTV distance with the MMD for Caussim which is gaussian distributed in Figure 11,
- Verifying that setups with penalized overlap from ACIC 2016 have a higher total variation distance than unpenalized setups in Figure 10.
- Verifying that the Inverse Propensity Weights extrema (the inverse of the ν overlap constant appearing in the overlap Assumption 3) positevely correlates with NTV for Caussim, ACIC 2016 and Twins in Figure 12. Even if the same value of the maximum IPW could lead to different values of NTV, we expect both measures to be correlated : the higher the extrem propensity weights, the higher the NTV.

Estimating NTV in practice. Finally, we verify that approximating the NTV distance with a learned plug-in estimates of e(x) is reasonnable. We used either a logistic regression or a gradient boosting classifier to learn the propensity models for the three datasets where we have access to the ground truth propensity scores: Caussim, Twins and ACIC 2016. We respectively sampled 1000, 1000 and 770 instances of these datasets with different seeds and overlap settings. We first run a hyperparameter search with cross-validation on the train set, then select the best estimator. We refit on the train set this estimator with or without calibration by cross validation and finally estimate the normalized TV with the obtained model. This training procedure reflects the one described in Algorithm 1 where nuisance models are fitted only on the train set.

The hyper parameters are : learning rate $\in [1e-3, 1e-2, 1e-1, 1]$, minimum samples leaf $\in [2, 10, 50, 100, 200]$ for boosting and L2 regularization $\in [1e-3, 1e-2, 1e-1, 1]$ for logistic regression.

Results in Figure 9 comparing bias to the true normalized Total Variation of each dataset instances versus growing true NTV indicate that calibration of the propensity model is crucial to recover a good approximation of the NTV.

A.7 Experiments

Details on the data generation process

We use Gaussian-distributed covariates and random basis expansion based on Radial Basis Function kernels. A random basis of RBF kernel enables modeling non-linear and complex relationships between covariates in a similar way to the well known spline expansion. The estimators of the response function are learned with a linear model on another random basis (which can be seen as a stochastic approximation of the full data kernel [65]). We carefully control the amount of overlap between treated and control populations, a crucial assumption for causal inference. Algorithm 2 describes the generation process for one simulation. Figure 13 illustrates 2D examples of the simulation.

- The raw features for both populations are drawn from a mixture of Gaussians: $\mathbb{P}(X) = p_A \mathbb{P}(X|A=1) + (1-p_A)\mathbb{P}(X|A=0)$ where $\mathbb{P}(x|A=a)$ is a rotated Gaussian:

$$\mathbb{P}(x|A=a) = W \cdot \mathcal{N}\Big(\begin{bmatrix} (1-2a)\theta \\ 0 \end{bmatrix} ; \begin{bmatrix} \sigma_0 & 0 \\ 0 & \sigma_1 \end{bmatrix} \Big) \tag{18}$$

  with θ a parameter controlling overlap (bigger yields poorer overlap), W a random rotation matrix and $\sigma_0^2 = 2; \sigma_1^2 = 5$.
  This generation process allows to analytically compute the oracle propensity scores $e(x)$, to simply control for overlap with the parameter θ, the distance between the two Gaussian main axes and to visualize response surfaces.
- A basis expansion of the raw features increases the problem dimension. Using Radial Basis Function (RBF) Nystroem transformation [1], we expand the raw features into a transformed space. The basis expansion samples randomly a small number of representers in the raw data. Then, it computes an approximation of the full N-dimensional kernel with these basis components, yielding the transformed features $z(x)$. The number of basis functions –*ie. knots*–, controls the complexity of the ground-truth response surfaces and treatment. We first use this process to draw the non-treated response surface μ_0 and the causal effect τ. We then draw the observations from a mixture two Gaussians, for the treated and non treated. We vary the separation between the two Gaussians to control the overlap between treated and non-treated populations, an important parameter for causal inference (related to η in Assumption 3). Finally, we generate observed outcomes adding Gaussian noise.
  More formally, we generate the basis following the original data distribution, $[b_1..b_D] \sim \mathbb{P}(x)$, with D=2 in our simulations. Then, we compute an approximation of the full kernel of the data generation process with these representers: $z(x) = [RBF_\gamma(x, b_d)]_{d=1..D} \cdot Z^T \in \mathbb{R}^D$ with RBF_γ being the Gaussian kernel $K(x,y) = exp(-\gamma||x-y||^2)$ and Z the normalization constant of the kernel basis, computed as the root inverse of the basis kernel $Z = [K(b_i, b_j)]_{i,j \in 1..D}^{-1/2}$
- Functions μ_0, τ are distinct linear functions of the transformed features:

$$\mu_0(x) = \Big[z(x);1\Big] \cdot \beta_\mu^T$$

$$\tau(x) = \Big[z(x);1\Big] \cdot \beta_\tau^T$$

  Where β_μ and β_τ are sampled from two normal distributions with mean 0 and unit variances $\mathcal{N}(0, I_{D+1})$.

1 We use the Sklearn implementation, [72]

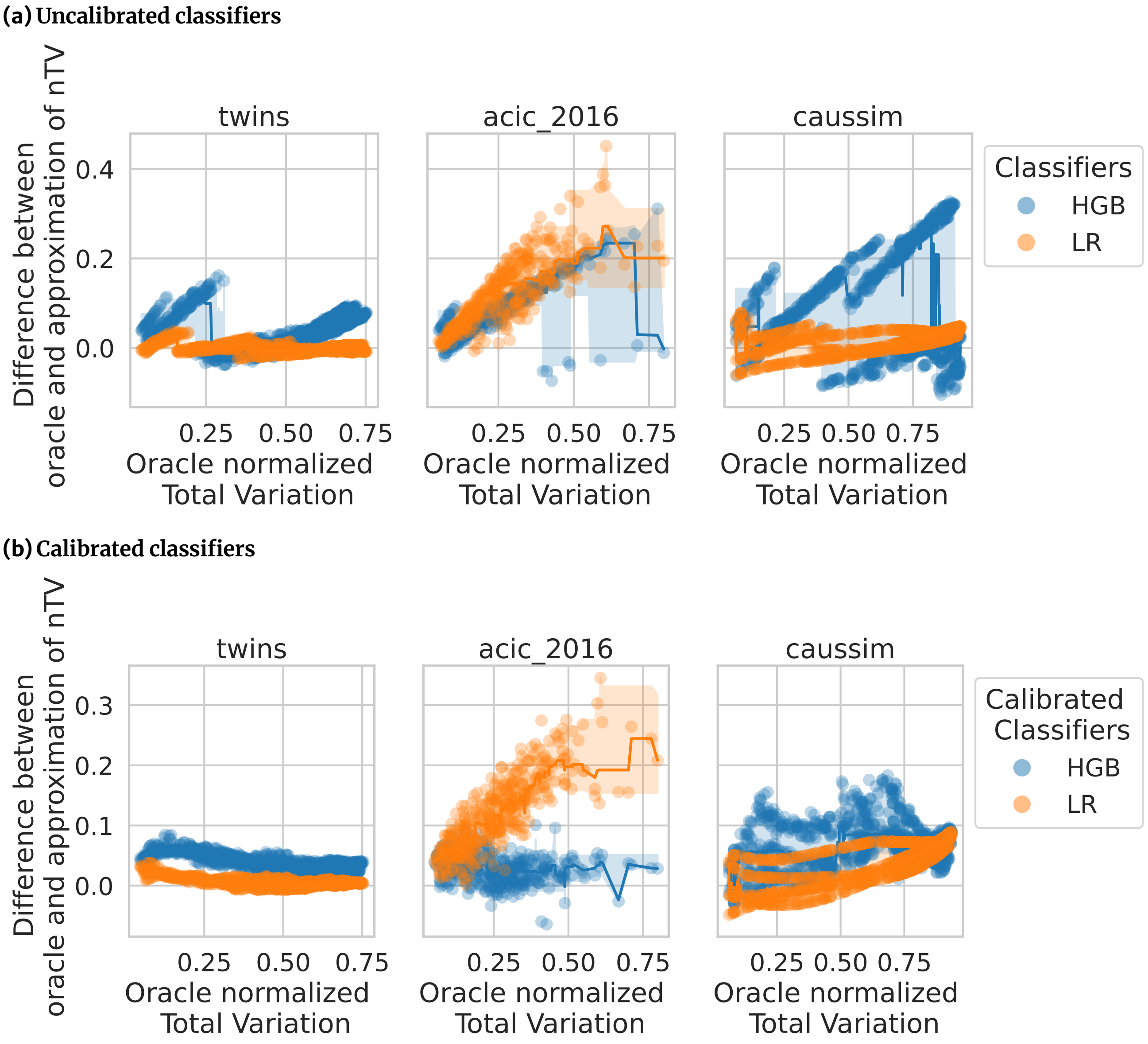

Figure 9. a) Without calibration, estimation of NTV is not trivial even for boosting models. b) Calibrated classifiers are able to recover the true Normalized Total Variation for all datasets where it is available.

Figure 10. NTV recovers well the overlap settings described in the ACIC paper [45]

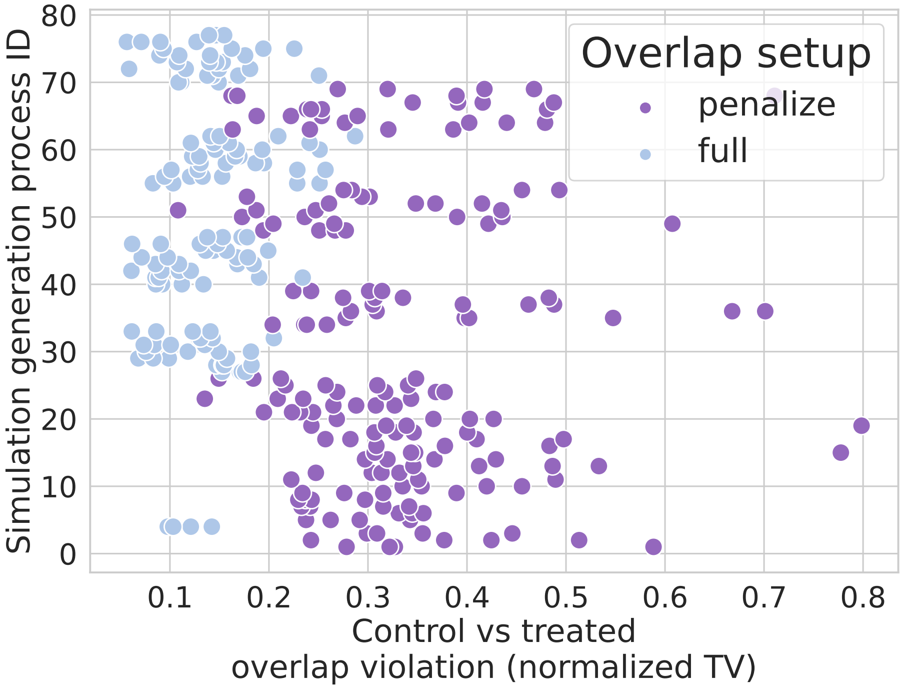

Figure 11. Good correlation between overlap measured as normalized Total Variation and Maximum Mean Discrepancy (200 sampled Caussim datasets)

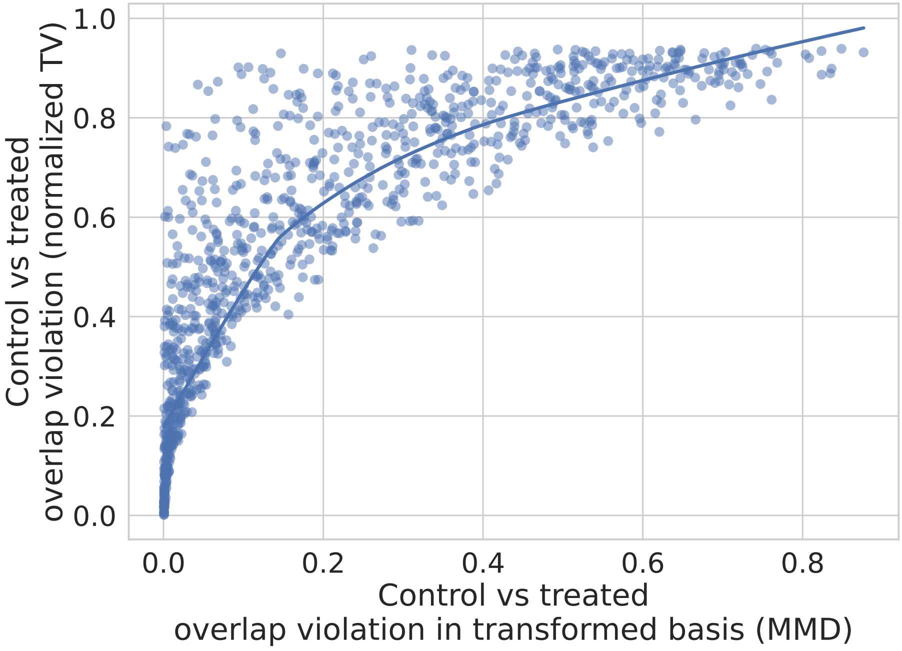

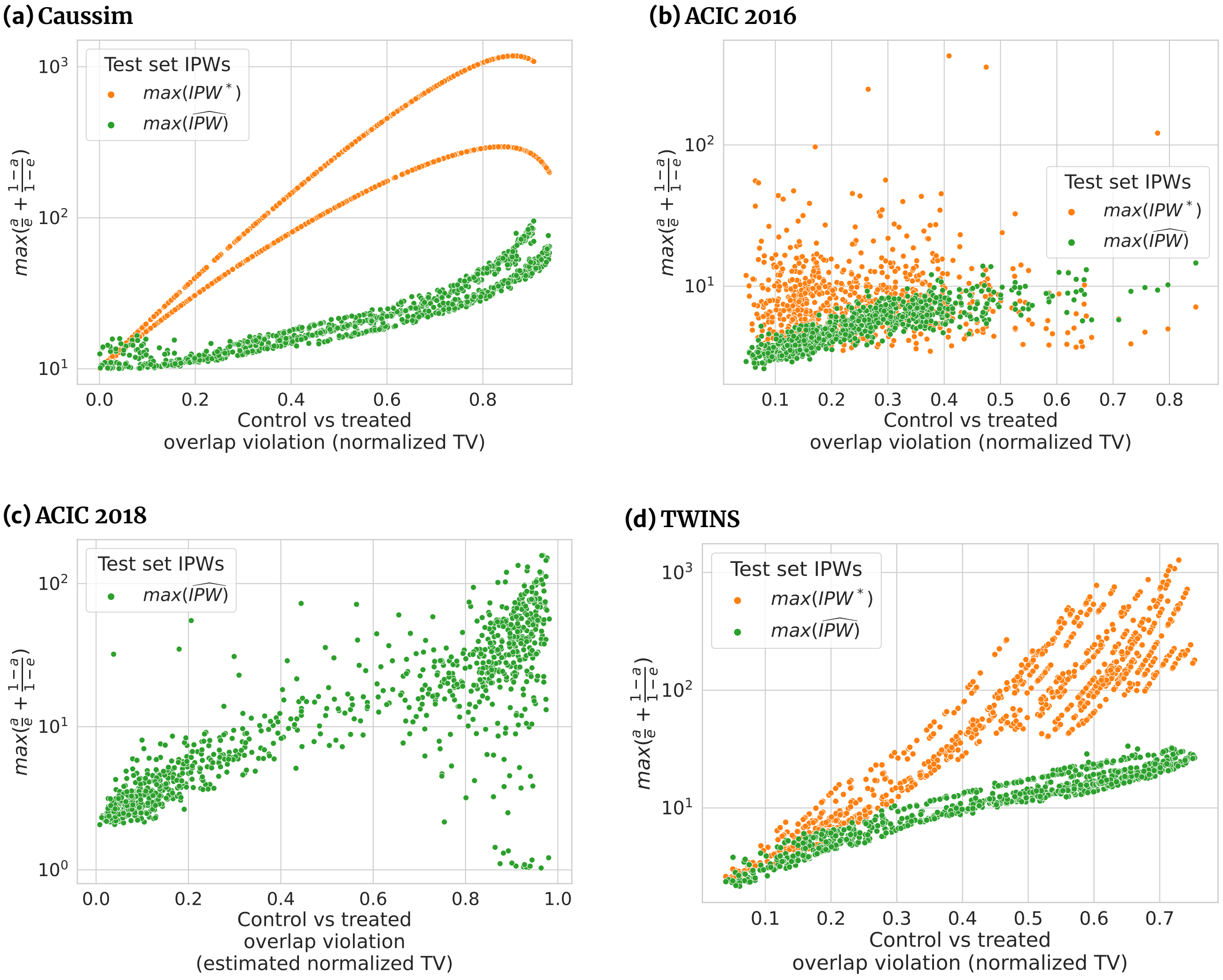

Figure 12. Maximal value of Inverse Propensity Weights increases exponentially with the overlap as measure by Normalized Total Variation.

- Adding a Gaussian noise, $\varepsilon \sim \mathcal{N}(0, \sigma(x; a))$, we construct the potential outcomes: $y(a) = (1 - \omega)\mu_0(x) + a\,\omega\tau(x) + \varepsilon(x, a)$ with ω the treatment effect relative size with respect to the baseline response.

We generated 1000 instances of this dataset with uniformly random overlap parameters $\theta \in [0, 2.5]$.

Details on the semi-simulated datasets

ACIC 2018 [45]: The initial intervention was a child's birth weight ($A = 1$ if weight $< 2.5kg$), and outcome was the child's IQ after a follow-up period. The study contained $N = 4\,802$ data points with $D = 55$ features (5 binary, 27 count data, and 23 continuous). They simulated 77 different setups varying parameters for treatment and response models, overlap, and interactions between treatment and covariates [2]. We used 10 different seeds for each setup, totaling 770 dataset instances.

ACIC 2018 [68]: Starting from data from the Linked Births and Infant Deaths Database (LBIDD) [69] with $D = 177$ covariates, treatment and outcome models are simulated with complex models to reflect different scenarii. The data do not provide the true propensity scores, so we evaluate only feasible metrics, which do not require this nuisance parameter. We used all 432 datasets[3] of size $N = 5\,000$.

Twins [70]: It is an augmentation of real data on twin births and mortality rates [71]. There are $N = 11\,984$ samples (pairs of twins), and $D = 50$ covariates[4], The outcome is the mortality and the treatment is the weight of the heavier twin at birth. This is a "true" counterfactual dataset [94] in the sense that we have both potential outcomes with each twin. They simulate the treatment with a sigmoid model based on GESTAT10 (number of gestation weeks before birth) and x the 45 other covariates:

$$t_i \mid \mathbf{x}_i, \mathbf{z}_i \sim \text{Bern}\left(\sigma\left(w_o^\top \mathbf{x} + w_h(\mathbf{z}/10 - 0.1)\right)\right) \quad (19)$$

$$\text{with } w_o \sim \mathcal{N}(0, 0.1 \cdot I),\; w_h \sim \mathcal{N}(5, 0.1)$$

2 Original R code available at https://github.com/vdorie/aciccomp/tree/master/2016 to generate 77 simulations settings.
3 Using the scaling part of the data, from github.com/IBM-HRL-MLHLS/IBM-Causal-Inference-Benchmarking-Framework
4 We obtained the dataset from https://github.com/AMLab-Amsterdam/CEVAE/tree/master/datasets/TWINS

Algorithm 2 Data simulation for the simulated dataset caussim

Let the parameters of a given simulation be:

- N the number of samples.
- D the dimension of the basis expansion.
- p_A the proportion of treated individuals.
- θ the overlap parameter.
- $\mathbb{P}(x|A=a) = W \cdot \mathcal{N}\left(\begin{bmatrix}(1-2a)\theta \\ 0\end{bmatrix}; \begin{bmatrix}\sigma_0 & 0 \\ 0 & \sigma_1\end{bmatrix}\right)$ the rotated gaussian for each population covariate. W is a random rotation matrix and $\sigma_0^2 = 2; \sigma_1^2 = 5$ are scaling parameters.
- $e(x) = \mathbb{P}(A=1|X=x)$ the oracle propensity score, obtained analytically from $\mathbb{P}(x|A=a)$.
- $\mathbb{P}(X) = p_A\mathbb{P}(X|A=1) + (1-p_A)\mathbb{P}(X|A=0)$ the mixture of gaussian for the whole population covariates.
- $b_1, .., b_D \sim \mathbb{P}(X)$, the basis sampled from the gaussian mixture.
- $z(x) = [RBF_\gamma(x, b_d)]_{d=1..D} \cdot Z^T \in \mathbb{R}^D$ the Nystroem expansion. RBF_γ is the Gaussian kernel $K(x,y) = exp(-\gamma||x-y||^2)$ and Z is the normalization constant of the kernel basis, computed as the root inverse of the basis kernel $Z = [K(b_i, b_j)]^{-1/2}_{i,j\in 1..D}$.
- β_μ and β_τ, the linear coefficients on top of the basis expansion. They are sampled from two normal distributions with mean 0 and unit variances $\mathcal{N}(0, I_{D+1})$.
- ω the treatment effect relative size with respect to the baseline response.
- $\varepsilon(x,a) \sim \mathcal{N}(0, \sigma_y)$ an exogen gaussian noise with σ_y the scale of the noise.

Generation process for one sample $(x, a, e(x), y(0), y(1), y)$:

1: $x \sim \mathbb{P}(X)$ ▷ Sample from rotated gaussian mixture
2: $z(x)$ ▷ Compute the Nystroem expansion
3: $\mu_0(x) = \left[z(x); 1\right] \cdot \beta_\mu^T$ ▷ Compute the non-treated response surface, ie. the baseline
4: $\tau(x) = \left[z(x); 1\right] \cdot \beta_\tau^T$ ▷ Compute the conditional treatment effect (CATE)
5: $y(a) = (1-\omega)\mu_0(x) + a\,\omega\tau(x) + \varepsilon(x,a) + \varepsilon(x,a)$ ▷ Compute the potential outcomes
6: $a \sim \text{Bernoulli}(e(x))$ ▷ Compute the treatment status
7: $y = a\,y(1) + (1-a)\,y(0)$ ▷ Compute the observed outcome

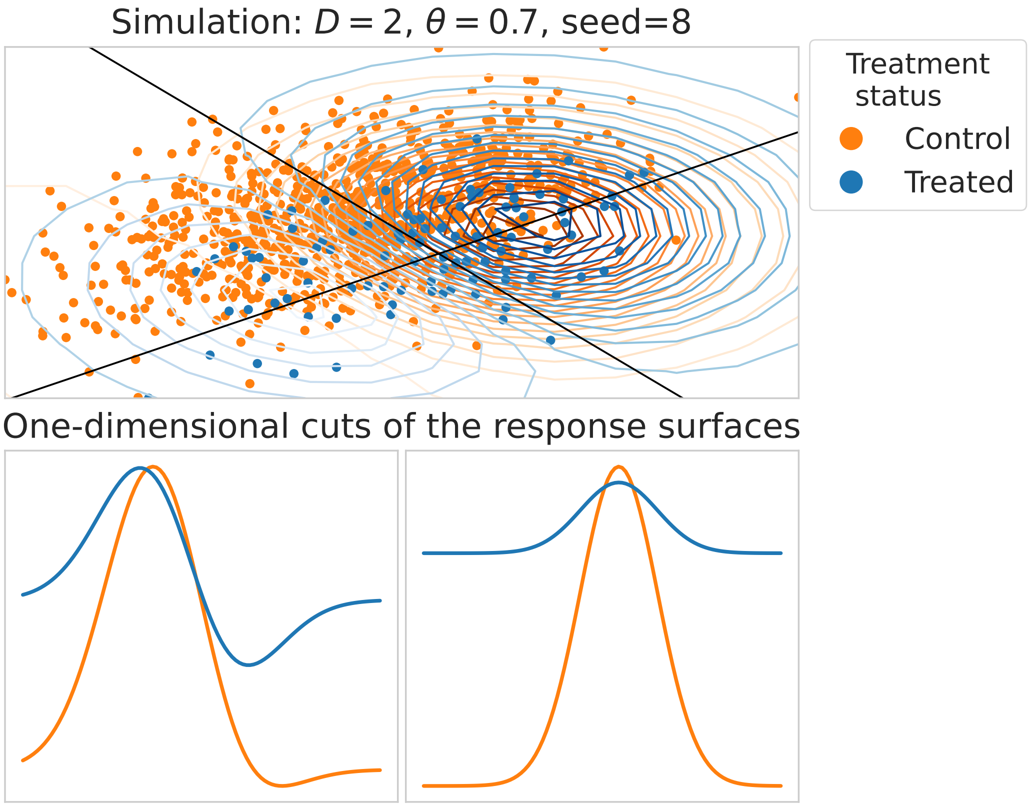

Figure 13. Example of the simulation setup in the input space with two knots –*ie.* basis functions. The top panel shows the observations in feature space, while the bottom panel displays the two response surfaces on a 1D cut along the black lines drawn on the top panel.

We add a non-constant slope in the sigmoid to control the overlap between treated and control populations. We sampled uniformly 1 000 different overlap parameters between 0 and 2.5, totaling 1 000 dataset instances. Unlike the previous datasets, only the overlap varies for these instances. The response surfaces are set by the original outcomes.

Model selection procedures

Nuisances estimation. The nuisances are estimated with a stacked regressor inspired by the Super Learner framework, [95]). We select hyper-parameters with randomized search on a validation set $\mathcal{V}$ and keep them fix for model selection The searc grid is detailed in Table 3. All implementations come from scikit-learn [72]. As extreme inverse propensity weights induce high variance, we use clipping [96, 97] to bound $min(\check{e}, 1 - \check{e})$ away from 0 with a fixed $\eta = 10^{-10}$, ensuring strict overlap for numerical stability.

Model	Estimator	Hyper-parameters grid
Outcome, m	StackedRegressor (HistGradientBoostingRegressor, ridge)	ridge regularization: [0.0001, 0.001, 0.01, 0.1, 1, 10, 100]<br>HistGradientBoostingRegressor learning rate: [0.01, 0.1, 1]<br>HistGradientBoostingRegressor max leaf nodes: [10, 20, 30, 50]
Treatment, e	StackedClassifier (HistgradientBoostingClassifier, LogisticRegression)	LogisticRegression C: [0.0001, 0.001, 0.01, 0.1, 1, 10, 100]<br>HistGradientBoostingClassifier learning rate: [0.01, 0.1, 1]<br>HistGradientBoostingClassifier max leaf nodes: [10, 20, 30, 50]

Table 3. Hyper-parameters grid used for nuisance models

Additional Results

Definition of the Kendall's tau, κ. The Kendall's tau is a widely used statistics to measure the rank correlation between two set of observations. It measures the number of concordant pairs minus the discordant pairs normalized by the total number of pairs. It takes values in the $[-1, 1]$ range.

$$\kappa = \frac{\text{(number of concordant pairs)} - \text{(number of discordant pairs)}}{\text{(number of pairs)}} \tag{20}$$

Values of relative $\kappa(\ell, \tau\text{-risk})$ compared to the mean over all metrics Kendall's as shown in the boxplots of Figure 3.

Figure 14 - Results measured in relative Kendall's for feasible and semi-oracle risks. Because of extreme propensity scores in the denominator and bayes error residuals in the numerator, the semi-oracle U-risk has poor performances at bad overlap. Estimating these propensity scores in the is feasible U-risk reduces the variance since clipping is performed.

Figure 15 - Results measured in absolute Kendall's.

Figure 16 - Results measured as distance to the oracle tau-risk. To see practical gain in term of τ-risk, we plot the results as the normalized distance between the estimator selected by the oracle τ-risk and the estimator selected by each causal metric.

Then, $\widehat{R\text{-risk}}^*$ is more efficient than all other metrics. The gain are substantial for every datasets.

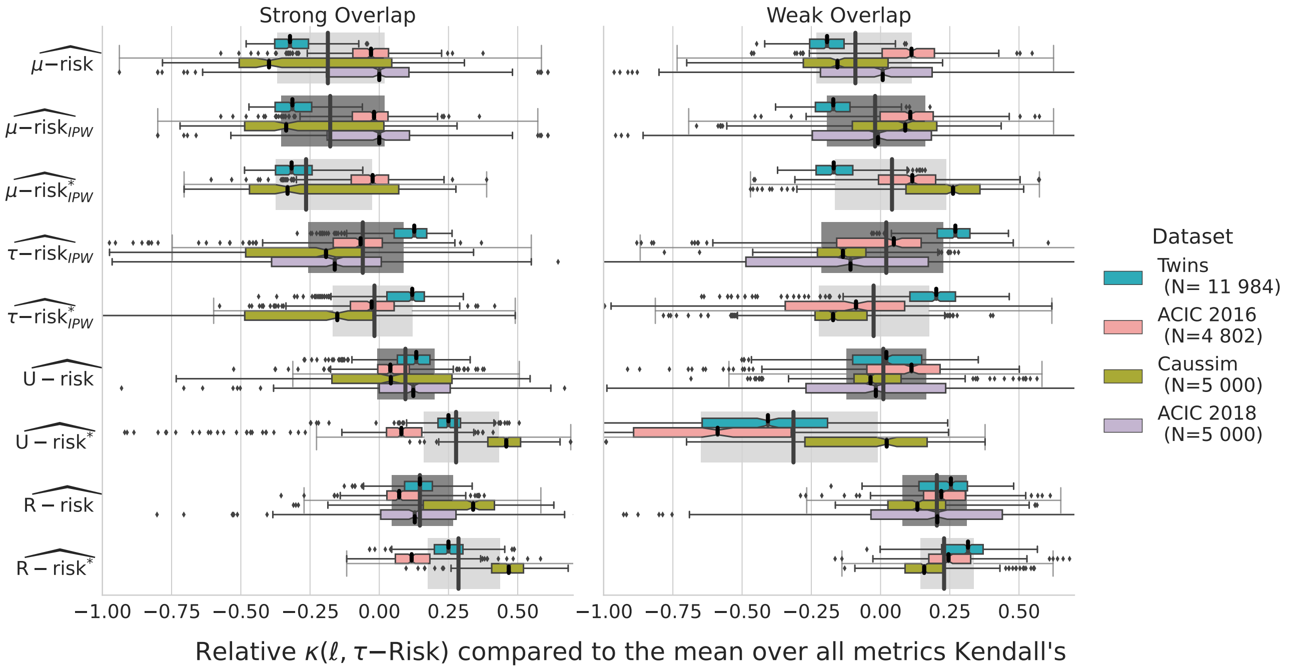

Figure 14. The R-risk is the best metric on average: Relative Kendall's τ agreement with τ-risk. Strong and Weak overlap correspond to the first and last tertiles of the overlap distribution measured with Normalized Total Variation eq. 17.

Metric	Dataset	Strong Overlap Median	Strong Overlap IQR	Weak Overlap Median	Weak Overlap IQR
$\widehat{\mu\text{-risk}}$	Twins (N=11 984)	-0.32	0.12	-0.19	0.12
	ACIC 2016 (N=4 802)	-0.03	0.13	0.11	0.19
	Caussim (N=5 000)	-0.40	0.55	-0.16	0.31
	ACIC 2018 (N=5 000)	0.00	0.30	0.01	0.40
$\widehat{\mu\text{-risk}}_{IPW}$	Twins (N= 11 984)	-0.31	0.13	-0.17	0.12
	ACIC 2016 (N=4 802)	-0.02	0.13	0.11	0.19
	Caussim (N=5 000)	-0.34	0.50	0.09	0.31
	ACIC 2018 (N=5 000)	0.00	0.30	-0.01	0.43
$\widehat{\mu\text{-risk}}^{*}_{IPW}$	Twins (N= 11 984)	-0.32	0.13	-0.17	0.13
	ACIC 2016 (N=4 802)	-0.02	0.13	0.11	0.21
	Caussim (N=5 000)	-0.33	0.54	0.26	0.27
$\widehat{\tau\text{-risk}}_{IPW}$	Twins (N= 11 984)	0.13	0.12	0.27	0.12
	ACIC 2016 (N=4 802)	-0.07	0.18	0.05	0.31
	Caussim (N=5 000)	-0.19	0.43	-0.14	0.18
	ACIC 2018 (N=5 000)	-0.16	0.40	-0.11	0.66
$\widehat{\tau\text{-risk}}^{*}_{IPW}$	Twins (N= 11 984)	0.12	0.14	0.20	0.16
	ACIC 2016 (N=4 802)	-0.03	0.16	-0.09	0.43
	Caussim (N=5 000)	-0.15	0.46	-0.17	0.19
$\widehat{U - \text{risk}}$	Twins (N= 11 984)	0.13	0.12	0.02	0.25
	ACIC 2016 (N=4 802)	0.04	0.11	0.11	0.26
	Caussim (N=5 000)	0.04	0.43	-0.04	0.17
	ACIC 2018 (N=5 000)	0.12	0.26	-0.02	0.50
$\widehat{U - \text{risk}}^{*}$	Twins (N= 11 984)	0.25	0.08	-0.41	0.45
	ACIC 2016 (N=4 802)	0.08	0.13	-0.59	0.57
	Caussim (N=5 000)	0.46	0.12	0.02	0.44
$\widehat{R - \text{risk}}$	Twins (N= 11 984)	0.15	0.10	0.25	0.18
	ACIC 2016 (N=4 802)	0.07	0.12	0.22	0.15
	Caussim (N=5 000)	0.34	0.26	0.13	0.21
	ACIC 2018 (N=5 000)	0.13	0.27	0.21	0.47
$\widehat{R - \text{risk}}^{*}$	Twins (N= 11 984)	0.25	0.10	0.32	0.15
	ACIC 2016 (N=4 802)	0.12	0.12	0.25	0.15
	Caussim (N=5 000)	0.47	0.11	0.16	0.14

Table 4. Values of relative $\kappa(\ell, \tau\text{-risk})$ compared to the mean over all metrics Kendall's as shown in the boxplots of Figure 3

(a) Caussim

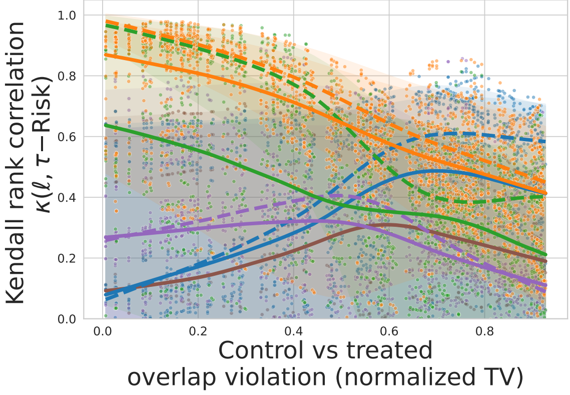

(b) ACIC 2016

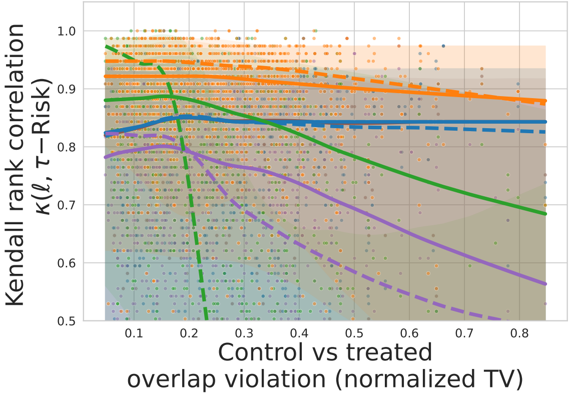

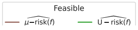

(c) ACIC 2018

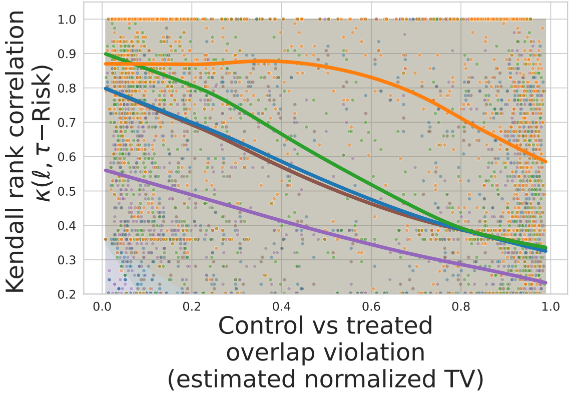

(d) TWINS

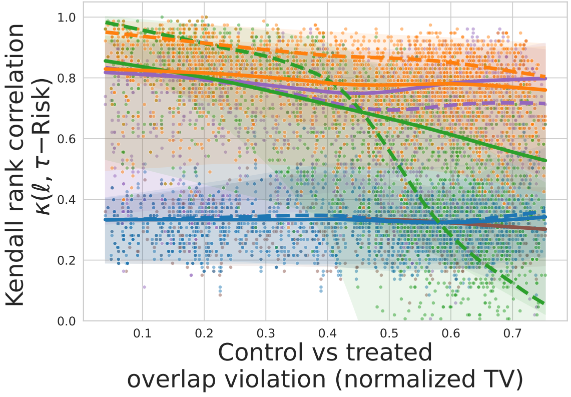

Figure 15. Agreement with τ-risk ranking of methods function of overlap violation. The lines represent medians, estimated with a lowess. The transparent bands denote the 5% and 95% confidence intervals.

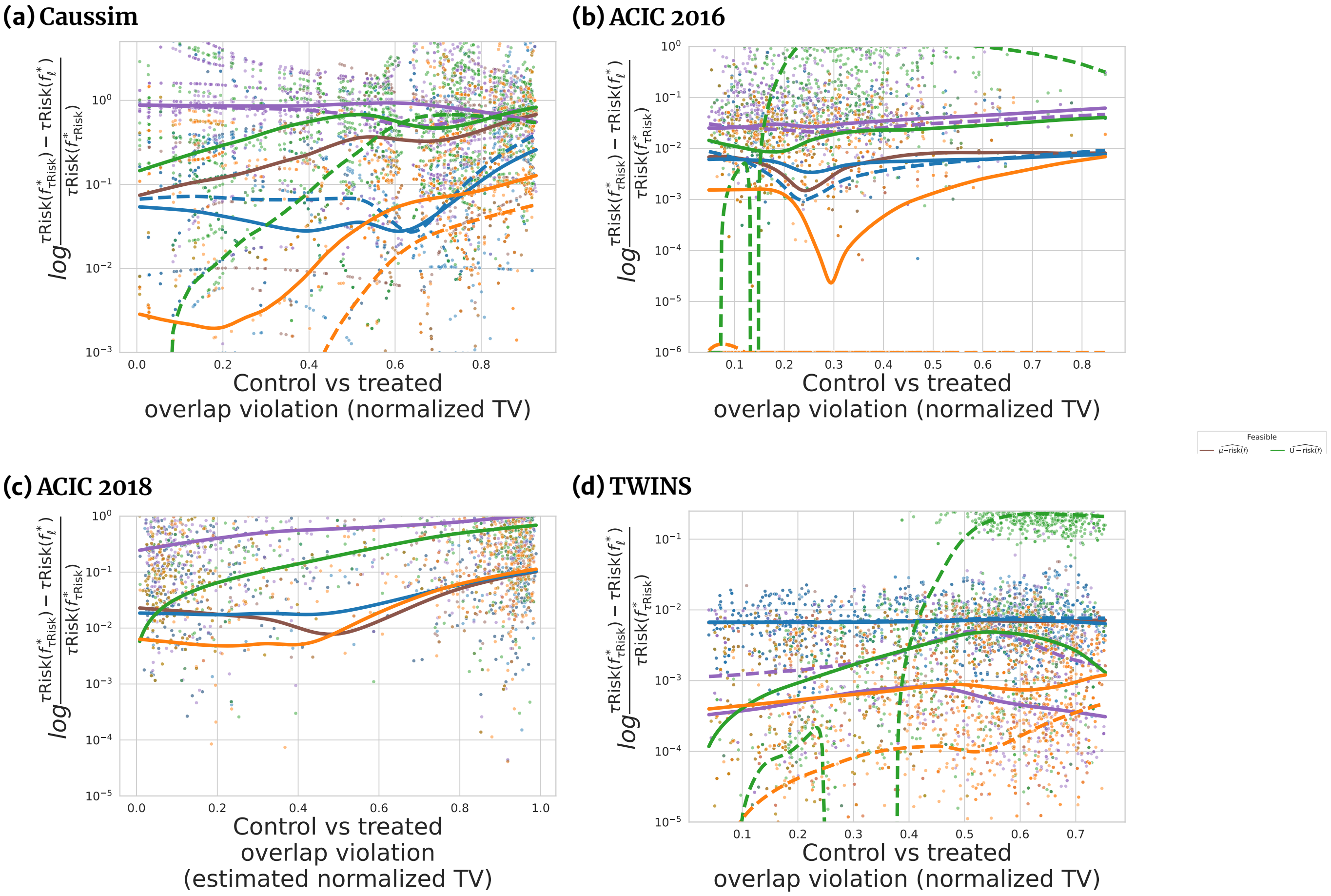

Figure 16. Metric performances by normalized tau-risk distance to the best method selected with τ-risk. All nuisances are learned with the same estimator stacking gradient boosting and ridge regression. Doted and plain lines corresponds to 60% lowess quantile estimates. This choice of quantile allows to see better the oracle metrics lines for which outliers with a value of 0 distord the curves.

Figure 17 - Stacked models for the nuisances is more efficient. For each metrics the benefit of using a stacked model of linear and boosting estimators for nuisances compared to a linear model. The evaluation measure is Kendall's tau relative to the oracle R-risk* to have a stable reference between exepriments. Thus, we do not include in this analysis the ACIC 2018 dataset since R-risk* is not available due to the lack of the true propensity score.

Figure 18 Low population overlap hinders model selection for all metrics.

Figure 19 - Stacked models for the nuisances is more efficient. For each metrics the benefit of using a stacked model of linear and boosting estimators for nuisances compared to a linear model. The evaluation measure is Kendall's tau relative to the oracle R-risk* to have a stable reference between exepriments. Thus, we do not include in this analysis the ACIC 2018 dataset since R-risk* is not available due to the lack of the true propensity score.

Figure 20 - Flexible models are performant in recovering nuisances even in linear setups.

Selecting different seeds and parameters is crucial to draw conclusions. One strength of our study is the various number of different simulated and semi-simulated datasets. We are convinced that the usual practice of using only a small number of generation processes does not allow to draw statistically significant conclusions.

Figure 21 illustrate the dependence of the results on the generation process for caussim simulations. We highlighted the different trajectories induced by three different seeds for data generation and three different treatment ratio instead of 1000 different seeds. The result curves are relatively stable from one setup to another for $R-risk$, but vary strongly for μ-risk and μ-risk$_{IPW}$.

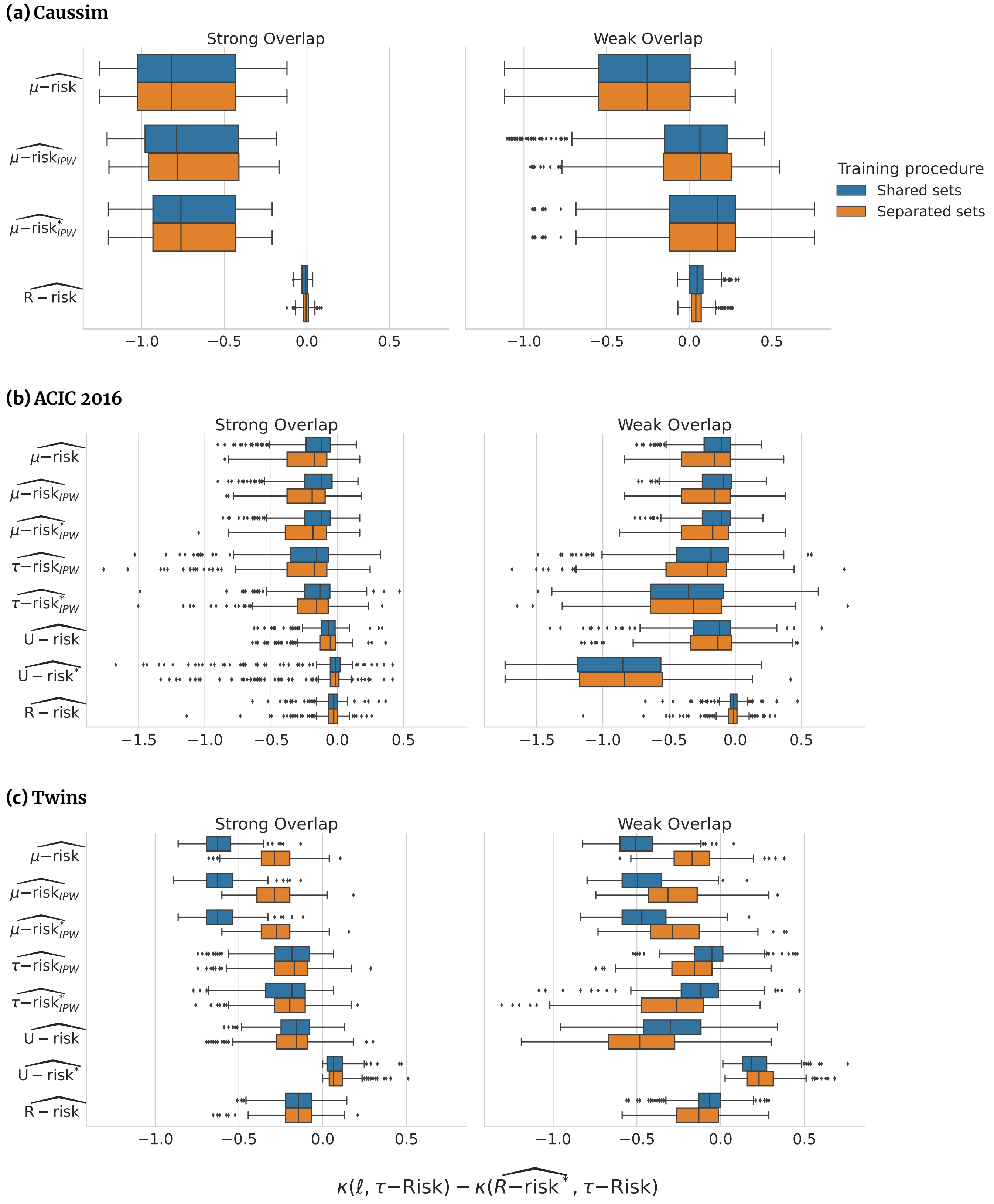

Figure 17. Results are similar between the Shared nuisances/candidate set and the Separated nuisances set procedure. The experience has not been run on the full metrics for Caussim due to computation costs.

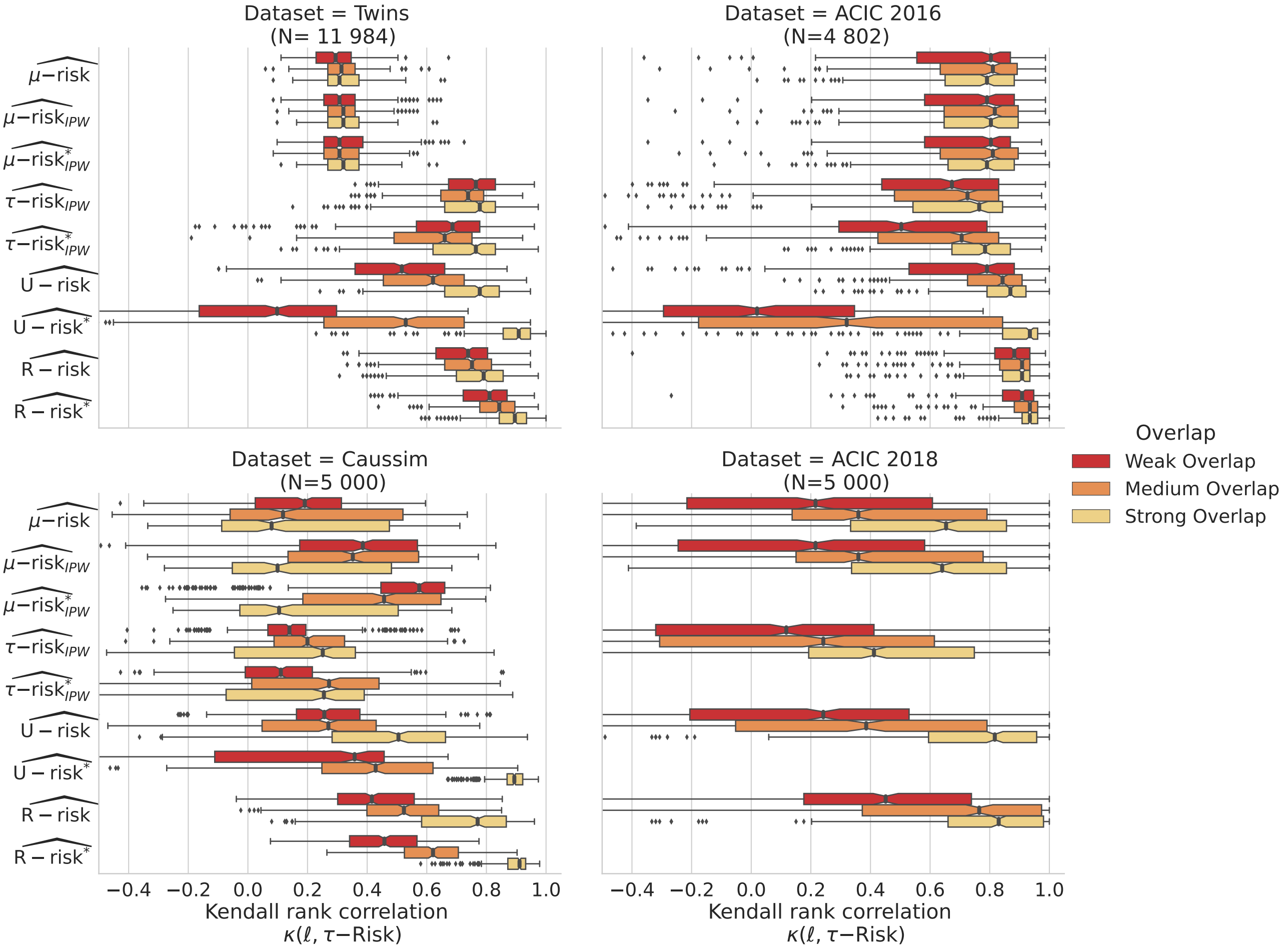

Figure 18. Low population overlap hinders causal model selection for all metrics: Kendall's τ agreement with τ-risk. Strong, medium and Weak overlap correspond to the tertiles of the overlap distribution measured with Normalized Total Varation eq. 17.

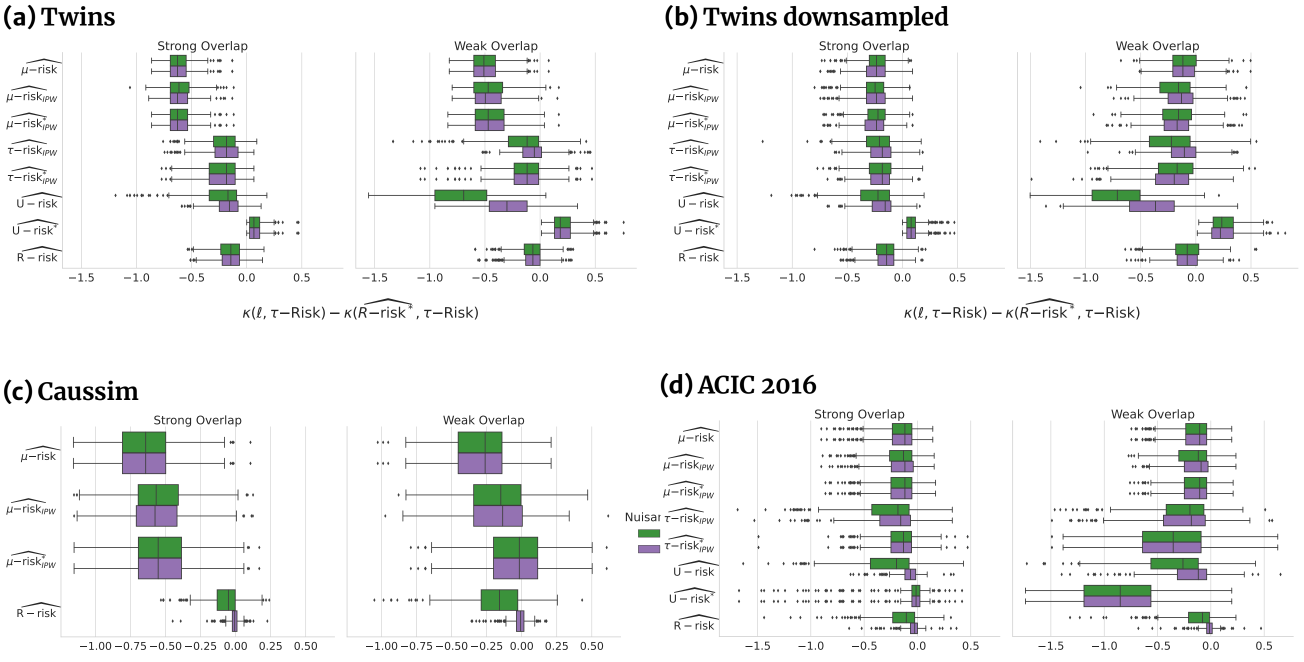

Figure 19. Learning the nuisances with stacked models (linear and gradient boosting) is important for successful model selection with R-risk. For Twins dataset, there is no improvement for stacked models compared to linear models because of the linearity of the propensity model.

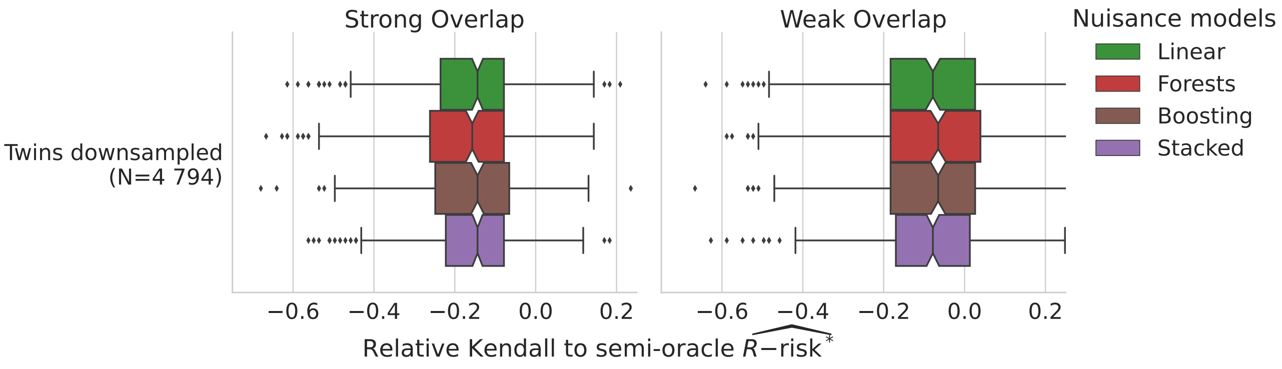

Figure 20. Flexible models are performant in recovering nuisances in the downsampled Twins dataset. The propensity score is linear in this setup, making it particularly challenging for flexible models compared to linear methods.

Figure 21. Kendall correlation coefficients for each causal metric. Each (color, shape) pair indicates a different (treatment ratio, seed) of the generation process.

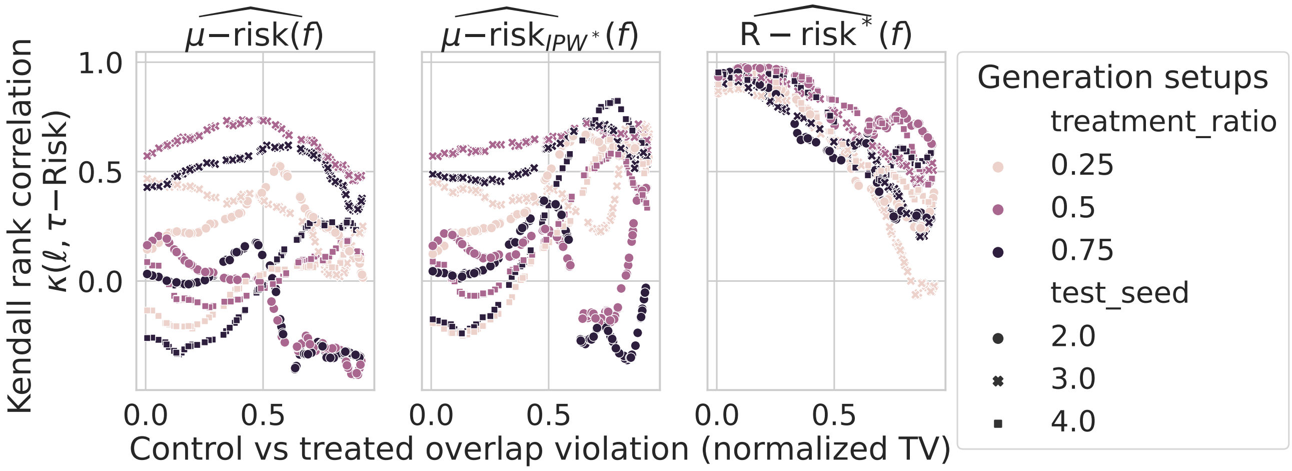

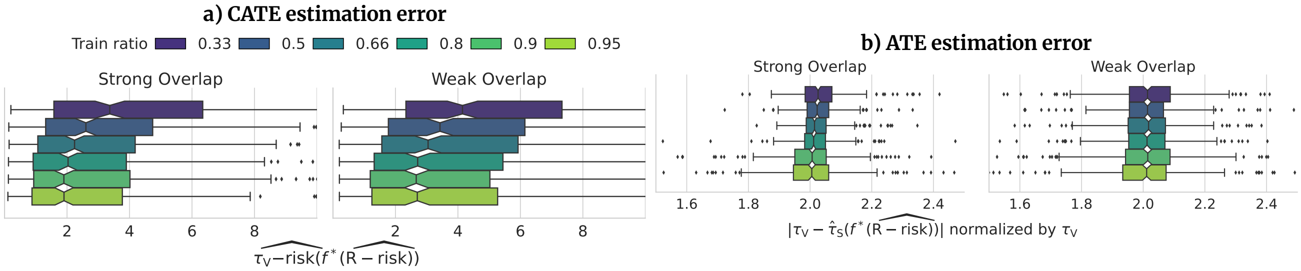

Figure 22. a) For CATE, a train/test ratio of 0.9/0.1 appears a good trade-off. b) For ATE, there is a small signal pointing also to 0.9/0.1 (K=10). for ATE. Experiences on 10 replications of all 78 instances of the ACIC 2016 data.

A.8 Data split choices

Use 90% of the data to estimate outcome models, 10% to select them

The analyst faces a compromise: given a finite data sample, should she allocate more data to estimate the outcome model, thus improving the quality of the outcome model but leaving little data for model selection. Or, she could choose a bigger test set for model selection and effect estimation. For causal model selection, there is no established practice (as reviewed in A.8).

We investigate such tradeoff varying the ratio between train and test data size. For this, we first split out 30% of the data as a holdout set $\mathcal{V}$ on which we use the oracle response functions to derive silver-standard estimates of causal quantities. We then use the standard estimation procedure on the remaining 70% of the data, splitting it into train $\mathcal{T}$ and test $\mathcal{S}$ of varying sizes. We finally measure the error between this estimate and the silver standard.

We consider two different analytic goals: estimating a average treatment effect –a single number used for policy making– and a CATE –a full model of the treatment effect as a function of covariates X. Given that the latter is a much more complex object than the former, the optimal train/test ratio might vary. To measure errors, we use for the ATE the relative absolute ATE bias between the ATE computed with the selected outcome model on the test set, and the true ATE as evaluated on the holdout set $\mathcal{V}$. For the CATE, we compare the τ-risk of the best selected model applied on the holdout set $\mathcal{V}$. We explore this trade-off for the ACIC 2016 dataset and the R-risk.

Figure 22 shows that a train/test ratio of 0.9/0.1 (K=10) or 0.8/0.2 (K=5) appears best to estimate CATE and ATE.

Heterogeneity in practices for data split

Splitting the data is common when using machine learning for causal inference, but practices vary widely in terms of the fraction of data to allocate to train models, outcomes and nuisances, and to evaluate them.

Before even model selection, data splitting is often required for estimation of the treatment effect, ATE or CATE, for instance to compute the nuisances required to optimize the outcome model (as the R-risk, Definition 5). The most frequent choice is use 80% of the data to fit the models, and 20% to evaluate them. For instance, for CATE estimation, the R-learner has been introduced using K-folds with K = 5 and K = 10: 80% of the data (4 folds) to train the nuisances and the remaining fold to minimize the corresponding R-loss [42]. Yet, it has been implemented with K=5 in causallib [98] or K=3 in econML [99]. Likewise, for ATE estimation, [43] introduce doubly-robust machine learning, recommending K=5 based on an empirical comparison K=2. However, subsequent works use doubly robust ML with varying choices of K: [100] use K=3, [101] use K=2. In the econML implementation, K is set to 3 [99]. [102] evaluate various machine-learning approaches –including R-learners– using K=5 and 10, drawing inspiration from the TMLE literature which sets K=5 in the TMLE package [103].

Causal model selection has been much less discussed. The only study that we are aware of, [50], use a different data split: a 2-folds train/test procedure, training the nuisances on the first half of the data, and using the second half to estimate the R-risk and select the best treatment effect model.

A.9 Sensitivity of the results to different ratio of causal effect to baseline response

Going beyond overlap, we study the effect of another important parameter of the data generation process: the ratio of causal effect to the baseline response.

Here, we detail the experimental setup of the results presented in Figure 7.

For each dataset of our simulation (caussim), we measure this ratio as the absolute mean difference between the causal effect and the baseline response, measured empirically on each dataset instance $\Delta_\mu = \frac{1}{N}\sum_{i=1}^{N}\left|\frac{\mu_1(x_i)-\mu_0(x_i)}{\mu_0(x_i)+\mu_1(x_i)-\frac{1}{N}\sum_{j=1}^{N}\mu_0(x_j)+\mu_1(x_j)}\right|$. We vary a dedicated parameter (ω in Algorithm 2) in the simulation to force this ratio to cover a wide range of values. For 179 dataset instances, it ranges several order of magnitudes with the full distribution of values given in Table 5.

	count	mean	std	min	1%	10%	25%	50%	75%	90%	99%	max
effect_ratio	179	16.95	24.92	0.043	0.054	0.32	1.34	9.12	22.55	39.97	101.12	206.53

Table 5. Distribution of the causal effect ratio Δ_μ for the experiment on causal effect ratio, results in Figure 7.

We also studied another measure, focusing on the ratio of the variance of the causal effect to the variance of the mean shared response ($\mu_0 + \mu_1$) of both population: $\frac{\hat{var}(\mu_1(x_i)-\mu_0(x_i))}{\hat{var}(\mu_1(x_i)+\mu_0(x_i))}$, covering a range from 0 to 11.08 with the full distribution described in Table 6. Figure 23 shows that for high values of the variance, $R-$risk is outperformed by the μ-risk$_{IPW}$ and the τ-risk$_{IPW}$. However, on average, the $R-$risk

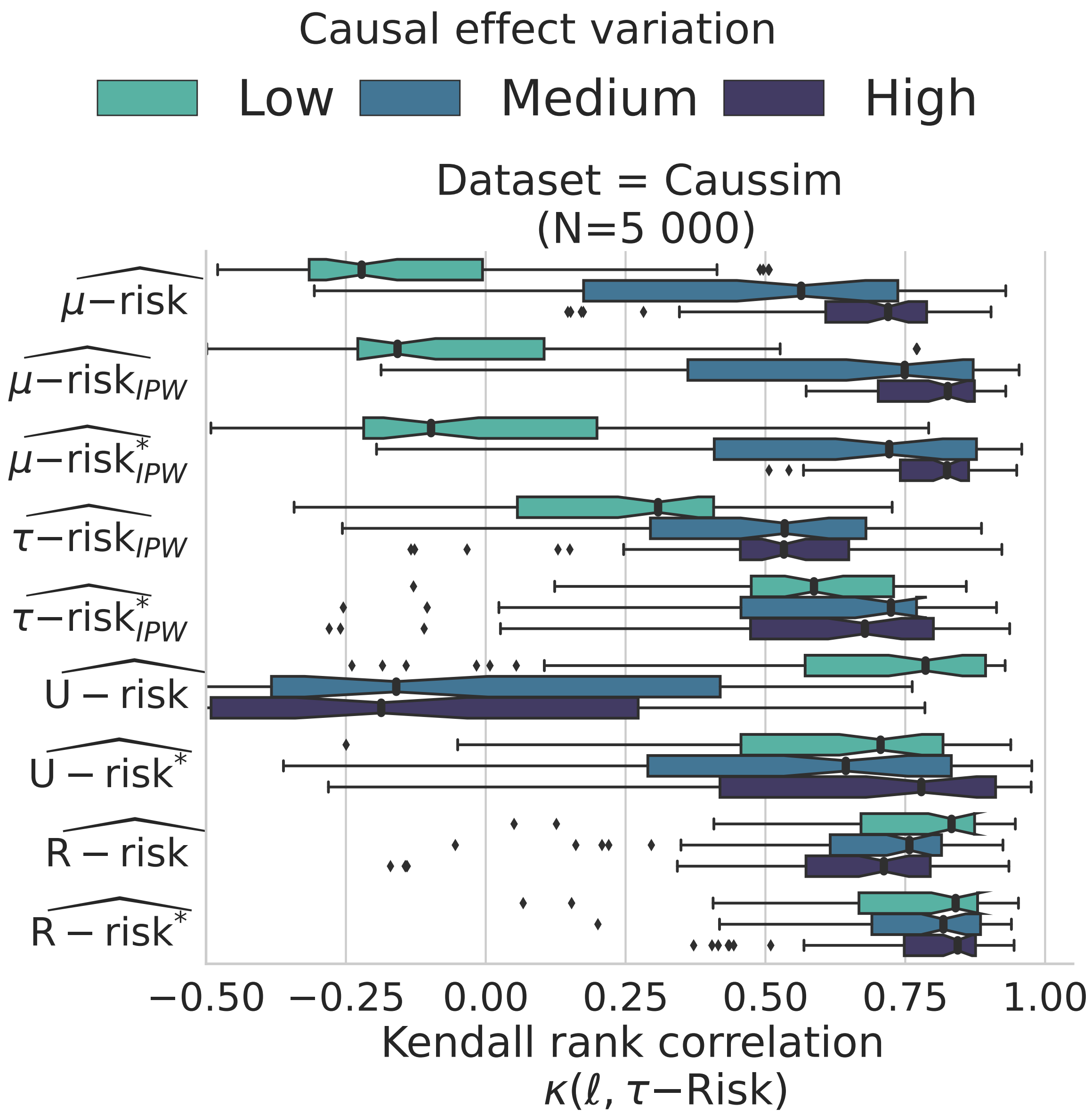

Figure 23. R-risk is robust to a wide range of effect variance: Kendall's τ agreement with τ-risk. Strong, medium and Weak Causal effect variance are the tertiles of the ratio of causal effect variance to the mean outcome variance: Low [0; 0.04[, Medium [0.04; 1.04[, High [1.04; 11.08[.

is still the better risk.

	count	mean	std	min	1%	10%	25%	50%	75%	90%	99%	max
**effect_variance**	179	1.013	1.781	1.680e-5	1.734e-5	4.875e-4	6.449e-3	0.789	1.160	1.807	9.641	11.079

Table 6. Statistical summary of the causal effect variance.

References

1. Karel GM Moons, Patrick Royston, Yvonne Vergouwe, Diederick E Grobbee, and Douglas G Altman. Prognosis and prognostic research: what, why, and how? *Bmj*, 338, 2009.
2. Ewout W Steyerberg. *Clinical prediction models*. Springer.
3. Andrew L Beam and Isaac S Kohane. Big data and machine learning in health care. *Jama*, 319(13):1317–1318, 2018.
4. Alvin Rajkomar, Jeffrey Dean, and Isaac Kohane. Machine learning in medicine. *New England Journal of Medicine*, 380(14):1347–1358, 2019.
5. M Khojaste-Sarakhsi, Seyedhamidreza Shahabi Haghighi, SMT Fatemi Ghomi, and Elena Marchiori. Deep learning for alzheimer's disease diagnosis: A survey. *Artificial Intelligence in Medicine*, page 102332, 2022.
6. Zhenwei Zhang and Ervin Sejdić. Radiological images and machine learning: trends, perspectives, and prospects. *Computers in biology and medicine*, 108:354–370, 2019.
7. Adam Yala, Constance Lehman, Tal Schuster, Tally Portnoi, and Regina Barzilay. A deep learning mammography-based model for improved breast cancer risk prediction. *Radiology*, 292(1):60–66, 2019.
8. Li Shen, Laurie R Margolies, Joseph H Rothstein, Eugene Fluder, Russell McBride, and Weiva Sieh. Deep learning to improve breast cancer detection on screening mammography. *Scientific reports*, 9(1):12495, 2019.
9. Ali Bou Nassif, Manar Abu Talib, Qassim Nasir, Yaman Afadar, and Omar Elgendy. Breast cancer detection using artificial intelligence techniques: A systematic literature review. *Artificial Intelligence in Medicine*, page 102276, 2022.
10. Stephen J Mooney and Vikas Pejaver. Big data in public health: terminology, machine learning, and privacy. *Annual review of public health*, 39:95, 2018.
11. Rishi J Desai, Shirley V Wang, Muthiah Vaduganathan, Thomas Evers, and Sebastian Schneeweiss. Comparison of machine learning methods with traditional models for use of administrative claims with electronic medical records to predict heart failure outcomes. *JAMA network open*, 3(1):e1918962–e1918962, 2020.
12. Gregory E Simon, Eric Johnson, Jean M Lawrence, Rebecca C Rossom, Brian Ahmedani, Frances L Lynch, Arne Beck, Beth Waitzfelder, Rebecca Ziebell, Robert B Penfold, et al. Predicting suicide attempts and suicide deaths following outpatient visits using electronic health records. *American Journal of Psychiatry*, 175(10):951–960, 2018.
13. Steven Horng, David A Sontag, Yoni Halpern, Yacine Jernite, Nathan I Shapiro, and Larry A Nathanson. Creating an automated trigger for sepsis clinical decision support at emergency department triage using machine learning. *PloS one*, 12(4):e0174708, 2017.
14. Hanyin Wang, Yikuan Li, Seema A Khan, and Yuan Luo. Prediction of breast cancer distant recurrence using natural language processing and knowledge-guided convolutional neural network. *Artificial intelligence in medicine*, 110:101977, 2020.
15. Irena Spasic, Goran Nenadic, et al. Clinical text data in machine learning: systematic review. *JMIR medical informatics*, 8(3):e17984, 2020.
16. Douglas G Altman, Yvonne Vergouwe, Patrick Royston, and Karel GM Moons. Prognosis and prognostic research: validating a prognostic model. *Bmj*, 338, 2009.
17. Russell A Poldrack, Grace Huckins, and Gael Varoquaux. Establishment of best practices for evidence for prediction: a review. *JAMA psychiatry*, 77(5):534–540, 2020.
18. Gaël Varoquaux and Olivier Colliot. Evaluating machine learning models and their diagnostic value, 2022.
19. Jeremy C Wyatt and Douglas G Altman. Commentary: Prognostic models: clinically useful or quickly forgotten? *Bmj*, 311(7019): 1539–1541, 1995.
20. Mark Alan Fontana, Stephen Lyman, Gourab K Sarker, Douglas E Padgett, and Catherine H MacLean. Can machine learning algorithms predict which patients will achieve minimally clinically important differences from total joint arthroplasty? *Clinical orthopaedics and related research*, 477(6):1267, 2019.
21. Jonathan M. Snowden, Sherri Rose, and Kathleen M. Mortimer. Implementation of G-computation on a simulated data set: demonstration of a causal inference technique. *American Journal of Epidemiology*, 173(7):731–738, April 2011.
22. Matthew Sperrin, David Jenkins, Glen P Martin, and Niels Peek. Explicit causal reasoning is needed to prevent prognostic models being victims of their own success. *Journal of the American Medical Informatics Association*, 26(12):1675–1676, 2019.
23. Tony Blakely, John Lynch, Koen Simons, Rebecca Bentley, and Sherri Rose. Reflection on modern methods: when worlds collide—prediction, machine learning and causal inference. *International journal of epidemiology*, 49(6):2058–2064, 2020.
24. MA Hernán and JM Robins. *Causal Inference: What If.* CRC Boca Raton, FL.
25. Tyler J VanderWeele. Principles of confounder selection. *European journal of epidemiology*, 34:211–219, 2019.
26. James M. Robins and Sander Greenland. The role of model selection in causal inference from non experimental data. *American Journal of Epidemiology*, 123(3):392–402.
27. T. Wendling, K. Jung, A. Callahan, A. Schuler, N. H. Shah, and B. Gallego. Comparing methods for estimation of heterogeneous treatment effects using observational data from health care databases. *Statistics in Medicine*, (23):3309–3324.
28. Paul R Rosenbaum and Donald B Rubin. The central role of the propensity score in observational studies for causal effects. *Biometrika*, 70:41–55.
29. Peter C. Austin and Elizabeth A. Stuart. Moving towards best practice when using inverse probability of treatment weighting (IPTW) using the propensity score to estimate causal treatment effects in observational studies. *Statistics in Medicine*, 34(28):3661–3679.
30. Sabrina Casucci, Li Lin, Sharon Hewner, and Alexander Nikolaev. Estimating the causal effects of chronic disease combinations on 30-day hospital readmissions based on observational medicaid data. *Journal of the American Medical Informatics Association*, 25(6): 670–678, 2018.
31. Elysia Grose, Samuel Wilson, Jeffrey Barkun, Kimberly Bertens, Guillaume Martel, Fady Balaa, and Jad Abou Khalil. Use of propensity score methodology in contemporary high-impact surgical literature. *Journal of the American College of Surgeons*, 230(1):101–112.e2.
32. Xiaogang Su, Annette T Peña, Lei Liu, and Richard A Levine. Random forests of interaction trees for estimating individualized treatment effects in randomized trials. *Statistics in medicine*, 37(17):2547–2560, 2018.
33. Andrea Lamont, Michael D Lyons, Thomas Jaki, Elizabeth Stuart, Daniel J Feaster, Kukatharmini Tharmaratnam, Daniel Oberski, Hemant Ishwaran, Dawn K Wilson, and M Lee Van Horn. Identification of predicted individual treatment effects in randomized clinical trials. *Statistical methods in medical research*, 27(1):142–157, 2018.

34. Jeroen Hoogland, Joanna IntHout, Michail Belias, Maroeska M Rovers, Richard D Riley, Frank E. Harrell Jr, Karel GM Moons, Thomas PA Debray, and Johannes B Reitsma. A tutorial on individualized treatment effect prediction from randomized trials with a binary endpoint. *Statistics in medicine*, 40(26):5961–5981, 2021.
35. Jennifer L. Hill. Bayesian nonparametric modeling for causal inference. *Journal of Computational and Graphical Statistics*, (1):217–240.
36. Mark J. van der Laan and Sherri Rose. *Targeted Learning*. Springer Series in Statistics.
37. Megan S. Schuler and Sherri Rose. Targeted maximum likelihood estimation for causal inference in observational studies. *American Journal of Epidemiology*, 185(1):65–73.
38. Scott Powers, Junyang Qian, Kenneth Jung, Alejandro Schuler, Nigam H. Shah, Trevor Hastie, and Robert Tibshirani. Some methods for heterogeneous treatment effect estimation in high dimensions. *Statistics in Medicine*, 37(11):1767–1787.
39. Stefan Wager and Susan Athey. Estimation and inference of heterogeneous treatment effects using random forests. *Journal of the American Statistical Association*, 113(523):1228–1242.
40. Susan Athey, Julie Tibshirani, and Stefan Wager. Generalized random forests. *Annals of Statistics*, 47(2):1148–1178.
41. Sören R. Künzel, Jasjeet S. Sekhon, Peter J. Bickel, and Bin Yu. Metalearners for estimating heterogeneous treatment effects using machine learning. *Proceedings of the National Academy of Sciences*, 116(10):4156–4165.
42. Xinkun Nie and Stefan Wager. Quasi-oracle estimation of heterogeneous treatment effects. *Biometrika*, 108(2):299–319.
43. Victor Chernozhukov, Denis Chetverikov, Mert Demirer, Esther Duflo, Christian Hansen, Whitney Newey, and James Robins. Double/debiased machine learning for treatment and structural parameters. *The Econometrics Journal*, page 71.
44. Gang Fang, Izabela E Annis, Jennifer Elston-Lafata, and Samuel Cykert. Applying machine learning to predict real-world individual treatment effects: insights from a virtual patient cohort. *Journal of the American Medical Informatics Association*, 26(10):977–988, 2019.
45. Vincent Dorie, Jennifer Hill, Uri Shalit, Marc Scott, and Dan Cervone. Automated versus do-it-yourself methods for causal inference: Lessons learned from a data analysis competition. *Statistical Science*, 34(1):43–68, 2019.
46. Florent Le Borgne, Arthur Chatton, Maxime Léger, Rémi Lenain, and Yohann Foucher. G-computation and machine learning for estimating the causal effects of binary exposure statuses on binary outcomes. *Scientific reports*, 11(1):1435, 2021.
47. Jinma Ren, Paul Cislo, Joseph C Cappelleri, Patrick Hlavacek, and Marco DiBonaventura. Comparing g-computation, propensity score-based weighting, and targeted maximum likelihood estimation for analyzing externally controlled trials with both measured and unmeasured confounders: a simulation study. *BMC Medical Research Methodology*, 23(1):18, 2023.
48. Jean Kaddour, Aengus Lynch, Qi Liu, Matt J Kusner, and Ricardo Silva. Causal machine learning: A survey and open problems. arxiv 2022. *arXiv preprint arXiv:2206.15475*, 2022.
49. Victor Chernozhukov, Christian Hansen, Nathan Kallus, Martin Spindler, and Vasilis Syrgkanis. Applied causal inference powered by ml and ai. *arXiv preprint arXiv:2403.02467*, 2024.
50. Alejandro Schuler, Michael Baiocchi, Robert Tibshirani, and Nigam Shah. A comparison of methods for model selection when estimating individual treatment effects. *arXiv:1804.05146 [cs, stat]*.
51. Ahmed Alaa and Mihaela Van Der Schaar. Validating causal inference models via influence functions. *International Conference on Machine Learning*, pages 191–201.
52. Mary E. Charlson, Peter Pompei, Kathy L. Ales, and C.Ronald MacKenzie. A new method of classifying prognostic comorbidity in longitudinal studies: Development and validation. *Journal of Chronic Diseases*, 40(5):373–383.
53. James Robins. A new approach to causal inference in mortality studies with a sustained exposure period—application to control of the healthy worker survivor effect. *Mathematical Modelling*, 7(9):1393–1512.
54. Ashley I Naimi and Brian W Whitcomb. Defining and identifying average treatment effects. *American Journal of Epidemiology*, 2023.
55. Guido W. Imbens and Donald B. Rubin. *Causal inference in statistics, social, and biomedical sciences*. Cambridge University Press.
56. Donald B Rubin. Causal inference using potential outcomes. *Journal of the American Statistical Association*, 100(469):322–331.
57. P. M. Robinson. Root-n-consistent semiparametric regression. *Econometrica*, (4):931–954.
58. Mark J van der Laan, MJ Laan, and JM Robins. *Unified methods for censored longitudinal data and causality*. Springer Science & Business Media.
59. Peter Schulam and Suchi Saria. Reliable decision support using counterfactual models. *Advances in neural information processing systems*, 30.
60. Uri Shalit, Fredrik D Johansson, and David Sontag. Estimating individual treatment effect: generalization bounds and algorithms. In *International Conference on Machine Learning*, pages 3076–3085. PMLR, 2017.
61. Matthieu Doutreligne and Gael Varoquaux. How to select predictive models for decision-making or causal inference? - caussim (version 1). [computer software]. https://archive.softwareheritage.org/swh:1:snp:d5621316ebbef4b5759ee54e4893a2954913134e;origin=https://github.com/soda-inria/caussim, 2025.
62. Matthieu Doutreligne and Gael Varoquaux. How to select predictive models for decision-making or causal inference? - causal model selection (version 1). [computer software]. https://archive.softwareheritage.org/swh:1:snp:36b289292192d9957b7b99f44654ab40fc49815d;origin=https://github.com/soda-inria/causal_model_selection, 2025.
63. Chanelle J. Howe, Stephen R. Cole, Daniel J. Westreich, Sander Greenland, Sonia Napravnik, and Joseph J. Eron. Splines for trend analysis and continuous confounder control. 22(6):874–875.
64. Aris Perperoglou, Willi Sauerbrei, Michal Abrahamowicz, and Matthias Schmid. A review of spline function procedures in r. 19(1):46.
65. Ali Rahimi and Benjamin Recht. Random features for large-scale kernel machines. In *Advances in Neural Information Processing Systems*, volume 20.
66. Lingjie Shen, Gijs Geleijnse, and Maurits Kaptein. Rctrep: An r package for the validation of estimates of average treatment effects. *Journal of Statistical Software*, 2023.
67. Kenneth R. Niswander and United States National Institute of Neurologiacal Diseases and Stroke. *The Women and Their Pregnancies: The Collaborative Perinatal Study of the National Institute of Neurological Diseases and Stroke*. National Institute of Health. Google-Books-ID: A0bdVhlhDQkC.
68. Yishai Shimoni, Chen Yanover, Ehud Karavani, and Yaara Goldschmnidt. Benchmarking framework for performance-evaluation of causal inference analysis. *arXiv:1802.05046 [cs, stat]*.
69. M. F. MacDorman and J. O. Atkinson. Infant mortality statistics from the linked birth/infant death data set–1995 period data. *Monthly Vital Statistics Report*, 46(6 Suppl 2):1–22, February 1998.

70. Christos Louizos, Uri Shalit, Joris Mooij, David Sontag, Richard Zemel, and Max Welling. Causal effect inference with deep latent-variable models. *Advances in neural information processing systems*.
71. Douglas Almond, Kenneth Y. Chay, and David S. Lee. The Costs of Low Birth Weight. *The Quarterly Journal of Economics*, 120(3), 2005.
72. Fabian Pedregosa, Gaël Varoquaux, Alexandre Gramfort, Vincent Michel, Bertrand Thirion, Olivier Grisel, Mathieu Blondel, Peter Prettenhofer, Ron Weiss, Vincent Dubourg, Jake Vanderplas, Alexandre Passos, David Cournapeau, Matthieu Brucher, Matthieu Perrot, and Édouard Duchesnay. Scikit-learn: Machine learning in python. *Journal of Machine Learning Research*, 12(85):2825–2830.
73. M. G. Kendall. A new measure of rank correlation. *Biometrika*, 30(1-2):81–93, June 1938.
74. Susan Athey and Guido Imbens. Recursive partitioning for heterogeneous causal effects. *Proceedings of the National Academy of Sciences*, 113(27):7353–7360, 2016.
75. Pierre Gutierrez and Jean-Yves Gerardy. Causal inference and uplift modeling a review of the literature. *Proceedings of The 3rd International Conference on Predictive Applications and APIs*, (67):14.
76. John C. Platt and John C. Platt. Probabilistic Outputs for Support Vector Machines and Comparisons to Regularized Likelihood Methods. *Advances in Large Margin Classifiers*, pages 61–74, 1999.
77. Bianca Zadrozny and Charles Elkan. Obtaining calibrated probability estimates from decision trees and naive Bayesian classifiers. page 8.
78. Alexandru Niculescu-Mizil and Rich Caruana. Predicting good probabilities with supervised learning. In *Proceedings of the 22nd international conference on Machine learning - ICML '05*, pages 625–632. ACM Press, 2005.
79. Matthias Minderer, Josip Djolonga, Rob Romijnders, Frances Hubis, Xiaohua Zhai, Neil Houlsby, Dustin Tran, and Mario Lucic. Revisiting the Calibration of Modern Neural Networks. *Advances in Neural Information Processing Systems*, 34:15682–15694, 2021.
80. Alexandre Perez-Lebel, Marine Le Morvan, and Gaël Varoquaux. Beyond calibration: estimating the grouping loss of modern neural networks. *arXiv preprint arXiv:2210.16315*, 2022.
81. Yishai Shimoni, Chen Yanover, Ehud Karavani, and Yaara Goldschmnidt. Ibm causal inference benchmarking data. `https://www.synapse.org/Synapse:syn11738767`, 2018.
82. Matthieu Doutreligne. Twins dataset used for experiment in the paper how to select predictive models for causal inference? [data set]. `https://doi.org/10.5281/zenodo.14674618`, 2025.
83. Matthieu Doutreligne. How to select predictive models for decision making or causal inference? experiments data [data set]. `https://doi.org/10.5281/zenodo.13765465`, 2024.
84. Xavier Bouthillier, Pierre Delaunay, Mirko Bronzi, Assya Trofimov, Brennan Nichyporuk, Justin Szeto, Nazanin Mohammadi Sepahvand, Edward Raff, Kanika Madan, Vikram Voleti, Samira Ebrahimi Kahou, Vincent Michalski, Tal Arbel, Chris Pal, Gael Varoquaux, and Pascal Vincent. Accounting for variance in machine learning benchmarks. *Proceedings of Machine Learning and Systems*, 3: 747–769.
85. Alexander D'Amour, Peng Ding, Avi Feller, Lihua Lei, and Jasjeet Sekhon. Overlap in observational studies with high-dimensional covariates. *Journal of Econometrics*, 221(2):644–654, 2021.
86. Craig A. Rolling and Yuhong Yang. Model selection for estimating treatment effects. 76(4):749–769.
87. Yuta Saito and Shota Yasui. Counterfactual cross-validation: Stable model selection procedure for causal inference models. In *International Conference on Machine Learning*, pages 8398–8407. PMLR, 2020.
88. Fredrik D Johansson, Uri Shalit, Nathan Kallus, and David Sontag. Generalization bounds and representation learning for estimation of potential outcomes and causal effects. *The Journal of Machine Learning Research*, 23(1):7489–7538, 2022.
89. Dylan J Foster and Vasilis Syrgkanis. Orthogonal statistical learning. *The Annals of Statistics*, 51(3):879–908, 2023.
90. Andrew Jesson, Sören Mindermann, Uri Shalit, and Yarin Gal. Identifying causal-effect inference failure with uncertainty-aware models. *Advances in Neural Information Processing Systems*, 33:11637–11649.
91. Peter C. Austin. An Introduction to Propensity Score Methods for Reducing the Effects of Confounding in Observational Studies. *Multivariate Behavioral Research*, (3):399–424, May 2011.
92. Arthur Gretton, Karsten M Borgwardt, Malte J Rasch, Bernhard Schölkopf, and Alexander Smola. A kernel two-sample test. *The Journal of Machine Learning Research*, 13(1):723–773.
93. Bharath K. Sriperumbudur, Kenji Fukumizu, Arthur Gretton, Bernhard Schölkopf, and Gert R. G. Lanckriet. On integral probability metrics, \phi-divergences and binary classification. *arXiv:0901.2698 [cs, math]*.
94. Alicia Curth, David Svensson, and James Weatherall. Really doing great at estimating CATE? a critical look at ML benchmarking practices in treatment effect estimation. *Neurips Process 2021*, page 14.
95. Mark J. van der Laan, Eric C. Polley, and Alan E. Hubbard. Super learner. 6.
96. Adith Swaminathan and Thorsten Joachims. Counterfactual risk minimization: Learning from logged bandit feedback. In *International Conference on Machine Learning*, pages 814–823. PMLR, 2015.
97. Edward L Ionides. Truncated importance sampling. *Journal of Computational and Graphical Statistics*, 17(2):295–311.
98. Yishai Shimoni, Ehud Karavani, Sivan Ravid, Peter Bak, Tan Hung Ng, Sharon Hensley Alford, Denise Meade, and Yaara Goldschmidt. An evaluation toolkit to guide model selection and cohort definition in causal inference. *arXiv preprint arXiv:1906.00442*, 2019.
99. Keith Battocchi, Eleanor Dillon, Maggie Hei, Greg Lewis, Paul Oka, Miruna Oprescu, and Vasilis Syrgkanis. EconML: A Python Package for ML-Based Heterogeneous Treatment Effects Estimation. https://github.com/microsoft/EconML.
100. Nicolas Loiseau, Paul Trichelair, Maxime He, Mathieu Andreux, Mikhail Zaslavskiy, Gilles Wainrib, and Michael G. B. Blum. External control arm analysis: an evaluation of propensity score approaches, G-computation, and doubly debiased machine learning. *BMC Medical Research Methodology*, 22, 2022.
101. Zijun Gao, Trevor Hastie, and Robert Tibshirani. Assessment of heterogeneous treatment effect estimation accuracy via matching. *Statistics in Medicine*, (17), 2021.
102. Ashley I Naimi, Alan E Mishler, and Edward H Kennedy. Challenges in obtaining valid causal effect estimates with machine learning algorithms. *American Journal of Epidemiology*, 2021.
103. Susan Gruber and Mark J. van der Laan. tmle: An R package for targeted maximum likelihood estimation. *Journal of Statistical Software*, 51(13):1–35, 2012.
