# Supplementary material for: How to select predictive models for decision-making or causal inference
Source: Gigascience. 2025 Mar 21;14:giaf016. doi: 10.1093/gigascience/giaf016 (PMC11927402; doi:10.1093/gigascience/giaf016)

GigaScience

How to select predictive models for decision-making or causal inference?

--Manuscript Draft--

**Manuscript Number:**	GIGA-D-24-00240R1
**Full Title:**	How to select predictive models for decision-making or causal inference?
**Article Type:**	Research
**Funding Information:**	
**Abstract:**	Background: We investigate which procedure selects the predictive model most trustworthy to reason on the effect of an<br>intervention and support decision making.<br>Methods: We study a large variety of model selection procedures in practical settings: finite samples settings and without<br>theoretical assumption of well-specified models. Beyond standard cross-validation or internal validation procedures, we also study<br>elaborate causal risks. These build proxies of the causal error using “nuisance” re-weighting to compute it on the observed data.<br>We evaluate whether empirically estimated nuisances, which are necessarily noisy, add noise to model selection. We compare<br>different metrics for causal model selection in an extensive empirical study based on a simulation and three healthcare datasets<br>based on real covariates.<br>Results: Among all metrics, the mean squared error, classically used to evaluate predictive modes, is worse. Re-weighting it with<br>propensity score does not bring much improvements in most cases. On average, the R-risk, which uses as nuisances a model of<br>mean outcome and propensity scores, leads to the best performances. Nuisance corrections are best estimated with flexible<br>estimators such as a super learner.<br>Conclusions: When predictive models are used to reason on the effect of an intervention, they must be evaluated with different<br>procedures than standard predictive settings; using the R-risk from causal inference.
**Corresponding Author:**	Matthieu Doutreligne<br>Inria Saclay: Inria Centre de Recherche Saclay-Ile-de-France<br>Palaiseau, FRANCE
**Corresponding Author Secondary Information:**	
**Corresponding Author's Institution:**	Inria Saclay: Inria Centre de Recherche Saclay-Ile-de-France
**Corresponding Author's Secondary Institution:**	
**First Author:**	Matthieu Doutreligne
**First Author Secondary Information:**	
**Order of Authors:**	Matthieu Doutreligne
	Gaël Varoquaux
**Order of Authors Secondary Information:**	
**Response to Reviewers:**	We thank the reviewers for their time, thoughts, and feedback on the<br>manuscript. We have addresed the comments through additional work as well<br>as clarifications in the revised manuscript.<br><br>To clarify the origin of the Robinson decomposition, as suggested by<br>reviewer 1, we have added an equation. As a result the equation numbers<br>in the revised manuscript are all shifted by one.<br><br># Reviewer 1

Powered by Editorial Manager® and ProduXion Manager® from Aries Systems Corporation

[...] The authors conceivably state that "The problem is that causal estimation requires controlling an error on both treated and non-treated outcome for the same individual.". Consequently, they report on estimator that attempt to consider this (in Section "Methods"). However, I cannot understand how this hint ("controlling error on both treated and non-treated sample (or patient?)") has been implemented by the procedure the authors describe in Equations (1), (2), and (3). If, I correctly understand", in Eq. (2) we see the g-computation estimators, a well-known concept introduced by [21] (and recently used also by https://www.nature.com/articles/s41598-020-65917-x), that splits the two configurations "treated" and "non-treated". [...] I understand that Eq. (2) and in Eq. (3) consider both treated and non-treated sample (or patient?), but those sums can be also equal to 0, even if their single terms are different from 0. Moreover, it is not clear if, and how Eq. (3) is related to Eq. (2): is it a variant? Is Eq. (3) derived from Eq. (2) under special assumptions?

> The reviewer is right that Eq. (2) is part of constructing the classic
> g-computation estimator, as well as Eq. (1) and Eq. (3). We added a
> sentence at the begining of the section to stress that it is a section
> summarizing a classic framework.

> Eq. (3) (now 4) is an expression of the estimator of the ATE based on a model of the outcome, sometimes called g-estimation. It is unbiased under consistency (Assumption. 3) and unconfoundedness (Assumption 1). To prove that, take the expectation of Eq. (3). Then apply Eq. (1) (now 2) which is true under consistency (Eq. 1 and Assumption 1) and unconfoundedness (Assumption 3). Then by the law of total expectancy, the conditionnality on X disappear and we recover Eq. (2) (now 3)

> G-estimation itself does not control error on both populations. The formulas in Eq. (1-3) (now 2-4) are all partly oracle formulas: they rely on conditional expectations but give no specific procedures on how to compute them. We added a clarification on this topic above Eq. (4) (now 5) and above Eq. (8) (now 9).

Furthermore, the Robinson decomposition (R-decomposition) should be better discussed and its role in the context of causal inference should be better commented. R-decomposition introduces two quantities, the conditional mean outcome and the probability to be treated (known as propensity score). What is the role of the propensity score? How is it calculated? Is it calculated simply by a frequentist approach (number of treated/total number of patients)?

> G-computation relies on one choice of decomposition of the CATE
> estimation (Eq. (2)), which leads to into a sequence of regressions,
> where the only statistical estimate is the response function μa(x)
> [Chernozhukov et al., 2024, chapter 15](https://causalml-book.org/). We
> have clarified that this decomposition is just a mathematical
> rewriting. The mean outcome from the treatment effect and the
> propensity score are two statistical quantity that enable this
> rewritting . Any statistical estimator can be chosen to model these
> quantities, in particular in the section were we introduce them, they
> are written in term of expectations, not detailing an estimation
> procedure, as this is discussed later. We have clarified this below
> formula 4 (formely 3) and in the begining of the section containing
> formula 9 (formely 8). A frequentist approach counting ratios of
> patients is one such estimator, but it will work only for a small
> number of discrete covariate. In practice, researchers often estimate
> the propensity score with a logistic regression, but other methods such
> as Boosted trees, Random Forests, or Neural Networks can be used. A
> good ps estimator should be well calibrated but this question is out of
> scope for this paper. In our discussion, we briefly mentioned this
> interesting research question on what properties should have a good
> estimator for the propensity score.

*Finally, I note that the paper mentions inference methods specific to machine learning (random forest, etc.). In this regard, there is a great deal of study in the literature as to their actual usefulness in this field. I suggest the authors consider/review some of the

Powered by Editorial Manager® and ProduXion Manager® from Aries Systems Corporation

following literature in the paper (those that the authors consider most appropriate to be included in their manuscript):*

https://link.springer.com/book/10.1007/978-3-031-35051-1

https://www.causal-machine-learning.com/

https://www.frontiersin.org/journals/bioinformatics/articles/10.3389/fbinf.2021.746712/full

https://www.research-collection.ethz.ch/handle/20.500.11850/650703

https://aisel.aisnet.org/pacis2022/181/

or some other that can be easily found in literature.

> We thank the reviewer for the references. We have added several
> references for Causal ML into the manuscript, in particular [Kaddour et al., 2022](https://arxiv.org/pdf/2206.15475), corresponding to
> https://www.causal-machine-learning.com/, and [Chernozhukov et al., 2024](https://causalml-book.org/) which both cover most of the
> subfields at the intersection of causal inference and machine learning.

Reviewer 2

Overall assessment:

Let me begin with positive comments. I think the paper is very important because it describes a sophisticated methodology for experimental examination of causal models. Despite some attempts this is still lacking in literature. I especially like the simulation scheme which uses different features for data generation and learning models. Moreover thorough experimental evaluations of causal models are not very frequent making the paper important. Code is available on Github which is a big plus.

I am however not sure about the overall conclusions, which are that R-risk is always the best. Performance of risk functions may depend on several factors and the overall best performance might be a result of bias in available data. The only factor taken into account by the authors is overlap. However, only one type of lack of overlap is tested in experiments, based on shifted Gaussians. This will likely lead to violation of the positivity assumption. How about different scenarios, e.g.: perfect overlap but different treatment/control distributions? Confounding need not necessarily lead to lack of overlap. Another possible factor is the ratio of causal effect to background responses. It is known that it may affect relative performance of HTE estimators.

Overall: I am no fully convinced that R-risk is always the best. The answer could be dependent on other factors and I hope the authors would explore more scenarios.

> We thank the reviewer for the thoughful comments and the call for
> nuance. Our first response is that our thoughts did not come out right
> and that it is not clear, indeed, that R-risk is always the best.
> Rather, that it is a good default solution. We have reformulate parts of
> the paper to convey this nuance better and added the nuance in the
> conclusion.

> In addition, the reviewer is correct that factors beyond overlap
> probably affect which risk is best. We have followed the suggestion to
> explore more diverse simulation scenarii, as detailing the sensitivity
> of our result on the R-risk makes a more nuanced and stronger analysis.
> Thus, we ran a dedicated experiment focusing on the ratio of causal
> effect to background response. We measure the ratio by the absolute
> mean difference between the causal effect and the baseline response,
> measured empirically on each dataset instance $\Delta_{\mu} =
> \frac{1}{N} \sum_{i=1}^N \big | \frac{\mu_{1}(x_i) -
> \mu_{0}(x_i)}{\mu_{0}(x_i) + \mu_{1}(x_i) - \frac{1}{N} \sum_{j=1}^N \mu_{0}(x_i) +

\mu_{1}(x_i)} \big|$. We vary a dedicated parameter
> (ω in Algorithm 2) in the simulation to force this ratio to
> cover a wide range of values. In 90 dataset instance, it ranges several
> orders of magnitude from 0.04 to 206. Figure 7 gives the main results
> of this experiment. We see that R-risk is still the best risk on
> average, but when the effect ratio is high (tertile between 9 and
> 9675), the reweighted mu-risk and the tau-risk_IPW are better than
> the R-risk. However, for low value of the ratio, the R-risk largely
> outperforms all other metrics. We added these new results to the main
> paper. We also studied another characteristic of the simulation: the variance of the causal effect measured as the empirical version of $\frac{var(\mu_1 - \mu_0)}{var(\mu_1+\mu_0)}$. For this last experiment, we found similar results to the causal effect experiment. We include it in the supp mat for completeness about our reflexion.

> We did not explore more sophisticated treatment/control distributions for our simulation. Building these new data generation processes would imply a big modification to our setup. We rely on the easy inversion of the distribution functions of the gaussians (p(X|A=a)) to analytically compute the true propensity score. In addition, we think that the semi-simulated datasets already provide some interesting argument in favor of r-risk with more more diverse treatment/control covariate distributions.

Another question: do the differences between risks in experiments follow known theoretical results? For example, it is well known (e.g. https://www.semanticscholar.org/reader/112aba9c62f98b9684693f4c20c30274bc9eb1dc or [Rudas ans jaroszewisz, 2017](https://link.springer.com/content/pdf/10.1007/s10618-018-0576-8.pdf)) that the tau risk performs poorly.

> The high variability of the U-learner has already been noted by [Nie and Wager, 2021](https://arxiv.org/pdf/1712.04912). This advantage of the R-loss that does not have a propensity score to the denominator is also discussed in [Chernozhukov et al. 2024, chapter 15.](https://causalml-book.org/assets/chapters/CausalML_chap_15.pdf). The convergence rate of the R-loss has been proven in [Foster and Syrgkanis, 2023](https://arxiv.org/pdf/1901.09036). We are not aware of theoretical results comparing simple robust losses (such as mu-risk or mu-risk IPW) to orthogonal losses such as R-loss, U-loss or DR-Loss.
> Empirically, the differences between risks have been highlighted in other papers such as [Schuler, et al., 2018](https://arxiv.org/pdf/1804.05146) for quadratic and stepwise data generation processes or [Curth et Van der Schaar, 2023](https://proceedings.mlr.press/v202/curth23b/curth23b.pdf) exploring more diverse outcome generation processes focusing on CATE sparsity (as a proxy of its complexity) based on a semi-simulated dataset for the covariate (ACIC2016). They both underline good performances for the R-risk (see Table.1 for Schuler and Fig. 3 for Curth). A remark that we made in our manuscript is that the results of Schuler are convincing for randomization setups but not for observational (non-randomized) setups. Curth's results are more convincing for observational setups but did not explore the overlap assumption.

> The second paper is from the uplift regression literature. It focus on full randomization problems and a linear specification with gaussian predictors. It shows theoretically that a linear T-learner (called double model approach in their approach) has smaller asymptotical variance than a treatment ratio reweighted outcome S learner. However, they note that in non normal cases the T-learner can be ill-defined or with high variance. They propose a corrected reweighted S-learner approach to reduce this variance. They show empirically that it behaves better than the non corrected version on a simulation and two real world problems.
> I am not sure to see the link with the tau-risk. The paper does not compare risks but corrected or uncorrected vs T-learners. In addition, they only consider full randomization and linear problems. In our case, we consider a more general setting with a variety of data generation procedures and we focus on non-randomized situations where the distribution of the treatment is conditioned by the predictors.

*Another problem is that the paper is hard to follow. The authors rapidly switch

Powered by Editorial Manager® and ProduXion Manager® from Aries Systems Corporation

between topics: risk, selection, nuisance parameter estimation so the reader quickly gets lost. The sections in supplementary materials contain important information but not all are referenced in main text. The paper gives an impression of several experiments put together in some order without guiding the reader through the maze. I think organization should be improved before publication.*

> We tried to better guide the reader through the method section and to clarify the motivation behind the different experiments.
> We checked the supplementary materials but we did not find any appendix that was not referenced at least once in the main text (several are referred in figure captions). Did you have a specific appendix in mind that would require a reference in the main text?

Other comments:

In the Methods section g-computation is described. It seems indeed the first name under which the technique appeared in literature, but other names, most importantly S-learner are being used frequently nowadays. Interestingly those names are mentioned in Empirical study on page 4, however, the close relationship (equivalence?) between S-learner and g-computation is not mentioned.

> G-computation is larger than S-learner. The first mention of S-learner that we are aware of is [Künzel, 2019](https://arxiv.org/pdf/1706.03461). In this paper, they distinguish T-learners from S-learners (or more elaborated ones). T-learners model $\mu(0)$ and $\mu(1)$ separately and train separately on each population. On the contrary S learner use only one model for both population and add the treatment as a covariate. A good example to understand the difference is to note that a linear T-learner is the same thing as a linear S-learner where we add an interaction term between the treatment and the covariates. Ultimately, both methods are G-computation in the sense that they rely on the identification of eq. (1) making the link between potential outcomes and observed quantities. To improve the manuscript, we added just after the eq. (3) that one can choose to model the response function with either a T-learner or a S-learner.

Equation 2: should the expectation be over X not Y?

> Thanks for the remark. We corrected a typo in the equation. There should not have any mention of X in the conditional expectation.

page 17: proposition number is missing

> Thanks for this remark. We fixed it.

A.7: the data generation procedure needs better explanations. Why not call (7) P(d|A) since that's how its used later? On page 17 you say '[b1,bD]~P(x)' and later 'x~P(x)' which causes confusion. Also, how are beta vectors generated?

> We assumed that you talked about the equation 17 rather than 7. Could you point where we use the P(d|A) notation? We did not find it in the manuscript.
> The notation b1, ..bD ~P(x) was meant to be understood as the beta vectors are drawn from a distribution P(x). The later mention of 'x~P(x)' was meant to be understood as the definition if the RBF function. We removed the second mention which causes confusion.
> The beta vectors are drawn from two normal distributions with mean 0 and unit variances. We added this information in the manuscript.
> We added the Algorithm 2, hopping that it would help the reader to understand exactly how we generate the data for our simulation.

page 4: TLearner uses different feature representation for each treatment arm. Does this really happen in practice? Usually all subgroups are described with the same set of features.

> It is indeed true that in usual practice all subgroups are described with the same set

Powered by Editorial Manager® and ProduXion Manager® from Aries Systems Corporation

	of features. However, as soon as we are using a machine learning (necessary to model CATE) such as a random forest or boosted trees, the inner representations of the features inside the model is different for both population. This idea has been exploited to build representation aware neural net models for cate estimation by [Johansson et al., 2022](https://www.jmlr.org/papers/volume23/19-511/19-511.pdf). It is also the case for simpler models such as a linear model with treatment interaction and lasso regularization.<br><br>*page 4/5: Family of candidate estimators: why are only linear models used in some dataset and forests for other? The leaf sizes of forests are chosen from a rather small range, why not use some percentage of the size of the training set?*<br><br>> For the simulations we chose estimators that are out of the functional class of the true data generation process (a candidate estimator cannot model exactly the data generation process) but that are quite similar to it. They are out of the functional class because the representers are different from the one of the true data (they are two randomly sampled data points). They are close because they have the same functional form : a linear model on top of two data representers. We used the simulations to explore the wider range of data generation processes, focusing on a gradient of overlap. Because of the sheer number of generated dataset, we did not include computationally costly boosted trees in the candidate family.<br><br>> The choice of the candidate family of estimators was guided by the idea to compare estimators that are not too different from each other, making the model selection task more challenging. The important point is that we are not focusing on the best possible estimator but on the best possible model selection procedure. To identify the optimal procedure, it is more convincing to compare close candidate estimators than a wide variety of candidate estimators. In the later case, one estimator could dominate all other estimators for every selection procedure. This explains why we are choosing a narrow range of leaf sizes for the forests.<br><br><br>Language comments:<br>------------------<br><br>*It seems that English might need improvement in some cases, for<br>example:*<br><br>*Abstract: "We study such a variety of model selection procedures in practical settings": a "large variety" seems more appropriate.*<br><br>*On page 1, the sentence "Predictive modeling bridges to causal inference theory under the name of outcome models ..." contains "bridges under the name" which also sounds strange.*<br><br>*On page 2: In the sentence "Then we anchor causal model selection in the potential outcome framework and details the causal risks and model-selection procedure." I believe 'details' should be 'detail'.*<br><br>*Algorithm 1: 'a candidate estimators f' should be 'a candidate estimator f'*<br><br>*throughout: "an RBF expansion" is more commonly used than "a RBF expansion"*<br><br>*page 4: 'for both population' should be 'for both populations'*<br><br>> Thanks for the remarks. We corrected the typos and improved the language as suggested.
**Additional Information:**	
**Question**	**Response**
Are you submitting this manuscript to a special series or article collection?	No

Powered by Editorial Manager® and ProduXion Manager® from Aries Systems Corporation

**Experimental design and statistics**<br><br>Full details of the experimental design and statistical methods used should be given in the Methods section, as detailed in our Minimum Standards Reporting Checklist. Information essential to interpreting the data presented should be made available in the figure legends.<br><br>Have you included all the information requested in your manuscript?	Yes
**Resources**<br><br>A description of all resources used, including antibodies, cell lines, animals and software tools, with enough information to allow them to be uniquely identified, should be included in the Methods section. Authors are strongly encouraged to cite Research Resource Identifiers (RRIDs) for antibodies, model organisms and tools, where possible.<br><br>Have you included the information requested as detailed in our Minimum Standards Reporting Checklist?	Yes
**Availability of data and materials**<br><br>All datasets and code on which the conclusions of the paper rely must be either included in your submission or deposited in publicly available repositories (where available and ethically appropriate), referencing such data using a unique identifier in the references and in the "Availability of Data and Materials" section of your manuscript.<br><br>Have you have met the above requirement as detailed in our Minimum Standards Reporting Checklist?	Yes

Powered by Editorial Manager® and ProduXion Manager® from Aries Systems Corporation

LaTeX - Main Document (TeX file)

Click here to access/download;LaTeX - Main Document (TeX file);how_to_select_latex.tex

```
This is pdfTeX, Version 3.141592653-2.6-1.40.26 (TeX Live 2024)
(preloaded format=pdflatex 2024.8.2)  10 NOV 2024 12:07
entering extended mode
 restricted \write18 enabled.
 %&-line parsing enabled.
**how_to_select_latex.tex
(./how_to_select_latex.tex
LaTeX2e <2024-06-01> patch level 2
L3 programming layer <2024-05-27>

! LaTeX Error: File `oup-contemporary.cls' not found.

Type X to quit or <RETURN> to proceed,
or enter new name. (Default extension: cls)

Enter file name:
! Emergency stop.
<read *>

l.23 ^^M

*** (cannot \read from terminal in nonstop modes)


Here is how much of TeX's memory you used:
 19 strings out of 473583
 504 string characters out of 5732343
 1925908 words of memory out of 5000000
 23012 multiletter control sequences out of 15000+600000
 558069 words of font info for 36 fonts, out of 8000000 for 9000
 1141 hyphenation exceptions out of 8191
 19i,0n,29p,115b,17s stack positions out of
10000i,1000n,20000p,200000b,200000s
!  ==> Fatal error occurred, no output PDF file produced!
```

Manuscript

Click here to access/download;Manuscript;how_to_select_latex.pdf

Placeholder for OUP logo oup.pdf

GigaScience, 2024, 1–29

doi: xx.xxxx/xxxx
Manuscript in Preparation
Paper

PAPER

How to select predictive models for decision-making or causal inference?

Matthieu Doutreligne[1,*] and Gaël Varoquaux[2]

[1]Soda, Inria Saclay, France and [2]Mission Data, Haute Autorité de Santé, France

[*]matt.dout@gmail.com; matthieu.doutreligne@inria.fr

Abstract

Background: We investigate which procedure selects the predictive model most trustworthy to reason on the effect of an intervention and support decision making.
Methods: We study a large variety of model selection procedures in practical settings: finite samples settings and without theoretical assumption of *well-specified* models. Beyond standard cross-validation or internal validation procedures, we also study elaborate causal risks. These build proxies of the causal error using "nuisance" re-weighting to compute it on the observed data. We evaluate whether empirically estimated nuisances, which are necessarily noisy, add noise to model selection. We compare different metrics for causal model selection in an extensive empirical study based on a simulation and three healthcare datasets based on real covariates.
Results: Among all metrics, the mean squared error, classically used to evaluate predictive modes, is worse. Re-weighting it with propensity score does not bring much improvements in most cases. On average, the R-risk, which uses as nuisances a model of mean outcome and propensity scores, leads to the best performances. Nuisance corrections are best estimated with flexible estimators such as a super learner.
Conclusions: When predictive models are used to reason on the effect of an intervention, they must be evaluated with different procedures than standard predictive settings; using the R-risk from causal inference.

Key words: Model Selection, Predictive model, Treatment Effect, G-computation, Machine Learning

Introduction

Extending prediction to prescription needs causality

Prediction models have long been used in biomedical settings, as with risk score or prognostic models [1, 2]. While these have historically been simple models on simple data, this is changing with progress in machine learning and richer medical data [3, 4]. Health predictions can now integrate medical images [5, 6, 7, 8, 9], patient records [10, 11, 12] or clinical notes [13, 14, 15]. Complex data is difficult to control and model, but these models are validated by verifying the accuracy of the prediction on left-out data [16, 17, 18]. Crucial to the clinical adoption of a model predicting a health outcome is that it "can support decisions about patient care" [19]. Precision medicine is about guiding decisions: *eg* will an individual benefit from an intervention such as surgery [20]? An estimate of the effect of the treatment can be obtained by contrasting model predictions with and without the treatment, but statistical validity requires causal inference [21, 22, 23].

Indeed, concluding on the effect of a treatment is a difficult causal-inference task, as it can be easily compromised by confounding: spurious associations between treatment allocation and baseline health, *e.g.* only prescribing a drug to mild cases [24, 25]. Predictive modeling is linked to causal inference theory by the concept of *outcome models* (or g-computation, g-estimation, g-formula [26], Q-model [21], conditional mean regression [27]). Medical statistics and epidemiology have mostly used other causal-inference methods, modeling treatment assignment with propensity scores [28, 29, 30, 31]. Outcome modeling brings the benefit of going beyond average effects, estimating individualized or conditional average treatment effects (CATE), central to precision medicine. For this purpose, such methods are also invaluable for

Compiled on: November 10, 2024.
Draft manuscript prepared by the author.

randomized trials [32, 33, 34].

Outcome-modeling methods, even when specifically designed for causal inference, are numerous: Bayesian Additive Regression Trees [35], Targeted Maximum Likelihood Estimation [36, 37], causal boosting [38], causal multivariate adaptive regression splines [38], random forests [39, 40], Meta-learners [41], R-learners [42], Doubly robust estimation [43]... The wide variety of methods raises the problem of selecting between different estimators based on the data at hand. Indeed, estimates of treatment effects can vary markedly across different predictive models [44, 45, 46, 47] (illustration in Appendix A.1).

Given complex health data, which predictive model is to be most trusted to yield valid causal estimates needed to motivate individual treatment decisions? As no single machine-learning method performs best across all datasets, there is a pressing need for clear guidelines to select outcome models for causal inference.

Objectives and structure of the paper. The intersection between machine learning and causal inference is growing rapidly [48, 49]. We focus on *model selection procedures* in practical settings, without theoretical assumptions often made in statistical literature such as *infinite* data or *well-specified* models (Appendix A.2). Asymptotic causal-inference theory recommends complex risks, but a practical question is whether model-selection procedures, that rely on data split, can estimate these risks reliably enough. Indeed, these risks come with more quantities to estimate, which may bring additional variance, leading to worse model selection.

We first illustrate the problem of causal model selection. Then we anchor causal model selection in the *potential outcome* framework and detail the causal risks and model-selection procedure. We then rewrite the so-called *R*-risk as a reweighted version of mean squared difference between the true and estimated individualized treatment effect. Finally, we conduct a thorough empirical study comparing the different metrics on diverse datasets, using a family of simulations and real health data, going beyond prior work limited to specific simulation settings [50, 51] (Appendix A.2).

Illustration: the best predictor may not estimate best causal effects

Using a predictor to reason on causal effects relies on contrasting the prediction of the outcome for a given individual with and without the treatment. Given various predictors of the outcome, which one should we use? Standard predictive modeling or machine-learning practice selects the predictor that minimizes the expected error on the outcome [17, 18]. However, this predictor may not be the best model to reason about causal effects of an intervention as Figure 1 illustrates. Consider the probability Y of an undesirable outcome (*e.g.*death), a binary treatment $A \in \{0,1\}$, and a covariate $X \in \mathbb{R}$ summarizing the patient health status (*e.g.*the Charlson index [52]). We simulate a treatment beneficial (decreases mortality) for patients with high Charlson scores (bad health status) but with little effect for patients in good condition (low Charlson scores).

Figure 1a shows a random forest predictor with a counter-intuitive behavior: it predicts well on average the outcome (as measured by a regression R^2 score) but perform poorly to estimate causal quantities: the average treatment effect τ (as visible via the error $|\tau - \hat{\tau}|$) or the conditional average treatment effect (the error $\mathbb{E}[(\tau(x) - \hat{\tau}(x))^2]$, called CATE). On the contrary, Figure 1b shows a linear model with smaller R^2 score but better causal inference.

The problem is that causal estimation requires controlling an error on both treated and non-treated outcome for the same individual: the observed outcome, and the non-observed *counterfactual* one. The linear model is misspecified –the outcome functions are not linear–, leading to poor R^2; but it interpolates better to regions where there are few untreated individuals –high Charlson score– and thus gives better causal estimates. Conversely, the random

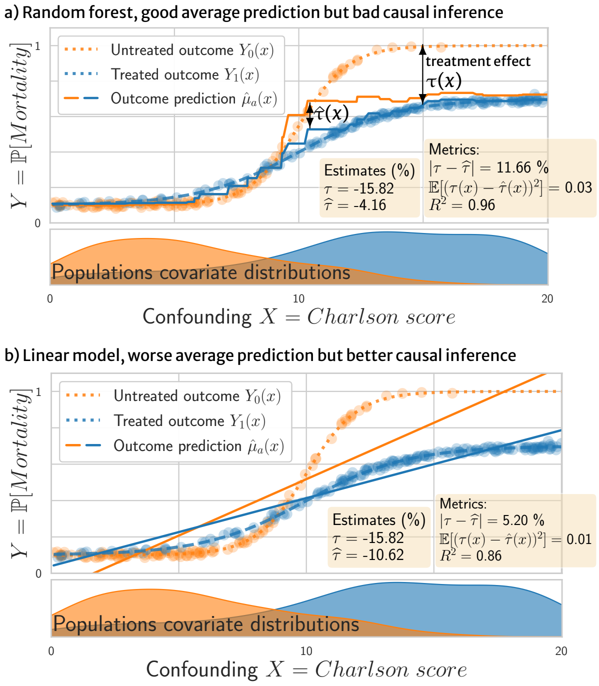

Figure 1. Illustration: a) a random-forest predictor with high performance for standard prediction (high R^2) but that yields poor causal estimates (large error between true effect τ and estimated $\hat{\tau}$), b) a linear predictor with smaller prediction performance leading to better causal estimation.

Selecting the predictor with the smallest error to the individual treatment effect $\mathbb{E}[(\tau(x) - \hat{\tau}(x))^2]$ –the τ-risk, eq. 10 – would lead to the best causal estimates; however computing this error is not feasible: it requires access to unknown quantities: $\tau(x)$.

While the random forest fits the data better than the linear model, it gives worse causal inference because its error is inhomogeneous between treated and untreated. The R^2 score does not capture this inhomogeneity.

forest puts weaker assumptions on the data, thus has higher R^2 score but is biased by the treated population in the poor-overlap region, leading to bad causal estimates.

This toy example illustrates that the classic minimum mean squared error (MSE) criterion is not suited to choosing a model among candidate estimators for causal inference.

Methods

Neyman-Rubin Potential Outcomes framework

We first expose the classic construction of the outcome modeling (or G-computation) estimators of causal effect [53, 21, 24].

Settings. The Neyman-Rubin Potential Outcomes framework [54, 55] enables statistical reasoning on causal treatment effects: Given an outcome $Y \in \mathbb{R}$ (*e.g.*mortality risk or hospitalization length), function of a binary treatment $A \in \mathcal{A} = \{0,1\}$ (*e.g.* a medical procedure), and baseline covariates $X \in \mathcal{X} \subset \mathbb{R}^d$, we observe the factual distribution, $O = (Y(A), X, A) \sim \mathcal{D} = \mathbb{P}(y, x, a)$. However, we want to model the existence of potential observations (unobserved ie. counterfactual) that correspond to a different treatment. Thus we want quantities on the counterfactual distribution $O^* = (Y(1), Y(0), X, A) \sim \mathcal{D}^* = \mathbb{P}(y(1), y(0), x, a)$.

Popular quantities of interest (estimands) are: at the population level, the Average Treatment Effect

$$\text{ATE} \qquad \tau \stackrel{\text{def}}{=} \mathbb{E}_{Y(1),Y(0)\sim\mathcal{D}^*}[Y(1) - Y(0)];$$

at the individual level, to model heterogeneity, the Conditional Average Treatment Effect

$$\text{CATE} \qquad \tau(x) \stackrel{\text{def}}{=} \mathbb{E}_{Y(1),Y(0)\sim\mathcal{D}^\star}[Y(1)-Y(0)|X=x].$$

Causal assumptions. A given data needs to meet a few assumptions to enable identifying causal estimands [56]. *1)* an individual's outcome Y is solely governed by the corresponding potential outcome:

$$\textit{Consistency}\text{ assumption,} \qquad Y = A\,Y(1) + (1-A)\,Y(0) \tag{1}$$

2) unconfoundedness $\{Y(0), Y(1)\} \perp\!\!\!\perp A|X$, *3) strong overlap* ie. every patient has a strictly positive probability to receive each treatment, and *4) generalization* –no covariate shift. These classic assumptions, called *strong ignorability*, are formally detailed in A.3.

Identifying treatment effects with outcome models – g-computation [53]. Should we know the two potential outcomes for a given X, we could compute the difference between them, which gives the causal effect of the treatment. These two potential outcomes can be estimated from observed data: the consistency 1 and unconfoundedness 2 assumptions imply the following equality, linking the target quantity to the observed data:

$$\mathbb{E}_{Y(a)\sim\mathcal{D}^\star}[Y(a)|X=x] = \mathbb{E}_{Y\sim\mathcal{D}}[Y|X=x, A=a] \tag{2}$$

On the left, the expectation is taken on the counterfactual unobserved distribution. On the right, the expectation is taken on the factual observed distribution conditionally on the treatment. For the rest of the paper, the expectations will always be taken on the factual observed distribution $\mathcal{D}$. This identification leads to outcome based estimators (ie. g-computation estimators [21]):

$$\begin{aligned}\tau &= \mathbb{E}_{Y\sim\mathcal{D}^\star}[Y(1)-Y(0)]\\ &= \mathbb{E}_{Y\sim\mathcal{D}}[Y|A=1] - \mathbb{E}_{Y\sim\mathcal{D}}[Y|A=0]\end{aligned} \tag{3}$$

This equation builds on the conditional expectation of the outcome given the treatment $\mathbb{E}_{\sim\mathcal{D}}[Y|A]$. Outcome based methods target this quantity conditionally on the covariates, called *response function*:

$$\text{Response function} \qquad \mu_a(x) \stackrel{\text{def}}{=} \mathbb{E}_{Y\sim\mathcal{D}}[Y|X=x, A=a]$$

Given a sample of data and the oracle response functions μ_0, μ_1, the finite sum version of Equation 3 leads to an unbiased estimator of the ATE written:

$$\hat{\tau} = \frac{1}{n}\left(\sum_{i=1}^{n} \mu_1(x_i) - \mu_0(x_i)\right) \tag{4}$$

This estimator is an oracle *finite sum* estimator by opposition to the population expression of τ, $\mathbb{E}[\mu_1(x_i) - \mu_0(x_i)]$, which involves an expectation taken on the full distribution $\mathcal{D}$, which is observable but requires infinite data. For each estimator ℓ taking an expectation over $\mathcal{D}$, we use the symbol $\hat{\ell}$ to note its finite sum version. The formulas in Eq. (2-4) are all partly oracle formulas: they rely on conditional expectations, the response functions, but give no specific procedures on how to compute or select them. This last point is the topic of our work, describe in the next section.

Similarly to the ATE, at the individual level Eq.2 links the CATE to statistical quantities:

$$\tau(x) = \mu_1(x) - \mu_0(x) \tag{5}$$

Robinson decomposition. G-computation is a choice of decomposition of the CATE estimation. Other choices of decomposition exist, such as the R-decomposition [57]. The latter introduces two new statistical estimates, the conditional mean outcome and the probability of being treated (known as propensity score [28]):

$$\text{Conditional mean outcome} \qquad m(x) \stackrel{\text{def}}{=} \mathbb{E}_{Y\sim\mathcal{D}}[Y|X=x] \tag{6}$$

$$\text{Propensity score} \qquad e(x) \stackrel{\text{def}}{=} \mathbb{P}[A=1|X=x] \tag{7}$$

with these, the outcome (Eq. 1) can be written

$$\begin{aligned}\text{R-decomposition} \qquad y(a) &= m(x) + \big(a - e(x)\big)\tau(x) + \varepsilon(x;a)\\ \text{with} \quad &\mathbb{E}[\varepsilon(X;A)|X,A] = 0\end{aligned} \tag{8}$$

m and e are often called *nuisances* [43]. They are unknown and must be estimated from the data.

Both the in the ATE and CATE formula Eq. (4, 5), and the Robinson decomposition involve conditional expectations –the response functions $\mu_a(x)$ or the nuisances $m(x)$ and $e(x)$. In practice those are given by statistical models: linear models, random forests, etc [48, 49].

Model-selection, oracle and feasible risks

Causal model selection. We formalize model selection for causal estimation. Thanks to the outcome model identification (Equation 2), a given model $f : \mathcal{X} \times \mathcal{A} \to \mathcal{Y}$ –learned from data or built from domain knowledge– induces feasible estimates of the ATE and CATE (eqs 4 and 5), $\hat{\tau}_f$ and $\hat{\tau}_f(x)$. However, the g-computation framework presented above is written in terms of "perfect" conditional expectations (oracles), it does not control an error, *eg* on both populations as highlighted in Figure 1. Selection procedures are needed to find the best conditional-expectation models.

A selection procedure combines a risk ℓ, evaluating the quality of a model f with observed data O, and a splitting strategy of the data to estimate different regressions (nuisances) involved in the risk. Formally, let $\mathcal{F} = \{f : \mathcal{X} \times \mathcal{A} \to \mathcal{Y}\}$ be a family of such estimators. Our goal is to select the best candidate in this family for the observed dataset O using a risk ℓ:

$$f^*_\ell = \operatorname*{argmin}_{f\in\mathcal{F}} \ell(f, O) \tag{9}$$

We now detail possible risks ℓ, risks useful for causal model selection, and how to compute them.

The τ-risk: an oracle error risk. As we would like to target the CATE, the following evaluation risk is natural (also called PEHE [59, 35]):

$$\tau\text{-risk}(f) \stackrel{\text{def}}{=} \mathbb{E}_{X\sim p(X)}[(\tau(X) - \hat{\tau}_f(X))^2] \tag{10}$$

Given observed data from $p(X)$, the expectation is computed with a finite sum, as in eq. 4, to give an estimated value $\widehat{\tau\text{-risk}}(f)$. However this risk is not feasible as the oracles $\tau(x)$ are not accessible with the observed data $(Y, X, A) \sim \mathcal{D}$.

Feasible error risks. Table 1 lists *feasible* risks (Detailed in Appendix A.4), based on the prediction error of the outcome model and *observable* quantities. These observable, called nuisances are e –propensity score, eq 7– and m –conditional mean outcome, eq 6. We give the definitions as *semi-oracles*, function of the true unknown nuisances, but later instantiate them with estimated nuisances, noted $(\check{e}, \check{m})$. Semi-oracles risks are superscripted with the $\star$ symbol.

Table 1. Review of causal risks — The R-risk* is called τ-risk$_R$ in [50].

Risk	Equation	Reference
$\tau\text{-risk} = \text{MSE}(\tau(X), \tau_f(X))$	$\mathbb{E}_{X\sim p(X)}[(\tau(X) - \hat{\tau}_f(X))^2]$	Eq. 10 [35]
$\mu\text{-risk} = \text{MSE}(Y, f(X))$	$\mathbb{E}_{(Y,X,A)\sim\mathcal{D}}\left[(Y - f(X;A))^2\right]$	Def. 1 [50]
$\mu\text{-risk}^*_{IPW}$	$\mathbb{E}_{(Y,X,A)\sim\mathcal{D}}\left[\left(\frac{A}{e(X)} + \frac{1-A}{1-e(X)}\right)(Y - f(X;A))^2\right]$	Def. 2 [58]
$\tau\text{-risk}^{\star}_{IPW}$	$\mathbb{E}_{(Y,X,A)\sim\mathcal{D}}\left[\left(Y\left(\frac{A}{e(X)} - \frac{1-A}{1-e(X)}\right) - \hat{\tau}_f(X)\right)^2\right]$	Def. 3 [39]
$U\text{-risk}^*$	$\mathbb{E}_{(Y,X,A)\sim\mathcal{D}}\left[\left(\frac{Y-m(X)}{A-e(X)} - \hat{\tau}_f(X)\right)^2\right]$	Def. 4 [42]
$R\text{-risk}^*$	$\mathbb{E}_{(Y,X,A)\sim\mathcal{D}}\left[\left((Y - m(X)) - (A - e(X))\,\hat{\tau}_f(X)\right)^2\right]$	Def. 5 [42]

Model selection procedure

Causal model selection (eq 9) may involve estimating various quantities from the observed data: the outcome model f, its induced risk as introduce in the previous section, and possibly nuisances required by the risk. Given a dataset with N samples, we split out a train and a test sets $(\mathcal{T}, \mathcal{S})$. We fit each candidate estimator $f \in \mathcal{F}$ on $\mathcal{T}$. We also fit the nuisance models $(\check{e}, \check{m})$ on the train set $\mathcal{T}$, setting hyperparameters by a nested cross-validation before fitting the nuisance estimators with these parameters on the full train set. Causal quantities are then computed by applying the fitted candidates estimators $f \in \mathcal{F}$ on the test set $\mathcal{S}$. Finally, we compute the model-selection metrics for each candidate model on the test set. This procedure is described in Algorithm 1 and Figure 2.

Algorithm 1 Model selection procedure

Given train and test sets $(\mathcal{T}, \mathcal{S}) \sim \mathcal{D}$, a candidate estimator f, a causal metrics ℓ:

i. Prefit: Learn estimators for unknown nuisance quantities $(\check{e}, \check{m})$ on the training set $\mathcal{T}$
ii. Fit: learn $\hat{f}(\cdot, a)$ on $\mathcal{T}$
iii. Model selection: $\forall x \in \mathcal{S}$ predict $\left(\hat{f}(x, 1), \hat{f}(x, 0)\right)$ and evaluate the estimator storing the metric value: $\ell(f, \mathcal{S})$ – possibly function of $\check{e}$ and $\check{m}$

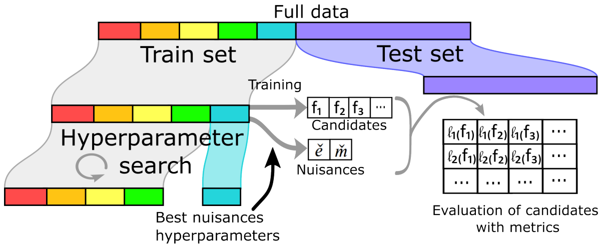

Figure 2. Estimation procedure for causal model selection.

R-risk as reweighted oracle metric

The R-risk can be rewritten as a rebalanced τ-risk.

This rewriting involves reweighted residuals: for each potential outcome, $a \in \{0;1\}$, the variance conditionally on x is [60]:

$$\sigma_y^2(x;a) \overset{\text{def}}{=} \int_y (y - \mu_a(x))^2\, p(y \mid x = x; A = a)\, dy$$

Integrating over the population, we get the Bayes squared error: $\sigma_B^2(a) = \int_{\mathcal{X}} \sigma_y^2(x;a) p(x) dx$ and its propensity weighted version: $\tilde{\sigma}_B^2(a) = \int_{\mathcal{X}} \sigma_y^2(x;a)\, p(x;a)\, dx$. In case of a purely deterministic link between the covariates, the treatment, and the outcome, these residual terms are null.

Proposition 1 (R-risk as reweighted τ-risk) *Given an outcome model f, its R-risk appears as weighted version of its τ-risk (Proof in A.5):*

$$R\text{-risk}^*(f) = \int_x e(x)(1 - e(x))\left(\tau(x) - \tau_f(x)\right)^2 p(x) dx + \tilde{\sigma}_B^2(1) + \tilde{\sigma}_B^2(0) \qquad (11)$$

The R-risk targets the oracle at the cost of an overlap reweighting and the addition of the reweighted Bayes residuals, which are independent of f. In good overlap regions the weights $e(x)(1 - e(x))$ are close to $\frac{1}{4}$, hence the R-risk is close to the desired gold-standard τ-risk. For randomized control trials, this weight is constant making the R-risk particularly suited for exploring heterogeneity (Appendix A.5)

Empirical Study

We evaluate the following causal metrics, oracle and feasible versions, presented in Table 1:
$\widehat{\mu\text{-risk}}^*_{IPW}$, $\widehat{R\text{-risk}}^*$, $\widehat{U\text{-risk}}^*$, $\widehat{\tau\text{-risk}_{IPW}}^*$, $\widehat{\mu\text{-risk}}$, $\widehat{\mu\text{-risk}}_{IPW}$, $\widehat{R\text{-risk}}$, $\widehat{U\text{-risk}}$, $\widehat{\tau\text{-risk}}_{IPW}$. We benchmark the metrics in a variety of settings: many different simulated data generation processes and three semi-simulated datasets [1].

The simulations, designed to evaluate the effect of the overlap parameter, also explore more diverse and noisy covariate distributions. They cover a diversity of causal settings such as different ratio of causal effect to background responses, and functional links between covariates, outcome and treatment.

Caussim: Extensive simulation settings

Data Generation. We use simulated data, on which the ground-truth causal effect is known. Going beyond prior empirical studies of causal model selection [50, 51], we use many generative processes, which is needed to reach general conclusions (Appendix A.7).

We generate the response functions using random bases extension, a common method in biostatistics, *e.g.*functional regression with splines [61, 62]. By allowing the function to vary at specific knots, we control the complexity of the non-linear outcome models. We use random approximation of Radial Basis Function (RBF) kernels [63] to generate the outcome and treatment functions. RBF use the same process as polynomial splines but replace polynomial by Gaussian kernels. Unlike polynomial, Gaussian kernels have decreasing influences in the input space. This avoids unrealistic divergences of the functions at the ends of the feature space. We generate 1 000 datasets based on these functions, with random overlap parameters. Example shown in Figure 13 and details in A.7.

1 Scripts for the simulations and the selection procedure are available at https://github.com/soda-inria/caussim.

Family of candidate estimators. We test model selection across different candidate estimators that approximate imperfectly the data-generating process. To build such estimators, we first use an RBF expansion similar to that used for data generation. We choose two random knots and transform the raw data features with a Gaussian kernel. This step is referred as the featurization. Then, we fit a linear regression on these transformed features. We consider two ways of combining these steps for outcome model; we use common nomenclature [41, 64] to refer to these different meta-learners that differ on how they model, jointly or not, the treated and the non treated:

- SLearner: A single learner for both populations, taking the treatment as a supplementary covariate.
- SftLearner: A single set of basis functions is sampled at random for both populations, leading to a given feature space used to model both the treated and controls, then two separate different regressors are fitted on this shared representation.
- TLearner: Two completely different learners for each population, hence separate feature representations and regressors.

For the regression step, we fit a Ridge regression on the transformed features with 6 different choices of the regularization parameter $\lambda \in [10^{-3}, 10^{-2}, 10^{-1}, 1, 10^{1}, 10^{2}]$, coupled with a TLearner or a SftLearner. We sample 10 different random basis for learning and featurization yielding a family $\mathcal{F}$ of 120 candidate estimators.

Semi-simulated datasets

Datasets. We also use three semi-simulated data adding a known synthetic causal effect to real –non synthetic– healthcare covariate. ACIC 2016 [45] is based on the Collaborative Perinatal Project [65], a RCT studying infants' developmental disorders containing 4,802 indivduals and 55 features. We used 770 dataset instances: 10 random seeds for each of the 77 simulated settings for the treatment and outcomes. ACIC 2018 [66] simulated treatment and outcomes for the Linked Births and Infant Deaths Database (LBIDD) [67] with $D = 177$ covariates. We used all 432 datasets of size $N = 5\,000$. Twins [68] is an augmentation of real data on twin births and mortality rates [69]. There are $N = 11\,984$ samples, and $D = 50$ covariates for which we simulated 1,000 different treatment allocations. Appendix A.7 gives datasets details.

Family of candidate estimators. For these three datasets, the family of candidate estimators are gradient boosting trees for both the response surfaces and the treatment [2] with S-learner, learning rate in $\{0.01, 0.1, 1\}$, and maximum number of leaf nodes in $\{25, 27, 30, 32, 35, 40\}$ resulting in a family of size 18.

Nuisance estimators. Drawing from the TMLE literature that uses combination of flexible machine learning methods [37], we model the nuisances $\check{e}$ (respectivley $\check{m}$) with a meta-learner: a stacked estimator of ridge and boosting classifiers (respectively regressions) (hyperparameter selection in Appendix A.7).

Measuring overlap between treated and non treated

Good overlap between treated and control population is crucial for causal inference (Assumption 3). We introduce the Normalized Total Variation (NTV), a divergence based on the propensity score summarizing the overlap between both populations (Appendix A.6).

2 Scikit-learn regressor, HistGradientBoostingRegressor, and classifier, HistGradientBoostingClassifier.

Results: factors driving good model selection

The R-risk is the best metric on average. Figure 3 shows the agreement between the ideal ranking of outcome models given the oracle τ-risk and the different feasible causal metrics. We measure this agreement with relative[3] Kendall tau κ (eq. 20) [70]. Given the importance of overlap in how well metrics approximate the oracle τ-risk, we separate strong and weak overlap.

Among all metrics, the classical mean squared error (ie. factual μ-risk) is worse and reweighting it with propensity score ($\mu\text{-risk}_{IPW}$) does not bring much improvements. The R-risk, which includes a model of mean outcome and propensity scores, leads to the best performances. Interestingly, the U-risk, which uses the same nuisances, deteriorates in weak overlap, probably due to variance inflation when dividing by extreme propensity scores.

Beyond rankings, the differences in terms of absolute ability to select the best model are large: The R-risk selects a model with a τ-risk only 1% higher than the best possible candidate for strong overlap on Caussim, but selecting with the μ-risk or $\mu\text{-risk}_{IPW}$ –as per machine-learning practice– leads to 10% excess risk and using $\tau\text{-risk}_{IPW}$ –as in some causal-inference methods [71, 72]– leads to 100% excess risk (Figure 16). Across datasets, the R-risk consistently decreases the risk compared to the μ-risk: from 0.1% to 1% on ACIC2016, 1% from to 20% on ACIC2018, and 0.05% from to 1% on Twins.

Model selection is harder for low population overlap. Model selection for causal inference becomes more and more difficult with increasingly different treated and control populations (Figure 4). The absolute Kendall's coefficient correlation with τ-risk drops from 0.9 (excellent agreement with oracle selection) to 0.6 on both Caussim and ACIC 2018 (15).

Nuisances can be estimated on the same data as outcome models. Using the train set $\mathcal{T}$ both to fit the candidate estimator and the nuisance estimates is a form of double dipping which can lead errors in nuisances correlated to that of outcome models [42]. In theory,

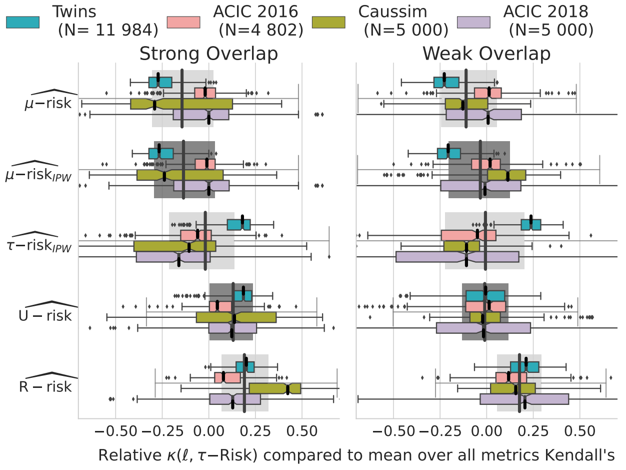

Figure 3. The R-risk is the best metric: Relative Kendall's τ agreement with τ-risk. Strong and Weak overlap correspond to the first and last tertiles of the overlap distribution measured with Normalized Total Variation eq. 17. A.7 presents the same results by adding semi-oracle risks in Figure 14, measured with absolute Kendall's in Figure 15 and with τ−risk gains in Figure 16. Table 4 gives median and IQR of the relative Kendall.

3 To remove the variance across datasets (some datasets lead to easier model selection than others), we report values for one metric relative to the mean of all metrics for a given dataset instance: Relative $\kappa(\ell, \tau\text{−risk}) = \kappa(\ell, \tau\text{−risk}) - mean_{\ell}\,(\kappa(\ell, \tau\text{−risk}))$

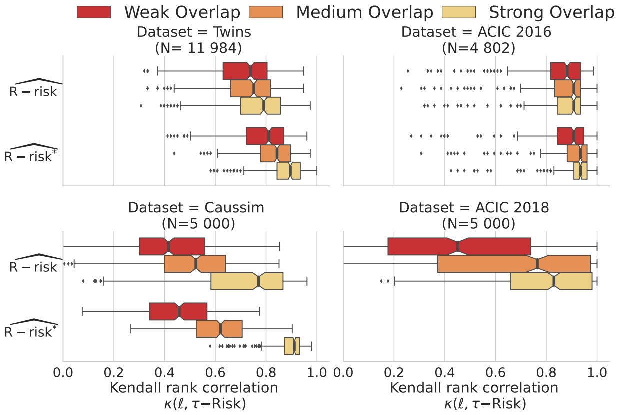

Figure 4. Model selection is harder for low population overlap: Kendall's τ agreement with τ-risk. Strong, medium and Weak overlap are the tertiles of the overlap measured with NTV eq. 17. Supplementary materials presents results for all metrics in Figure 18 in absolute Kendall's and continuous overlap values in Figure 15.

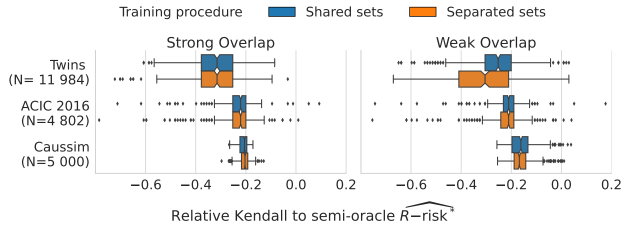

Figure 5. Nuisances can be estimated on the same data as outcome models: Results for the R-risk are similar between the shared nuisances/candidate set and the separated nuisances set procedures. Figure 17 details results for all metrics.

these correlations can bias model selection and, strictly speaking, push to split out a third separated dataset –a "nuisance set"– to fit the nuisance models. The drawback is that it depletes the data available for model estimation and selection. However, Figure 5 shows no substantial difference between a procedure with a separated nuisance set and the simpler shared nuisance-candidate set procedure.

Empirically, the best split is 90 %/10 %: using 90 % of the data to estimate both the nuisances and candidates, then computing the risks on the remaining test set for model selection (experiments in Appendix A.8).

Stacked models are good overall estimators of nuisances. Stacked nuisances estimators (boosting and linear) lead to feasible metrics with close performances to the oracles ones: the corresponding estimators recover well-enough the true nuisances. One may wonder if simpler models for the nuisance could be useful, in particular in data-poor settings or when the true models are linear. Figure 6 compares causal model selection estimating nuisances with stacked estimators or linear model. It comprises the Twins data, where the true propensity model is linear, and a downsampled version of this data, to study a situation favorable to linear models. In these settings, stacked and linear estimations of the nuisances performs equivalently. Detailed analysis (Figure 20) confirms that using adaptive models –as built by stacking linear models and gradient-boosted trees– suffices to estimate nuisance.

R-risk is robust to a wide range of effect ratio values. Beyond overlap, we study for caussim simulations, the effect on model selection of different causal effect ratio to baseline. We vary the empirical mean absolute difference between the causal effect and the baseline, $\Delta_\mu = \frac{1}{N}\sum_{i=1}^{N} \left|\frac{\mu_1(x_i)-\mu_0(x_i)}{\mu_0(x_i)+\mu_1(x_i)-\frac{1}{N}\sum_{j=1}^{N}\mu_0(x_j)+\mu_1(x_j)}\right|$, covering a ratio

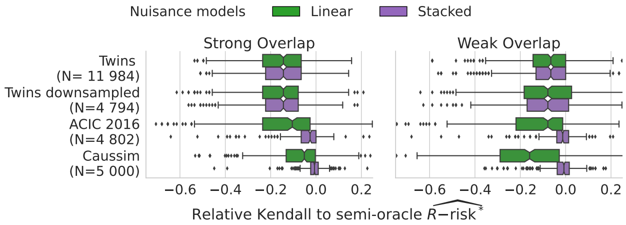

Figure 6. Stacked models are good overall estimators of the nuisances: Results are shown only for the R-risk; Figure 19 details every metrics. For Twins, where the true propensity model is linear, stacked and linear estimations of the nuisances performs equivalently, even for a downsampled version (N=4,794).

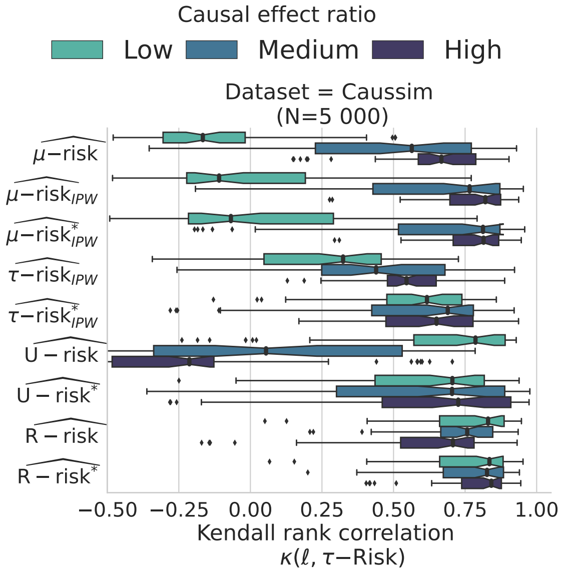

Figure 7. R-risk is robust to a wide range of effect ratio: Kendall's τ agreement with τ-risk. Strong, medium and Weak Causal effect ratio are the tertiles of the absolute ratio causal effect to baseline response, Δ_μ: Low [0.04; 2.86[, Medium [2.86; 16.65[, High [16.65; 206.53[. Appendix A.9 details this simulation.

range from 0.04 to 206 (median = 9.1). Appendix A.9 details this setup as well as an alternive measure of effect ratio. Figure 7 shows that for high values of the ratio, $R-$ risk is outperformed by the μ-risk$_{IPW}$ and the τ-risk$_{IPW}$. However, on average, the $R-$ risk is still the better risk.

Discussion and conclusion

Nuisance models: more gain than pain. Predictive models are increasingly used to reason about treatment effects, for instance in precision medicine to drive individualized decision. Our results highlight that they should be selected, validated, and tuned using different procedures and error measures than those classically used to assess prediction. Rather, selecting the best outcome model according to the R-risk (eq. Definition 5) leads to more valid causal estimates on average. Estimating the R-risk requires a more complex procedure than standard cross-validation used *e.g.*in machine learning: it involves fitting nuisance models necessary for model evaluation. Our results show that these can be learned on the same set of data as the outcome models evaluated. The nuisance models must be well estimated (Figure 6). Our results show that using for nuisance models a flexible stacking-based family of estimator suffices for good model selection. To select propensity score models, we used the Brier score, minimized by the true individual probability. An easy mistake is to use calibration errors popular in machine learning [73, 74, 75, 76] as these select not for the individual posterior

probability but for an aggregate error rate [77].

More R-risk to select models driving decisions. Increasingly complex prediction models integrating richer medical data have flourished because their predictions can be easily demonstrated and validated on left-out data. But using them to underpin a decision on whether to treat or not requires more careful validation, using a metric accounting for the putative intervention, the R-risk. On average, the R-risk brings a sizeable benefit to select the most adequate model, even when model development is based on treated and untreated population with little differences, as in RCTs. Our conclusions are that without prior knowledge, the R-risk is a good default. However, there is much remaining variation, and the R-risk will not be optimal for every situation. We have identified one such specific situation: when the causal effect is large compared to the variation of the baseline effect, the μ-risk$_{IPW}$ performs slightly better.

To facilitate better model selection, we provide Python code[4]. This model-selection procedure puts no constraints on the models used to build predictive models: it opens the door to evaluating a wide range of models, from gradient boosting to convolutional neural network, or language models.

Availability of source code and requirements

Lists the following:

- Project name: Caussim
- Project home page: https://github.com/soda-inria/caussim
- Operating system(s): Platform independent
- Programming language: Python
- License: BSD 3-Clause License

Declaration

Competing interests

No competing interest is declared.

Author contributions statement

M.D. conceived and conducted the experiments, M.D. and G.V. analyzed the results. M.D. and G.V. wrote and reviewed the manuscript.

Acknowledgments

We acknowledge fruitful discussions with Bénédicte Colnet.

4 https://github.com/soda-inria/causal_model_selection

A Additional files

A.1 Variability of ATE estimation on ACIC 2016

Figure 8 shows ATE estimations for six different models used in g-computation estimators on the 76 configurations of the ACIC 2016 dataset. Outcome models are fitted on half of the data and inference is done on the other half –ie. train/test with a split ratio of 0.5. For each configuration, and each model, this train test split was repeated ten times, yielding non parametric variance estimates [78]. Figure 8 shows large variations obtained across different outcome estimators on semi-synthetic datasets [45]. Flexible models such as random forests are doing well in most settings except when treated and untreated populations differ noticeably, in which case a linear model (ridge) is to be preferred. However random forests with different hyper-parameters (max depth= 2) yield poor estimates. A simple rule of thumb such as preferring flexible models does not work in general; model selection is needed.

Outcome models are implemented with scikit-learn [79] and the following hyper-parameters:

Outcome Model	Hyper-parameters grid
Random Forests	Max depth: [2, 10]
Ridge regression without treatment interaction	Ridge regularization: [0.1]
Ridge regression with treatment interaction	Ridge regularization: [0.1]

Table 2. Hyper-parameters grid used for ACIC 2016 ATE variability

A.2 Prior work : model selection for outcome modeling (g-computation)

A natural way to select a predictive model for causal inference would be an error measure between a causal quantity such as the CATE and models' estimate. But such error is not a "feasible" risk: it cannot be computed solely from observed data and requires oracle knowledge.

Simulation studies of causal model selection. Using eight simulations setups from [38], where the oracle CATE is known, [50] compare four causal risks, concluding that for CATE estimation the best model-selection risk is the so-called R-risk [42] –def. 5, below. Their empirical results are clear for randomized treatment allocation but less convincing for observational settings where both simple MSE –μ-risk(f) def. 1– and reweighted MSE –μ-risk$_{IPW}$ def. 2– appear to perform better than R-risk on half of the simulations. Another work [51] studied empirically both MSE and reweighted MSE risks on the semi-synthetic ACIC 2016 datasets [45], but did not include the R-risk. We complete these prior empirical work by studying a wider variety of data generative processes and varying the influence of overlap, an

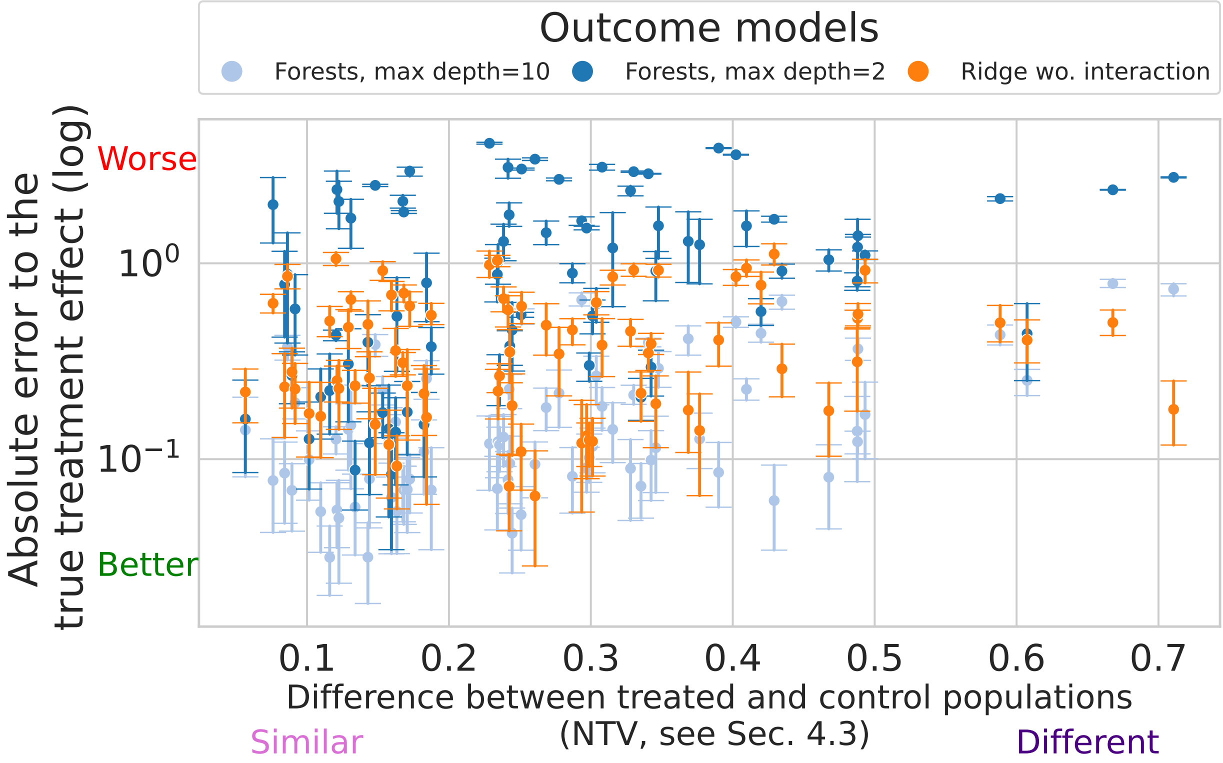

Figure 8. Different outcome models lead to different estimation errors on the Average Treatment Effects, on 77 classic simulations with known true causal effect [45]. The different models are ridge regression and random forests with different hyper-parameters (details A.1). The different configurations are plotted as a function of increasing difference between treated and untreated population –see subsection . There is no systematic best performer; data-driven model selection is important.

important parameter of the data generation process which makes a given causal metric appropriate [80]. We also study how to best adapt cross-validation procedures to causal metrics which themselves come with models to estimate.

Theoretical studies of causal model selection. Several theoretical works have proposed causal model selection procedures that are *consistent*: select the best model in a family given asymptotically large data. These work rely on introducing a CATE estimator in the testing procedure: matching [81], an IPW estimate [72], a doubly robust estimator [82], or debiasing the error with influence functions [51]. However, for theoretical guarantees to hold, the test-set correction needs to converge to the oracle: it needs to be flexible enough –well-posed– and asymptotic data. From a practical perspective, meeting such requirements implies having a good CATE estimate, thus having solved the original problem of causal model selection.

Statistical guarantees on causal estimation procedures. Much work in causal inference has focused on procedures that guarantee asymptotically consistent estimators, such as Targeted Machine Learning Estimation (TMLE) [36, 37] or Double Machine Learning [43]. Here also, theories require asymptotic regimes and models to be *well-specified*.

By contrast, without assuming that estimators are well specified, there exists an upper bound on the oracle error to the CATE (τ-risk) that involves the error on the outcome and the similarity of the distributions of treated and control patients [83]. However, they use this upper bound for model optimization, and do not give insights on model selection. In addition, for hyperparameter selection, they rely on a plugin estimate of the τ-risk built with counterfactual nearest neighbors, which has been shown ineffective [50]. An interesting direction is taken in [84] where the authors derive convergence rates for orthogonal losses such as the R-loss, or the DR-Loss without assuming well-specification of the model for target parameter.

A.3 Causal assumptions

We assume the following four assumptions, referred as strong ignorability and necessary to assure identifiability of the causal estimands with observational data [56]:

Assumption 1 (Consistency) *The observed outcome is the potential outcome of the assigned treatment:*

$$Y = A\,Y(1) + (1-A)\,Y(0)$$

Here, we assume that the intervention A has been well defined. This assumption focuses on the design of the experiment. It clearly states the link between the observed outcome and the potential outcomes through the intervention [24].

Assumption 2 (Unconfoundedness)

$$\{Y(0), Y(1)\} \perp\!\!\!\perp A|X$$

This condition –also called ignorability– is equivalent to the conditional independence on $e(X)$ [28]: $\{Y(0), Y(1)\} \perp\!\!\!\perp A|e(X)$.

Assumption 3 (Overlap, also known as Positivity))

$$\eta < e(x) < 1-\eta \quad \forall x \in \mathcal{X} \text{ and some } \eta > 0$$

The treatment is not perfectly predictable. Or with different words, every patient has a chance to be treated and not to be treated. For a given set of covariates, we need examples of both to recover the ATE.

As noted by [80], the choice of covariates X can be viewed as a trade-off between these two central assumptions. A bigger covariates set generally reinforces the ignorability assumption. In the contrary, overlap can be weakened by large $\mathcal{X}$ because of the potential inclusion of instruments: variables only linked to the treatment which could lead to arbitrarily small propensity scores.

Assumption 4 (Generalization) *The training data on which we build the estimator and the test data on which we make the estimation are drawn from the same distribution $\mathcal{D}^*$, also known as the "no covariate shift" assumption [85].*

A.4 Definitions of feasible risks

Definition 1 (Factual μ-risk) *[60] This is the usual Mean Squared Error on the target y. It is what is typically meant by "generalization error" in supervised learning:*

$$\mu\text{-}risk(f) = \mathbb{E}\left[(Y - f(X;A))^2\right]$$

Definition 2 (μ-risk$^{\star}_{IPW}$) *[58] Let the inverse propensity weighting function $w(x,a) = \frac{a}{e(x)} + \frac{1-a}{1-e(x)}$, we define the semi-oracle Inverse Propensity Weighting risk,*

$$\mu\text{-}risk^{\star}_{IPW}(f) = \mathbb{E}\left[\left(\frac{A}{e(X)} + \frac{1-A}{1-e(X)}\right)(Y - f(X;A))^2\right]$$

Definition 3 (τ-risk$^{\star}_{IPW}$) *[39] The CATE $\tau(x)$ can be estimated with a regression against inverse propensity weighted outcomes [71, 72, 39], the τ-risk$_{IPW}$.*

$$\tau\text{-risk}^{\star}_{IPW}(f) = \mathbb{E}\left[\left(Y\frac{A-e(X)}{e(X)(1-e(X))} - \tau_f(X)\right)^2\right]$$

Definition 4 (U-risk*) *[41, 42] Based on the Robinson decomposition –eq. 8, the U-learner uses the $A - e(X)$ term in the denominator. The derived risk is:*

$$U\text{-risk}^{\star}(f) = \mathbb{E}\left[\left(\frac{Y-m(X)}{A-e(X)} - \tau_f(X)\right)^2\right]$$

Note that extreme propensity weights in the denominator term might inflate errors in the numerator due to imperfect estimation of the mean outcome m.

Definition 5 (R-risk*) *[42, 50] The R-risk also uses two nuisance m and e:*

$$R\text{-risk}^{\star}(f) = \mathbb{E}[((Y-m(X)) - (A-e(X))\tau_f(X))^2]$$

It is also based on the Robinson decomposition –eq. 8.

A.5 Proofs: Links between feasible and oracle risks

Reformulation of the R-risk as reweighted τ-risk
Proposition 1 (R-risk as reweighted τ-risk) ***Proof 1*** *We consider the R-decomposition: [57],*

$$y(a) = m(x) + (a - e(x))\tau(x) + \varepsilon(x; a) \quad (12)$$

Where $\mathbb{E}[\varepsilon(X; A)|X, A] = 0$ We can use it as plug in the R-risk formula:

$$\begin{aligned}
R\text{-risk}(f) &= \int_{\mathcal{Y}\times\mathcal{X}\times\mathcal{A}} [(y-m(x)) - (a-e(x))\tau_f(x)]^2 p(y;x;a)dydxda \\
&= \int_{\mathcal{Y}\times\mathcal{X}\times\mathcal{A}} \left[(a-e(x))\tau(x) + \varepsilon(x;a) - (a-e(x))\tau_f(x)\right]^2 p(y;x;a)dydxda \\
&= \int_{\mathcal{X}\times\mathcal{A}} (a-e(x))^2(\tau(x)-\tau_f(x))^2 p(x;a)dxda \\
&+ 2\int_{\mathcal{Y}\times\mathcal{X}\times\mathcal{A}} (a-e(x))(\tau(x)-\tau_f(x)) \int_{\mathcal{Y}} \varepsilon(x;a)p(y \mid x;a)dyp(x;a)dxda \\
&+ \int_{\mathcal{X}\times\mathcal{A}} \int_{\mathcal{Y}} \varepsilon^2(x;a)p(y \mid x;a)dyp(x;a)dxda
\end{aligned}$$

The first term can be decomposed on control and treated populations to force e(x) to appear:

$$\begin{aligned}
&\int_{\mathcal{X}} (\tau(x)-\tau_f(x))^2 \left[e(x)^2 p(x;0) + (1-e(x))^2 p(x;1)\right] dx \\
&= \int_{\mathcal{X}} (\tau(x)-\tau_f(x))^2 \left[e(x)^2(1-e(x))p(x) + (1-e(x))^2 e(x)p(x)\right] dx \\
&= \int_{\mathcal{X}} (\tau(x)-\tau_f(x))^2 (1-e(x))e(x)[1-e(x)+e(x)]p(x)dx \\
&= \int_{\mathcal{X}} (\tau(x)-\tau_f(x))^2 (1-e(x))e(x)p(x)dx.
\end{aligned}$$

The second term is null since, $\mathbb{E}[\varepsilon(x, a)|X, A] = 0$.
The third term corresponds to the modulated residuals : $\tilde{\sigma}^2_B(0) + \tilde{\sigma}^2_B(1)$

Interesting special cases
Randomization special case. If the treatment is randomized as in RCTs, $p(A = 1 \mid X = x) = p(A = 1) = p_A$, thus μ-risk$_{IPW}$ takes a simpler form:

$$\mu\text{-risk}_{IPW} = \mathbb{E}_{(Y,X,A)\sim\mathcal{D}}\left[\left(\frac{A}{p_A} + \frac{1-A}{1-p_A}\right)(Y - f(X;A))^2\right]$$

However, we still can have large differences between τ-risk and μ-risk$_{IPW}$ coming from heterogeneous errors between populations as shown experimentally in [50] and our results below.

Concerning the R-risk, replacing $e(x)$ by its randomized value p_A in Proposition 1 yields the oracle τ-risk up to multiplicative and additive constants:

$$R\text{-risk} = p_A(1-p_A)\,\tau\text{-risk} + (1-p_A)\,\sigma^2_B(0) + p_A\,\sigma^2_B(1)$$

Thus, selecting estimators with R-risk* in randomized setting controls the τ-risk. This explains the strong performances of R-risk in randomized setups [50] and is a strong argument to use it to estimate heterogeneity in RCTs.

Oracle Bayes predictor. If we have access to the oracle Bayes predictor for the outcome ie. $f(x, a) = \mu(x, a)$, then all risks are equivalent up to the residual variance:

$$\tau\text{-risk}(\mu) = \mathbb{E}_{X\sim p(X)}[(\tau(X) - \tau_\mu(X))^2] = 0 \tag{13}$$

$$\begin{aligned}\mu\text{-risk}(\mu) &= \mathbb{E}_{(Y,X,A)\sim p(Y;X;A)}[(Y - \mu_A(X))^2] \\ &= \int_{\mathcal{X},\mathcal{A}} \varepsilon(x, a)^2 p(a \mid x)\, p(x)\, dx\, da \leq \sigma_B^2(0) + \sigma_B^2(1)\end{aligned} \tag{14}$$

$$\mu\text{-risk}_{IPW}(\mu) = \sigma_B^2(0) + \sigma_B^2(1) \quad \text{from Lemma ??} \tag{15}$$

$$R\text{-risk}(\mu) = \tilde{\sigma}_B^2(0) + \tilde{\sigma}_B^2(1) \leq \sigma_B^2(0) + \sigma_B^2(1) \qquad \text{from Proposition 1} \tag{16}$$

Thus, differences between causal risks only matter in finite sample regimes. Universally consistent learners converge to the Bayes risk in asymptotic regimes, making all model selection risks equivalent. In practice however, choices must be made in non-asymptotic regimes.

A.6 Measuring overlap

Motivation of the Normalized Total Variation. Overlap is often assessed by comparing visually population distributions as in Figure 1 or computing standardized difference on each feature [86, 29]. While these methods are useful to decide if positivity holds, they do not yield a single measure. Rather, we compute the divergence between the population covariate distributions $\mathbb{P}(X|A = 0)$ and $\mathbb{P}(X|A = 1)$ [80, 83]. Computing overlap when working only on samples of the observed distribution, outside of simulation, requires a sophisticated estimator of discrepancy between distributions, as two data points never have the same exact set of features. Maximum Mean Discrepancy [87] is typically used in the context of causal inference [60, 83]. However it needs a kernel, typically Gaussian, to extrapolate across neighboring observations. We prefer avoiding the need to specify such a kernel, as it must be adapted to the data which is tricky with categorical or non-Gaussian features, a common situation for medical data.

For simulated and some semi-simulated data, we have access to the probability of treatment for each data point, which sample both densities in the same data point. Thus, we can directly use distribution discrepancy measures and rely on the Normalized Total Variation (NTV) distance to measure the overlap between the treated and control propensities. This is the empirical measure of the total variation distance [88] between the distributions, $TV(\mathbb{P}(X|A = 1), \mathbb{P}(X|A = 0))$. As we have both distribution sampled on the same points, we can rewrite it a sole function of the propensity score, a low dimensional score more tractable than the full distribution $\mathbb{P}(X|A)$:

$$\widehat{NTV}(e, 1 - e) = \frac{1}{2N} \sum_{i=1}^{N} \Big| \frac{e(x_i)}{p_A} - \frac{1 - e(x_i)}{1 - p_A} \Big| \tag{17}$$

Formally, we can rewrite NTV as the Total Variation distance between the two population distributions. For a population $O = (Y(A), X, A) \sim \mathcal{D}$:

$$\begin{aligned} NTV(O) &= \frac{1}{2N} \sum_{i=1}^{N} \Big| \frac{e(x_i)}{p_A} - \frac{1 - e(x_i)}{1 - p_A} \Big| \\ &= \frac{1}{2N} \sum_{i=1}^{N} \Big| \frac{P(A = 1|X = x_i)}{p_A} - \frac{P(A = 0|X = x_i)}{1 - p_A} \Big| \end{aligned}$$

Thus NTV approximates the following quantity in expectation over the data distribution $\mathcal{D}$:

$$\begin{aligned} NTV(\mathcal{D}) &= \int_{\mathcal{X}} \Big| \frac{p(A = 1|X = x)}{p_A} - \frac{p(A = 0|X = x)}{1 - p_A} \Big| p(x) dx \\ &= \int_{\mathcal{X}} \Big| \frac{p(A = 1, X = x)}{p_A} - \frac{p(A = 0, X = x)}{1 - p_A} \Big| dx \\ &= \int_{\mathcal{X}} |p(X = x|A = 1) - p(X = x|A = 0)| dx \end{aligned}$$

For countable sets, this expression corresponds to the Total Variation distance between treated and control populations covariate distributions : $TV(p_0(x), p_1(x))$.

Measuring overlap without the oracle propensity scores:. For ACIC 2018, or for non-simulated data, the true propensity scores are not known. To measure overlap, we rely on flexible estimations of the Normalized Total Variation, using gradient boosting trees to approximate the propensity score. Empirical arguments for this plug-in approach is given in Figure 9.

Empirical arguments. We show empirically that NTV is an appropriate measure of overlap by :

- Comparing the NTV distance with the MMD for Caussim which is gaussian distributed in Figure 11,
- Verifying that setups with penalized overlap from ACIC 2016 have a higher total variation distance than unpenalized setups in Figure 10.
- Verifying that the Inverse Propensity Weights extrema (the inverse of the ν overlap constant appearing in the overlap Assumption 3) positevely correlates with NTV for Caussim, ACIC 2016 and Twins in Figure 12. Even if the same value of the maximum IPW could lead to different values of NTV, we expect both measures to be correlated : the higher the extrem propensity weights, the higher the NTV.

Estimating NTV in practice. Finally, we verify that approximating the NTV distance with a learned plug-in estimates of e(x) is reasonnable. We used either a logistic regression or a gradient boosting classifier to learn the propensity models for the three datasets where we have access to the ground truth propensity scores: Caussim, Twins and ACIC 2016. We respectively sampled 1000, 1000 and 770 instances of these datasets with different seeds and overlap settings. We first run a hyperparameter search with cross-validation on the train set, then select the best estimator. We refit on the train set this estimator with or without calibration by cross validation and finally estimate the normalized TV with the obtained model. This training procedure reflects the one described in Algorithm 1 where nuisance models are fitted only on the train set.

The hyper parameters are : learning rate $\in [1e-3, 1e-2, 1e-1, 1]$, minimum samples leaf $\in [2, 10, 50, 100, 200]$ for boosting and L2 regularization $\in [1e-3, 1e-2, 1e-1, 1]$ for logistic regression.

Results in Figure 9 comparing bias to the true normalized Total Variation of each dataset instances versus growing true NTV indicate that calibration of the propensity model is crucial to recover a good approximation of the NTV.

A.7 Experiments

Details on the data generation process

We use Gaussian-distributed covariates and random basis expansion based on Radial Basis Function kernels. A random basis of RBF kernel enables modeling non-linear and complex relationships between covariates in a similar way to the well known spline expansion. The estimators of the response function are learned with a linear model on another random basis (which can be seen as a stochastic approximation of the full data kernel [63]). We carefully control the amount of overlap between treated and control populations, a crucial assumption for causal inference. Algorithm 2 describes the generation process for one simulation. Figure 13 illustrates 2D examples of the simulation.

- The raw features for both populations are drawn from a mixture of Gaussians: $\mathbb{P}(X) = p_A \mathbb{P}(X|A=1) + (1-p_A)\mathbb{P}(X|A=0)$ where $\mathbb{P}(x|A=a)$ is a rotated Gaussian:

$$\mathbb{P}(x|A=a) = W \cdot \mathcal{N}\Big(\begin{bmatrix} (1-2a)\theta \\ 0 \end{bmatrix} ; \begin{bmatrix} \sigma_0 & 0 \\ 0 & \sigma_1 \end{bmatrix} \Big) \tag{18}$$

  with θ a parameter controlling overlap (bigger yields poorer overlap), W a random rotation matrix and $\sigma_0^2 = 2; \sigma_1^2 = 5$.
  This generation process allows to analytically compute the oracle propensity scores $e(x)$, to simply control for overlap with the parameter θ, the distance between the two Gaussian main axes and to visualize response surfaces.
- A basis expansion of the raw features increases the problem dimension. Using Radial Basis Function (RBF) Nystroem transformation [5], we expand the raw features into a transformed space. The basis expansion samples randomly a small number of representers in the raw data. Then, it computes an approximation of the full N-dimensional kernel with these basis components, yielding the transformed features $z(x)$. The number of basis functions –*ie. knots*–, controls the complexity of the ground-truth response surfaces and treatment. We first use this process to draw the non-treated response surface μ_0 and the causal effect τ. We then draw the observations from a mixture two Gaussians, for the treated and non treated. We vary the separation between the two Gaussians to control the overlap between treated and non-treated populations, an important parameter for causal inference (related to η in Assumption 3). Finally, we generate observed outcomes adding Gaussian noise.
  More formally, we generate the basis following the original data distribution, $[b_1..b_D] \sim \mathbb{P}(x)$, with D=2 in our simulations. Then, we compute an approximation of the full kernel of the data generation process with these representers: $z(x) = [RBF_\gamma(x, b_d)]_{d=1..D} \cdot Z^T \in \mathbb{R}^D$ with RBF_γ being the Gaussian kernel $K(x,y) = exp(-\gamma||x-y||^2)$ and Z the normalization constant of the kernel basis, computed as the root inverse of the basis kernel $Z = [K(b_i, b_j)]_{i,j\in 1..D}^{-1/2}$
- Functions μ_0, τ are distinct linear functions of the transformed features:

$$\mu_0(x) = \big[z(x); 1\big] \cdot \beta_\mu^T$$

$$\tau(x) = \big[z(x); 1\big] \cdot \beta_\tau^T$$

  Where β_μ and β_τ are sampled from two normal distributions with mean 0 and unit variances $\mathcal{N}(0, I_{D+1})$.

5 We use the Sklearn implementation, [79]

(a) Uncalibrated classifiers

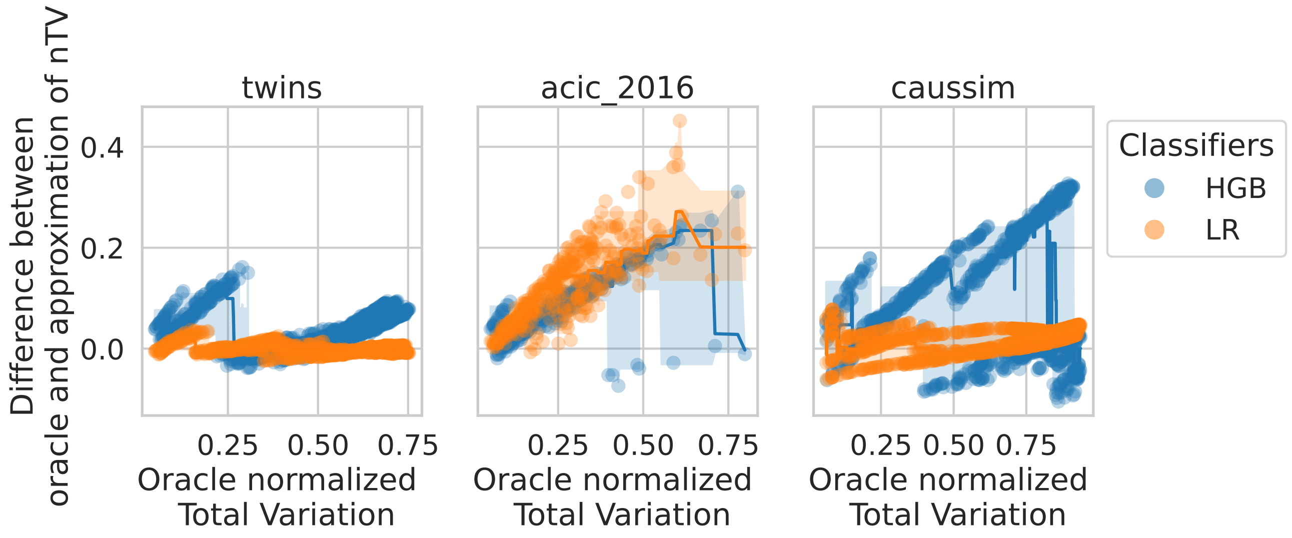

(b) Calibrated classifiers

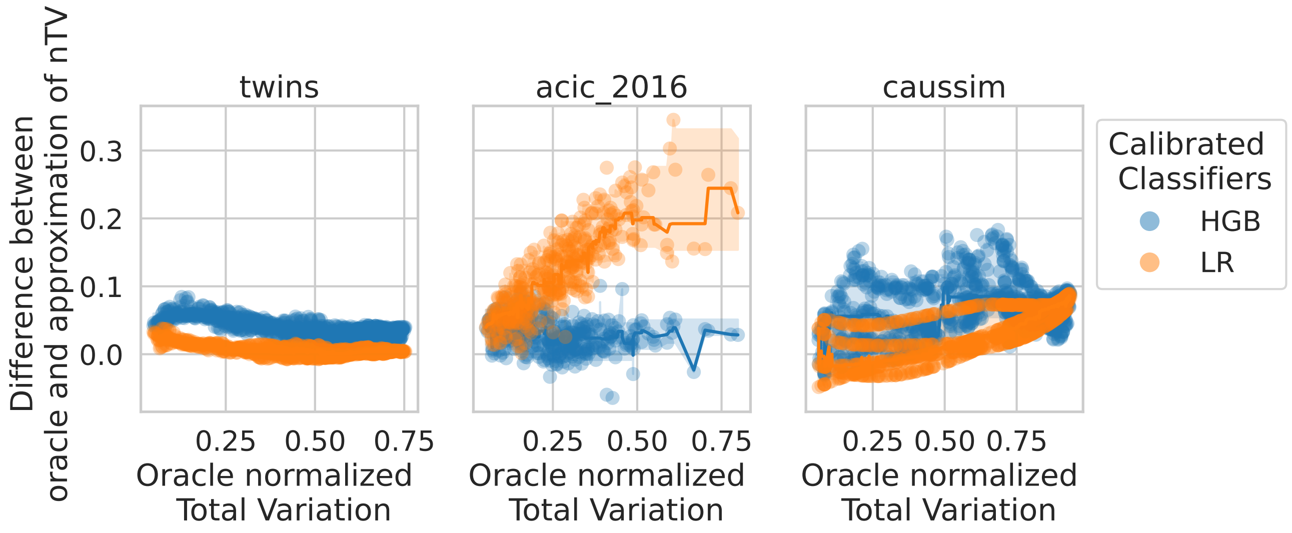

Figure 9. a) Without calibration, estimation of NTV is not trivial even for boosting models. b) Calibrated classifiers are able to recover the true Normalized Total Variation for all datasets where it is available.

Figure 10. NTV recovers well the overlap settings described in the ACIC paper [45]

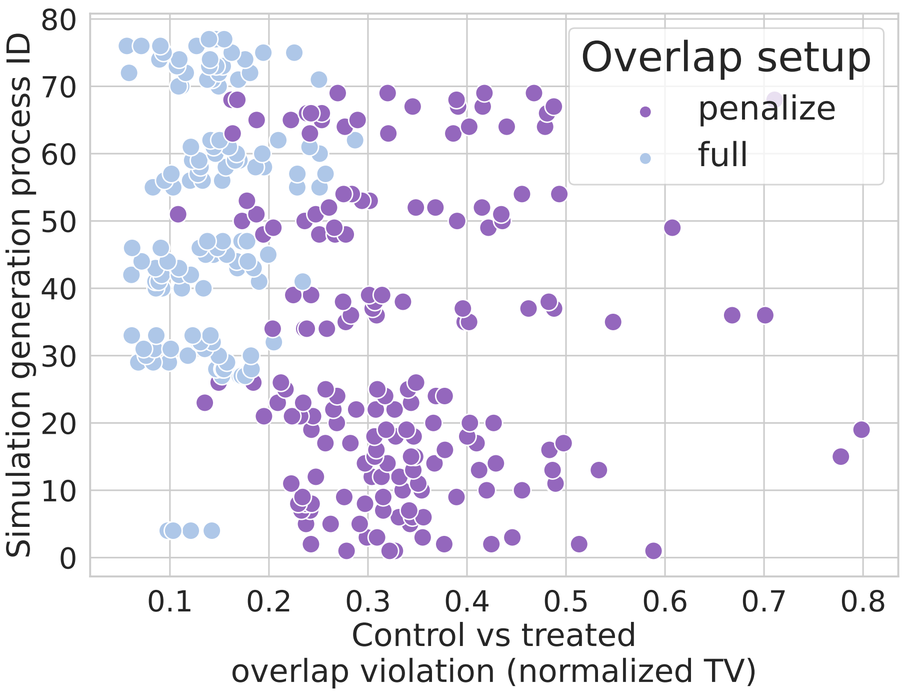

Figure 11. Good correlation between overlap measured as normalized Total Variation and Maximum Mean Discrepancy (200 sampled Caussim datasets)

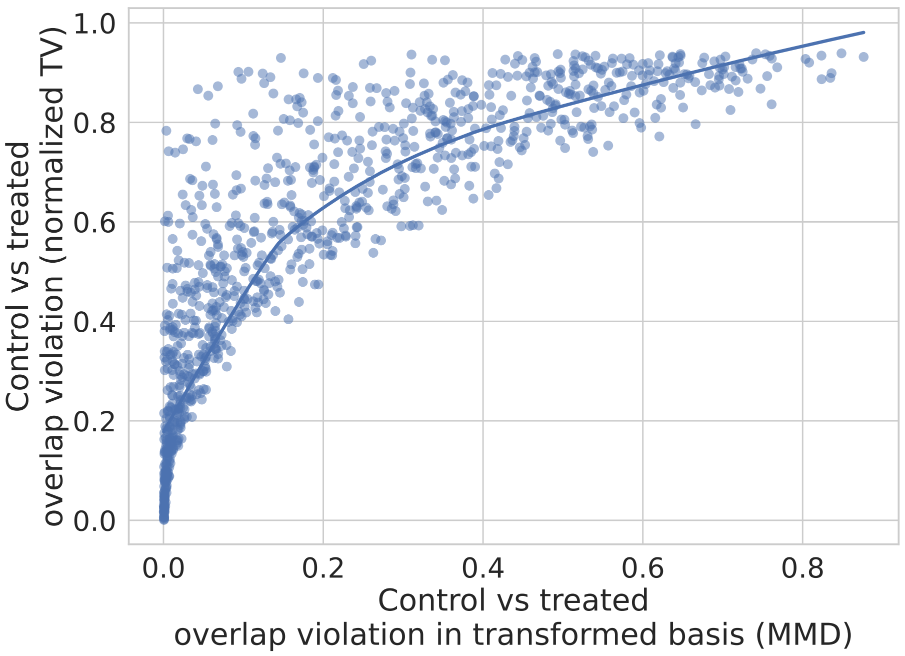

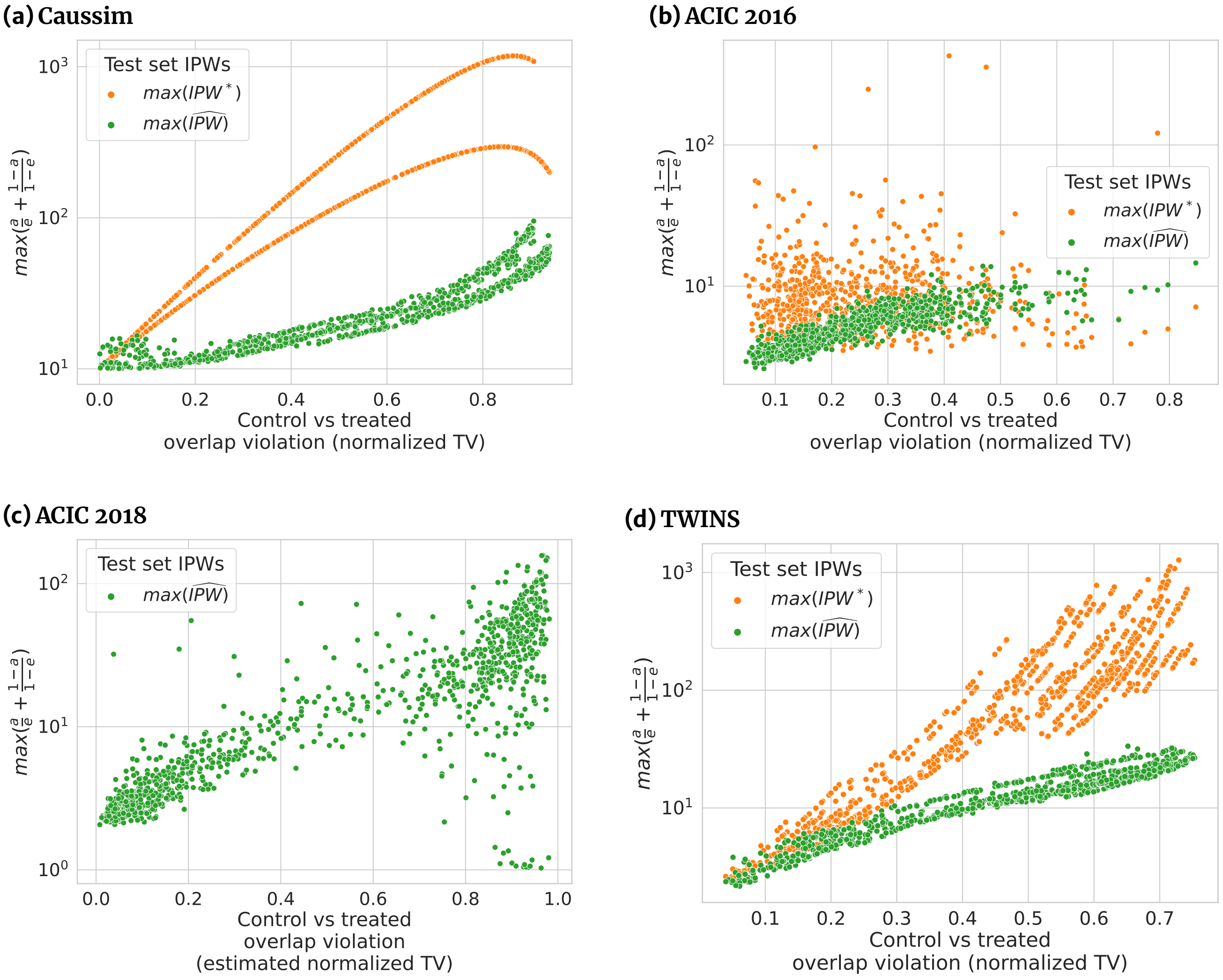

Figure 12. Maximal value of Inverse Propensity Weights increases exponentially with the overlap as measure by Normalized Total Variation.

- Adding a Gaussian noise, $\varepsilon \sim \mathcal{N}(0, \sigma(x; a))$, we construct the potential outcomes: $y(a) = (1 - \omega)\mu_0(x) + a\,\omega\tau(x) + \varepsilon(x, a)$ with ω the treatment effect relative size with respect to the baseline response.

We generated 1000 instances of this dataset with uniformly random overlap parameters $\theta \in [0, 2.5]$.

Details on the semi-simulated datasets

ACIC 2018 [45]: The initial intervention was a child's birth weight ($A = 1$ if weight $< 2.5kg$), and outcome was the child's IQ after a follow-up period. The study contained $N = 4\,802$ data points with $D = 55$ features (5 binary, 27 count data, and 23 continuous). They simulated 77 different setups varying parameters for treatment and response models, overlap, and interactions between treatment and covariates [6]. We used 10 different seeds for each setup, totaling 770 dataset instances.

ACIC 2018 [66]: Starting from data from the Linked Births and Infant Deaths Database (LBIDD) [67] with $D = 177$ covariates, treatment and outcome models are simulated with complex models to reflect different scenarii. The data do not provide the true propensity scores, so we evaluate only feasible metrics, which do not require this nuisance parameter. We used all 432 datasets[7] of size $N = 5\,000$.

Twins [68]: It is an augmentation of real data on twin births and mortality rates [69]. There are $N = 11\,984$ samples (pairs of twins), and $D = 50$ covariates[8], The outcome is the mortality and the treatment is the weight of the heavier twin at birth. This is a "true" counterfactual dataset [89] in the sense that we have both potential outcomes with each twin. They simulate the treatment with a sigmoid model based on GESTAT10 (number of gestation weeks before birth) and x the 45 other covariates:

$$\mathbf{t}_i \mid \mathbf{x}_i, \mathbf{z}_i \sim \text{Bern}\left(\sigma\left(w_o^\top \mathbf{x} + w_h(\mathbf{z}/10 - 0.1)\right)\right) \quad (19)$$
$$\text{with } w_o \sim \mathcal{N}(0, 0.1 \cdot I),\ w_h \sim \mathcal{N}(5, 0.1)$$

6 Original R code available at `https://github.com/vdorie/aciccomp/tree/master/2016` to generate 77 simulations settings.
7 Using the scaling part of the data, from github.com/IBM-HRL-MLHLS/IBM-Causal-Inference-Benchmarking-Framework
8 We obtained the dataset from https://github.com/AMLab-Amsterdam/CEVAE/tree/master/datasets/TWINS

Algorithm 2 Data simulation for the simulated dataset caussim

Let the parameters of a given simulation be:

- N the number of samples.
- D the dimension of the basis expansion.
- p_A the proportion of treated individuals.
- θ the overlap parameter.
- $\mathbb{P}(x|A=a) = W \cdot \mathcal{N}\left(\begin{bmatrix}(1-2a)\theta \\ 0\end{bmatrix}; \begin{bmatrix}\sigma_0 & 0 \\ 0 & \sigma_1\end{bmatrix}\right)$ the rotated gaussian for each population covariate. W is a random rotation matrix and $\sigma_0^2 = 2; \sigma_1^2 = 5$ are scaling parameters.
- $e(x) = \mathbb{P}(A=1|X=x)$ the oracle propensity score, obtained analytically from $\mathbb{P}(x|A=a)$.
- $\mathbb{P}(X) = p_A \mathbb{P}(X|A=1) + (1-p_A)\mathbb{P}(X|A=0)$ the mixture of gaussian for the whole population covariates.
- $b_1, .., b_D \sim \mathbb{P}(X)$, the basis sampled from the gaussian mixture.
- $z(x) = [RBF_\gamma(x, b_d)]_{d=1..D} \cdot Z^T \in \mathbb{R}^D$ the Nystroem expansion. RBF_γ is the Gaussian kernel $K(x,y) = exp(-\gamma||x-y||^2)$ and Z is the normalization constant of the kernel basis, computed as the root inverse of the basis kernel $Z = [K(b_i, b_j)]^{-1/2}_{i,j \in 1..D}$.
- β_μ and β_τ, the linear coefficients on top of the basis expansion. They are sampled from two normal distributions with mean 0 and unit variances $\mathcal{N}(0, I_{D+1})$.
- ω the treatment effect relative size with respect to the baseline response.
- $\varepsilon(x,a) \sim \mathcal{N}(0, \sigma_y)$ an exogen gaussian noise with σ_y the scale of the noise.

Generation process for one sample $(x, a, \mathrm{e}(x), y(0), y(1), y)$:

$x \sim \mathbb{P}(X)$ ▷ Sample from rotated gaussian mixture
$z(x)$ ▷ Compute the Nystroem expansion
$\mu_0(x) = \left[z(x); 1\right] \cdot \beta_\mu^T$ ▷ Compute the non-treated response surface, ie. the baseline
$\tau(x) = \left[z(x); 1\right] \cdot \beta_\tau^T$ ▷ Compute the conditional treatment effect (CATE)
$y(a) = (1-\omega)\mu_0(x) + a\,\omega\tau(x) + \varepsilon(x,a) + \varepsilon(x,a)$ ▷ Compute the potential outcomes
$a \sim \mathrm{Bernoulli}(e(x))$ ▷ Compute the treatment status
$y = a\,y(1) + (1-a)\,y(0)$ ▷ Compute the observed outcome

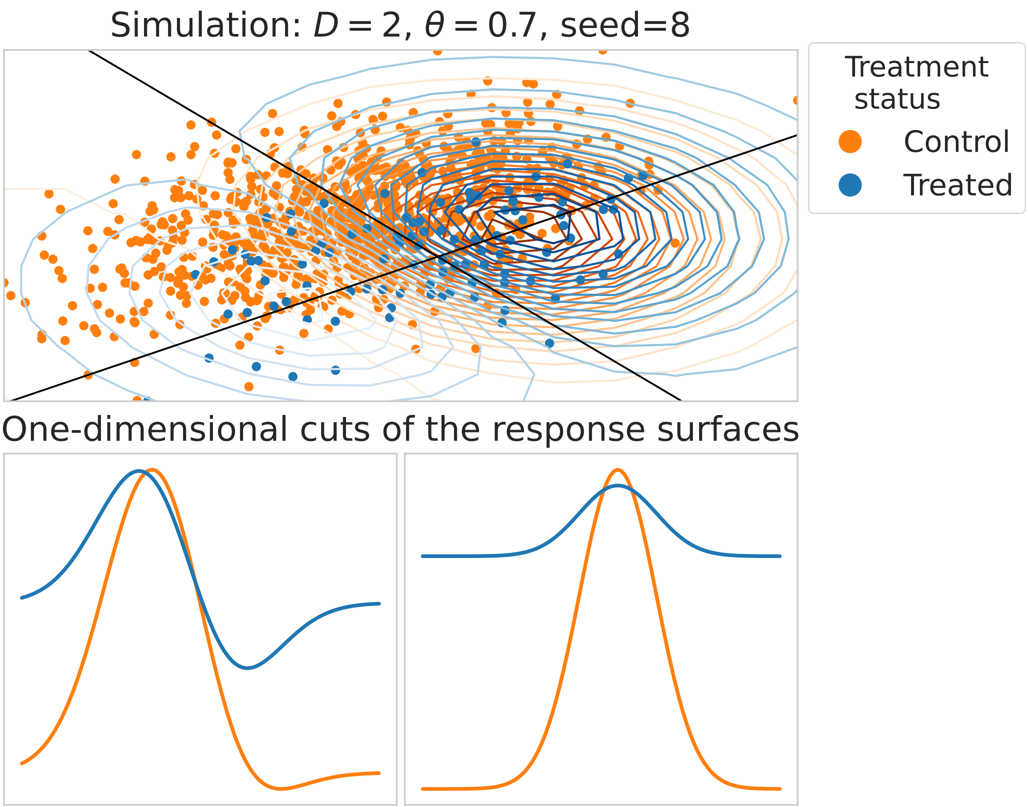

Figure 13. Example of the simulation setup in the input space with two knots –*ie.* basis functions. The top panel shows the observations in feature space, while the bottom panel displays the two response surfaces on a 1D cut along the black lines drawn on the top panel.

We add a non-constant slope in the sigmoid to control the overlap between treated and control populations. We sampled uniformly 1 000 different overlap parameters between 0 and 2.5, totaling 1 000 dataset instances. Unlike the previous datasets, only the overlap varies for these instances. The response surfaces are set by the original outcomes.

Model selection procedures

Nuisances estimation. The nuisances are estimated with a stacked regressor inspired by the Super Learner framework, [90]). We select hyper-parameters with randomized search on a validation set $\mathcal{V}$ and keep them fix for model selection The searc grid is detailed in Table 3. All implementations come from scikit-learn [79]. As extreme inverse propensity weights induce high variance, we use clipping [91, 92] to bound $min(\check{e}, 1-\check{e})$ away from 0 with a fixed $\eta = 10^{-10}$, ensuring strict overlap for numerical stability.

Model	Estimator	Hyper-parameters grid
Outcome, m	StackedRegressor (HistGradientBoostingRegressor, ridge)	ridge regularization: [0.0001, 0.001, 0.01, 0.1, 1, 10, 100]<br>HistGradientBoostingRegressor learning rate: [0.01, 0.1, 1]<br>HistGradientBoostingRegressor max leaf nodes: [10, 20, 30, 50]
Treatment, e	StackedClassifier (HistgradientBoostingClassifier, LogisticRegression)	LogisticRegression C: [0.0001, 0.001, 0.01, 0.1, 1, 10, 100]<br>HistGradientBoostingClassifier learning rate: [0.01, 0.1, 1]<br>HistGradientBoostingClassifier max leaf nodes: [10, 20, 30, 50]

Table 3. Hyper-parameters grid used for nuisance models

Additional Results

Definition of the Kendall's tau, κ. The Kendall's tau is a widely used statistics to measure the rank correlation between two set of observations. It measures the number of concordant pairs minus the discordant pairs normalized by the total number of pairs. It takes values in the $[-1, 1]$ range.

$$\kappa = \frac{\text{(number of concordant pairs)} - \text{(number of discordant pairs)}}{\text{(number of pairs)}} \tag{20}$$

Values of relative $\kappa(\ell, \tau\text{–risk})$ compared to the mean over all metrics Kendall's as shown in the boxplots of Figure 3.

Figure 14 - Results measured in relative Kendall's for feasible and semi-oracle risks. Because of extreme propensity scores in the denominator and bayes error residuals in the numerator, the semi-oracle U-risk has poor performances at bad overlap. Estimating these propensity scores in the is feasible U-risk reduces the variance since clipping is performed.

Figure 15 - Results measured in absolute Kendall's.

Figure 16 - Results measured as distance to the oracle tau-risk. To see practical gain in term of τ-risk, we plot the results as the normalized distance between the estimator selected by the oracle τ-risk and the estimator selected by each causal metric.

Then, $\widehat{R\text{-risk}}^*$ is more efficient than all other metrics. The gain are substantial for every datasets.

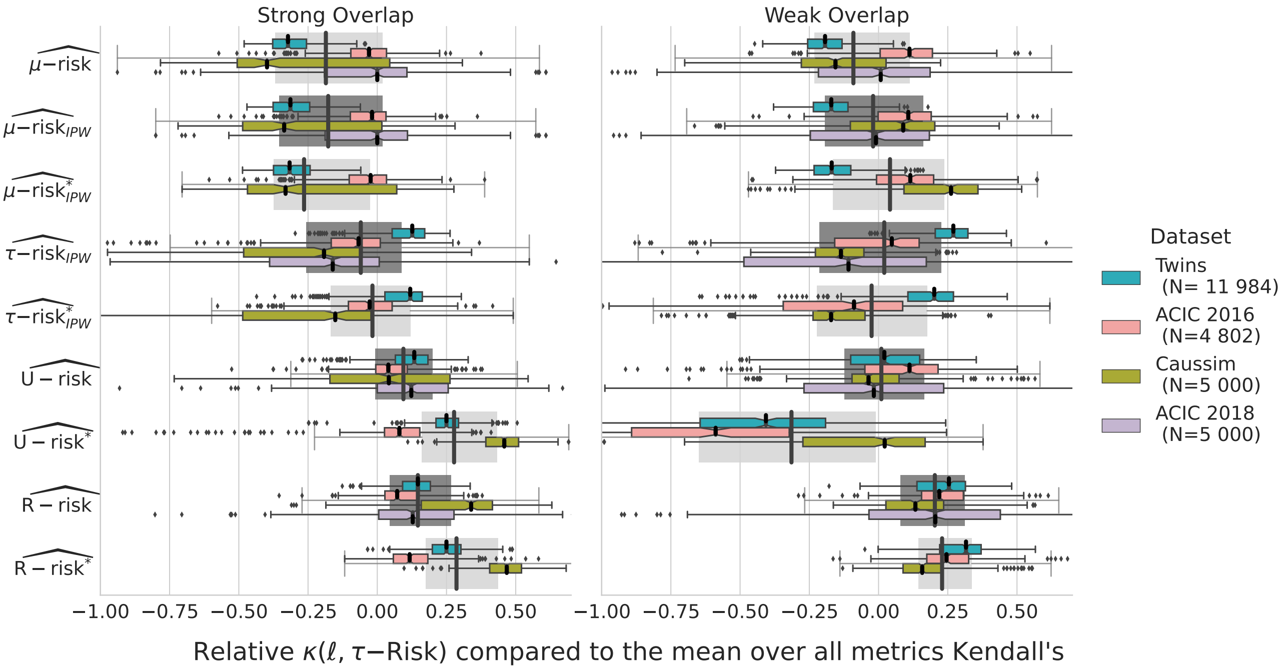

Figure 14. The R-risk is the best metric on average: Relative Kendall's τ agreement with τ-risk. Strong and Weak overlap correspond to the first and last tertiles of the overlap distribution measured with Normalized Total Variation eq. 17.

Metric	Dataset	Strong Overlap Median	Strong Overlap IQR	Weak Overlap Median	Weak Overlap IQR
$\widehat{\mu-\text{risk}}$	Twins (N=11 984)	-0.32	0.12	-0.19	0.12
	ACIC 2016 (N=4 802)	-0.03	0.13	0.11	0.19
	Caussim (N=5 000)	-0.40	0.55	-0.16	0.31
	ACIC 2018 (N=5 000)	0.00	0.30	0.01	0.40
$\widehat{\mu-\text{risk}}_{IPW}$	Twins (N= 11 984)	-0.31	0.13	-0.17	0.12
	ACIC 2016 (N=4 802)	-0.02	0.13	0.11	0.19
	Caussim (N=5 000)	-0.34	0.50	0.09	0.31
	ACIC 2018 (N=5 000)	0.00	0.30	-0.01	0.43
$\widehat{\mu-\text{risk}}^{*}_{IPW}$	Twins (N= 11 984)	-0.32	0.13	-0.17	0.13
	ACIC 2016 (N=4 802)	-0.02	0.13	0.11	0.21
	Caussim (N=5 000)	-0.33	0.54	0.26	0.27
$\widehat{\tau-\text{risk}}_{IPW}$	Twins (N= 11 984)	0.13	0.12	0.27	0.12
	ACIC 2016 (N=4 802)	-0.07	0.18	0.05	0.31
	Caussim (N=5 000)	-0.19	0.43	-0.14	0.18
	ACIC 2018 (N=5 000)	-0.16	0.40	-0.11	0.66
$\widehat{\tau-\text{risk}}^{*}_{IPW}$	Twins (N= 11 984)	0.12	0.14	0.20	0.16
	ACIC 2016 (N=4 802)	-0.03	0.16	-0.09	0.43
	Caussim (N=5 000)	-0.15	0.46	-0.17	0.19
$\widehat{U-\text{risk}}$	Twins (N= 11 984)	0.13	0.12	0.02	0.25
	ACIC 2016 (N=4 802)	0.04	0.11	0.11	0.26
	Caussim (N=5 000)	0.04	0.43	-0.04	0.17
	ACIC 2018 (N=5 000)	0.12	0.26	-0.02	0.50
$\widehat{U-\text{risk}}^{*}$	Twins (N= 11 984)	0.25	0.08	-0.41	0.45
	ACIC 2016 (N=4 802)	0.08	0.13	-0.59	0.57
	Caussim (N=5 000)	0.46	0.12	0.02	0.44
$\widehat{R-\text{risk}}$	Twins (N= 11 984)	0.15	0.10	0.25	0.18
	ACIC 2016 (N=4 802)	0.07	0.12	0.22	0.15
	Caussim (N=5 000)	0.34	0.26	0.13	0.21
	ACIC 2018 (N=5 000)	0.13	0.27	0.21	0.47
$\widehat{R-\text{risk}}^{*}$	Twins (N= 11 984)	0.25	0.10	0.32	0.15
	ACIC 2016 (N=4 802)	0.12	0.12	0.25	0.15
	Caussim (N=5 000)	0.47	0.11	0.16	0.14

Table 4. Values of relative $\kappa(\ell, \tau-\text{risk})$ compared to the mean over all metrics Kendall's as shown in the boxplots of Figure 3

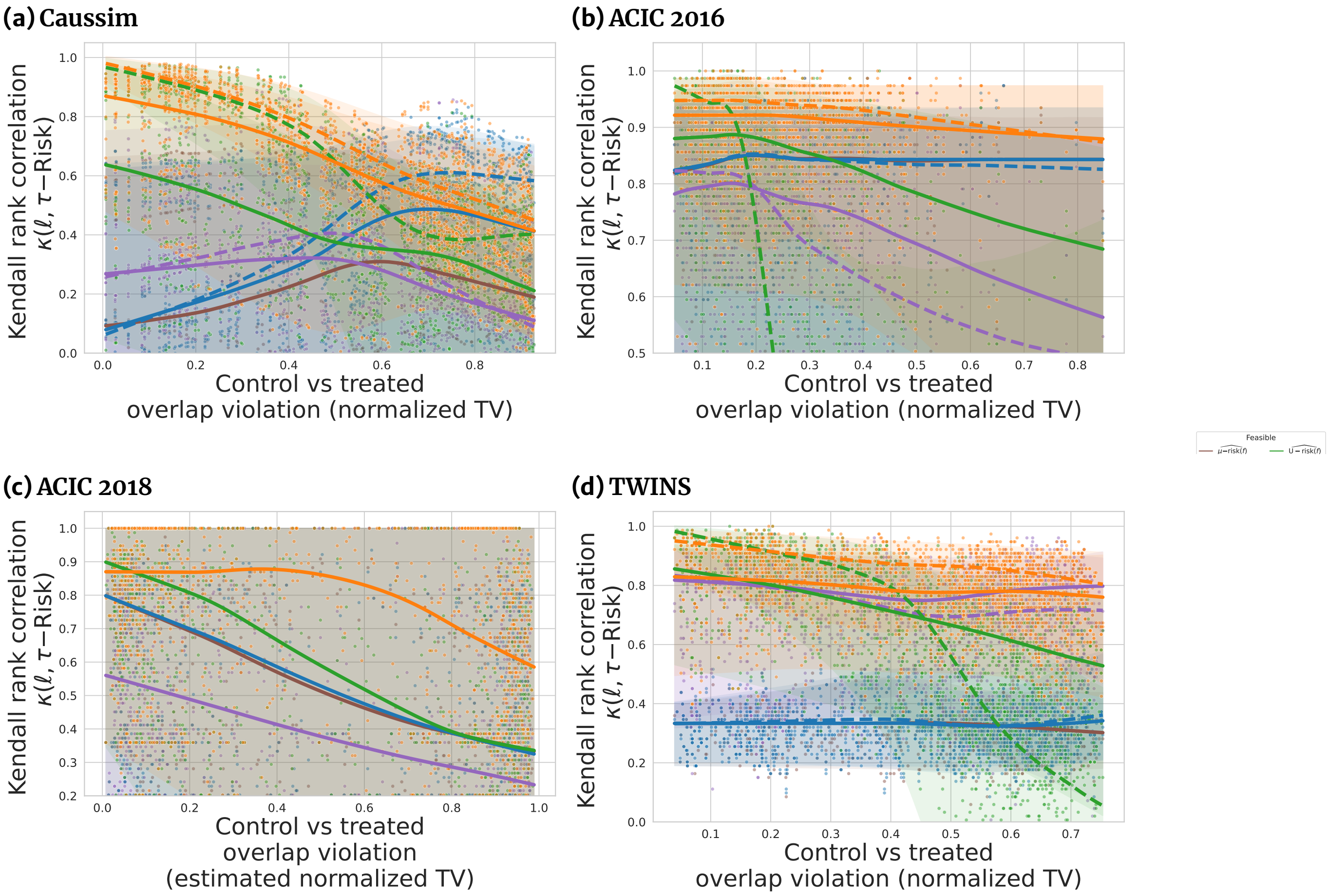

Figure 15. Agreement with τ-risk ranking of methods function of overlap violation. The lines represent medians, estimated with a lowess. The transparent bands denote the 5% and 95% confidence intervals.

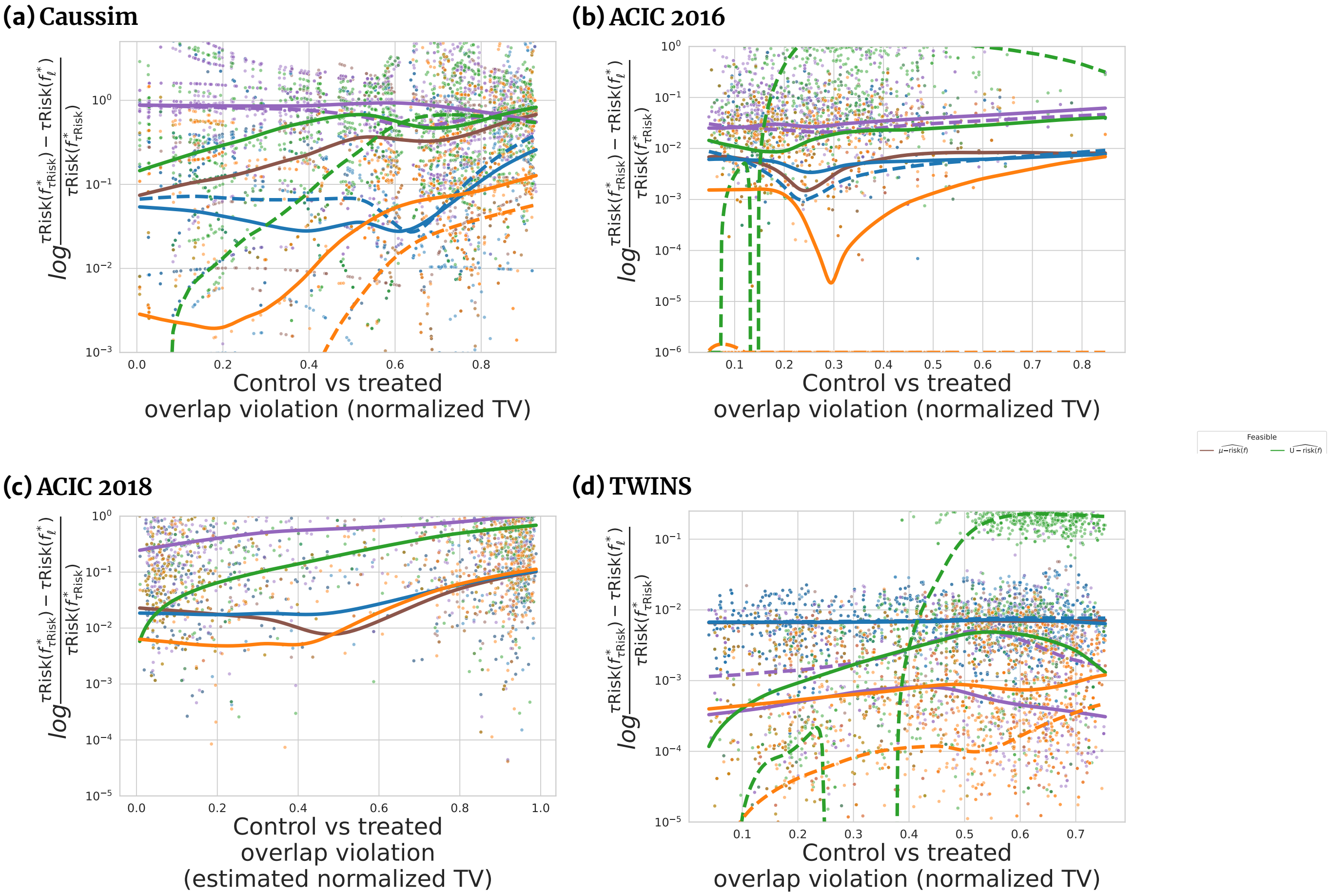

Figure 16. Metric performances by normalized tau-risk distance to the best method selected with τ-risk. All nuisances are learned with the same estimator stacking gradient boosting and ridge regression. Doted and plain lines corresponds to 60% lowess quantile estimates. This choice of quantile allows to see better the oracle metrics lines for which outliers with a value of 0 distord the curves.

Figure 17 - Stacked models for the nuisances is more efficient. For each metrics the benefit of using a stacked model of linear and boosting estimators for nuisances compared to a linear model. The evaluation measure is Kendall's tau relative to the oracle R-risk* to have a stable reference between exepriments. Thus, we do not include in this analysis the ACIC 2018 dataset since R-risk* is not available due to the lack of the true propensity score.

Figure 18 Low population overlap hinders model selection for all metrics.

Figure 19 - Stacked models for the nuisances is more efficient. For each metrics the benefit of using a stacked model of linear and boosting estimators for nuisances compared to a linear model. The evaluation measure is Kendall's tau relative to the oracle R-risk* to have a stable reference between exepriments. Thus, we do not include in this analysis the ACIC 2018 dataset since R-risk* is not available due to the lack of the true propensity score.

Figure 20 - Flexible models are performant in recovering nuisances even in linear setups.

Selecting different seeds and parameters is crucial to draw conclusions. One strength of our study is the various number of different simulated and semi-simulated datasets. We are convinced that the usual practice of using only a small number of generation processes does not allow to draw statistically significant conclusions.

Figure 21 illustrate the dependence of the results on the generation process for caussim simulations. We highlighted the different trajectories induced by three different seeds for data generation and three different treatment ratio instead of 1000 different seeds. The result curves are relatively stable from one setup to another for $R-risk$, but vary strongly for μ-risk and μ-risk$_{IPW}$.

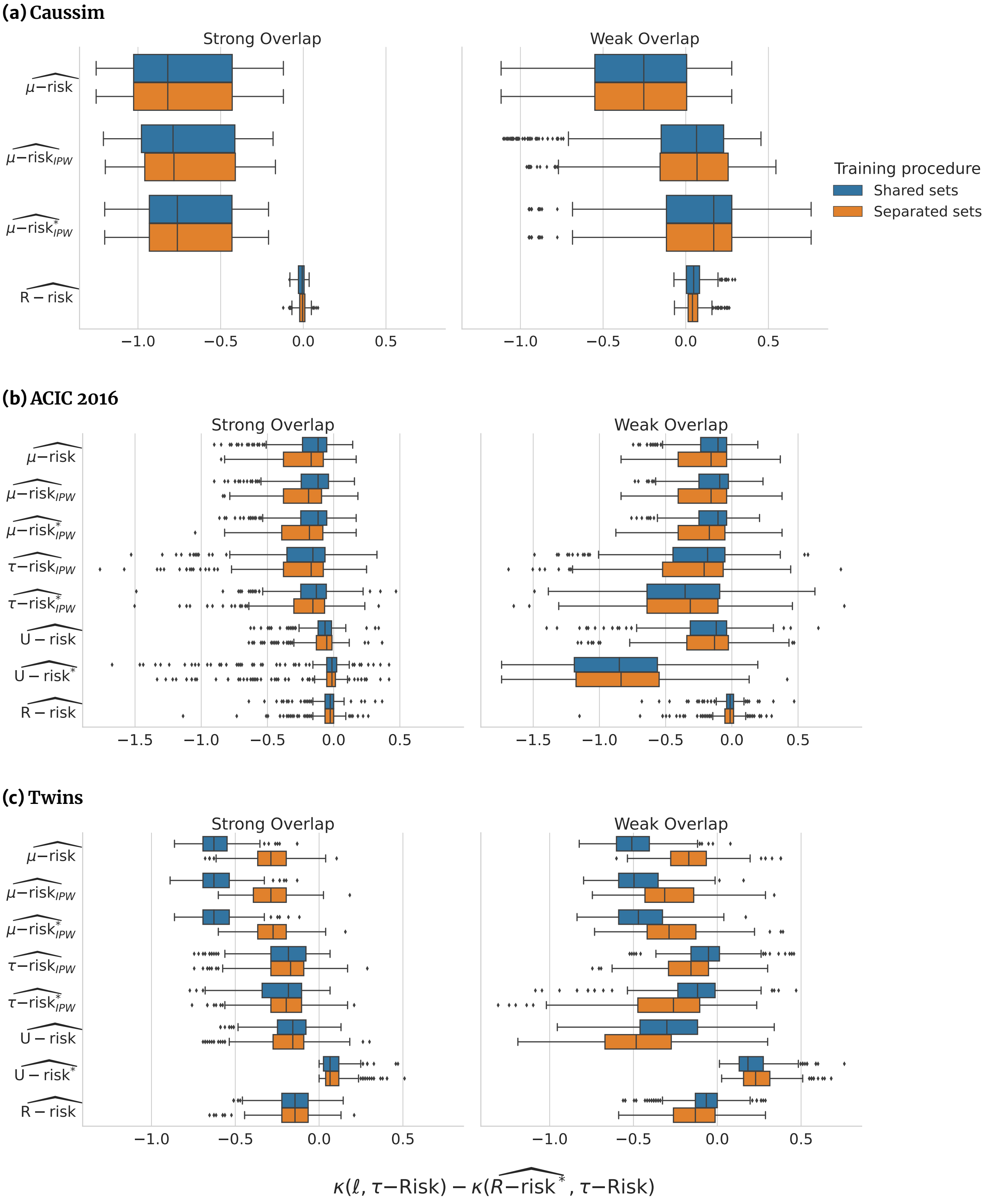

Figure 17. Results are similar between the Shared nuisances/candidate set and the Separated nuisances set procedure. The experience has not been run on the full metrics for Caussim due to computation costs.

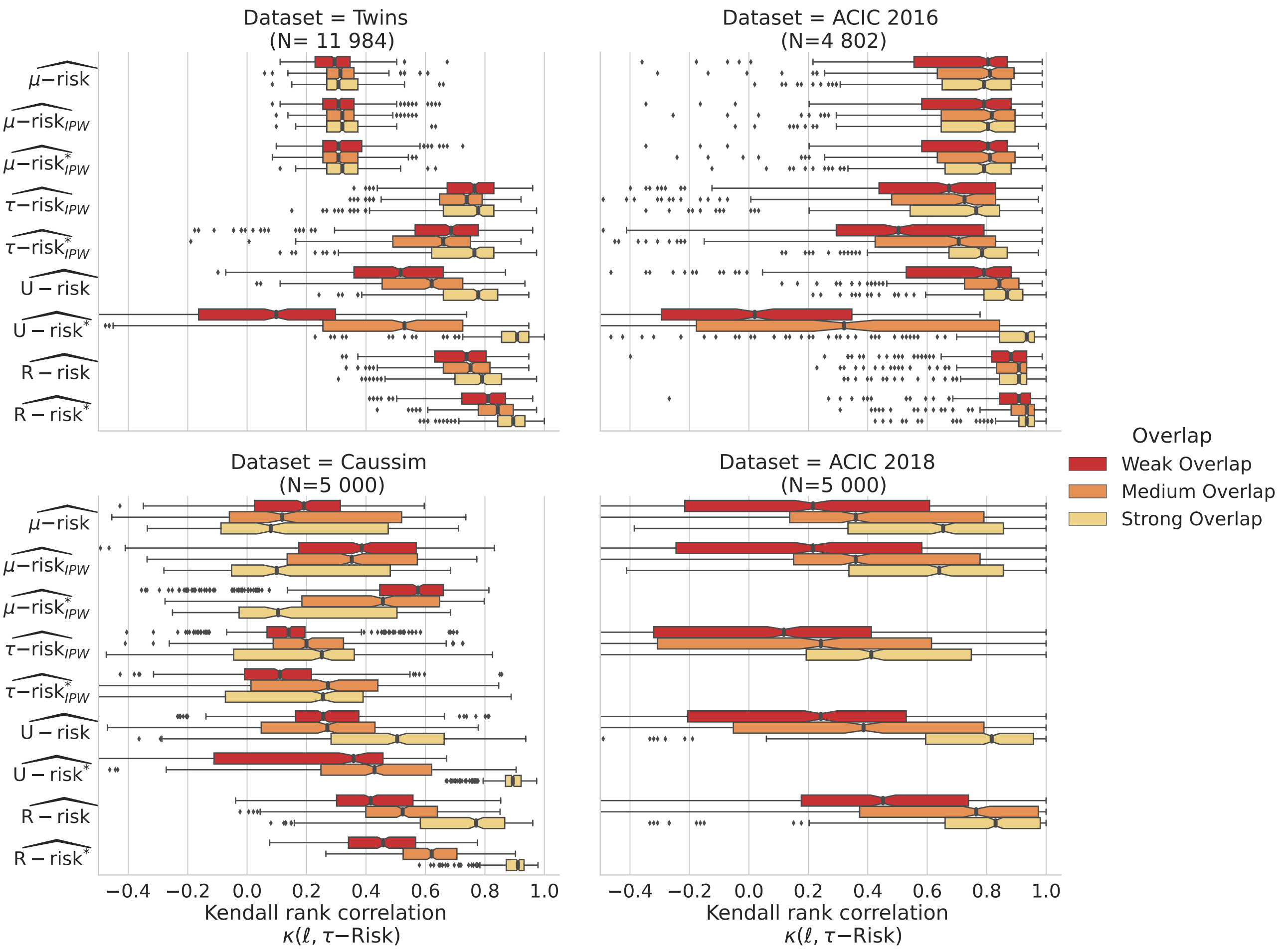

Figure 18. Low population overlap hinders causal model selection for all metrics: Kendall's τ agreement with τ-risk. Strong, medium and Weak overlap correspond to the tertiles of the overlap distribution measured with Normalized Total Varation eq. 17.

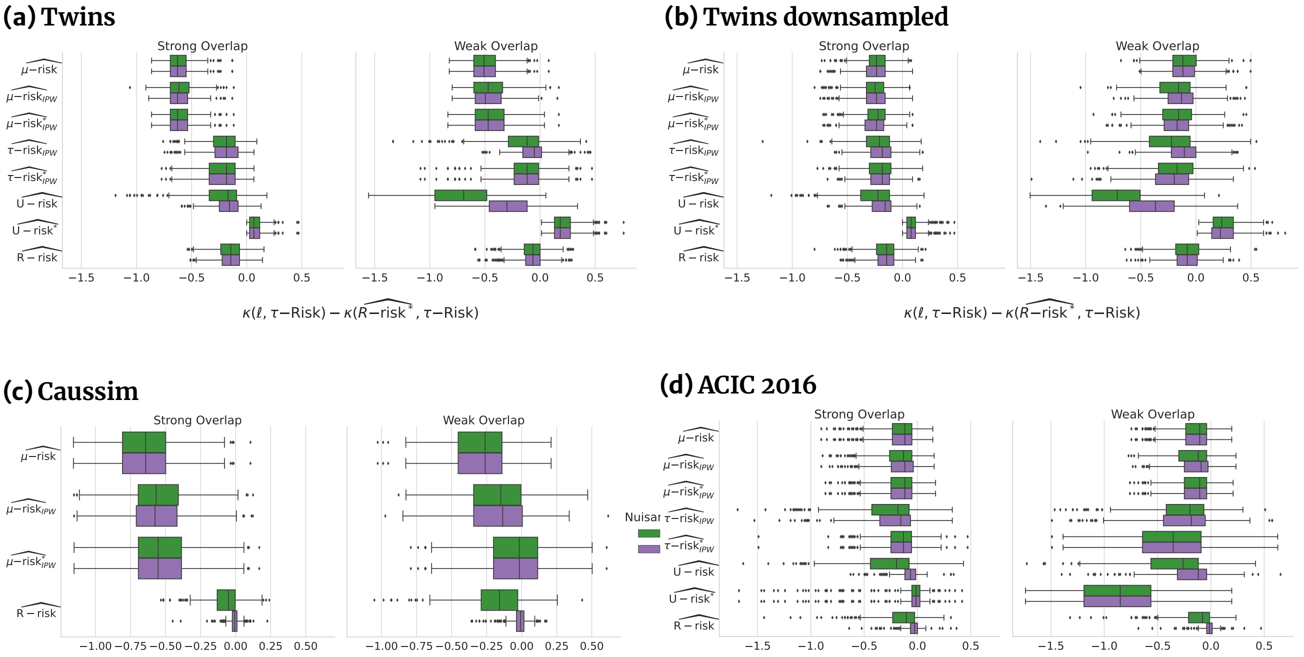

Figure 19. Learning the nuisances with stacked models (linear and gradient boosting) is important for successful model selection with R-risk. For Twins dataset, there is no improvement for stacked models compared to linear models because of the linearity of the propensity model.

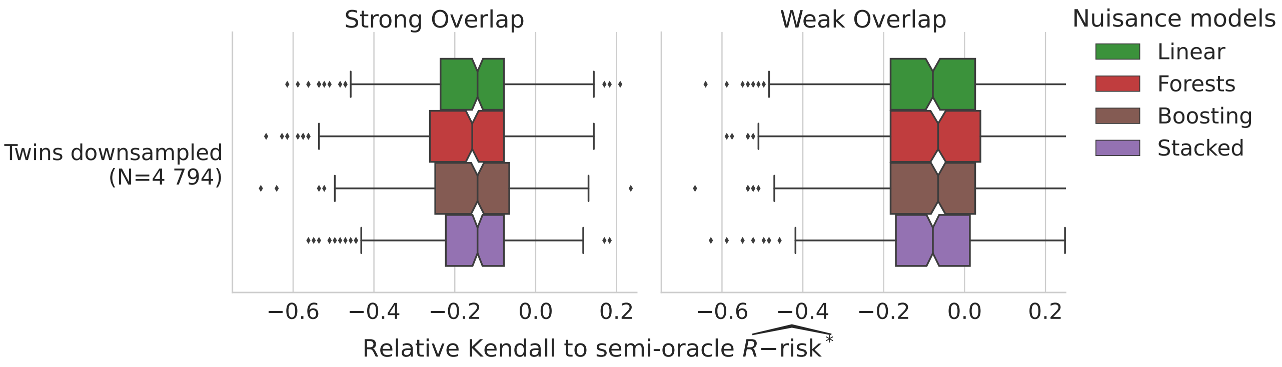

Figure 20. Flexible models are performant in recovering nuisances in the downsampled Twins dataset. The propensity score is linear in this setup, making it particularly challenging for flexible models compared to linear methods.

Figure 21. Kendall correlation coefficients for each causal metric. Each (color, shape) pair indicates a different (treatment ratio, seed) of the generation process.

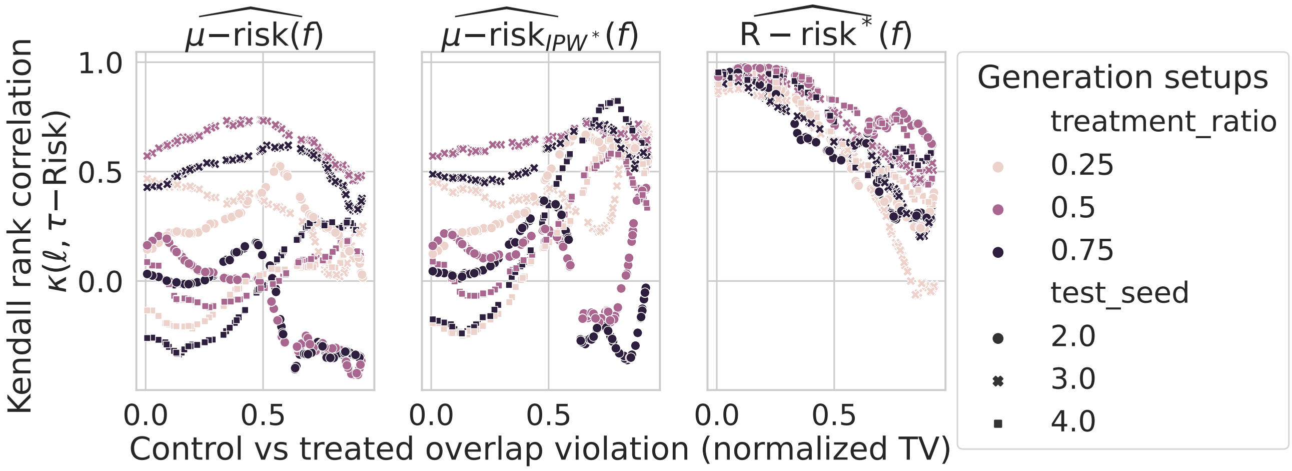

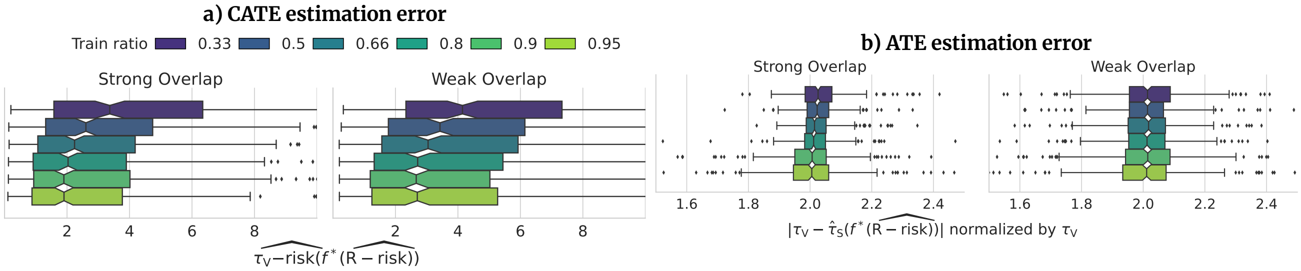

Figure 22. a) For CATE, a train/test ratio of 0.9/0.1 appears a good trade-off. b) For ATE, there is a small signal pointing also to 0.9/0.1 (K=10). for ATE. Experiences on 10 replications of all 78 instances of the ACIC 2016 data.

A.8 Data split choices

Use 90% of the data to estimate outcome models, 10% to select them

The analyst faces a compromise: given a finite data sample, should she allocate more data to estimate the outcome model, thus improving the quality of the outcome model but leaving little data for model selection. Or, she could choose a bigger test set for model selection and effect estimation. For causal model selection, there is no established practice (as reviewed in A.8).

We investigate such tradeoff varying the ratio between train and test data size. For this, we first split out 30% of the data as a holdout set $\mathcal{V}$ on which we use the oracle response functions to derive silver-standard estimates of causal quantities. We then use the standard estimation procedure on the remaining 70% of the data, splitting it into train $\mathcal{T}$ and test $\mathcal{S}$ of varying sizes. We finally measure the error between this estimate and the silver standard.

We consider two different analytic goals: estimating a average treatment effect –a single number used for policy making– and a CATE –a full model of the treatment effect as a function of covariates X. Given that the latter is a much more complex object than the former, the optimal train/test ratio might vary. To measure errors, we use for the ATE the relative absolute ATE bias between the ATE computed with the selected outcome model on the test set, and the true ATE as evaluated on the holdout set $\mathcal{V}$. For the CATE, we compare the τ-risk of the best selected model applied on the holdout set $\mathcal{V}$. We explore this trade-off for the ACIC 2016 dataset and the R-risk.

Figure 22 shows that a train/test ratio of 0.9/0.1 (K=10) or 0.8/0.2 (K=5) appears best to estimate CATE and ATE.

Heterogeneity in practices for data split

Splitting the data is common when using machine learning for causal inference, but practices vary widely in terms of the fraction of data to allocate to train models, outcomes and nuisances, and to evaluate them.

Before even model selection, data splitting is often required for estimation of the treatment effect, ATE or CATE, for instance to compute the nuisances required to optimize the outcome model (as the R-risk, Definition 5). The most frequent choice is use 80% of the data to fit the models, and 20% to evaluate them. For instance, for CATE estimation, the R-learner has been introduced using K-folds with K = 5 and K = 10: 80% of the data (4 folds) to train the nuisances and the remaining fold to minimize the corresponding R-loss [42]. Yet, it has been implemented with K=5 in causallib [93] or K=3 in econML [94]. Likewise, for ATE estimation, [43] introduce doubly-robust machine learning, recommending K=5 based on an empirical comparison K=2. However, subsequent works use doubly robust ML with varying choices of K: [95] use K=3, [96] use K=2. In the econML implementation, K is set to 3 [94]. [97] evaluate various machine-learning approaches –including R-learners– using K=5 and 10, drawing inspiration from the TMLE literature which sets K=5 in the TMLE package [98].

Causal model selection has been much less discussed. The only study that we are aware of, [50], use a different data split: a 2-folds train/test procedure, training the nuisances on the first half of the data, and using the second half to estimate the R-risk and select the best treatment effect model.

A.9 Sensitivity of the results to different ratio of causal effect to baseline response

Going beyond overlap, we study the effect of another important parameter of the data generation process: the ratio of causal effect to the baseline response.

Here, we detail the experimental setup of the results presented in Figure 7.

For each dataset of our simulation (caussim), we measure this ratio as the absolute mean difference between the causal effect and the baseline response, measured empirically on each dataset instance $\Delta_\mu = \frac{1}{N}\sum_{i=1}^{N} \left| \frac{\mu_1(x_i)-\mu_0(x_i)}{\mu_0(x_i)+\mu_1(x_i)-\frac{1}{N}\sum_{j=1}^{N}\mu_0(x_j)+\mu_1(x_j)} \right|$. We vary a dedicated parameter (ω in Algorithm 2) in the simulation to force this ratio to cover a wide range of values. For 179 dataset instances, it ranges several order of magnitudes with the full distribution of values given in Table 5.

	count	mean	std	min	1%	10%	25%	50%	75%	90%	99%	max
**effect_ratio**	179	16.95	24.92	0.043	0.054	0.32	1.34	9.12	22.55	39.97	101.12	206.53

Table 5. Distribution of the causal effect ratio Δ_μ for the experiment on causal effect ratio, results in Figure 7.

We also studied another measure, focusing on the ratio of the variance of the causal effect to the variance of the mean shared response ($\mu_0 + \mu_1$) of both population: $\frac{\hat{var}(\mu_1(x_i)-\mu_0(x_i))}{\hat{var}(\mu_1(x_i)+\mu_0(x_i))}$, covering a range from 0 to 11.08 with the full distribution described in Table 6. Figure 23 shows that for high values of the variance, $R-$risk is outperformed by the μ-risk$_{IPW}$ and the τ-risk$_{IPW}$. However, on average, the $R-$risk

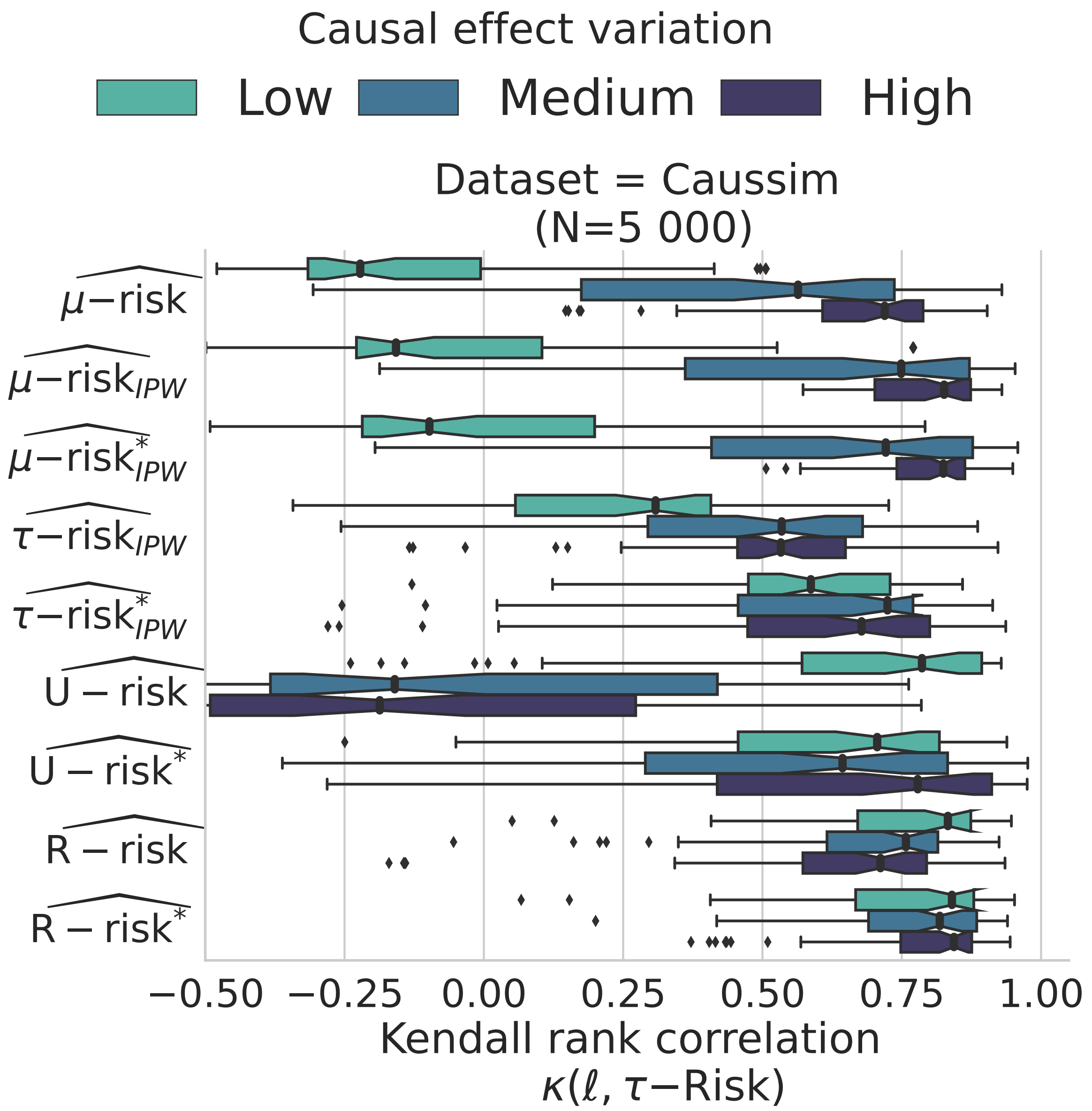

Figure 23. R-risk is robust to a wide range of effect variance: Kendall's τ agreement with τ-risk. Strong, medium and Weak Causal effect variance are the tertiles of the ratio of causal effect variance to the mean outcome variance: Low [0; 0.04[, Medium [0.04; 1.04[, High [1.04; 11.08[.

is still the better risk.

	count	mean	std	min	1%	10%	25%	50%	75%	90%	99%	max
**effect_variance**	179	1.013	1.781	1.680e-5	1.734e-5	4.875e-4	6.449e-3	0.789	1.160	1.807	9.641	11.079

Table 6. Statistical summary of the causal effect variance.

References

1. Karel GM Moons, Patrick Royston, Yvonne Vergouwe, Diederick E Grobbee, and Douglas G Altman. Prognosis and prognostic research: what, why, and how? *Bmj*, 338, 2009.
2. Ewout W Steyerberg. *Clinical prediction models*. Springer.
3. Andrew L Beam and Isaac S Kohane. Big data and machine learning in health care. *Jama*, 319(13):1317–1318, 2018.
4. Alvin Rajkomar, Jeffrey Dean, and Isaac Kohane. Machine learning in medicine. *New England Journal of Medicine*, 380(14):1347–1358, 2019.
5. M Khojaste-Sarakhsi, Seyedhamidreza Shahabi Haghighi, SMT Fatemi Ghomi, and Elena Marchiori. Deep learning for alzheimer's disease diagnosis: A survey. *Artificial Intelligence in Medicine*, page 102332, 2022.

6. Zhenwei Zhang and Ervin Sejdić. Radiological images and machine learning: trends, perspectives, and prospects. *Computers in biology and medicine*, 108:354–370, 2019.
7. Adam Yala, Constance Lehman, Tal Schuster, Tally Portnoi, and Regina Barzilay. A deep learning mammography-based model for improved breast cancer risk prediction. *Radiology*, 292(1):60–66, 2019.
8. Li Shen, Laurie R Margolies, Joseph H Rothstein, Eugene Fluder, Russell McBride, and Weiva Sieh. Deep learning to improve breast cancer detection on screening mammography. *Scientific reports*, 9(1):12495, 2019.
9. Ali Bou Nassif, Manar Abu Talib, Qassim Nasir, Yaman Afadar, and Omar Elgendy. Breast cancer detection using artificial intelligence techniques: A systematic literature review. *Artificial Intelligence in Medicine*, page 102276, 2022.
10. Stephen J Mooney and Vikas Pejaver. Big data in public health: terminology, machine learning, and privacy. *Annual review of public health*, 39:95, 2018.
11. Rishi J Desai, Shirley V Wang, Muthiah Vaduganathan, Thomas Evers, and Sebastian Schneeweiss. Comparison of machine learning methods with traditional models for use of administrative claims with electronic medical records to predict heart failure outcomes. *JAMA network open*, 3(1):e1918962–e1918962, 2020.
12. Gregory E Simon, Eric Johnson, Jean M Lawrence, Rebecca C Rossom, Brian Ahmedani, Frances L Lynch, Arne Beck, Beth Waitzfelder, Rebecca Ziebell, Robert B Penfold, et al. Predicting suicide attempts and suicide deaths following outpatient visits using electronic health records. *American Journal of Psychiatry*, 175(10):951–960, 2018.
13. Steven Horng, David A Sontag, Yoni Halpern, Yacine Jernite, Nathan I Shapiro, and Larry A Nathanson. Creating an automated trigger for sepsis clinical decision support at emergency department triage using machine learning. *PloS one*, 12(4):e0174708, 2017.
14. Hanyin Wang, Yikuan Li, Seema A Khan, and Yuan Luo. Prediction of breast cancer distant recurrence using natural language processing and knowledge-guided convolutional neural network. *Artificial intelligence in medicine*, 110:101977, 2020.
15. Irena Spasic, Goran Nenadic, et al. Clinical text data in machine learning: systematic review. *JMIR medical informatics*, 8(3):e17984, 2020.
16. Douglas G Altman, Yvonne Vergouwe, Patrick Royston, and Karel GM Moons. Prognosis and prognostic research: validating a prognostic model. *Bmj*, 338, 2009.
17. Russell A Poldrack, Grace Huckins, and Gael Varoquaux. Establishment of best practices for evidence for prediction: a review. *JAMA psychiatry*, 77(5):534–540, 2020.
18. Gaël Varoquaux and Olivier Colliot. Evaluating machine learning models and their diagnostic value, 2022.
19. Jeremy C Wyatt and Douglas G Altman. Commentary: Prognostic models: clinically useful or quickly forgotten? *Bmj*, 311(7019): 1539–1541, 1995.
20. Mark Alan Fontana, Stephen Lyman, Gourab K Sarker, Douglas E Padgett, and Catherine H MacLean. Can machine learning algorithms predict which patients will achieve minimally clinically important differences from total joint arthroplasty? *Clinical orthopaedics and related research*, 477(6):1267, 2019.
21. Jonathan M. Snowden, Sherri Rose, and Kathleen M. Mortimer. Implementation of G-computation on a simulated data set: demonstration of a causal inference technique. *American Journal of Epidemiology*, 173(7):731–738, April 2011.
22. Matthew Sperrin, David Jenkins, Glen P Martin, and Niels Peek. Explicit causal reasoning is needed to prevent prognostic models being victims of their own success. *Journal of the American Medical Informatics Association*, 26(12):1675–1676, 2019.
23. Tony Blakely, John Lynch, Koen Simons, Rebecca Bentley, and Sherri Rose. Reflection on modern methods: when worlds collide—prediction, machine learning and causal inference. *International journal of epidemiology*, 49(6):2058–2064, 2020.
24. MA Hernán and JM Robins. *Causal Inference: What If.* CRC Boca Raton, FL.
25. Tyler J VanderWeele. Principles of confounder selection. *European journal of epidemiology*, 34:211–219, 2019.
26. James M. Robins and Sander Greenland. The role of model selection in causal inference from non experimental data. *American Journal of Epidemiology*, 123(3):392–402.
27. T. Wendling, K. Jung, A. Callahan, A. Schuler, N. H. Shah, and B. Gallego. Comparing methods for estimation of heterogeneous treatment effects using observational data from health care databases. *Statistics in Medicine*, (23):3309–3324.
28. Paul R Rosenbaum and Donald B Rubin. The central role of the propensity score in observational studies for causal effects. *Biometrika*, 70:41–55.
29. Peter C. Austin and Elizabeth A. Stuart. Moving towards best practice when using inverse probability of treatment weighting (IPTW) using the propensity score to estimate causal treatment effects in observational studies. *Statistics in Medicine*, 34(28):3661–3679.
30. Sabrina Casucci, Li Lin, Sharon Hewner, and Alexander Nikolaev. Estimating the causal effects of chronic disease combinations on 30-day hospital readmissions based on observational medicaid data. *Journal of the American Medical Informatics Association*, 25(6): 670–678, 2018.
31. Elysia Grose, Samuel Wilson, Jeffrey Barkun, Kimberly Bertens, Guillaume Martel, Fady Balaa, and Jad Abou Khalil. Use of propensity score methodology in contemporary high-impact surgical literature. *Journal of the American College of Surgeons*, 230(1):101–112.e2.
32. Xiaogang Su, Annette T Peña, Lei Liu, and Richard A Levine. Random forests of interaction trees for estimating individualized treatment effects in randomized trials. *Statistics in medicine*, 37(17):2547–2560, 2018.
33. Andrea Lamont, Michael D Lyons, Thomas Jaki, Elizabeth Stuart, Daniel J Feaster, Kukatharmini Tharmaratnam, Daniel Oberski, Hemant Ishwaran, Dawn K Wilson, and M Lee Van Horn. Identification of predicted individual treatment effects in randomized clinical trials. *Statistical methods in medical research*, 27(1):142–157, 2018.
34. Jeroen Hoogland, Joanna IntHout, Michail Belias, Maroeska M Rovers, Richard D Riley, Frank E. Harrell Jr, Karel GM Moons, Thomas PA Debray, and Johannes B Reitsma. A tutorial on individualized treatment effect prediction from randomized trials with a binary endpoint. *Statistics in medicine*, 40(26):5961–5981, 2021.
35. Jennifer L. Hill. Bayesian nonparametric modeling for causal inference. *Journal of Computational and Graphical Statistics*, (1):217–240.
36. Mark J. van der Laan and Sherri Rose. *Targeted Learning.* Springer Series in Statistics.
37. Megan S. Schuler and Sherri Rose. Targeted maximum likelihood estimation for causal inference in observational studies. *American Journal of Epidemiology*, 185(1):65–73.
38. Scott Powers, Junyang Qian, Kenneth Jung, Alejandro Schuler, Nigam H. Shah, Trevor Hastie, and Robert Tibshirani. Some methods for heterogeneous treatment effect estimation in high dimensions. *Statistics in Medicine*, 37(11):1767–1787.
39. Stefan Wager and Susan Athey. Estimation and inference of heterogeneous treatment effects using random forests. *Journal of the*

American Statistical Association, 113(523):1228–1242.

40. Susan Athey, Julie Tibshirani, and Stefan Wager. Generalized random forests. *Annals of Statistics*, 47(2):1148–1178.
41. Sören R. Künzel, Jasjeet S. Sekhon, Peter J. Bickel, and Bin Yu. Metalearners for estimating heterogeneous treatment effects using machine learning. *Proceedings of the National Academy of Sciences*, 116(10):4156–4165.
42. Xinkun Nie and Stefan Wager. Quasi-oracle estimation of heterogeneous treatment effects. *Biometrika*, 108(2):299–319.
43. Victor Chernozhukov, Denis Chetverikov, Mert Demirer, Esther Duflo, Christian Hansen, Whitney Newey, and James Robins. Double/debiased machine learning for treatment and structural parameters. *The Econometrics Journal*, page 71.
44. Gang Fang, Izabela E Annis, Jennifer Elston-Lafata, and Samuel Cykert. Applying machine learning to predict real-world individual treatment effects: insights from a virtual patient cohort. *Journal of the American Medical Informatics Association*, 26(10):977–988, 2019.
45. Vincent Dorie, Jennifer Hill, Uri Shalit, Marc Scott, and Dan Cervone. Automated versus do-it-yourself methods for causal inference: Lessons learned from a data analysis competition. *Statistical Science*, 34(1):43–68, 2019.
46. Florent Le Borgne, Arthur Chatton, Maxime Léger, Rémi Lenain, and Yohann Foucher. G-computation and machine learning for estimating the causal effects of binary exposure statuses on binary outcomes. *Scientific reports*, 11(1):1435, 2021.
47. Jinma Ren, Paul Cislo, Joseph C Cappelleri, Patrick Hlavacek, and Marco DiBonaventura. Comparing g-computation, propensity score-based weighting, and targeted maximum likelihood estimation for analyzing externally controlled trials with both measured and unmeasured confounders: a simulation study. *BMC Medical Research Methodology*, 23(1):18, 2023.
48. Jean Kaddour, Aengus Lynch, Qi Liu, Matt J Kusner, and Ricardo Silva. Causal machine learning: A survey and open problems. arxiv 2022. *arXiv preprint arXiv:2206.15475*, 2022.
49. Victor Chernozhukov, Christian Hansen, Nathan Kallus, Martin Spindler, and Vasilis Syrgkanis. Applied causal inference powered by ml and ai. *arXiv preprint arXiv:2403.02467*, 2024.
50. Alejandro Schuler, Michael Baiocchi, Robert Tibshirani, and Nigam Shah. A comparison of methods for model selection when estimating individual treatment effects. *arXiv:1804.05146 [cs, stat]*.
51. Ahmed Alaa and Mihaela Van Der Schaar. Validating causal inference models via influence functions. *International Conference on Machine Learning*, pages 191–201.
52. Mary E. Charlson, Peter Pompei, Kathy L. Ales, and C.Ronald MacKenzie. A new method of classifying prognostic comorbidity in longitudinal studies: Development and validation. *Journal of Chronic Diseases*, 40(5):373–383.
53. James Robins. A new approach to causal inference in mortality studies with a sustained exposure period—application to control of the healthy worker survivor effect. *Mathematical Modelling*, 7(9):1393–1512.
54. Ashley I Naimi and Brian W Whitcomb. Defining and identifying average treatment effects. *American Journal of Epidemiology*, 2023.
55. Guido W. Imbens and Donald B. Rubin. *Causal inference in statistics, social, and biomedical sciences*. Cambridge University Press.
56. Donald B Rubin. Causal inference using potential outcomes. *Journal of the American Statistical Association*, 100(469):322–331.
57. P. M. Robinson. Root-n-consistent semiparametric regression. *Econometrica*, (4):931–954.
58. Mark J van der Laan, MJ Laan, and JM Robins. *Unified methods for censored longitudinal data and causality*. Springer Science & Business Media.
59. Peter Schulam and Suchi Saria. Reliable decision support using counterfactual models. *Advances in neural information processing systems*, 30.
60. Uri Shalit, Fredrik D Johansson, and David Sontag. Estimating individual treatment effect: generalization bounds and algorithms. In *International Conference on Machine Learning*, pages 3076–3085. PMLR, 2017.
61. Chanelle J. Howe, Stephen R. Cole, Daniel J. Westreich, Sander Greenland, Sonia Napravnik, and Joseph J. Eron. Splines for trend analysis and continuous confounder control. 22(6):874–875.
62. Aris Perperoglou, Willi Sauerbrei, Michal Abrahamowicz, and Matthias Schmid. A review of spline function procedures in r. 19(1):46.
63. Ali Rahimi and Benjamin Recht. Random features for large-scale kernel machines. In *Advances in Neural Information Processing Systems*, volume 20.
64. Lingjie Shen, Gijs Geleijnse, and Maurits Kaptein. Rctrep: An r package for the validation of estimates of average treatment effects. *Journal of Statistical Software*, 2023.
65. Kenneth R. Niswander and United States National Institute of Neurologiacal Diseases and Stroke. *The Women and Their Pregnancies: The Collaborative Perinatal Study of the National Institute of Neurological Diseases and Stroke*. National Institute of Health. Google-Books-ID: A0bdVhlhDQkC.
66. Yishai Shimoni, Chen Yanover, Ehud Karavani, and Yaara Goldschmnidt. Benchmarking framework for performance-evaluation of causal inference analysis. *arXiv:1802.05046 [cs, stat]*.
67. M. F. MacDorman and J. O. Atkinson. Infant mortality statistics from the linked birth/infant death data set–1995 period data. *Monthly Vital Statistics Report*, 46(6 Suppl 2):1–22, February 1998.
68. Christos Louizos, Uri Shalit, Joris Mooij, David Sontag, Richard Zemel, and Max Welling. Causal effect inference with deep latent-variable models. *Advances in neural information processing systems*.
69. Douglas Almond, Kenneth Y. Chay, and David S. Lee. The Costs of Low Birth Weight. *The Quarterly Journal of Economics*, 120(3), 2005.
70. M. G. Kendall. A new measure of rank correlation. *Biometrika*, 30(1-2):81–93, June 1938.
71. Susan Athey and Guido Imbens. Recursive partitioning for heterogeneous causal effects. *Proceedings of the National Academy of Sciences*, 113(27):7353–7360, 2016.
72. Pierre Gutierrez and Jean-Yves Gerardy. Causal inference and uplift modeling a review of the literature. *Proceedings of The 3rd International Conference on Predictive Applications and APIs*, (67):14.
73. John C. Platt and John C. Platt. Probabilistic Outputs for Support Vector Machines and Comparisons to Regularized Likelihood Methods. *Advances in Large Margin Classifiers*, pages 61–74, 1999.
74. Bianca Zadrozny and Charles Elkan. Obtaining calibrated probability estimates from decision trees and naive Bayesian classifiers. page 8.
75. Alexandru Niculescu-Mizil and Rich Caruana. Predicting good probabilities with supervised learning. In *Proceedings of the 22nd international conference on Machine learning - ICML '05*, pages 625–632. ACM Press, 2005.
76. Matthias Minderer, Josip Djolonga, Rob Romijnders, Frances Hubis, Xiaohua Zhai, Neil Houlsby, Dustin Tran, and Mario Lucic. Revisiting the Calibration of Modern Neural Networks. *Advances in Neural Information Processing Systems*, 34:15682–15694, 2021.

77. Alexandre Perez-Lebel, Marine Le Morvan, and Gaël Varoquaux. Beyond calibration: estimating the grouping loss of modern neural networks. *arXiv preprint arXiv:2210.16315*, 2022.
78. Xavier Bouthillier, Pierre Delaunay, Mirko Bronzi, Assya Trofimov, Brennan Nichyporuk, Justin Szeto, Nazanin Mohammadi Sepahvand, Edward Raff, Kanika Madan, Vikram Voleti, Samira Ebrahimi Kahou, Vincent Michalski, Tal Arbel, Chris Pal, Gael Varoquaux, and Pascal Vincent. Accounting for variance in machine learning benchmarks. *Proceedings of Machine Learning and Systems*, 3:747–769.
79. Fabian Pedregosa, Gaël Varoquaux, Alexandre Gramfort, Vincent Michel, Bertrand Thirion, Olivier Grisel, Mathieu Blondel, Peter Prettenhofer, Ron Weiss, Vincent Dubourg, Jake Vanderplas, Alexandre Passos, David Cournapeau, Matthieu Brucher, Matthieu Perrot, and Édouard Duchesnay. Scikit-learn: Machine learning in python. *Journal of Machine Learning Research*, 12(85):2825–2830.
80. Alexander D'Amour, Peng Ding, Avi Feller, Lihua Lei, and Jasjeet Sekhon. Overlap in observational studies with high-dimensional covariates. *Journal of Econometrics*, 221(2):644–654, 2021.
81. Craig A. Rolling and Yuhong Yang. Model selection for estimating treatment effects. 76(4):749–769.
82. Yuta Saito and Shota Yasui. Counterfactual cross-validation: Stable model selection procedure for causal inference models. In *International Conference on Machine Learning*, pages 8398–8407. PMLR, 2020.
83. Fredrik D Johansson, Uri Shalit, Nathan Kallus, and David Sontag. Generalization bounds and representation learning for estimation of potential outcomes and causal effects. *The Journal of Machine Learning Research*, 23(1):7489–7538, 2022.
84. Dylan J Foster and Vasilis Syrgkanis. Orthogonal statistical learning. *The Annals of Statistics*, 51(3):879–908, 2023.
85. Andrew Jesson, Sören Mindermann, Uri Shalit, and Yarin Gal. Identifying causal-effect inference failure with uncertainty-aware models. *Advances in Neural Information Processing Systems*, 33:11637–11649.
86. Peter C. Austin. An Introduction to Propensity Score Methods for Reducing the Effects of Confounding in Observational Studies. *Multivariate Behavioral Research*, (3):399–424, May 2011.
87. Arthur Gretton, Karsten M Borgwardt, Malte J Rasch, Bernhard Schölkopf, and Alexander Smola. A kernel two-sample test. *The Journal of Machine Learning Research*, 13(1):723–773.
88. Bharath K. Sriperumbudur, Kenji Fukumizu, Arthur Gretton, Bernhard Schölkopf, and Gert R. G. Lanckriet. On integral probability metrics, \phi-divergences and binary classification. *arXiv:0901.2698 [cs, math]*.
89. Alicia Curth, David Svensson, and James Weatherall. Really doing great at estimating CATE? a critical look at ML benchmarking practices in treatment effect estimation. *Neurips Process 2021*, page 14.
90. Mark J. van der Laan, Eric C. Polley, and Alan E. Hubbard. Super learner. 6.
91. Adith Swaminathan and Thorsten Joachims. Counterfactual risk minimization: Learning from logged bandit feedback. In *International Conference on Machine Learning*, pages 814–823. PMLR, 2015.
92. Edward L Ionides. Truncated importance sampling. *Journal of Computational and Graphical Statistics*, 17(2):295–311.
93. Yishai Shimoni, Ehud Karavani, Sivan Ravid, Peter Bak, Tan Hung Ng, Sharon Hensley Alford, Denise Meade, and Yaara Goldschmidt. An evaluation toolkit to guide model selection and cohort definition in causal inference. *arXiv preprint arXiv:1906.00442*, 2019.
94. Keith Battocchi, Eleanor Dillon, Maggie Hei, Greg Lewis, Paul Oka, Miruna Oprescu, and Vasilis Syrgkanis. EconML: A Python Package for ML-Based Heterogeneous Treatment Effects Estimation. https://github.com/microsoft/EconML.
95. Nicolas Loiseau, Paul Trichelair, Maxime He, Mathieu Andreux, Mikhail Zaslavskiy, Gilles Wainrib, and Michael G. B. Blum. External control arm analysis: an evaluation of propensity score approaches, G-computation, and doubly debiased machine learning. *BMC Medical Research Methodology*, 22, 2022.
96. Zijun Gao, Trevor Hastie, and Robert Tibshirani. Assessment of heterogeneous treatment effect estimation accuracy via matching. *Statistics in Medicine*, (17), 2021.
97. Ashley I Naimi, Alan E Mishler, and Edward H Kennedy. Challenges in obtaining valid causal effect estimates with machine learning algorithms. *American Journal of Epidemiology*, 2021.
98. Susan Gruber and Mark J. van der Laan. tmle: An R package for targeted maximum likelihood estimation. *Journal of Statistical Software*, 51(13):1–35, 2012.

Click here to
access/download;Table;_1_r_risk_domination_boxplot_df.tex

```
This is pdfTeX, Version 3.141592653-2.6-1.40.26 (TeX Live 2024)
(preloaded format=pdflatex 2024.8.2)  10 NOV 2024 12:07
entering extended mode
 restricted \write18 enabled.
 %&-line parsing enabled.
**_1_r_risk_domination_boxplot_df.tex
(./_1_r_risk_domination_boxplot_df.tex
LaTeX2e <2024-06-01> patch level 2
L3 programming layer <2024-05-27>

! LaTeX Error: Missing \begin{document}.

See the LaTeX manual or LaTeX Companion for explanation.
Type  H <return>  for immediate help.
 ...

l.1 \begin{tabular}
                   {llrrrr}
You're in trouble here.  Try typing  <return>  to proceed.
If that doesn't work, type  X <return>  to quit.

LaTeX Font Info:    External font `cmex10' loaded for size
(Font)              <7> on input line 1.
LaTeX Font Info:    External font `cmex10' loaded for size
(Font)              <5> on input line 1.
! Undefined control sequence.
l.2     \toprule

The control sequence at the end of the top line
of your error message was never \def'ed. If you have
misspelled it (e.g., `\hobx'), type `I' and the correct
spelling (e.g., `I\hbox'). Otherwise just continue,
and I'll forget about whatever was undefined.

Missing character: There is no S in font nullfont!
Missing character: There is no t in font nullfont!
Missing character: There is no r in font nullfont!
Missing character: There is no o in font nullfont!
Missing character: There is no n in font nullfont!
Missing character: There is no g in font nullfont!
Missing character: There is no O in font nullfont!
Missing character: There is no v in font nullfont!
Missing character: There is no e in font nullfont!
Missing character: There is no r in font nullfont!
Missing character: There is no l in font nullfont!
Missing character: There is no a in font nullfont!
Missing character: There is no p in font nullfont!
Missing character: There is no W in font nullfont!
Missing character: There is no e in font nullfont!
Missing character: There is no a in font nullfont!
Missing character: There is no k in font nullfont!
Missing character: There is no O in font nullfont!
Missing character: There is no v in font nullfont!
Missing character: There is no e in font nullfont!
```

```
Missing character: There is no r in font nullfont!
Missing character: There is no l in font nullfont!
Missing character: There is no a in font nullfont!
Missing character: There is no p in font nullfont!
! Undefined control sequence.
<recently read> \midrule

l.8     \\ \midrule

The control sequence at the end of the top line
of your error message was never \def'ed. If you have
misspelled it (e.g., `\hobx'), type `I' and the correct
spelling (e.g., `I\hbox'). Otherwise just continue,
and I'll forget about whatever was undefined.

Missing character: There is no M in font nullfont!
Missing character: There is no e in font nullfont!
Missing character: There is no d in font nullfont!
Missing character: There is no i in font nullfont!
Missing character: There is no a in font nullfont!
Missing character: There is no n in font nullfont!
Missing character: There is no I in font nullfont!
Missing character: There is no Q in font nullfont!
Missing character: There is no R in font nullfont!
Missing character: There is no M in font nullfont!
Missing character: There is no e in font nullfont!
Missing character: There is no d in font nullfont!
Missing character: There is no i in font nullfont!
Missing character: There is no a in font nullfont!
Missing character: There is no n in font nullfont!
Missing character: There is no I in font nullfont!
Missing character: There is no Q in font nullfont!
Missing character: There is no R in font nullfont!
Missing character: There is no M in font nullfont!
Missing character: There is no e in font nullfont!
Missing character: There is no t in font nullfont!
Missing character: There is no r in font nullfont!
Missing character: There is no i in font nullfont!
Missing character: There is no c in font nullfont!
Missing character: There is no D in font nullfont!
Missing character: There is no a in font nullfont!
Missing character: There is no t in font nullfont!
Missing character: There is no a in font nullfont!
Missing character: There is no s in font nullfont!
Missing character: There is no e in font nullfont!
Missing character: There is no t in font nullfont!
! Undefined control sequence.
<recently read> \midrule

l.11     \midrule

The control sequence at the end of the top line
of your error message was never \def'ed. If you have
misspelled it (e.g., `\hobx'), type `I' and the correct
```

```
spelling (e.g., `I\hbox'). Otherwise just continue,
and I'll forget about whatever was undefined.

! Undefined control sequence.
l.12     \multirow
                  [c]{4}{*}{$\widehat{\mu\mathrm{-risk}}$}            &
Twins
The control sequence at the end of the top line
of your error message was never \def'ed. If you have
misspelled it (e.g., `\hobx'), type `I' and the correct
spelling (e.g., `I\hbox'). Otherwise just continue,
and I'll forget about whatever was undefined.

Missing character: There is no [ in font nullfont!
Missing character: There is no c in font nullfont!
Missing character: There is no ] in font nullfont!
Missing character: There is no 4 in font nullfont!
Missing character: There is no * in font nullfont!
Missing character: There is no T in font nullfont!
Missing character: There is no w in font nullfont!
Missing character: There is no i in font nullfont!
Missing character: There is no n in font nullfont!
Missing character: There is no s in font nullfont!
Missing character: There is no ( in font nullfont!
Missing character: There is no N in font nullfont!
Missing character: There is no = in font nullfont!
Missing character: There is no 1 in font nullfont!
Missing character: There is no 1 in font nullfont!
Missing character: There is no 9 in font nullfont!
Missing character: There is no 8 in font nullfont!
Missing character: There is no 4 in font nullfont!
Missing character: There is no ) in font nullfont!
Missing character: There is no - in font nullfont!
Missing character: There is no 0 in font nullfont!
Missing character: There is no . in font nullfont!
Missing character: There is no 3 in font nullfont!
Missing character: There is no 2 in font nullfont!
Missing character: There is no 0 in font nullfont!
Missing character: There is no . in font nullfont!
Missing character: There is no 1 in font nullfont!
Missing character: There is no 2 in font nullfont!
Missing character: There is no - in font nullfont!
Missing character: There is no 0 in font nullfont!
Missing character: There is no . in font nullfont!
Missing character: There is no 1 in font nullfont!
Missing character: There is no 9 in font nullfont!
Missing character: There is no 0 in font nullfont!
Missing character: There is no . in font nullfont!
Missing character: There is no 1 in font nullfont!
Missing character: There is no 2 in font nullfont!
Missing character: There is no A in font nullfont!
Missing character: There is no C in font nullfont!
Missing character: There is no I in font nullfont!
Missing character: There is no C in font nullfont!
```

Missing character: There is no 2 in font nullfont!
Missing character: There is no 0 in font nullfont!
Missing character: There is no 1 in font nullfont!
Missing character: There is no 6 in font nullfont!
Missing character: There is no (in font nullfont!
Missing character: There is no N in font nullfont!
Missing character: There is no = in font nullfont!
Missing character: There is no 4 in font nullfont!
Missing character: There is no 8 in font nullfont!
Missing character: There is no 0 in font nullfont!
Missing character: There is no 2 in font nullfont!
Missing character: There is no) in font nullfont!
Missing character: There is no - in font nullfont!
Missing character: There is no 0 in font nullfont!
Missing character: There is no . in font nullfont!
Missing character: There is no 0 in font nullfont!
Missing character: There is no 3 in font nullfont!
Missing character: There is no 0 in font nullfont!
Missing character: There is no . in font nullfont!
Missing character: There is no 1 in font nullfont!
Missing character: There is no 3 in font nullfont!
Missing character: There is no 0 in font nullfont!
Missing character: There is no . in font nullfont!
Missing character: There is no 1 in font nullfont!
Missing character: There is no 1 in font nullfont!
Missing character: There is no 0 in font nullfont!
Missing character: There is no . in font nullfont!
Missing character: There is no 1 in font nullfont!
Missing character: There is no 9 in font nullfont!
Missing character: There is no C in font nullfont!
Missing character: There is no a in font nullfont!
Missing character: There is no u in font nullfont!
Missing character: There is no s in font nullfont!
Missing character: There is no s in font nullfont!
Missing character: There is no i in font nullfont!
Missing character: There is no m in font nullfont!
Missing character: There is no (in font nullfont!
Missing character: There is no N in font nullfont!
Missing character: There is no = in font nullfont!
Missing character: There is no 5 in font nullfont!
Missing character: There is no 0 in font nullfont!
Missing character: There is no 0 in font nullfont!
Missing character: There is no 0 in font nullfont!
Missing character: There is no) in font nullfont!
Missing character: There is no - in font nullfont!
Missing character: There is no 0 in font nullfont!
Missing character: There is no . in font nullfont!
Missing character: There is no 4 in font nullfont!
Missing character: There is no 0 in font nullfont!
Missing character: There is no 0 in font nullfont!
Missing character: There is no . in font nullfont!
Missing character: There is no 5 in font nullfont!
Missing character: There is no 5 in font nullfont!
Missing character: There is no - in font nullfont!

```
Missing character: There is no 0 in font nullfont!
Missing character: There is no . in font nullfont!
Missing character: There is no 1 in font nullfont!
Missing character: There is no 6 in font nullfont!
Missing character: There is no 0 in font nullfont!
Missing character: There is no . in font nullfont!
Missing character: There is no 3 in font nullfont!
Missing character: There is no 1 in font nullfont!
Missing character: There is no A in font nullfont!
Missing character: There is no C in font nullfont!
Missing character: There is no I in font nullfont!
Missing character: There is no C in font nullfont!
Missing character: There is no 2 in font nullfont!
Missing character: There is no 0 in font nullfont!
Missing character: There is no 1 in font nullfont!
Missing character: There is no 8 in font nullfont!
Missing character: There is no ( in font nullfont!
Missing character: There is no N in font nullfont!
Missing character: There is no = in font nullfont!
Missing character: There is no 5 in font nullfont!
Missing character: There is no 0 in font nullfont!
Missing character: There is no 0 in font nullfont!
Missing character: There is no 0 in font nullfont!
Missing character: There is no ) in font nullfont!
Missing character: There is no 0 in font nullfont!
Missing character: There is no . in font nullfont!
Missing character: There is no 0 in font nullfont!
Missing character: There is no 0 in font nullfont!
Missing character: There is no 0 in font nullfont!
Missing character: There is no . in font nullfont!
Missing character: There is no 3 in font nullfont!
Missing character: There is no 0 in font nullfont!
Missing character: There is no 0 in font nullfont!
Missing character: There is no . in font nullfont!
Missing character: There is no 0 in font nullfont!
Missing character: There is no 1 in font nullfont!
Missing character: There is no 0 in font nullfont!
Missing character: There is no . in font nullfont!
Missing character: There is no 4 in font nullfont!
Missing character: There is no 0 in font nullfont!
! Undefined control sequence.
l.24     \multirow
                  [c]{4}{*}{$\widehat{\mu\mathrm{-risk}}_{IPW}$}      &
Twins
The control sequence at the end of the top line
of your error message was never \def'ed. If you have
misspelled it (e.g., `\hobx'), type `I' and the correct
spelling (e.g., `I\hbox'). Otherwise just continue,
and I'll forget about whatever was undefined.

Missing character: There is no [ in font nullfont!
Missing character: There is no c in font nullfont!
Missing character: There is no ] in font nullfont!
Missing character: There is no 4 in font nullfont!
```

Missing character: There is no * in font nullfont!
Missing character: There is no T in font nullfont!
Missing character: There is no w in font nullfont!
Missing character: There is no i in font nullfont!
Missing character: There is no n in font nullfont!
Missing character: There is no s in font nullfont!
Missing character: There is no (in font nullfont!
Missing character: There is no N in font nullfont!
Missing character: There is no = in font nullfont!
Missing character: There is no 1 in font nullfont!
Missing character: There is no 1 in font nullfont!
Missing character: There is no 9 in font nullfont!
Missing character: There is no 8 in font nullfont!
Missing character: There is no 4 in font nullfont!
Missing character: There is no) in font nullfont!
Missing character: There is no - in font nullfont!
Missing character: There is no 0 in font nullfont!
Missing character: There is no . in font nullfont!
Missing character: There is no 3 in font nullfont!
Missing character: There is no 1 in font nullfont!
Missing character: There is no 0 in font nullfont!
Missing character: There is no . in font nullfont!
Missing character: There is no 1 in font nullfont!
Missing character: There is no 3 in font nullfont!
Missing character: There is no - in font nullfont!
Missing character: There is no 0 in font nullfont!
Missing character: There is no . in font nullfont!
Missing character: There is no 1 in font nullfont!
Missing character: There is no 7 in font nullfont!
Missing character: There is no 0 in font nullfont!
Missing character: There is no . in font nullfont!
Missing character: There is no 1 in font nullfont!
Missing character: There is no 2 in font nullfont!
Missing character: There is no A in font nullfont!
Missing character: There is no C in font nullfont!
Missing character: There is no I in font nullfont!
Missing character: There is no C in font nullfont!
Missing character: There is no 2 in font nullfont!
Missing character: There is no 0 in font nullfont!
Missing character: There is no 1 in font nullfont!
Missing character: There is no 6 in font nullfont!
Missing character: There is no (in font nullfont!
Missing character: There is no N in font nullfont!
Missing character: There is no = in font nullfont!
Missing character: There is no 4 in font nullfont!
Missing character: There is no 8 in font nullfont!
Missing character: There is no 0 in font nullfont!
Missing character: There is no 2 in font nullfont!
Missing character: There is no) in font nullfont!
Missing character: There is no - in font nullfont!
Missing character: There is no 0 in font nullfont!
Missing character: There is no . in font nullfont!
Missing character: There is no 0 in font nullfont!
Missing character: There is no 2 in font nullfont!

Missing character: There is no 0 in font nullfont!
Missing character: There is no . in font nullfont!
Missing character: There is no 1 in font nullfont!
Missing character: There is no 3 in font nullfont!
Missing character: There is no 0 in font nullfont!
Missing character: There is no . in font nullfont!
Missing character: There is no 1 in font nullfont!
Missing character: There is no 1 in font nullfont!
Missing character: There is no 0 in font nullfont!
Missing character: There is no . in font nullfont!
Missing character: There is no 1 in font nullfont!
Missing character: There is no 9 in font nullfont!
Missing character: There is no C in font nullfont!
Missing character: There is no a in font nullfont!
Missing character: There is no u in font nullfont!
Missing character: There is no s in font nullfont!
Missing character: There is no s in font nullfont!
Missing character: There is no i in font nullfont!
Missing character: There is no m in font nullfont!
Missing character: There is no (in font nullfont!
Missing character: There is no N in font nullfont!
Missing character: There is no = in font nullfont!
Missing character: There is no 5 in font nullfont!
Missing character: There is no 0 in font nullfont!
Missing character: There is no 0 in font nullfont!
Missing character: There is no 0 in font nullfont!
Missing character: There is no) in font nullfont!
Missing character: There is no - in font nullfont!
Missing character: There is no 0 in font nullfont!
Missing character: There is no . in font nullfont!
Missing character: There is no 3 in font nullfont!
Missing character: There is no 4 in font nullfont!
Missing character: There is no 0 in font nullfont!
Missing character: There is no . in font nullfont!
Missing character: There is no 5 in font nullfont!
Missing character: There is no 0 in font nullfont!
Missing character: There is no 0 in font nullfont!
Missing character: There is no . in font nullfont!
Missing character: There is no 0 in font nullfont!
Missing character: There is no 9 in font nullfont!
Missing character: There is no 0 in font nullfont!
Missing character: There is no . in font nullfont!
Missing character: There is no 3 in font nullfont!
Missing character: There is no 1 in font nullfont!
Missing character: There is no A in font nullfont!
Missing character: There is no C in font nullfont!
Missing character: There is no I in font nullfont!
Missing character: There is no C in font nullfont!
Missing character: There is no 2 in font nullfont!
Missing character: There is no 0 in font nullfont!
Missing character: There is no 1 in font nullfont!
Missing character: There is no 8 in font nullfont!
Missing character: There is no (in font nullfont!
Missing character: There is no N in font nullfont!

```
Missing character: There is no = in font nullfont!
Missing character: There is no 5 in font nullfont!
Missing character: There is no 0 in font nullfont!
Missing character: There is no 0 in font nullfont!
Missing character: There is no 0 in font nullfont!
Missing character: There is no ) in font nullfont!
Missing character: There is no 0 in font nullfont!
Missing character: There is no . in font nullfont!
Missing character: There is no 0 in font nullfont!
Missing character: There is no 0 in font nullfont!
Missing character: There is no 0 in font nullfont!
Missing character: There is no . in font nullfont!
Missing character: There is no 3 in font nullfont!
Missing character: There is no 0 in font nullfont!
Missing character: There is no - in font nullfont!
Missing character: There is no 0 in font nullfont!
Missing character: There is no . in font nullfont!
Missing character: There is no 0 in font nullfont!
Missing character: There is no 1 in font nullfont!
Missing character: There is no 0 in font nullfont!
Missing character: There is no . in font nullfont!
Missing character: There is no 4 in font nullfont!
Missing character: There is no 3 in font nullfont!
! Undefined control sequence.
l.36     \multirow
                  [c]{3}{*}{$\widehat{\mu\mathrm{-risk}}^{*}_{IPW}$}  &
Twins
The control sequence at the end of the top line
of your error message was never \def'ed. If you have
misspelled it (e.g., `\hobx'), type `I' and the correct
spelling (e.g., `I\hbox'). Otherwise just continue,
and I'll forget about whatever was undefined.

Missing character: There is no [ in font nullfont!
Missing character: There is no c in font nullfont!
Missing character: There is no ] in font nullfont!
Missing character: There is no 3 in font nullfont!
Missing character: There is no * in font nullfont!
Missing character: There is no T in font nullfont!
Missing character: There is no w in font nullfont!
Missing character: There is no i in font nullfont!
Missing character: There is no n in font nullfont!
Missing character: There is no s in font nullfont!
Missing character: There is no ( in font nullfont!
Missing character: There is no N in font nullfont!
Missing character: There is no = in font nullfont!
Missing character: There is no 1 in font nullfont!
Missing character: There is no 1 in font nullfont!
Missing character: There is no 9 in font nullfont!
Missing character: There is no 8 in font nullfont!
Missing character: There is no 4 in font nullfont!
Missing character: There is no ) in font nullfont!
Missing character: There is no - in font nullfont!
Missing character: There is no 0 in font nullfont!
```

Missing character: There is no . in font nullfont!
Missing character: There is no 3 in font nullfont!
Missing character: There is no 2 in font nullfont!
Missing character: There is no 0 in font nullfont!
Missing character: There is no . in font nullfont!
Missing character: There is no 1 in font nullfont!
Missing character: There is no 3 in font nullfont!
Missing character: There is no - in font nullfont!
Missing character: There is no 0 in font nullfont!
Missing character: There is no . in font nullfont!
Missing character: There is no 1 in font nullfont!
Missing character: There is no 7 in font nullfont!
Missing character: There is no 0 in font nullfont!
Missing character: There is no . in font nullfont!
Missing character: There is no 1 in font nullfont!
Missing character: There is no 3 in font nullfont!
Missing character: There is no A in font nullfont!
Missing character: There is no C in font nullfont!
Missing character: There is no I in font nullfont!
Missing character: There is no C in font nullfont!
Missing character: There is no 2 in font nullfont!
Missing character: There is no 0 in font nullfont!
Missing character: There is no 1 in font nullfont!
Missing character: There is no 6 in font nullfont!
Missing character: There is no (in font nullfont!
Missing character: There is no N in font nullfont!
Missing character: There is no = in font nullfont!
Missing character: There is no 4 in font nullfont!
Missing character: There is no 8 in font nullfont!
Missing character: There is no 0 in font nullfont!
Missing character: There is no 2 in font nullfont!
Missing character: There is no) in font nullfont!
Missing character: There is no - in font nullfont!
Missing character: There is no 0 in font nullfont!
Missing character: There is no . in font nullfont!
Missing character: There is no 0 in font nullfont!
Missing character: There is no 2 in font nullfont!
Missing character: There is no 0 in font nullfont!
Missing character: There is no . in font nullfont!
Missing character: There is no 1 in font nullfont!
Missing character: There is no 3 in font nullfont!
Missing character: There is no 0 in font nullfont!
Missing character: There is no . in font nullfont!
Missing character: There is no 1 in font nullfont!
Missing character: There is no 1 in font nullfont!
Missing character: There is no 0 in font nullfont!
Missing character: There is no . in font nullfont!
Missing character: There is no 2 in font nullfont!
Missing character: There is no 1 in font nullfont!
Missing character: There is no C in font nullfont!
Missing character: There is no a in font nullfont!
Missing character: There is no u in font nullfont!
Missing character: There is no s in font nullfont!
Missing character: There is no s in font nullfont!

```
Missing character: There is no i in font nullfont!
Missing character: There is no m in font nullfont!
Missing character: There is no ( in font nullfont!
Missing character: There is no N in font nullfont!
Missing character: There is no = in font nullfont!
Missing character: There is no 5 in font nullfont!
Missing character: There is no 0 in font nullfont!
Missing character: There is no 0 in font nullfont!
Missing character: There is no 0 in font nullfont!
Missing character: There is no ) in font nullfont!
Missing character: There is no - in font nullfont!
Missing character: There is no 0 in font nullfont!
Missing character: There is no . in font nullfont!
Missing character: There is no 3 in font nullfont!
Missing character: There is no 3 in font nullfont!
Missing character: There is no 0 in font nullfont!
Missing character: There is no . in font nullfont!
Missing character: There is no 5 in font nullfont!
Missing character: There is no 4 in font nullfont!
Missing character: There is no 0 in font nullfont!
Missing character: There is no . in font nullfont!
Missing character: There is no 2 in font nullfont!
Missing character: There is no 6 in font nullfont!
Missing character: There is no 0 in font nullfont!
Missing character: There is no . in font nullfont!
Missing character: There is no 2 in font nullfont!
Missing character: There is no 7 in font nullfont!
! Undefined control sequence.
l.45     \multirow
                  [c]{4}{*}{$\widehat{\tau\mathrm{-risk}}_{IPW}$}     &
Twins
The control sequence at the end of the top line
of your error message was never \def'ed. If you have
misspelled it (e.g., `\hobx'), type `I' and the correct
spelling (e.g., `I\hbox'). Otherwise just continue,
and I'll forget about whatever was undefined.

Missing character: There is no [ in font nullfont!
Missing character: There is no c in font nullfont!
Missing character: There is no ] in font nullfont!
Missing character: There is no 4 in font nullfont!
Missing character: There is no * in font nullfont!
Missing character: There is no T in font nullfont!
Missing character: There is no w in font nullfont!
Missing character: There is no i in font nullfont!
Missing character: There is no n in font nullfont!
Missing character: There is no s in font nullfont!
Missing character: There is no ( in font nullfont!
Missing character: There is no N in font nullfont!
Missing character: There is no = in font nullfont!
Missing character: There is no 1 in font nullfont!
Missing character: There is no 1 in font nullfont!
Missing character: There is no 9 in font nullfont!
Missing character: There is no 8 in font nullfont!
```

Missing character: There is no 4 in font nullfont!
Missing character: There is no) in font nullfont!
Missing character: There is no 0 in font nullfont!
Missing character: There is no . in font nullfont!
Missing character: There is no 1 in font nullfont!
Missing character: There is no 3 in font nullfont!
Missing character: There is no 0 in font nullfont!
Missing character: There is no . in font nullfont!
Missing character: There is no 1 in font nullfont!
Missing character: There is no 2 in font nullfont!
Missing character: There is no 0 in font nullfont!
Missing character: There is no . in font nullfont!
Missing character: There is no 2 in font nullfont!
Missing character: There is no 7 in font nullfont!
Missing character: There is no 0 in font nullfont!
Missing character: There is no . in font nullfont!
Missing character: There is no 1 in font nullfont!
Missing character: There is no 2 in font nullfont!
Missing character: There is no A in font nullfont!
Missing character: There is no C in font nullfont!
Missing character: There is no I in font nullfont!
Missing character: There is no C in font nullfont!
Missing character: There is no 2 in font nullfont!
Missing character: There is no 0 in font nullfont!
Missing character: There is no 1 in font nullfont!
Missing character: There is no 6 in font nullfont!
Missing character: There is no (in font nullfont!
Missing character: There is no N in font nullfont!
Missing character: There is no = in font nullfont!
Missing character: There is no 4 in font nullfont!
Missing character: There is no 8 in font nullfont!
Missing character: There is no 0 in font nullfont!
Missing character: There is no 2 in font nullfont!
Missing character: There is no) in font nullfont!
Missing character: There is no - in font nullfont!
Missing character: There is no 0 in font nullfont!
Missing character: There is no . in font nullfont!
Missing character: There is no 0 in font nullfont!
Missing character: There is no 7 in font nullfont!
Missing character: There is no 0 in font nullfont!
Missing character: There is no . in font nullfont!
Missing character: There is no 1 in font nullfont!
Missing character: There is no 8 in font nullfont!
Missing character: There is no 0 in font nullfont!
Missing character: There is no . in font nullfont!
Missing character: There is no 0 in font nullfont!
Missing character: There is no 5 in font nullfont!
Missing character: There is no 0 in font nullfont!
Missing character: There is no . in font nullfont!
Missing character: There is no 3 in font nullfont!
Missing character: There is no 1 in font nullfont!
Missing character: There is no C in font nullfont!
Missing character: There is no a in font nullfont!
Missing character: There is no u in font nullfont!

Missing character: There is no s in font nullfont!
Missing character: There is no s in font nullfont!
Missing character: There is no i in font nullfont!
Missing character: There is no m in font nullfont!
Missing character: There is no (in font nullfont!
Missing character: There is no N in font nullfont!
Missing character: There is no = in font nullfont!
Missing character: There is no 5 in font nullfont!
Missing character: There is no 0 in font nullfont!
Missing character: There is no 0 in font nullfont!
Missing character: There is no 0 in font nullfont!
Missing character: There is no) in font nullfont!
Missing character: There is no - in font nullfont!
Missing character: There is no 0 in font nullfont!
Missing character: There is no . in font nullfont!
Missing character: There is no 1 in font nullfont!
Missing character: There is no 9 in font nullfont!
Missing character: There is no 0 in font nullfont!
Missing character: There is no . in font nullfont!
Missing character: There is no 4 in font nullfont!
Missing character: There is no 3 in font nullfont!
Missing character: There is no - in font nullfont!
Missing character: There is no 0 in font nullfont!
Missing character: There is no . in font nullfont!
Missing character: There is no 1 in font nullfont!
Missing character: There is no 4 in font nullfont!
Missing character: There is no 0 in font nullfont!
Missing character: There is no . in font nullfont!
Missing character: There is no 1 in font nullfont!
Missing character: There is no 8 in font nullfont!
Missing character: There is no A in font nullfont!
Missing character: There is no C in font nullfont!
Missing character: There is no I in font nullfont!
Missing character: There is no C in font nullfont!
Missing character: There is no 2 in font nullfont!
Missing character: There is no 0 in font nullfont!
Missing character: There is no 1 in font nullfont!
Missing character: There is no 8 in font nullfont!
Missing character: There is no (in font nullfont!
Missing character: There is no N in font nullfont!
Missing character: There is no = in font nullfont!
Missing character: There is no 5 in font nullfont!
Missing character: There is no 0 in font nullfont!
Missing character: There is no 0 in font nullfont!
Missing character: There is no 0 in font nullfont!
Missing character: There is no) in font nullfont!
Missing character: There is no - in font nullfont!
Missing character: There is no 0 in font nullfont!
Missing character: There is no . in font nullfont!
Missing character: There is no 1 in font nullfont!
Missing character: There is no 6 in font nullfont!
Missing character: There is no 0 in font nullfont!
Missing character: There is no . in font nullfont!
Missing character: There is no 4 in font nullfont!

```
Missing character: There is no 0 in font nullfont!
Missing character: There is no - in font nullfont!
Missing character: There is no 0 in font nullfont!
Missing character: There is no . in font nullfont!
Missing character: There is no 1 in font nullfont!
Missing character: There is no 1 in font nullfont!
Missing character: There is no 0 in font nullfont!
Missing character: There is no . in font nullfont!
Missing character: There is no 6 in font nullfont!
Missing character: There is no 6 in font nullfont!
! Undefined control sequence.
l.57     \multirow
                 [c]{3}{*}{$\widehat{\tau\mathrm{-risk}}_{IPW}^{*}$} &
Twins
The control sequence at the end of the top line
of your error message was never \def'ed. If you have
misspelled it (e.g., `\hobx'), type `I' and the correct
spelling (e.g., `I\hbox'). Otherwise just continue,
and I'll forget about whatever was undefined.

Missing character: There is no [ in font nullfont!
Missing character: There is no c in font nullfont!
Missing character: There is no ] in font nullfont!
Missing character: There is no 3 in font nullfont!
Missing character: There is no * in font nullfont!
Missing character: There is no T in font nullfont!
Missing character: There is no w in font nullfont!
Missing character: There is no i in font nullfont!
Missing character: There is no n in font nullfont!
Missing character: There is no s in font nullfont!
Missing character: There is no ( in font nullfont!
Missing character: There is no N in font nullfont!
Missing character: There is no = in font nullfont!
Missing character: There is no 1 in font nullfont!
Missing character: There is no 1 in font nullfont!
Missing character: There is no 9 in font nullfont!
Missing character: There is no 8 in font nullfont!
Missing character: There is no 4 in font nullfont!
Missing character: There is no ) in font nullfont!
Missing character: There is no 0 in font nullfont!
Missing character: There is no . in font nullfont!
Missing character: There is no 1 in font nullfont!
Missing character: There is no 2 in font nullfont!
Missing character: There is no 0 in font nullfont!
Missing character: There is no . in font nullfont!
Missing character: There is no 1 in font nullfont!
Missing character: There is no 4 in font nullfont!
Missing character: There is no 0 in font nullfont!
Missing character: There is no . in font nullfont!
Missing character: There is no 2 in font nullfont!
Missing character: There is no 0 in font nullfont!
Missing character: There is no 0 in font nullfont!
Missing character: There is no . in font nullfont!
Missing character: There is no 1 in font nullfont!
```

Missing character: There is no 6 in font nullfont!
Missing character: There is no A in font nullfont!
Missing character: There is no C in font nullfont!
Missing character: There is no I in font nullfont!
Missing character: There is no C in font nullfont!
Missing character: There is no 2 in font nullfont!
Missing character: There is no 0 in font nullfont!
Missing character: There is no 1 in font nullfont!
Missing character: There is no 6 in font nullfont!
Missing character: There is no (in font nullfont!
Missing character: There is no N in font nullfont!
Missing character: There is no = in font nullfont!
Missing character: There is no 4 in font nullfont!
Missing character: There is no 8 in font nullfont!
Missing character: There is no 0 in font nullfont!
Missing character: There is no 2 in font nullfont!
Missing character: There is no) in font nullfont!
Missing character: There is no - in font nullfont!
Missing character: There is no 0 in font nullfont!
Missing character: There is no . in font nullfont!
Missing character: There is no 0 in font nullfont!
Missing character: There is no 3 in font nullfont!
Missing character: There is no 0 in font nullfont!
Missing character: There is no . in font nullfont!
Missing character: There is no 1 in font nullfont!
Missing character: There is no 6 in font nullfont!
Missing character: There is no - in font nullfont!
Missing character: There is no 0 in font nullfont!
Missing character: There is no . in font nullfont!
Missing character: There is no 0 in font nullfont!
Missing character: There is no 9 in font nullfont!
Missing character: There is no 0 in font nullfont!
Missing character: There is no . in font nullfont!
Missing character: There is no 4 in font nullfont!
Missing character: There is no 3 in font nullfont!
Missing character: There is no C in font nullfont!
Missing character: There is no a in font nullfont!
Missing character: There is no u in font nullfont!
Missing character: There is no s in font nullfont!
Missing character: There is no s in font nullfont!
Missing character: There is no i in font nullfont!
Missing character: There is no m in font nullfont!
Missing character: There is no (in font nullfont!
Missing character: There is no N in font nullfont!
Missing character: There is no = in font nullfont!
Missing character: There is no 5 in font nullfont!
Missing character: There is no 0 in font nullfont!
Missing character: There is no 0 in font nullfont!
Missing character: There is no 0 in font nullfont!
Missing character: There is no) in font nullfont!
Missing character: There is no - in font nullfont!
Missing character: There is no 0 in font nullfont!
Missing character: There is no . in font nullfont!
Missing character: There is no 1 in font nullfont!

```
Missing character: There is no 5 in font nullfont!
Missing character: There is no 0 in font nullfont!
Missing character: There is no . in font nullfont!
Missing character: There is no 4 in font nullfont!
Missing character: There is no 6 in font nullfont!
Missing character: There is no - in font nullfont!
Missing character: There is no 0 in font nullfont!
Missing character: There is no . in font nullfont!
Missing character: There is no 1 in font nullfont!
Missing character: There is no 7 in font nullfont!
Missing character: There is no 0 in font nullfont!
Missing character: There is no . in font nullfont!
Missing character: There is no 1 in font nullfont!
Missing character: There is no 9 in font nullfont!
! Undefined control sequence.
l.66     \multirow
                  [c]{4}{*}{$\widehat{\mathrm{U-risk}}$}              &
Twins
The control sequence at the end of the top line
of your error message was never \def'ed. If you have
misspelled it (e.g., `\hobx'), type `I' and the correct
spelling (e.g., `I\hbox'). Otherwise just continue,
and I'll forget about whatever was undefined.

Missing character: There is no [ in font nullfont!
Missing character: There is no c in font nullfont!
Missing character: There is no ] in font nullfont!
Missing character: There is no 4 in font nullfont!
Missing character: There is no * in font nullfont!
Missing character: There is no T in font nullfont!
Missing character: There is no w in font nullfont!
Missing character: There is no i in font nullfont!
Missing character: There is no n in font nullfont!
Missing character: There is no s in font nullfont!
Missing character: There is no ( in font nullfont!
Missing character: There is no N in font nullfont!
Missing character: There is no = in font nullfont!
Missing character: There is no 1 in font nullfont!
Missing character: There is no 1 in font nullfont!
Missing character: There is no 9 in font nullfont!
Missing character: There is no 8 in font nullfont!
Missing character: There is no 4 in font nullfont!
Missing character: There is no ) in font nullfont!
Missing character: There is no 0 in font nullfont!
Missing character: There is no . in font nullfont!
Missing character: There is no 1 in font nullfont!
Missing character: There is no 3 in font nullfont!
Missing character: There is no 0 in font nullfont!
Missing character: There is no . in font nullfont!
Missing character: There is no 1 in font nullfont!
Missing character: There is no 2 in font nullfont!
Missing character: There is no 0 in font nullfont!
Missing character: There is no . in font nullfont!
Missing character: There is no 0 in font nullfont!
```

Missing character: There is no 2 in font nullfont!
Missing character: There is no 0 in font nullfont!
Missing character: There is no . in font nullfont!
Missing character: There is no 2 in font nullfont!
Missing character: There is no 5 in font nullfont!
Missing character: There is no A in font nullfont!
Missing character: There is no C in font nullfont!
Missing character: There is no I in font nullfont!
Missing character: There is no C in font nullfont!
Missing character: There is no 2 in font nullfont!
Missing character: There is no 0 in font nullfont!
Missing character: There is no 1 in font nullfont!
Missing character: There is no 6 in font nullfont!
Missing character: There is no (in font nullfont!
Missing character: There is no N in font nullfont!
Missing character: There is no = in font nullfont!
Missing character: There is no 4 in font nullfont!
Missing character: There is no 8 in font nullfont!
Missing character: There is no 0 in font nullfont!
Missing character: There is no 2 in font nullfont!
Missing character: There is no) in font nullfont!
Missing character: There is no 0 in font nullfont!
Missing character: There is no . in font nullfont!
Missing character: There is no 0 in font nullfont!
Missing character: There is no 4 in font nullfont!
Missing character: There is no 0 in font nullfont!
Missing character: There is no . in font nullfont!
Missing character: There is no 1 in font nullfont!
Missing character: There is no 1 in font nullfont!
Missing character: There is no 0 in font nullfont!
Missing character: There is no . in font nullfont!
Missing character: There is no 1 in font nullfont!
Missing character: There is no 1 in font nullfont!
Missing character: There is no 0 in font nullfont!
Missing character: There is no . in font nullfont!
Missing character: There is no 2 in font nullfont!
Missing character: There is no 6 in font nullfont!
Missing character: There is no C in font nullfont!
Missing character: There is no a in font nullfont!
Missing character: There is no u in font nullfont!
Missing character: There is no s in font nullfont!
Missing character: There is no s in font nullfont!
Missing character: There is no i in font nullfont!
Missing character: There is no m in font nullfont!
Missing character: There is no (in font nullfont!
Missing character: There is no N in font nullfont!
Missing character: There is no = in font nullfont!
Missing character: There is no 5 in font nullfont!
Missing character: There is no 0 in font nullfont!
Missing character: There is no 0 in font nullfont!
Missing character: There is no 0 in font nullfont!
Missing character: There is no) in font nullfont!
Missing character: There is no 0 in font nullfont!
Missing character: There is no . in font nullfont!

```
Missing character: There is no 0 in font nullfont!
Missing character: There is no 4 in font nullfont!
Missing character: There is no 0 in font nullfont!
Missing character: There is no . in font nullfont!
Missing character: There is no 4 in font nullfont!
Missing character: There is no 3 in font nullfont!
Missing character: There is no - in font nullfont!
Missing character: There is no 0 in font nullfont!
Missing character: There is no . in font nullfont!
Missing character: There is no 0 in font nullfont!
Missing character: There is no 4 in font nullfont!
Missing character: There is no 0 in font nullfont!
Missing character: There is no . in font nullfont!
Missing character: There is no 1 in font nullfont!
Missing character: There is no 7 in font nullfont!
Missing character: There is no A in font nullfont!
Missing character: There is no C in font nullfont!
Missing character: There is no I in font nullfont!
Missing character: There is no C in font nullfont!
Missing character: There is no 2 in font nullfont!
Missing character: There is no 0 in font nullfont!
Missing character: There is no 1 in font nullfont!
Missing character: There is no 8 in font nullfont!
Missing character: There is no ( in font nullfont!
Missing character: There is no N in font nullfont!
Missing character: There is no = in font nullfont!
Missing character: There is no 5 in font nullfont!
Missing character: There is no 0 in font nullfont!
Missing character: There is no 0 in font nullfont!
Missing character: There is no 0 in font nullfont!
Missing character: There is no ) in font nullfont!
Missing character: There is no 0 in font nullfont!
Missing character: There is no . in font nullfont!
Missing character: There is no 1 in font nullfont!
Missing character: There is no 2 in font nullfont!
Missing character: There is no 0 in font nullfont!
Missing character: There is no . in font nullfont!
Missing character: There is no 2 in font nullfont!
Missing character: There is no 6 in font nullfont!
Missing character: There is no - in font nullfont!
Missing character: There is no 0 in font nullfont!
Missing character: There is no . in font nullfont!
Missing character: There is no 0 in font nullfont!
Missing character: There is no 2 in font nullfont!
Missing character: There is no 0 in font nullfont!
Missing character: There is no . in font nullfont!
Missing character: There is no 5 in font nullfont!
Missing character: There is no 0 in font nullfont!
! Undefined control sequence.
l.78     \multirow
                  [c]{3}{*}{$\widehat{\mathrm{U-risk}}^{*}$}          &
Twins
The control sequence at the end of the top line
of your error message was never \def'ed. If you have
```

```
misspelled it (e.g., `\hobx'), type `I' and the correct
spelling (e.g., `I\hbox'). Otherwise just continue,
and I'll forget about whatever was undefined.

Missing character: There is no [ in font nullfont!
Missing character: There is no c in font nullfont!
Missing character: There is no ] in font nullfont!
Missing character: There is no 3 in font nullfont!
Missing character: There is no * in font nullfont!
Missing character: There is no T in font nullfont!
Missing character: There is no w in font nullfont!
Missing character: There is no i in font nullfont!
Missing character: There is no n in font nullfont!
Missing character: There is no s in font nullfont!
Missing character: There is no ( in font nullfont!
Missing character: There is no N in font nullfont!
Missing character: There is no = in font nullfont!
Missing character: There is no 1 in font nullfont!
Missing character: There is no 1 in font nullfont!
Missing character: There is no 9 in font nullfont!
Missing character: There is no 8 in font nullfont!
Missing character: There is no 4 in font nullfont!
Missing character: There is no ) in font nullfont!
Missing character: There is no 0 in font nullfont!
Missing character: There is no . in font nullfont!
Missing character: There is no 2 in font nullfont!
Missing character: There is no 5 in font nullfont!
Missing character: There is no 0 in font nullfont!
Missing character: There is no . in font nullfont!
Missing character: There is no 0 in font nullfont!
Missing character: There is no 8 in font nullfont!
Missing character: There is no - in font nullfont!
Missing character: There is no 0 in font nullfont!
Missing character: There is no . in font nullfont!
Missing character: There is no 4 in font nullfont!
Missing character: There is no 1 in font nullfont!
Missing character: There is no 0 in font nullfont!
Missing character: There is no . in font nullfont!
Missing character: There is no 4 in font nullfont!
Missing character: There is no 5 in font nullfont!
Missing character: There is no A in font nullfont!
Missing character: There is no C in font nullfont!
Missing character: There is no I in font nullfont!
Missing character: There is no C in font nullfont!
Missing character: There is no 2 in font nullfont!
Missing character: There is no 0 in font nullfont!
Missing character: There is no 1 in font nullfont!
Missing character: There is no 6 in font nullfont!
Missing character: There is no ( in font nullfont!
Missing character: There is no N in font nullfont!
Missing character: There is no = in font nullfont!
Missing character: There is no 4 in font nullfont!
Missing character: There is no 8 in font nullfont!
Missing character: There is no 0 in font nullfont!
```

```
Missing character: There is no 2 in font nullfont!
Missing character: There is no ) in font nullfont!
Missing character: There is no 0 in font nullfont!
Missing character: There is no . in font nullfont!
Missing character: There is no 0 in font nullfont!
Missing character: There is no 8 in font nullfont!
Missing character: There is no 0 in font nullfont!
Missing character: There is no . in font nullfont!
Missing character: There is no 1 in font nullfont!
Missing character: There is no 3 in font nullfont!
Missing character: There is no - in font nullfont!
Missing character: There is no 0 in font nullfont!
Missing character: There is no . in font nullfont!
Missing character: There is no 5 in font nullfont!
Missing character: There is no 9 in font nullfont!
Missing character: There is no 0 in font nullfont!
Missing character: There is no . in font nullfont!
Missing character: There is no 5 in font nullfont!
Missing character: There is no 7 in font nullfont!
Missing character: There is no C in font nullfont!
Missing character: There is no a in font nullfont!
Missing character: There is no u in font nullfont!
Missing character: There is no s in font nullfont!
Missing character: There is no s in font nullfont!
Missing character: There is no i in font nullfont!
Missing character: There is no m in font nullfont!
Missing character: There is no ( in font nullfont!
Missing character: There is no N in font nullfont!
Missing character: There is no = in font nullfont!
Missing character: There is no 5 in font nullfont!
Missing character: There is no 0 in font nullfont!
Missing character: There is no 0 in font nullfont!
Missing character: There is no 0 in font nullfont!
Missing character: There is no ) in font nullfont!
Missing character: There is no 0 in font nullfont!
Missing character: There is no . in font nullfont!
Missing character: There is no 4 in font nullfont!
Missing character: There is no 6 in font nullfont!
Missing character: There is no 0 in font nullfont!
Missing character: There is no . in font nullfont!
Missing character: There is no 1 in font nullfont!
Missing character: There is no 2 in font nullfont!
Missing character: There is no 0 in font nullfont!
Missing character: There is no . in font nullfont!
Missing character: There is no 0 in font nullfont!
Missing character: There is no 2 in font nullfont!
Missing character: There is no 0 in font nullfont!
Missing character: There is no . in font nullfont!
Missing character: There is no 4 in font nullfont!
Missing character: There is no 4 in font nullfont!
! Undefined control sequence.
l.87     \multirow
                  [c]{4}{*}{$\widehat{\mathrm{R-risk}}$}              &
Twins
```

```
The control sequence at the end of the top line
of your error message was never \def'ed. If you have
misspelled it (e.g., `\hobx'), type `I' and the correct
spelling (e.g., `I\hbox'). Otherwise just continue,
and I'll forget about whatever was undefined.

Missing character: There is no [ in font nullfont!
Missing character: There is no c in font nullfont!
Missing character: There is no ] in font nullfont!
Missing character: There is no 4 in font nullfont!
Missing character: There is no * in font nullfont!
Missing character: There is no T in font nullfont!
Missing character: There is no w in font nullfont!
Missing character: There is no i in font nullfont!
Missing character: There is no n in font nullfont!
Missing character: There is no s in font nullfont!
Missing character: There is no ( in font nullfont!
Missing character: There is no N in font nullfont!
Missing character: There is no = in font nullfont!
Missing character: There is no 1 in font nullfont!
Missing character: There is no 1 in font nullfont!
Missing character: There is no 9 in font nullfont!
Missing character: There is no 8 in font nullfont!
Missing character: There is no 4 in font nullfont!
Missing character: There is no ) in font nullfont!
Missing character: There is no 0 in font nullfont!
Missing character: There is no . in font nullfont!
Missing character: There is no 1 in font nullfont!
Missing character: There is no 5 in font nullfont!
Missing character: There is no 0 in font nullfont!
Missing character: There is no . in font nullfont!
Missing character: There is no 1 in font nullfont!
Missing character: There is no 0 in font nullfont!
Missing character: There is no 0 in font nullfont!
Missing character: There is no . in font nullfont!
Missing character: There is no 2 in font nullfont!
Missing character: There is no 5 in font nullfont!
Missing character: There is no 0 in font nullfont!
Missing character: There is no . in font nullfont!
Missing character: There is no 1 in font nullfont!
Missing character: There is no 8 in font nullfont!
Missing character: There is no A in font nullfont!
Missing character: There is no C in font nullfont!
Missing character: There is no I in font nullfont!
Missing character: There is no C in font nullfont!
Missing character: There is no 2 in font nullfont!
Missing character: There is no 0 in font nullfont!
Missing character: There is no 1 in font nullfont!
Missing character: There is no 6 in font nullfont!
Missing character: There is no ( in font nullfont!
Missing character: There is no N in font nullfont!
Missing character: There is no = in font nullfont!
Missing character: There is no 4 in font nullfont!
Missing character: There is no 8 in font nullfont!
```

Missing character: There is no 0 in font nullfont!
Missing character: There is no 2 in font nullfont!
Missing character: There is no) in font nullfont!
Missing character: There is no 0 in font nullfont!
Missing character: There is no . in font nullfont!
Missing character: There is no 0 in font nullfont!
Missing character: There is no 7 in font nullfont!
Missing character: There is no 0 in font nullfont!
Missing character: There is no . in font nullfont!
Missing character: There is no 1 in font nullfont!
Missing character: There is no 2 in font nullfont!
Missing character: There is no 0 in font nullfont!
Missing character: There is no . in font nullfont!
Missing character: There is no 2 in font nullfont!
Missing character: There is no 2 in font nullfont!
Missing character: There is no 0 in font nullfont!
Missing character: There is no . in font nullfont!
Missing character: There is no 1 in font nullfont!
Missing character: There is no 5 in font nullfont!
Missing character: There is no C in font nullfont!
Missing character: There is no a in font nullfont!
Missing character: There is no u in font nullfont!
Missing character: There is no s in font nullfont!
Missing character: There is no s in font nullfont!
Missing character: There is no i in font nullfont!
Missing character: There is no m in font nullfont!
Missing character: There is no (in font nullfont!
Missing character: There is no N in font nullfont!
Missing character: There is no = in font nullfont!
Missing character: There is no 5 in font nullfont!
Missing character: There is no 0 in font nullfont!
Missing character: There is no 0 in font nullfont!
Missing character: There is no 0 in font nullfont!
Missing character: There is no) in font nullfont!
Missing character: There is no 0 in font nullfont!
Missing character: There is no . in font nullfont!
Missing character: There is no 3 in font nullfont!
Missing character: There is no 4 in font nullfont!
Missing character: There is no 0 in font nullfont!
Missing character: There is no . in font nullfont!
Missing character: There is no 2 in font nullfont!
Missing character: There is no 6 in font nullfont!
Missing character: There is no 0 in font nullfont!
Missing character: There is no . in font nullfont!
Missing character: There is no 1 in font nullfont!
Missing character: There is no 3 in font nullfont!
Missing character: There is no 0 in font nullfont!
Missing character: There is no . in font nullfont!
Missing character: There is no 2 in font nullfont!
Missing character: There is no 1 in font nullfont!
Missing character: There is no A in font nullfont!
Missing character: There is no C in font nullfont!
Missing character: There is no I in font nullfont!
Missing character: There is no C in font nullfont!

```
Missing character: There is no 2 in font nullfont!
Missing character: There is no 0 in font nullfont!
Missing character: There is no 1 in font nullfont!
Missing character: There is no 8 in font nullfont!
Missing character: There is no ( in font nullfont!
Missing character: There is no N in font nullfont!
Missing character: There is no = in font nullfont!
Missing character: There is no 5 in font nullfont!
Missing character: There is no 0 in font nullfont!
Missing character: There is no 0 in font nullfont!
Missing character: There is no 0 in font nullfont!
Missing character: There is no ) in font nullfont!
Missing character: There is no 0 in font nullfont!
Missing character: There is no . in font nullfont!
Missing character: There is no 1 in font nullfont!
Missing character: There is no 3 in font nullfont!
Missing character: There is no 0 in font nullfont!
Missing character: There is no . in font nullfont!
Missing character: There is no 2 in font nullfont!
Missing character: There is no 7 in font nullfont!
Missing character: There is no 0 in font nullfont!
Missing character: There is no . in font nullfont!
Missing character: There is no 2 in font nullfont!
Missing character: There is no 1 in font nullfont!
Missing character: There is no 0 in font nullfont!
Missing character: There is no . in font nullfont!
Missing character: There is no 4 in font nullfont!
Missing character: There is no 7 in font nullfont!
! Undefined control sequence.
l.99     \multirow
                  [c]{3}{*}{$\widehat{\mathrm{R-risk}}^{*}$}          &
Twins
The control sequence at the end of the top line
of your error message was never \def'ed. If you have
misspelled it (e.g., `\hobx'), type `I' and the correct
spelling (e.g., `I\hbox'). Otherwise just continue,
and I'll forget about whatever was undefined.

Missing character: There is no [ in font nullfont!
Missing character: There is no c in font nullfont!
Missing character: There is no ] in font nullfont!
Missing character: There is no 3 in font nullfont!
Missing character: There is no * in font nullfont!
Missing character: There is no T in font nullfont!
Missing character: There is no w in font nullfont!
Missing character: There is no i in font nullfont!
Missing character: There is no n in font nullfont!
Missing character: There is no s in font nullfont!
Missing character: There is no ( in font nullfont!
Missing character: There is no N in font nullfont!
Missing character: There is no = in font nullfont!
Missing character: There is no 1 in font nullfont!
Missing character: There is no 1 in font nullfont!
Missing character: There is no 9 in font nullfont!
```

Missing character: There is no 8 in font nullfont!
Missing character: There is no 4 in font nullfont!
Missing character: There is no) in font nullfont!
Missing character: There is no 0 in font nullfont!
Missing character: There is no . in font nullfont!
Missing character: There is no 2 in font nullfont!
Missing character: There is no 5 in font nullfont!
Missing character: There is no 0 in font nullfont!
Missing character: There is no . in font nullfont!
Missing character: There is no 1 in font nullfont!
Missing character: There is no 0 in font nullfont!
Missing character: There is no 0 in font nullfont!
Missing character: There is no . in font nullfont!
Missing character: There is no 3 in font nullfont!
Missing character: There is no 2 in font nullfont!
Missing character: There is no 0 in font nullfont!
Missing character: There is no . in font nullfont!
Missing character: There is no 1 in font nullfont!
Missing character: There is no 5 in font nullfont!
Missing character: There is no A in font nullfont!
Missing character: There is no C in font nullfont!
Missing character: There is no I in font nullfont!
Missing character: There is no C in font nullfont!
Missing character: There is no 2 in font nullfont!
Missing character: There is no 0 in font nullfont!
Missing character: There is no 1 in font nullfont!
Missing character: There is no 6 in font nullfont!
Missing character: There is no (in font nullfont!
Missing character: There is no N in font nullfont!
Missing character: There is no = in font nullfont!
Missing character: There is no 4 in font nullfont!
Missing character: There is no 8 in font nullfont!
Missing character: There is no 0 in font nullfont!
Missing character: There is no 2 in font nullfont!
Missing character: There is no) in font nullfont!
Missing character: There is no 0 in font nullfont!
Missing character: There is no . in font nullfont!
Missing character: There is no 1 in font nullfont!
Missing character: There is no 2 in font nullfont!
Missing character: There is no 0 in font nullfont!
Missing character: There is no . in font nullfont!
Missing character: There is no 1 in font nullfont!
Missing character: There is no 2 in font nullfont!
Missing character: There is no 0 in font nullfont!
Missing character: There is no . in font nullfont!
Missing character: There is no 2 in font nullfont!
Missing character: There is no 5 in font nullfont!
Missing character: There is no 0 in font nullfont!
Missing character: There is no . in font nullfont!
Missing character: There is no 1 in font nullfont!
Missing character: There is no 5 in font nullfont!
Missing character: There is no C in font nullfont!
Missing character: There is no a in font nullfont!
Missing character: There is no u in font nullfont!

```
Missing character: There is no s in font nullfont!
Missing character: There is no s in font nullfont!
Missing character: There is no i in font nullfont!
Missing character: There is no m in font nullfont!
Missing character: There is no ( in font nullfont!
Missing character: There is no N in font nullfont!
Missing character: There is no = in font nullfont!
Missing character: There is no 5 in font nullfont!
Missing character: There is no 0 in font nullfont!
Missing character: There is no 0 in font nullfont!
Missing character: There is no 0 in font nullfont!
Missing character: There is no ) in font nullfont!
Missing character: There is no 0 in font nullfont!
Missing character: There is no . in font nullfont!
Missing character: There is no 4 in font nullfont!
Missing character: There is no 7 in font nullfont!
Missing character: There is no 0 in font nullfont!
Missing character: There is no . in font nullfont!
Missing character: There is no 1 in font nullfont!
Missing character: There is no 1 in font nullfont!
Missing character: There is no 0 in font nullfont!
Missing character: There is no . in font nullfont!
Missing character: There is no 1 in font nullfont!
Missing character: There is no 6 in font nullfont!
Missing character: There is no 0 in font nullfont!
Missing character: There is no . in font nullfont!
Missing character: There is no 1 in font nullfont!
Missing character: There is no 4 in font nullfont!
! Undefined control sequence.
l.108     \bottomrule

The control sequence at the end of the top line
of your error message was never \def'ed. If you have
misspelled it (e.g., `\hobx'), type `I' and the correct
spelling (e.g., `I\hbox'). Otherwise just continue,
and I'll forget about whatever was undefined.

)
! Emergency stop.
<*> _1_r_risk_domination_boxplot_df.tex

*** (job aborted, no legal \end found)


Here is how much of TeX's memory you used:
 22 strings out of 473583
 558 string characters out of 5732343
 1940908 words of memory out of 5000000
 23015 multiletter control sequences out of 15000+600000
 558069 words of font info for 36 fonts, out of 8000000 for 9000
 1141 hyphenation exceptions out of 8191
 19i,9n,18p,149b,111s stack positions out of
10000i,1000n,20000p,200000b,200000s
!  ==> Fatal error occurred, no output PDF file produced!
```

Click here to access/download;Figure;acic_2016_ate_hte.pdf

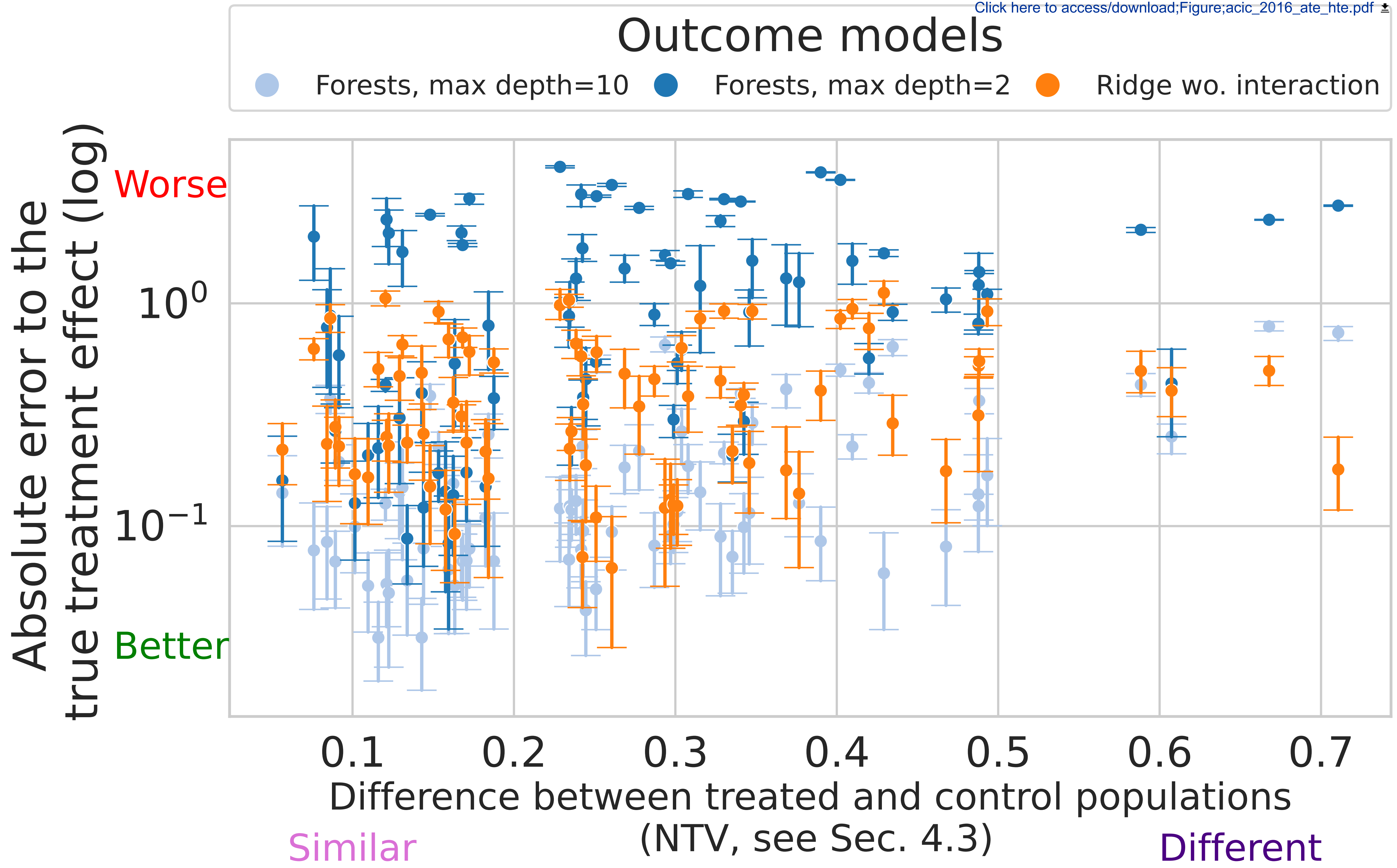

Click here to access/download;Figure;_2_overlap_influence_by_bin_k_by_ds_r_risk_2cols.pdf

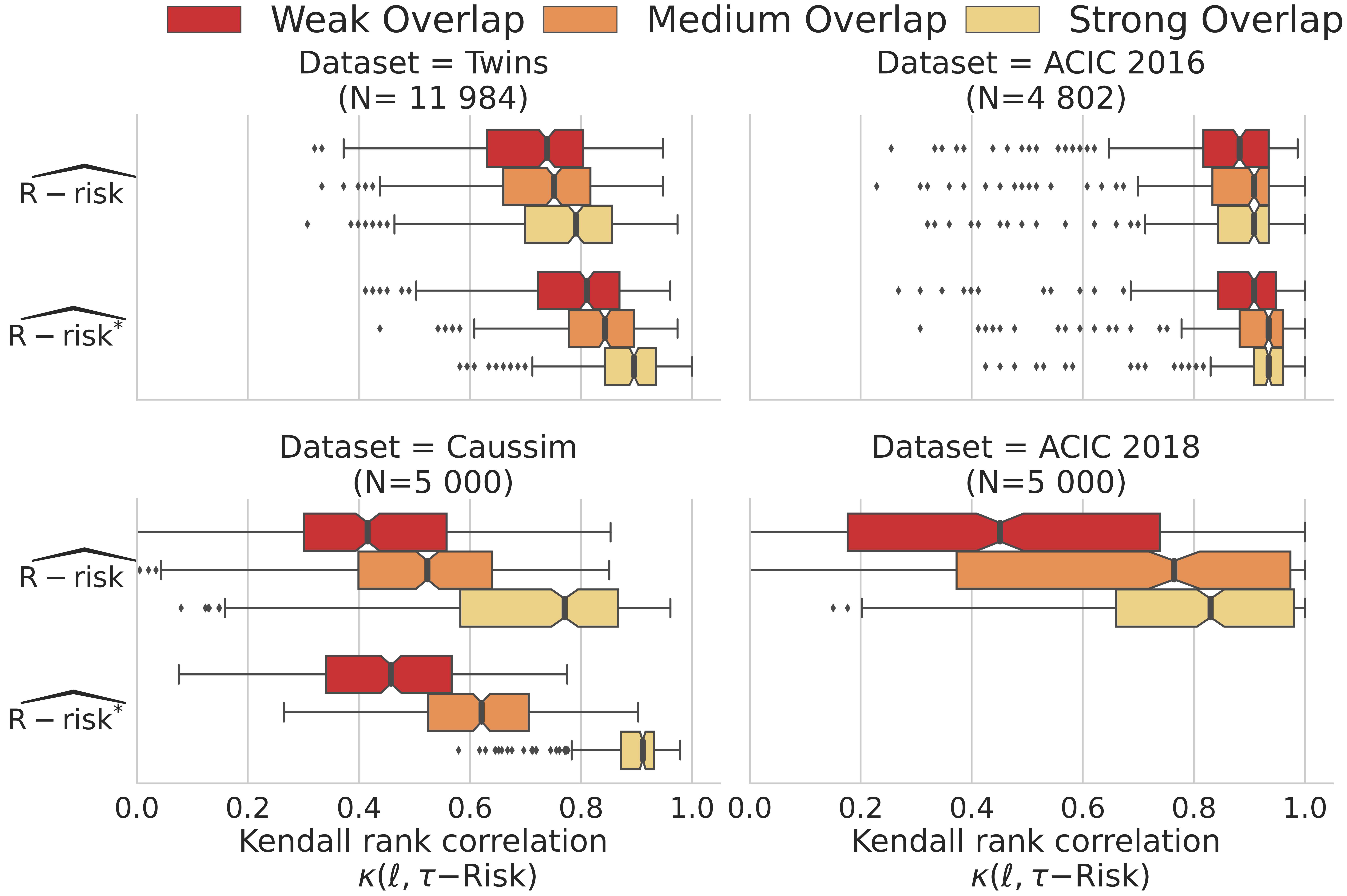

Click here to access/download;Figure;ktau_acic2016_nuisance_nlin_models_hgb_dgp1-77_rs1-10.pdf

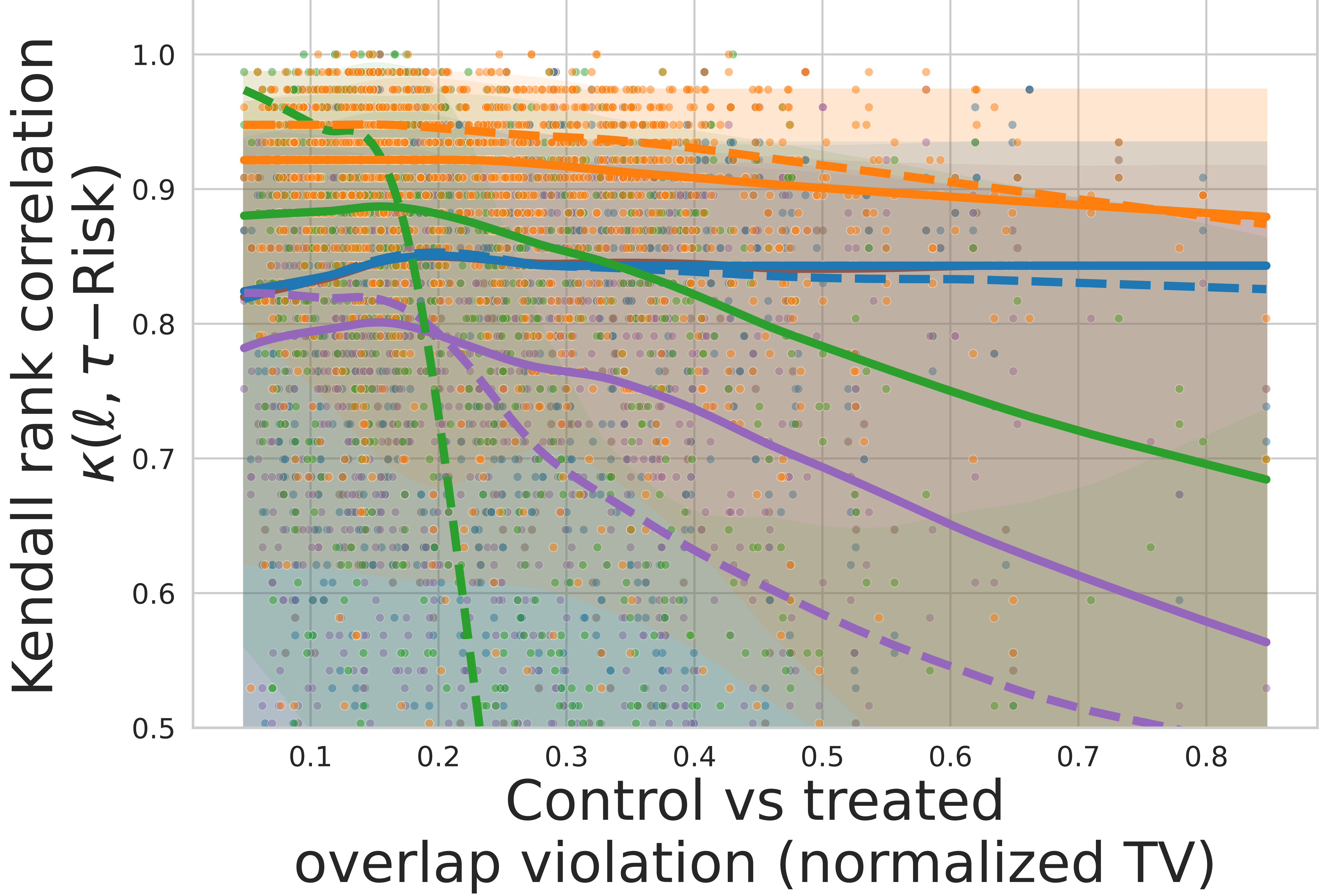

Click here to access/download;Figure;norm_bias_trisk_tobest_acic2016_nuis_nlin_models_hgb.pdf

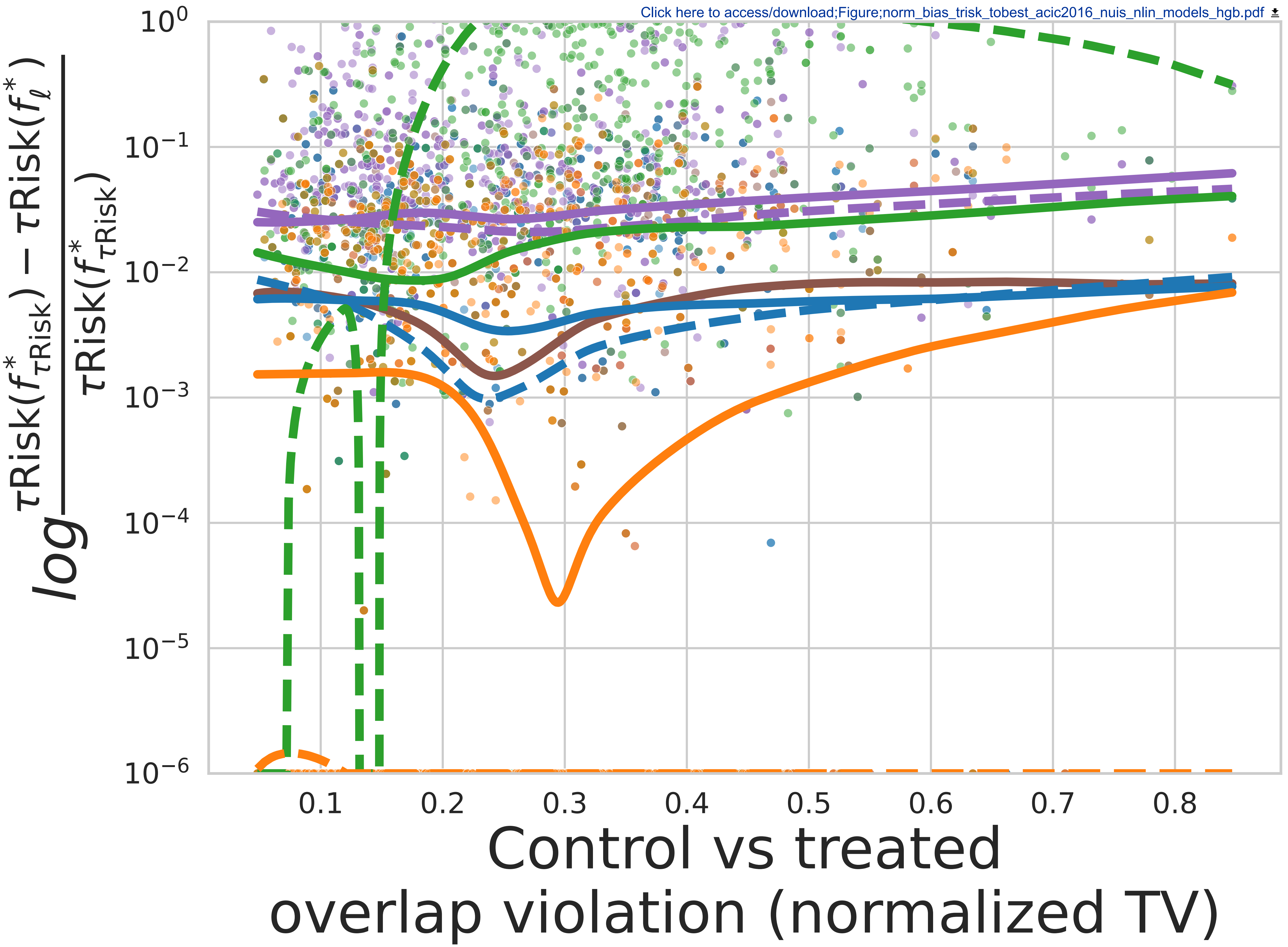

Click here to
access/download;Figure;legend_m

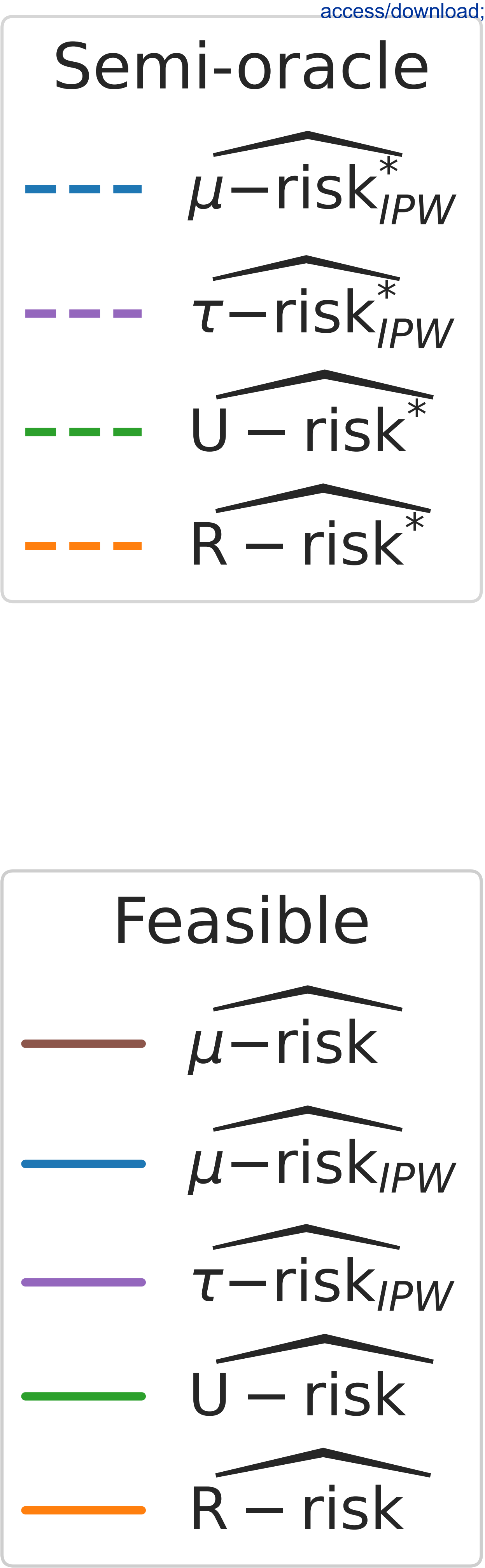

Click here to access/download;Figure;_3_procedure_r_risk_only_3datasets.pdf

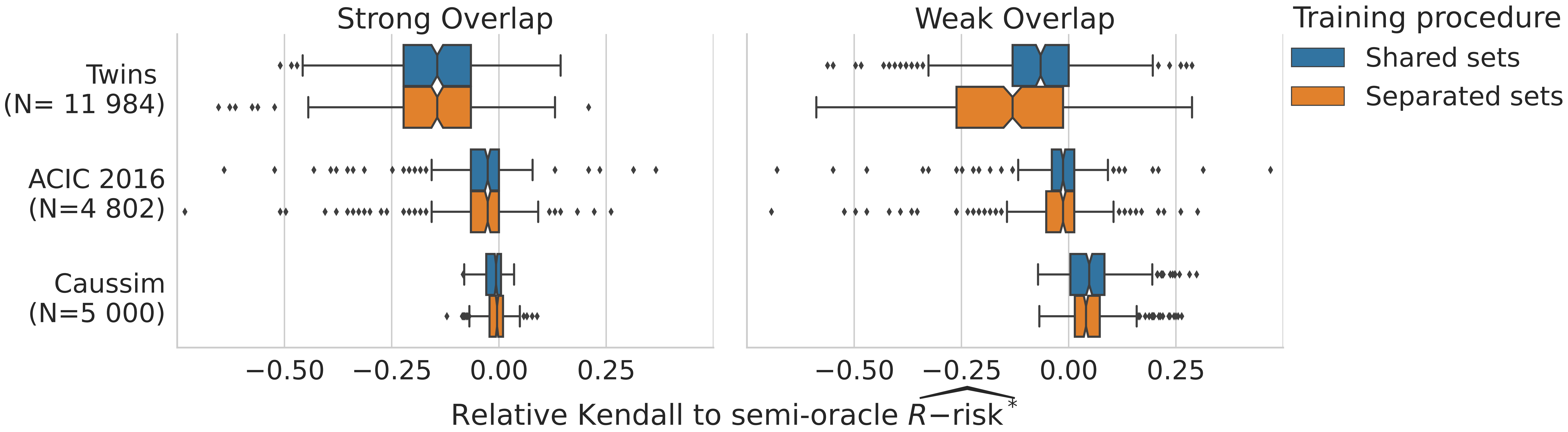

Click here to access/download;Figure;toy_tlinear_model_small_R2_small_tau_risk.pdf

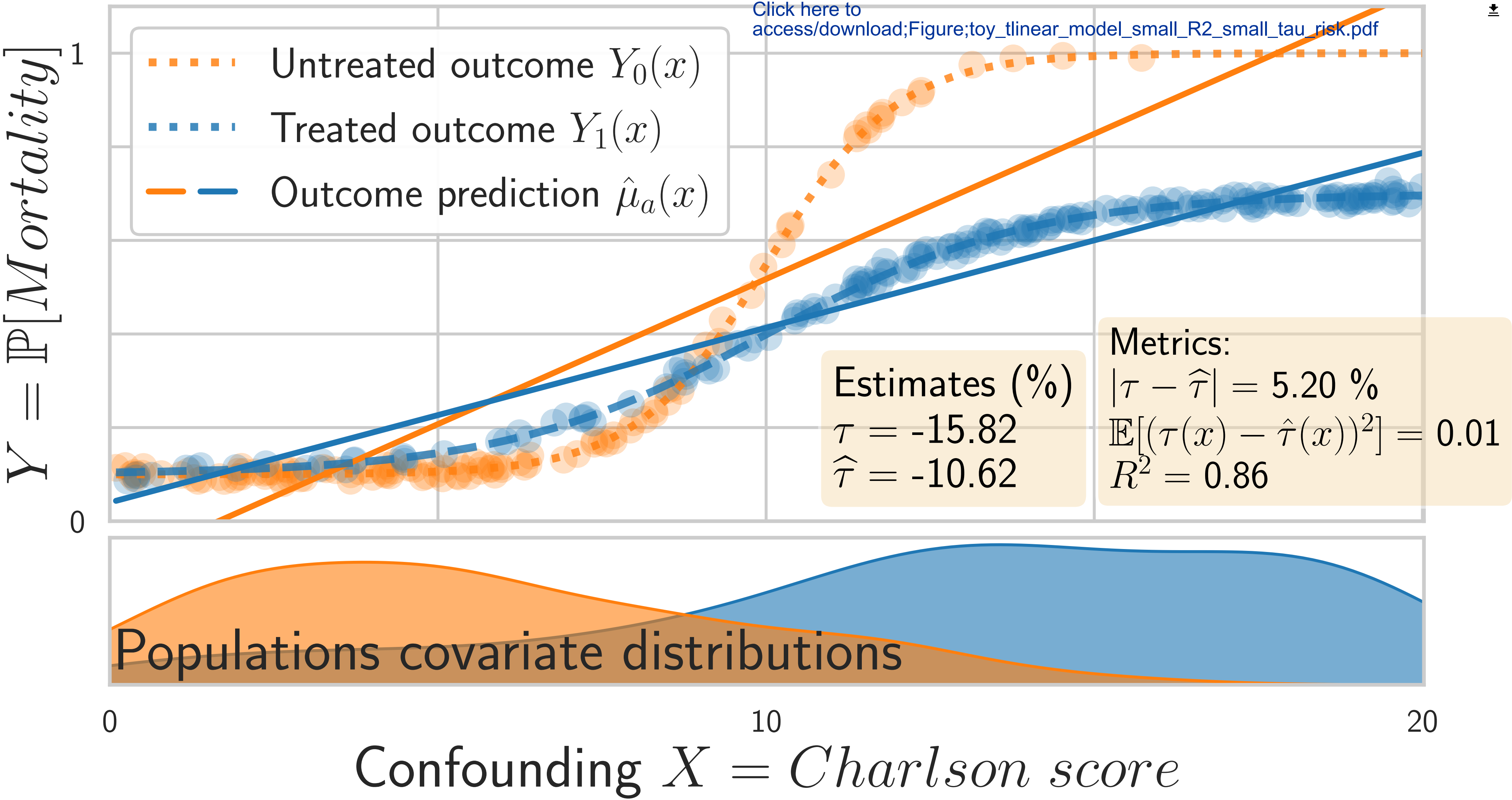

Click here to access/download;Figure;ktau_twins_nuisance_stacking__models_hgb.pdf

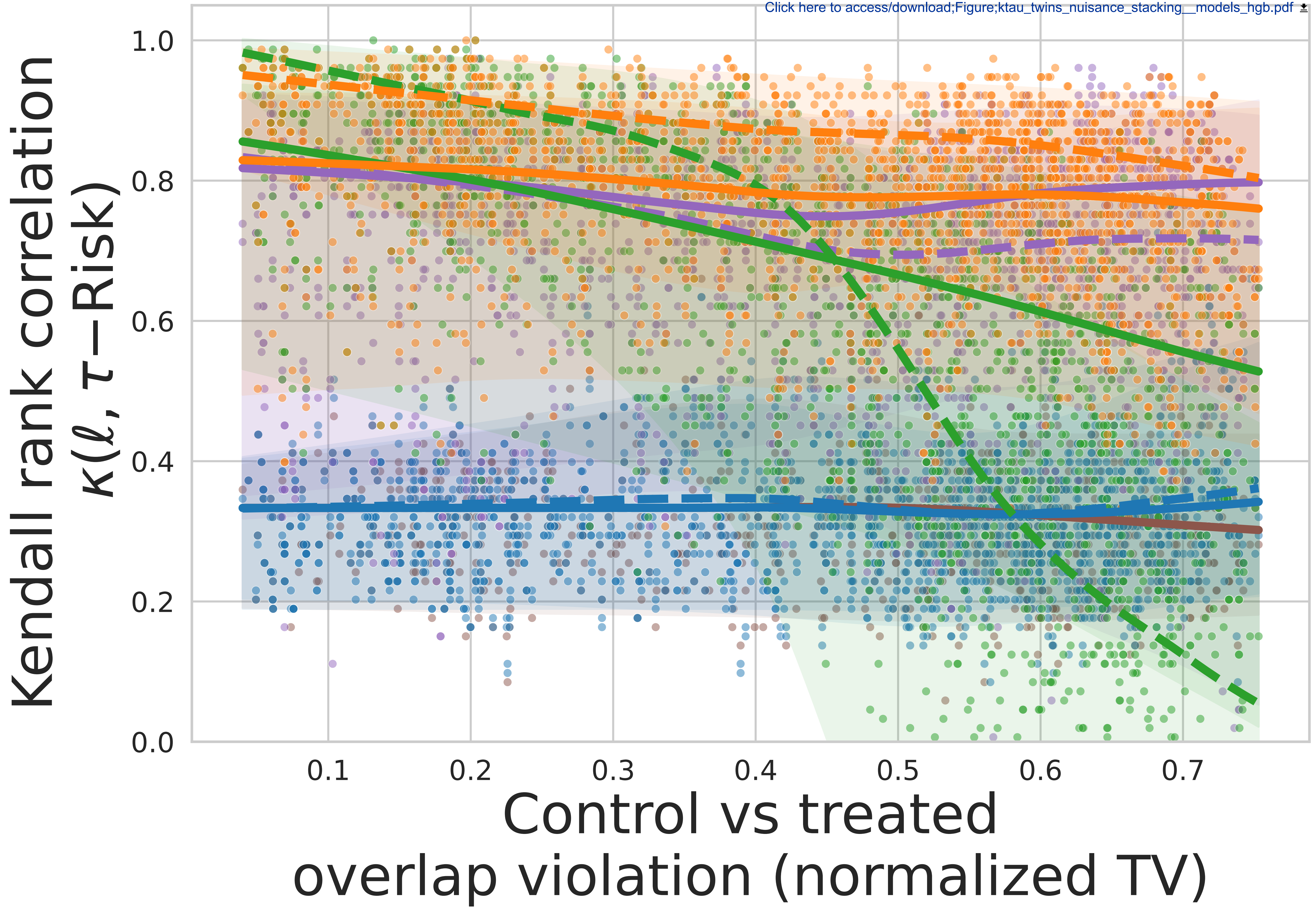

Click here to access/download;Figure;_3_procedure_Twins_N11984__ref_oracle_r_risk.pdf

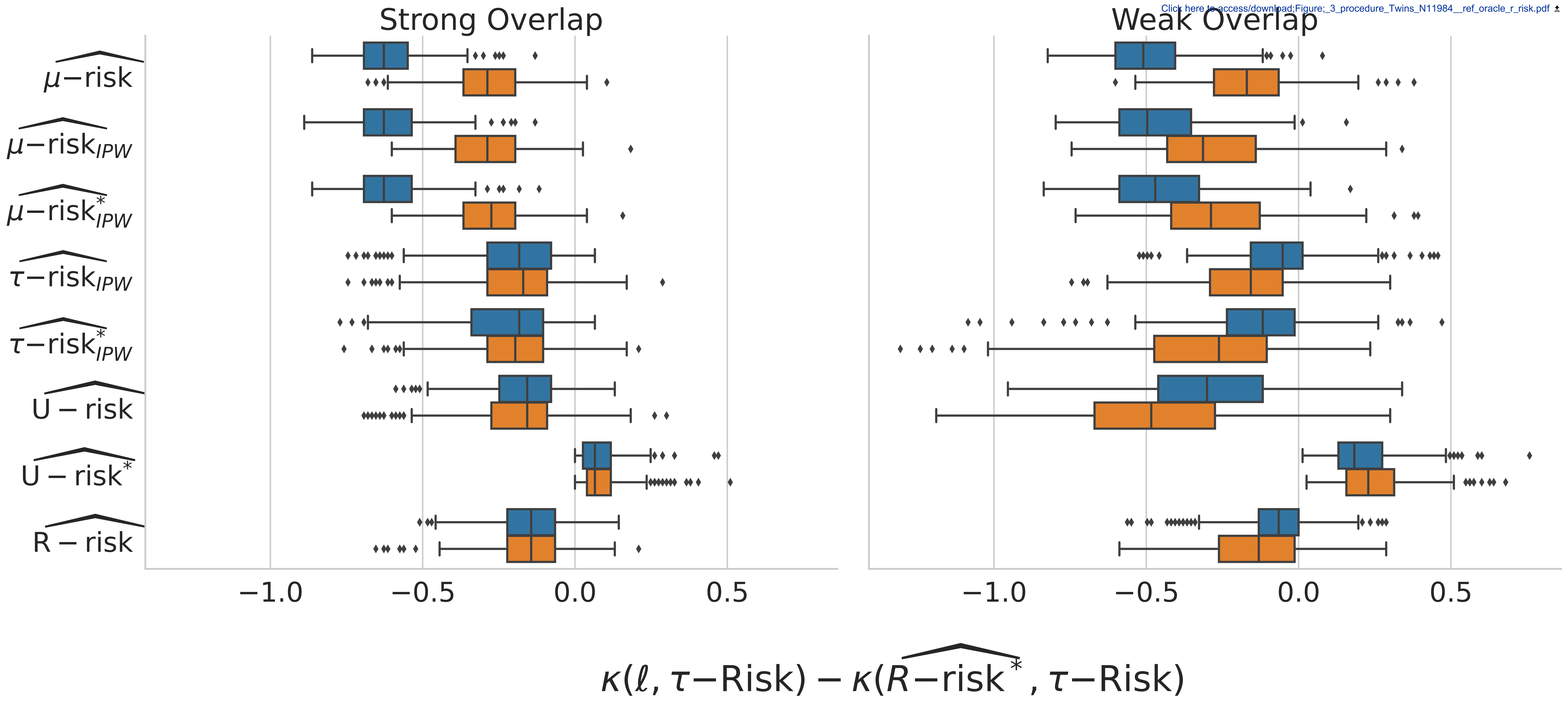

Click here to access/download;Figure;overlap_measure_acic_2018_max_ipw_vs_ntv.pdf

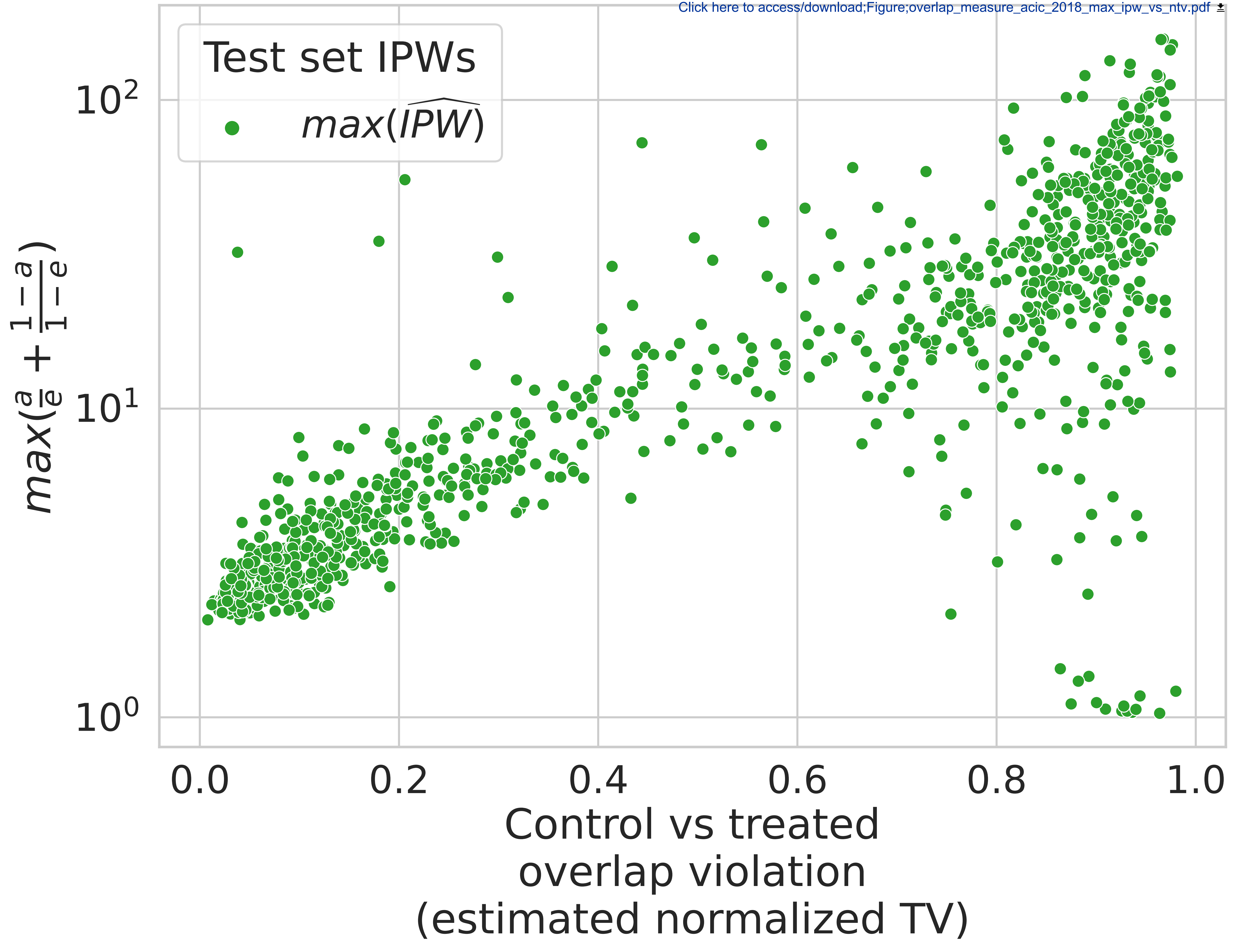

Click here to access/download;Figure;_4_nuisance_models_r_risk_only_3datasets.pdf

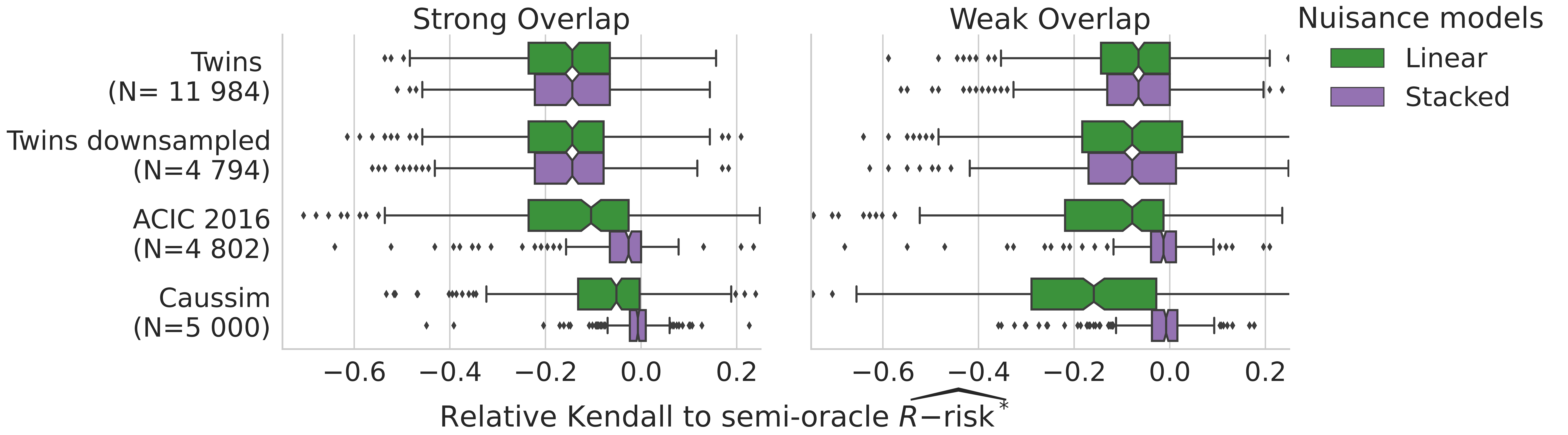

Click here to access/download;Figure;_3_procedure_Caussim_N5000__ref_metric_oracle_r_risk.pdf

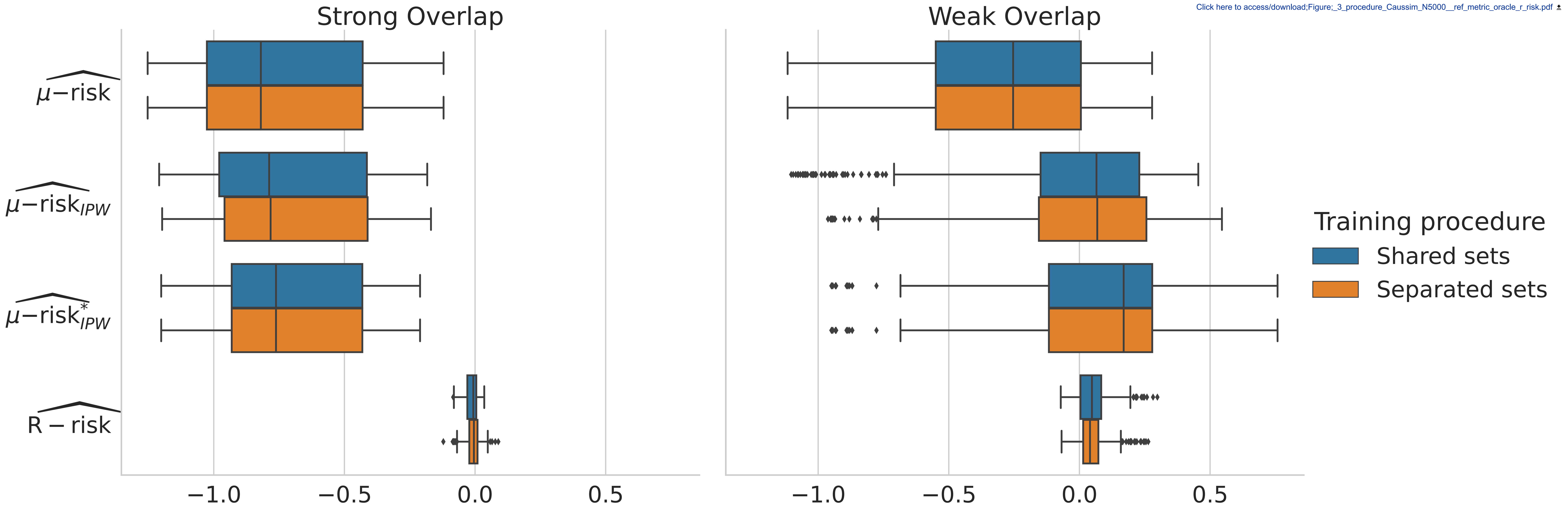

Click here to access/download;Figure;norm_bias_trisk_tobest_caussim_nuis_nlin_models_ridge.pdf

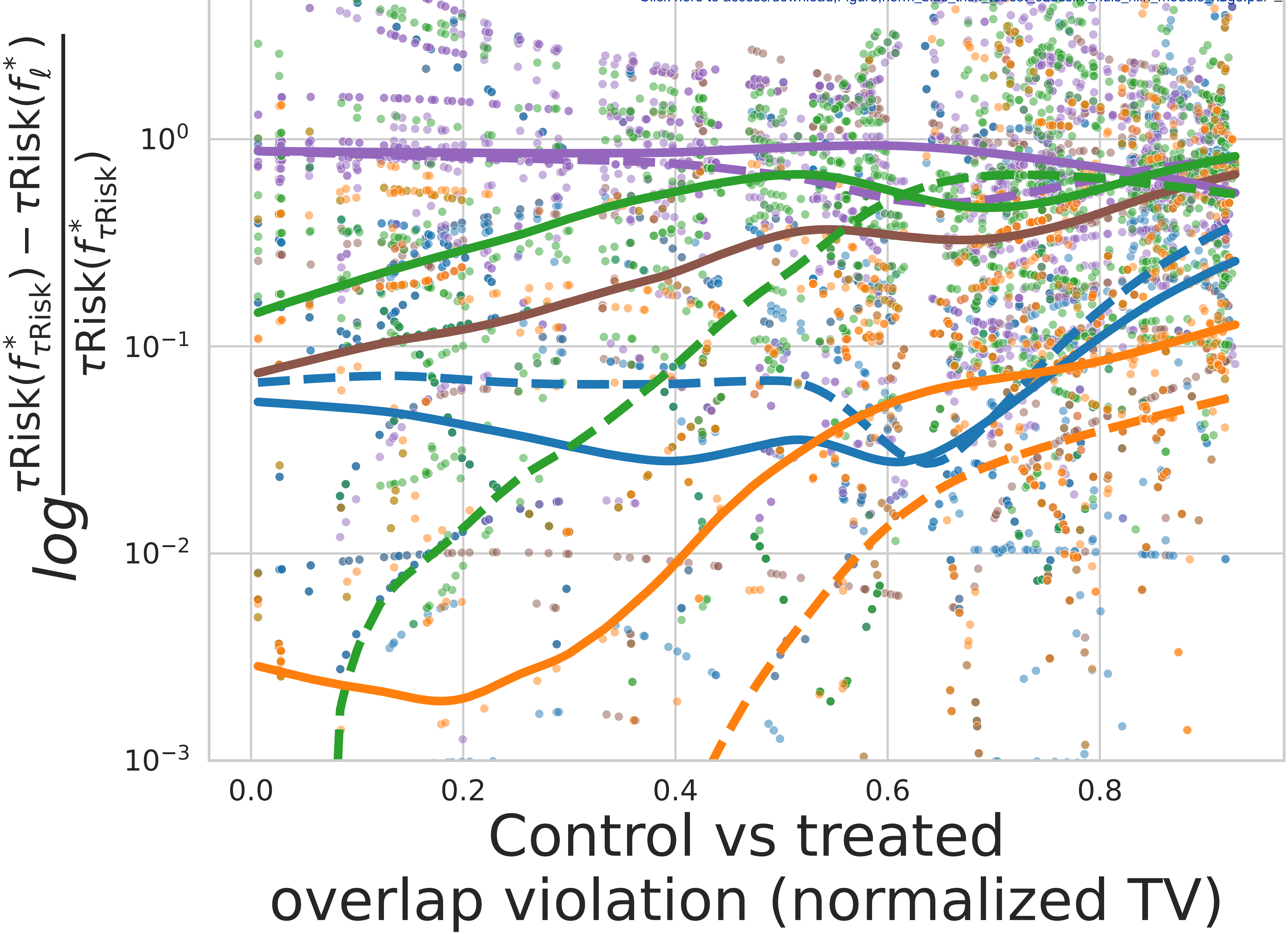

Click here to access/download;Figure;toy_random_forest_high_R2_high_tau_risk.pdf

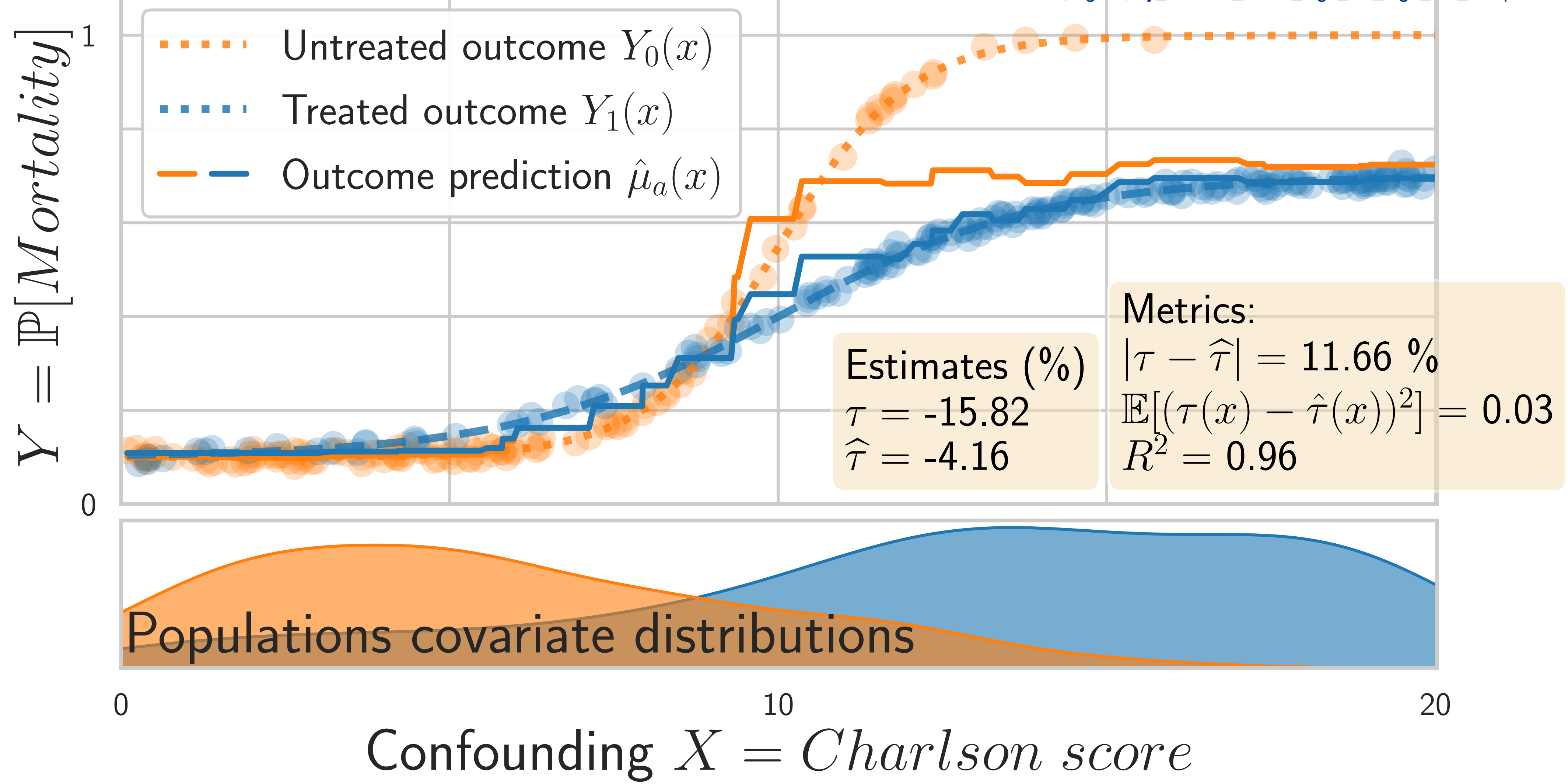

Click here to access/download;Figure;norm_bias_trisk_tobest_acic2018_nuis_nlin_models_hgb.pdf

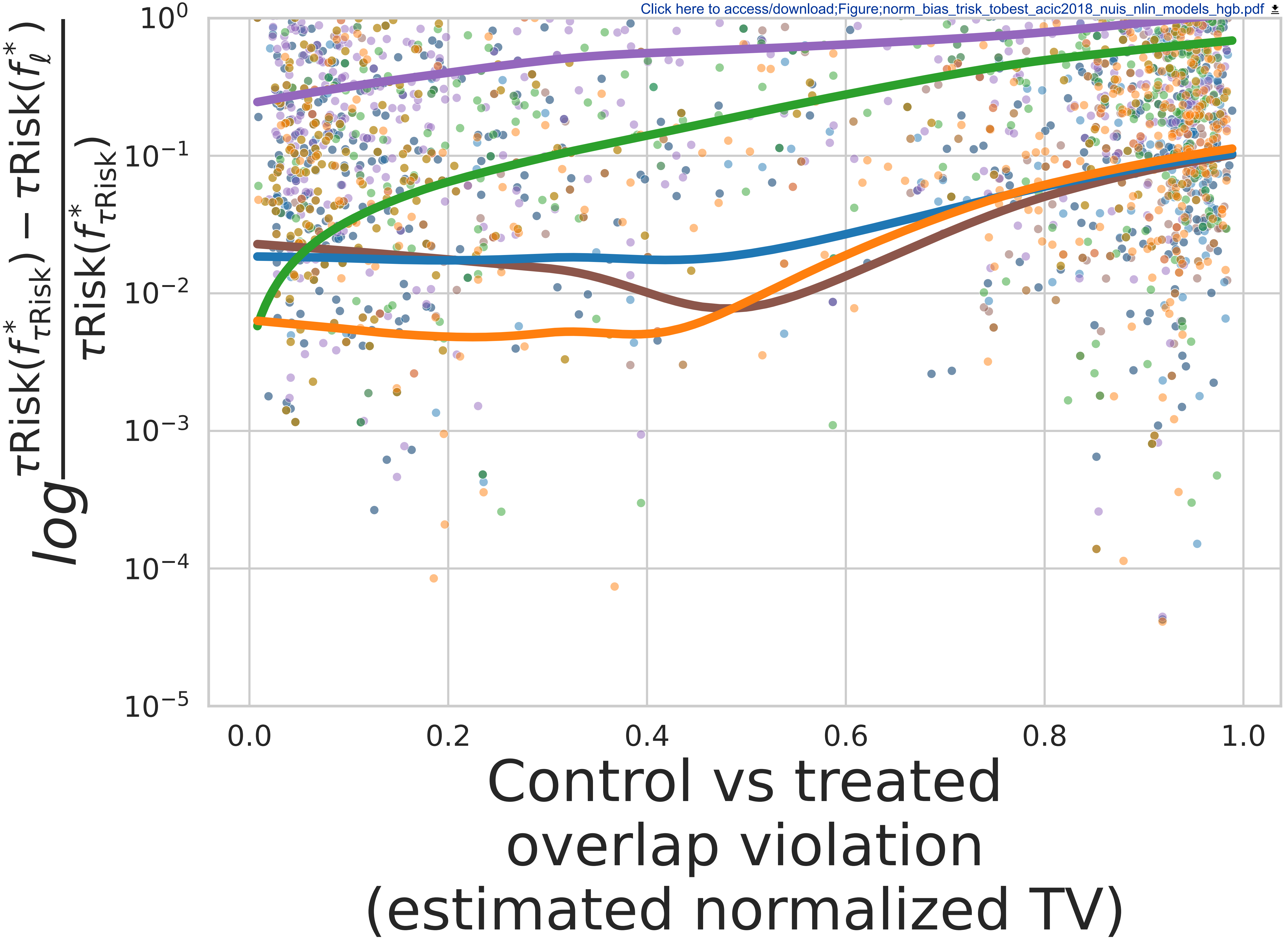

Click here to access/download;Figure;overlap_measure_caussim_max_ipw_vs_ntv.pdf

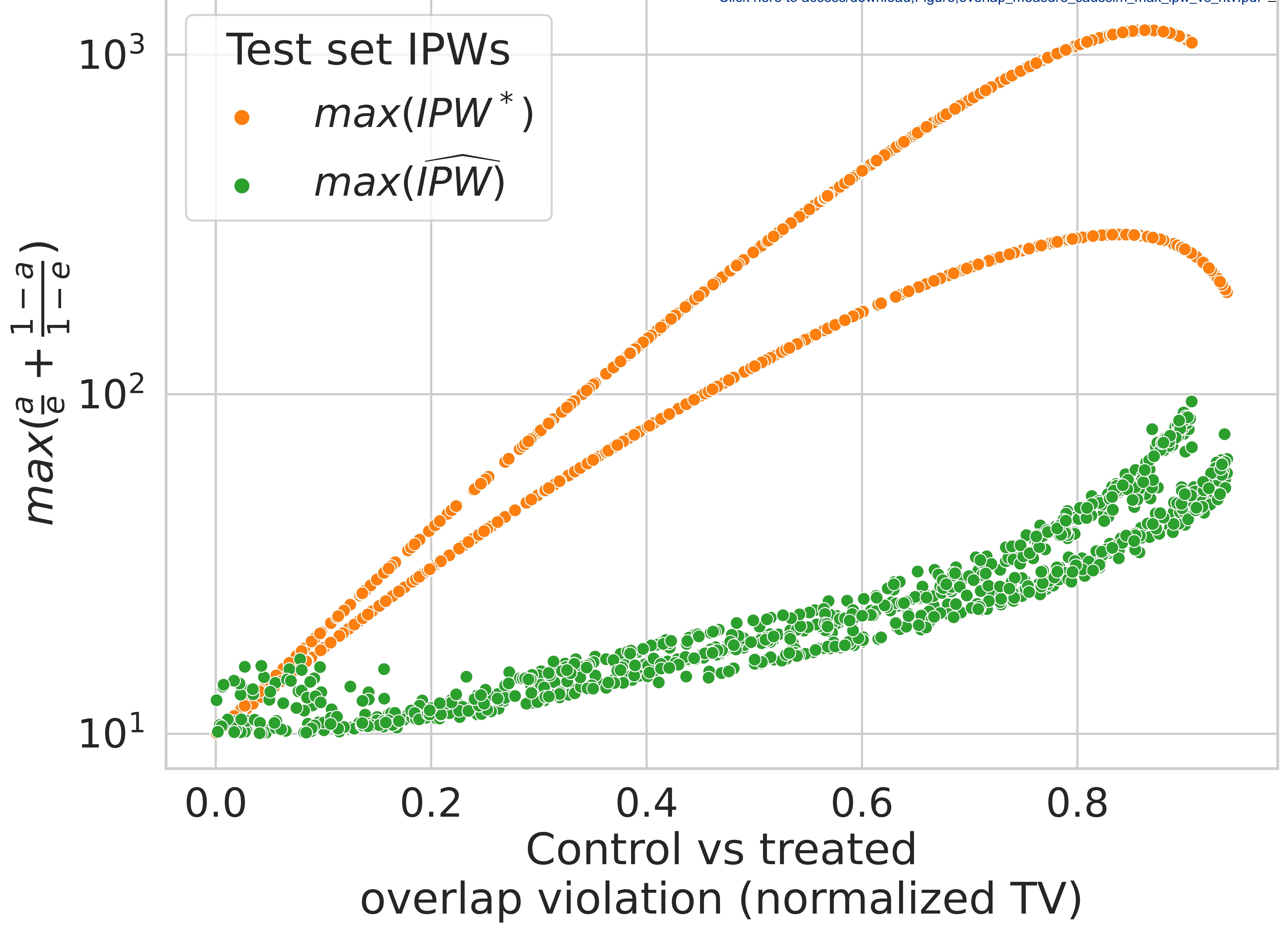

Click here to access/download;Figure: 4_nuisance_models_Twinssmall_N4794__ref_oracle_r_risk.pdf

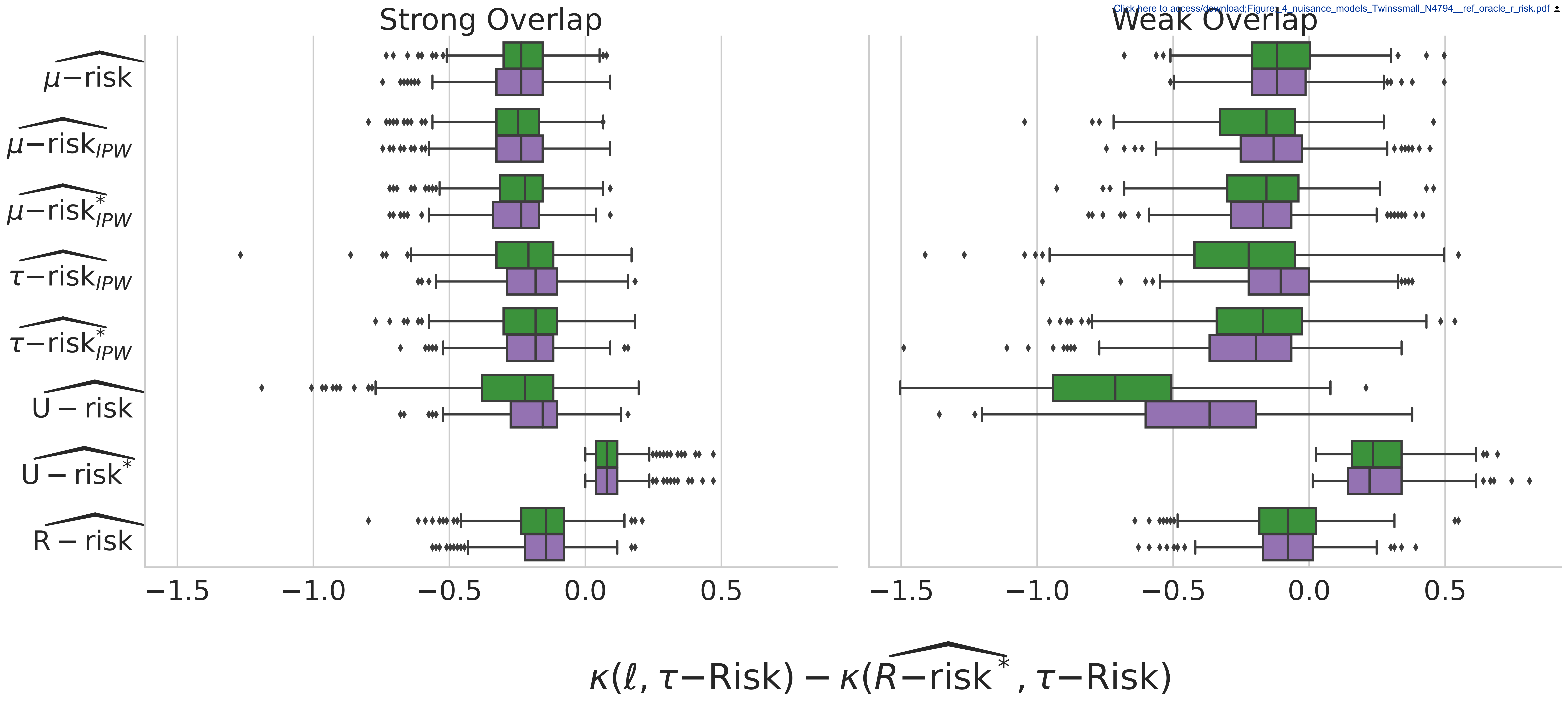

Click here to access/download;Figure;ktau_caussim_nuisance_nlin_models_ridge_ov1-247.pdf

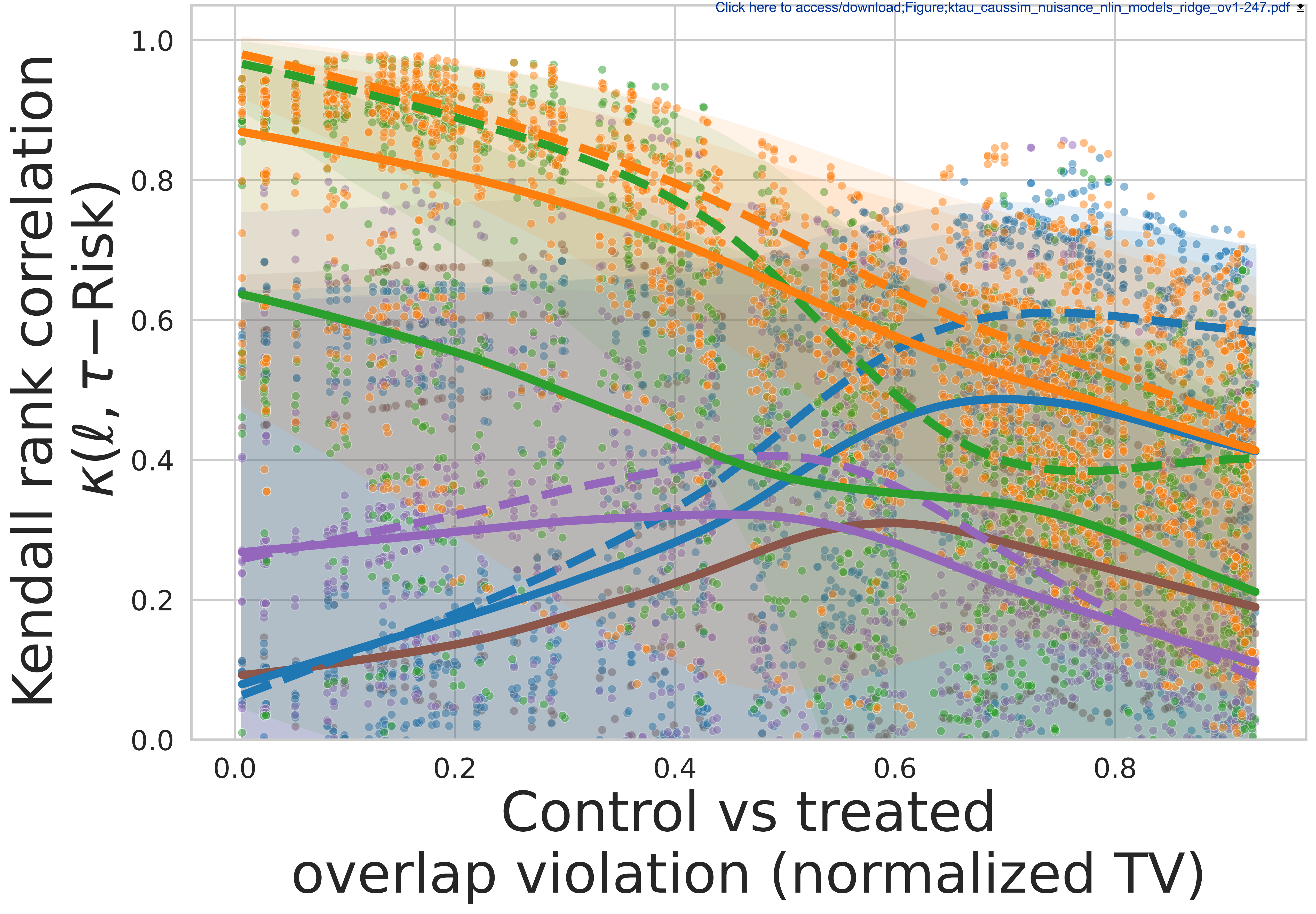

Click here to access/download;Figure;_2_overlap_influence_by_bin_k_by_ds.pdf

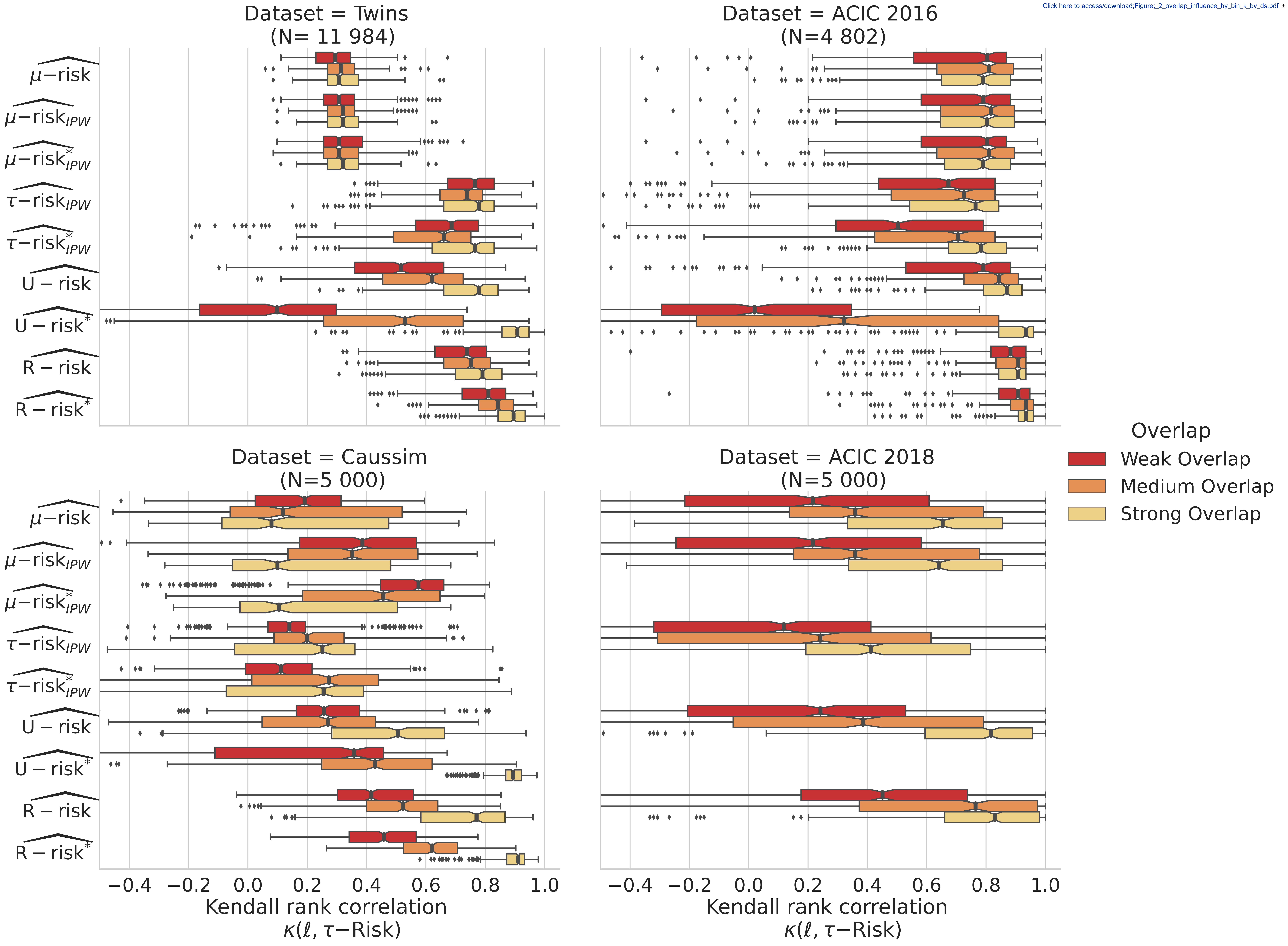

Click here to access/download;Figure;overlap_measure_acic_2016_max_ipw_vs_ntv.pdf

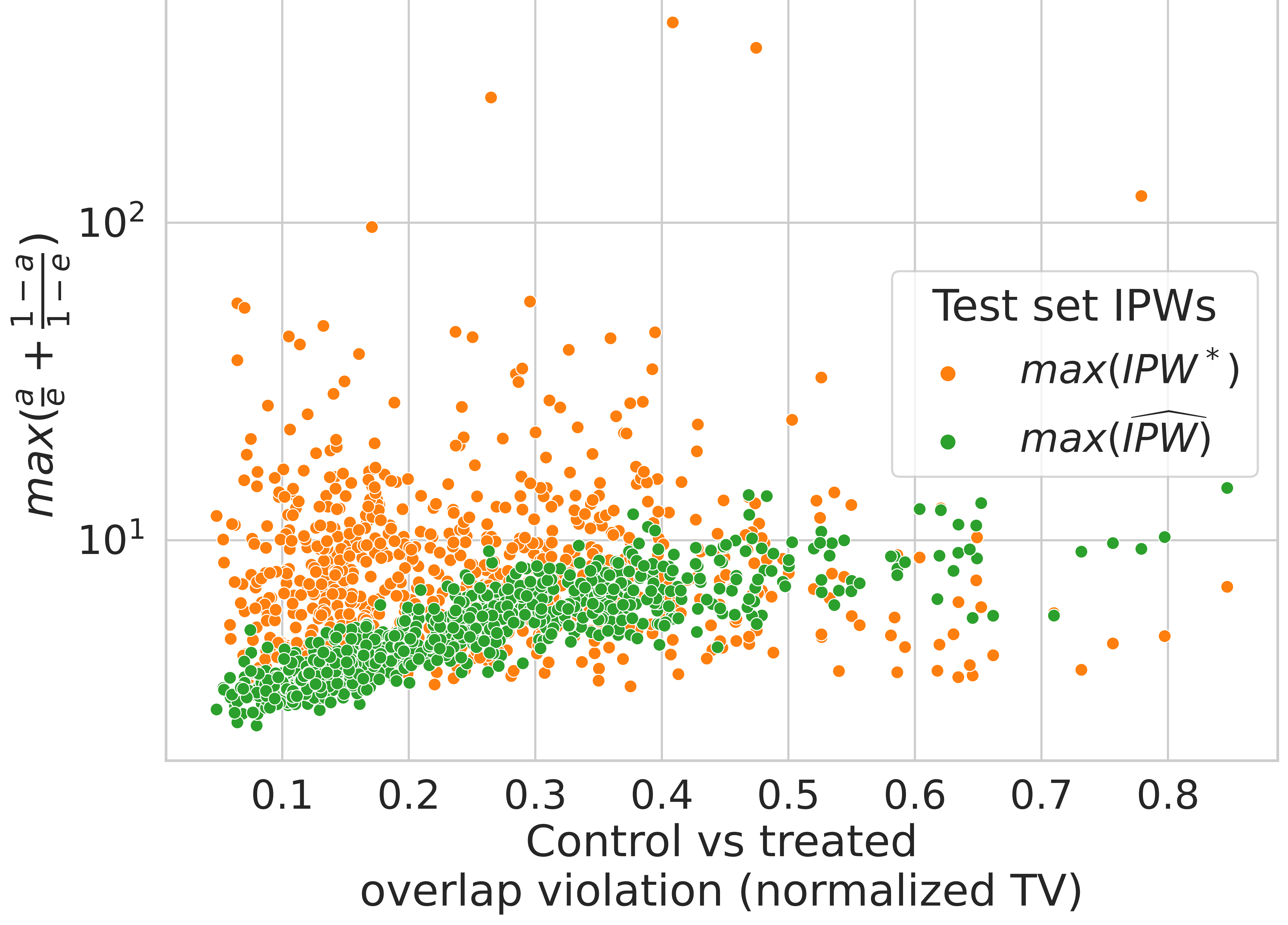

Click here to access/download;Figure;_5_train_size_r_risk__cate__acic16_2cols.pdf

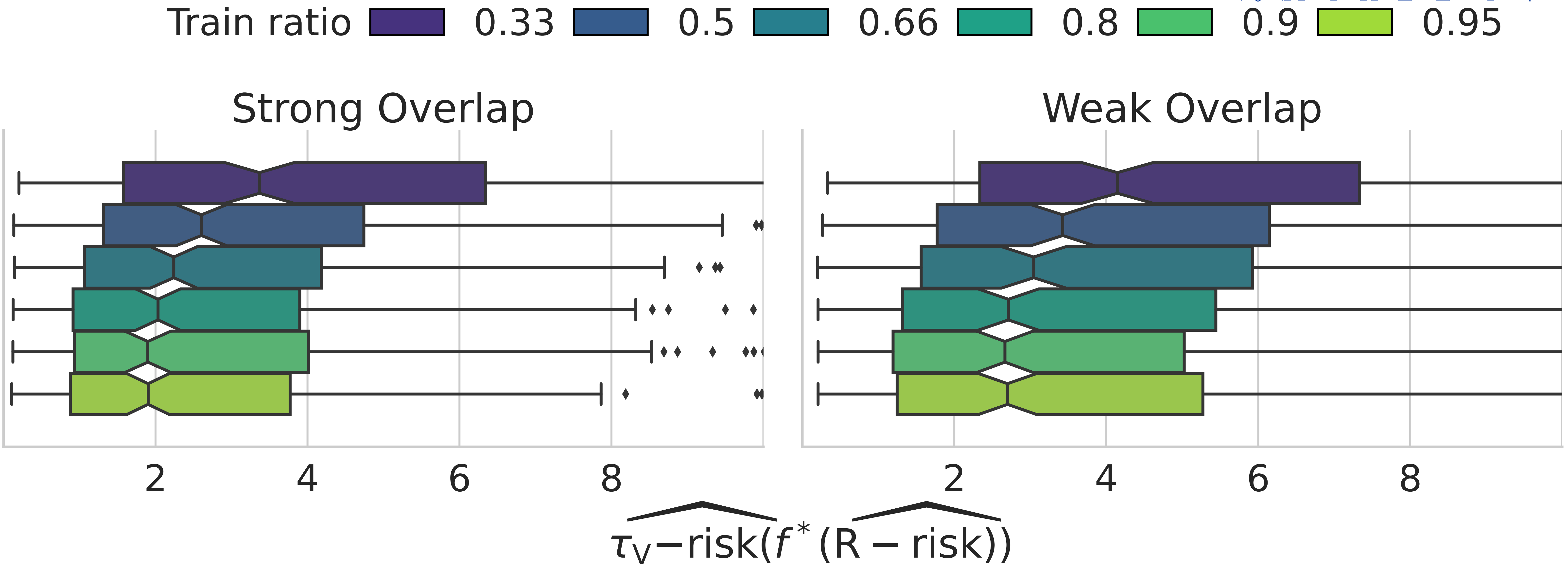

Click here to
access/download;Figure;estimation_procedure_causal_selection_procedure.png

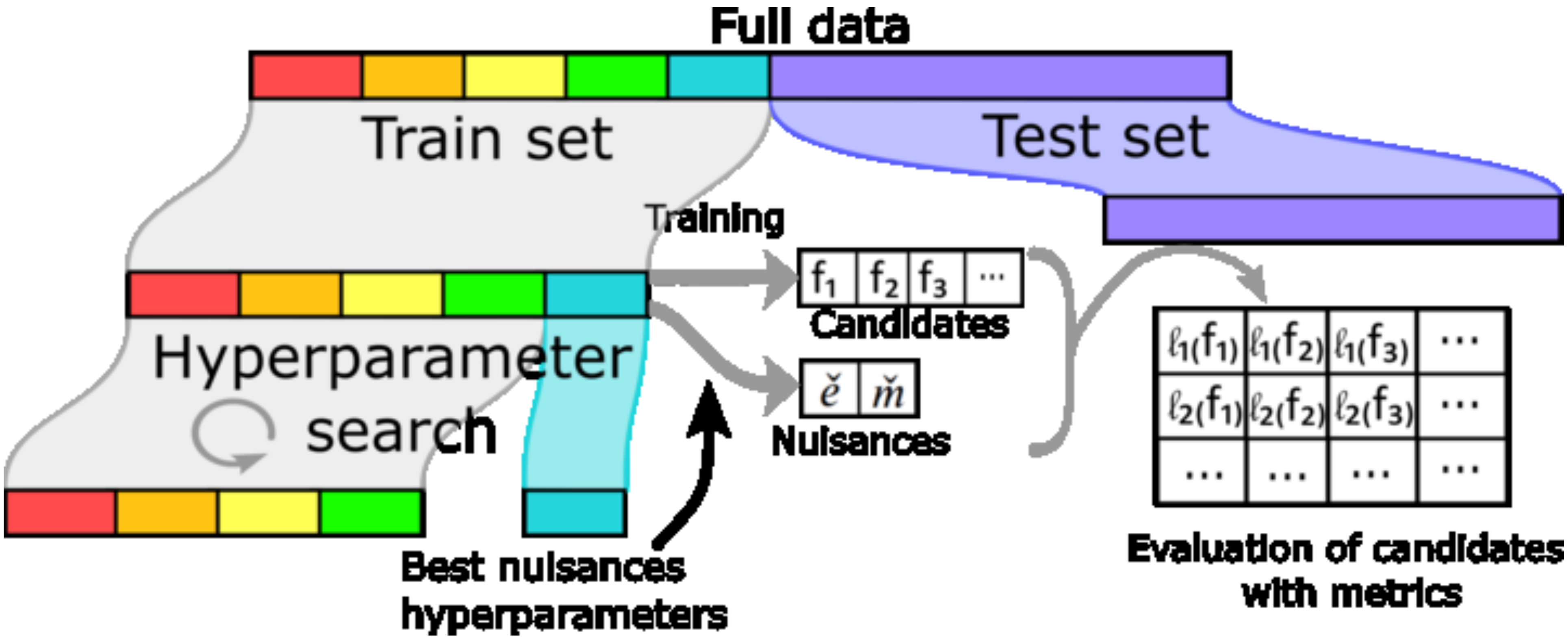

Click here to access/download;Figure;_1_r_risk_domination_mrisks_by_ds.pdf

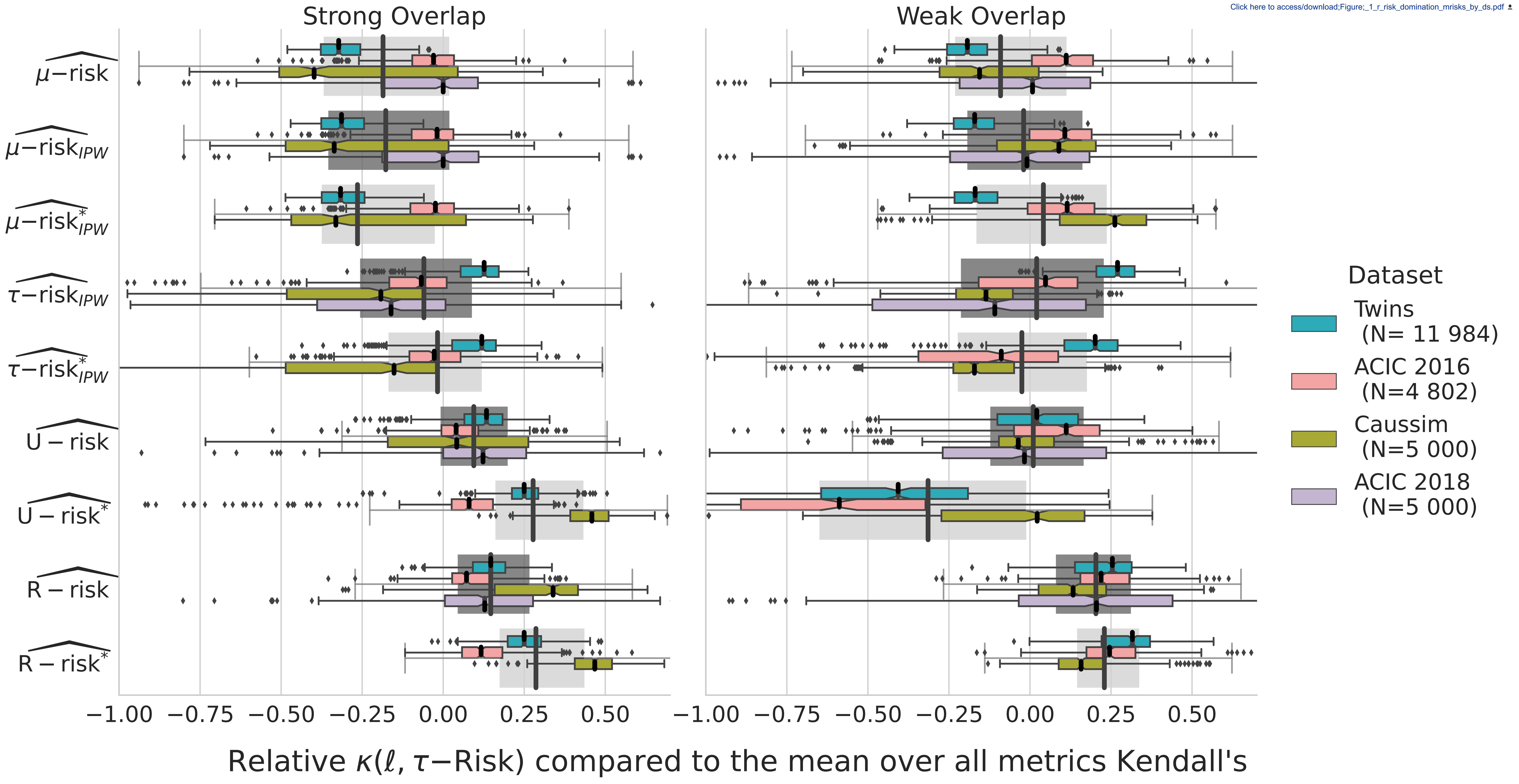

Click here to access/download;Figure;caussim_seed_effect.pdf

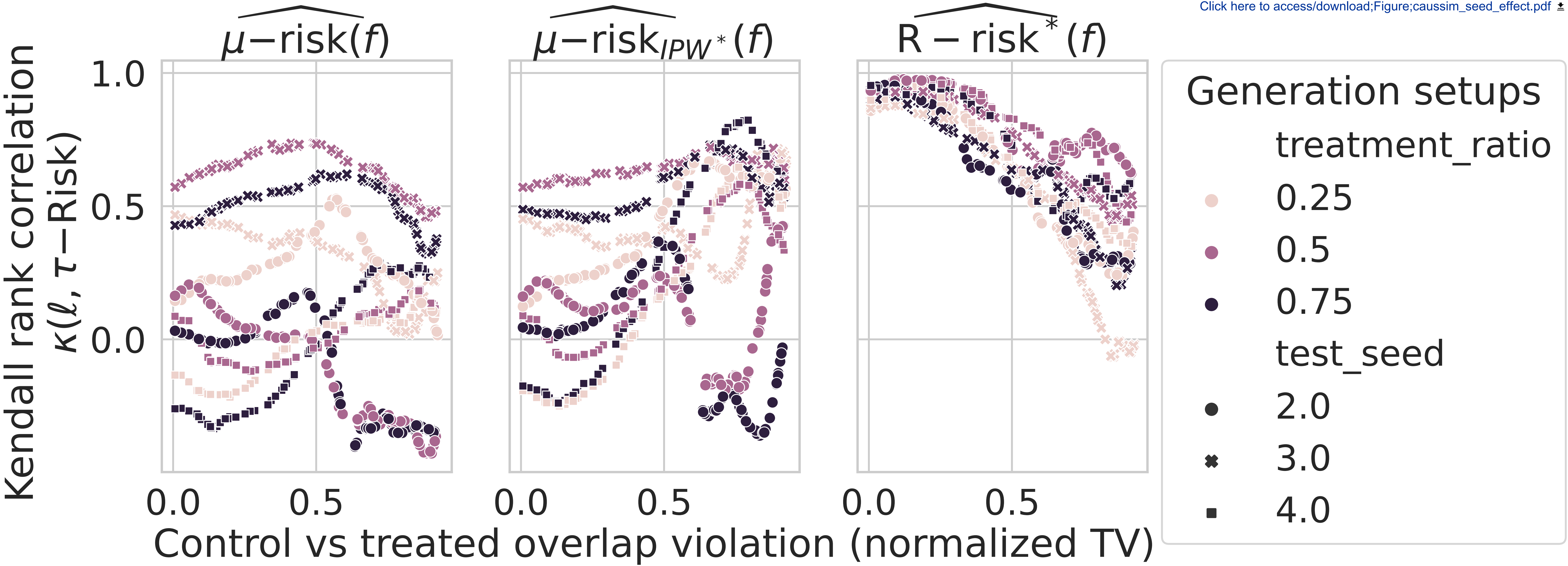

Click here to access/download;Figure;ktau_acic2018_nuisance_stacking_models_hgb_tset_50.pdf

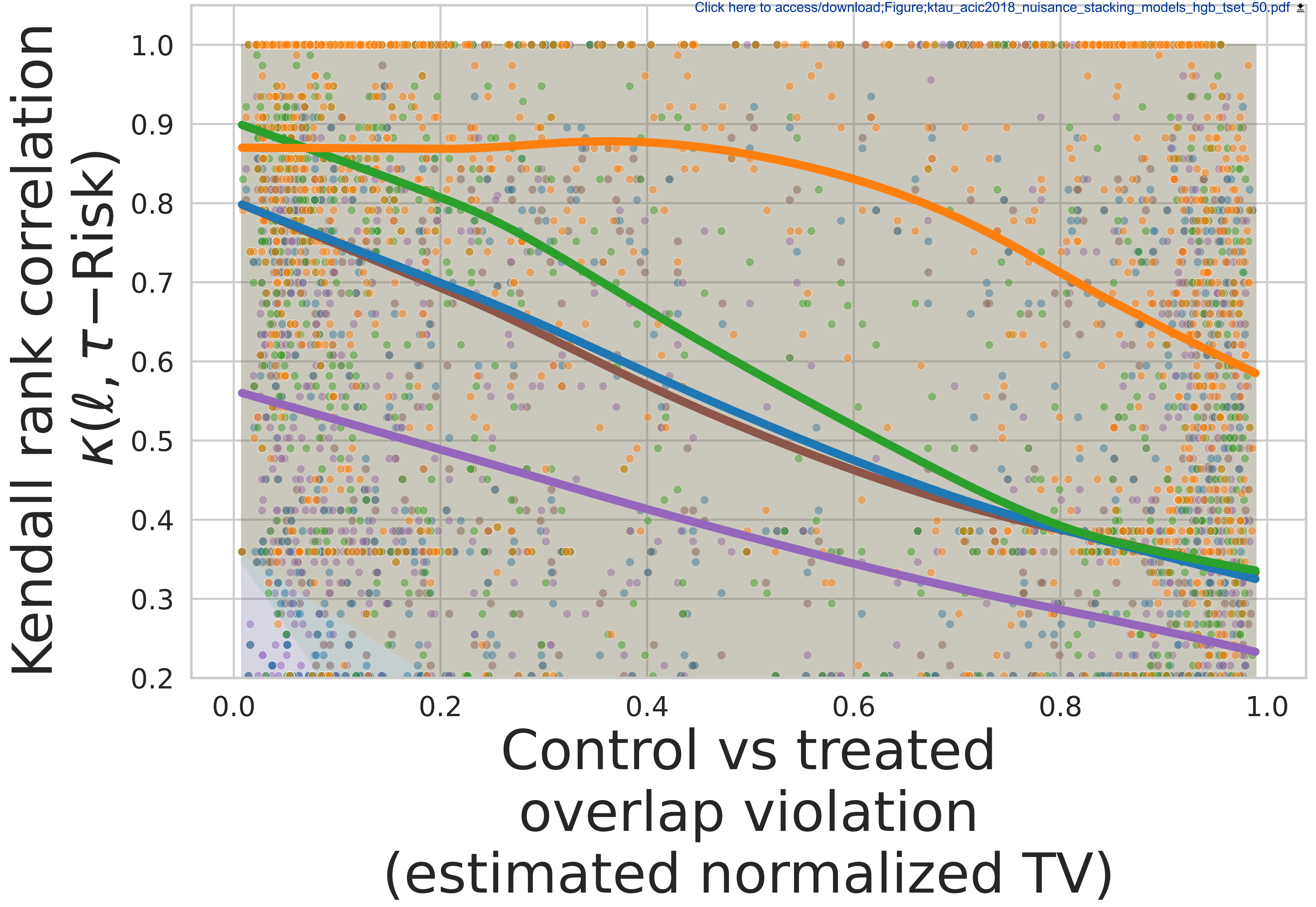

Click here to access/download;Figure;_4_nuisance_models_Caussim_N5000__ref_oracle_r_risk.pdf

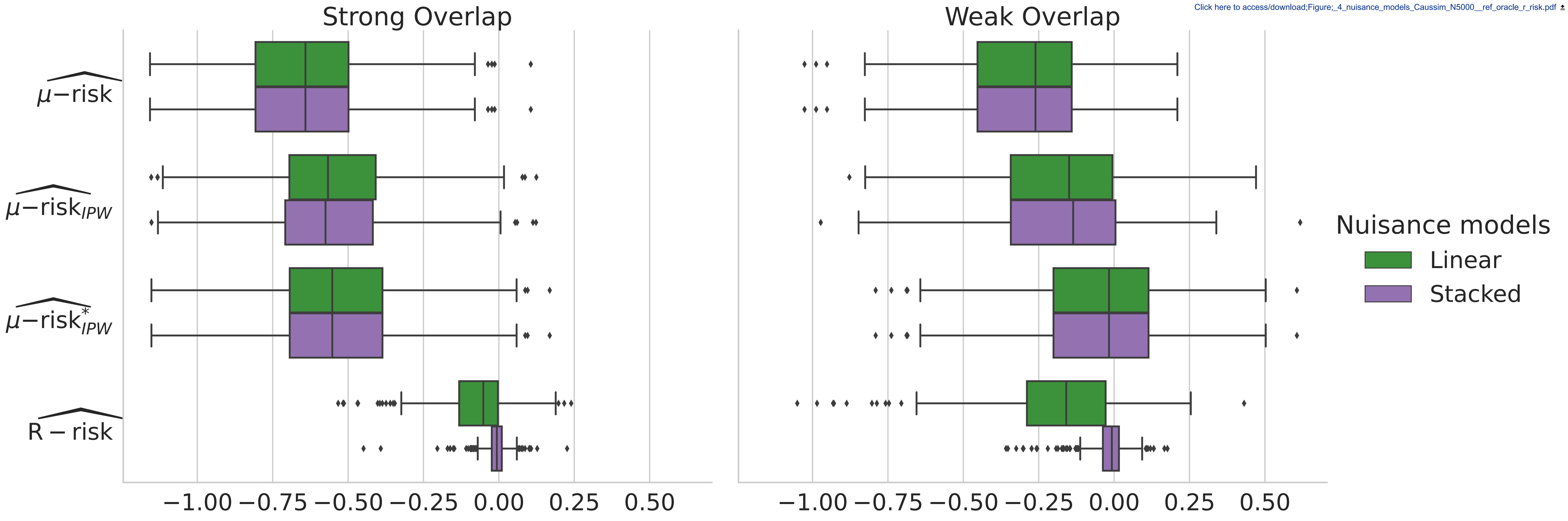

Click here to access/download;Figure_4_nuisance_models_ACIC2016_N4802__ref_oracle_r_risk.pdf

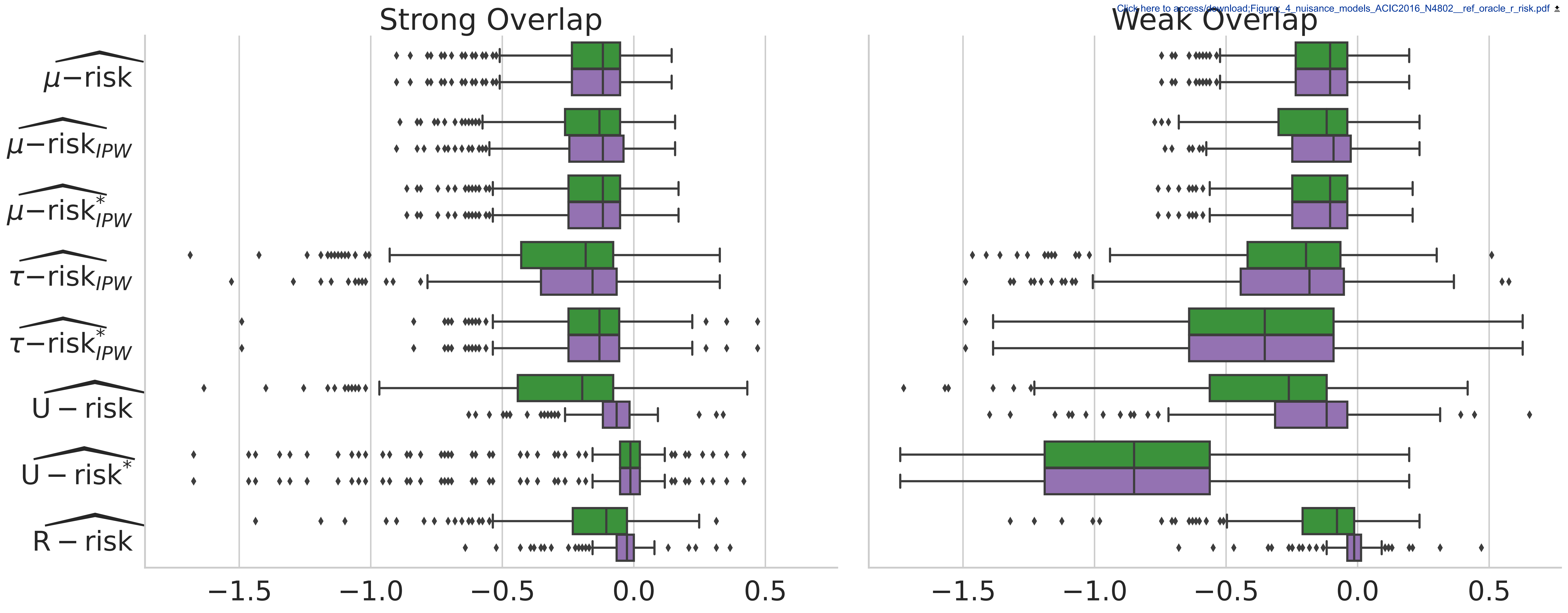

Click here to access/download;Figure;_4_nuisance_models_r_risk_3ds_2cols.pdf

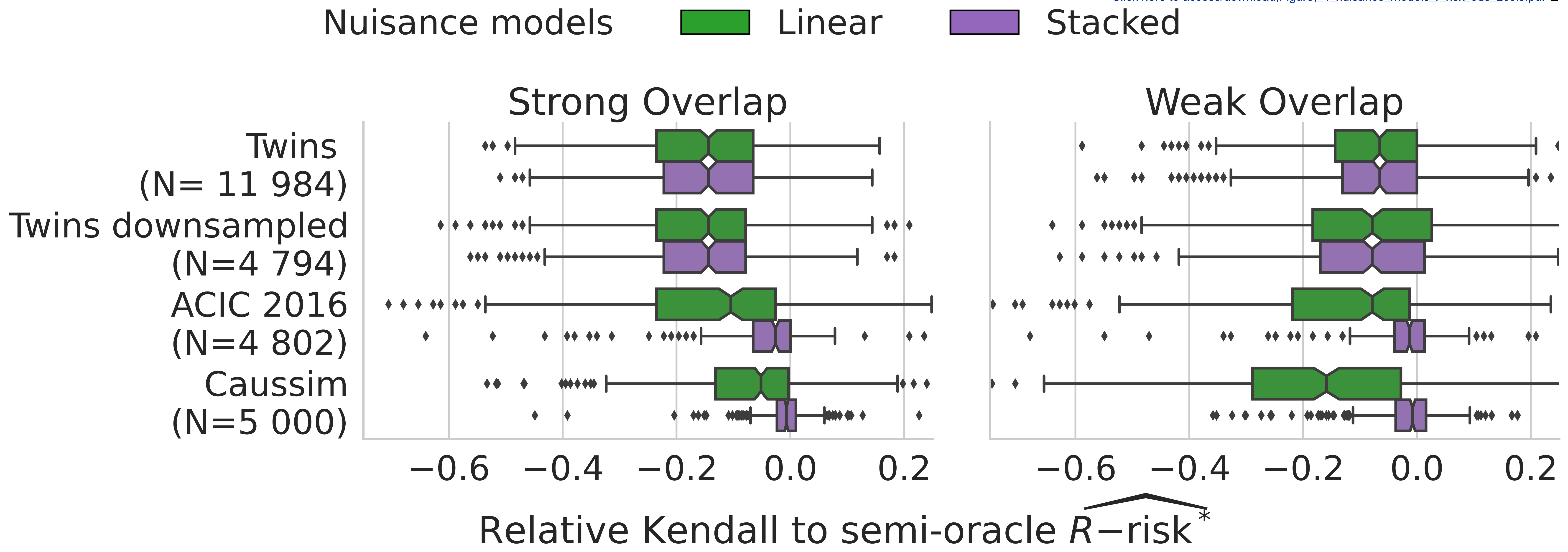

Click here to access/download;Figure;caussim_example.pdf

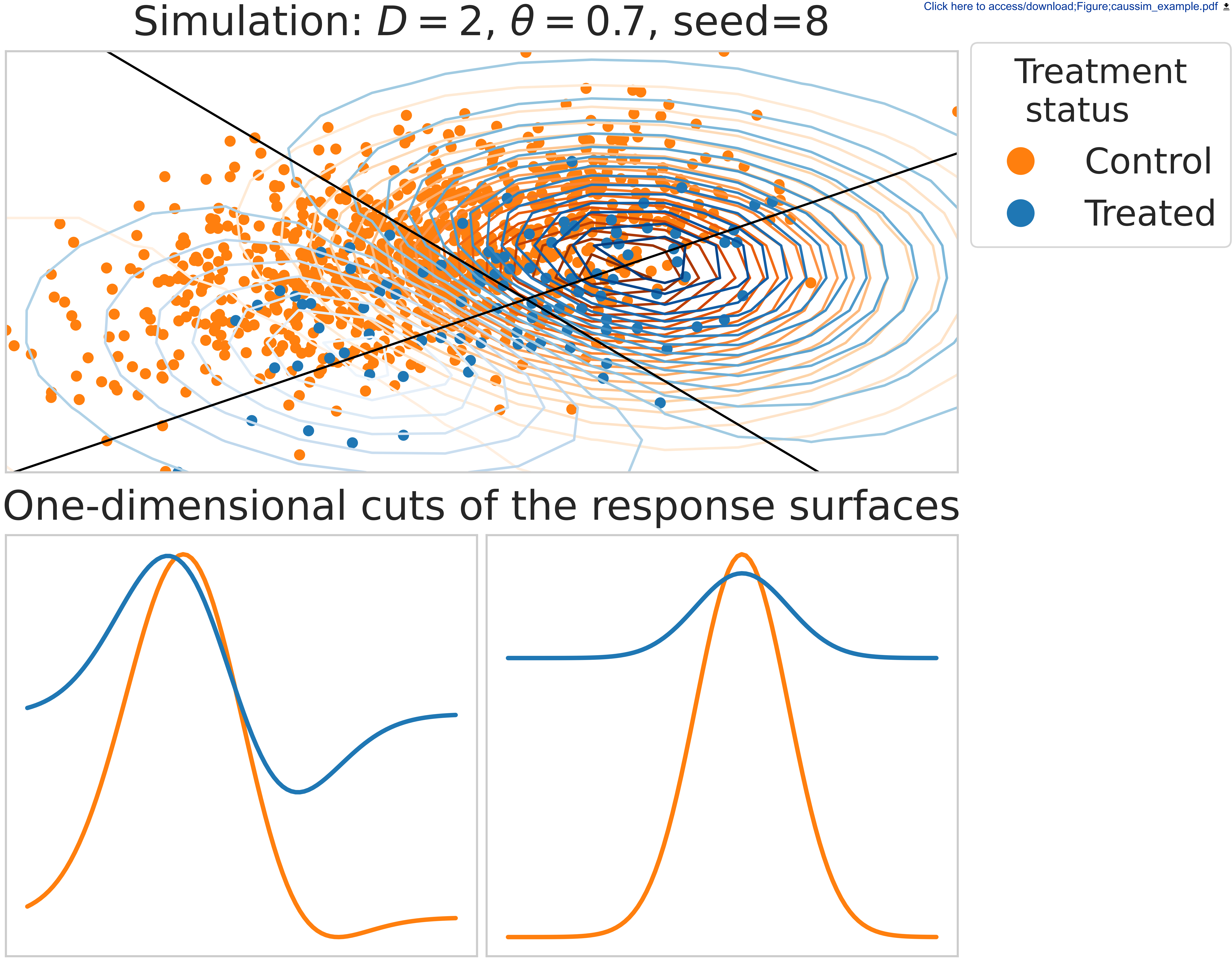

Click here to access/download;Figure;_5_train_size_r_risk__ate__acic16_2cols.pdf

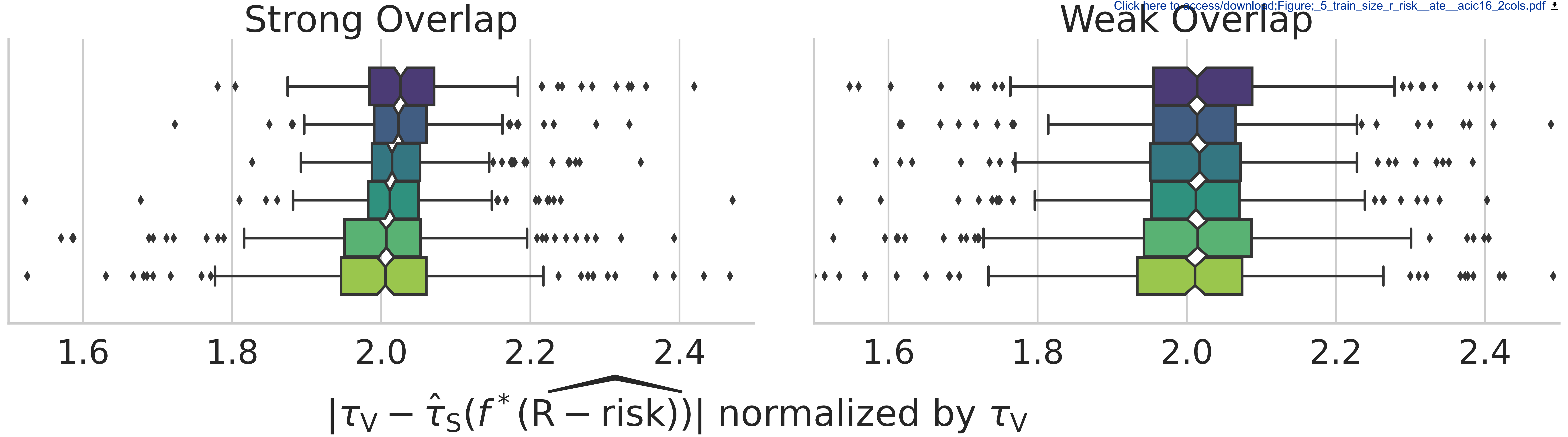

Click here to access/download;Figure;norm_bias_trisk_tobest_twins_nuis_stack_models_hgb.pdf

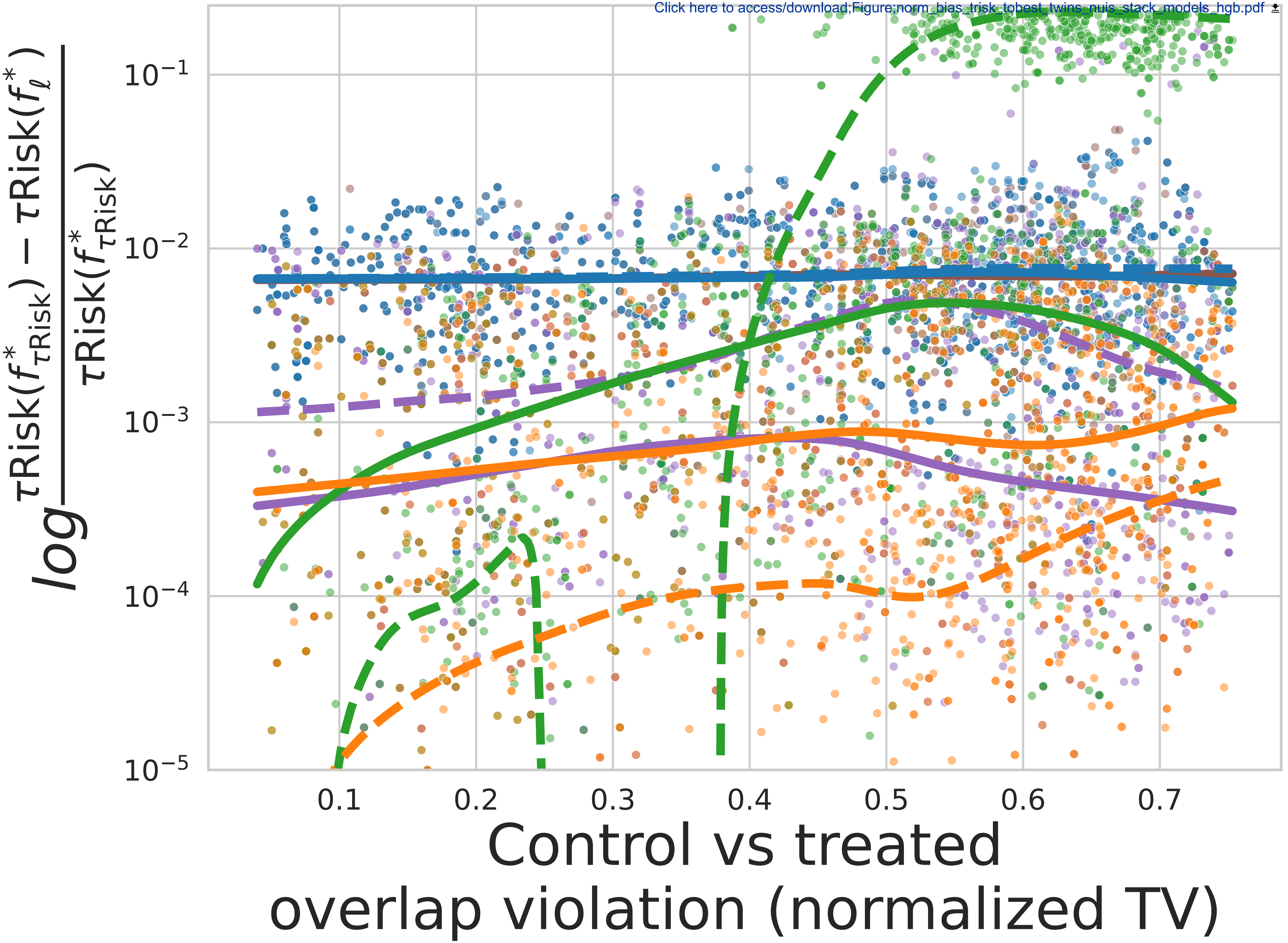

Click here to access/download;Figure;_3_procedure_r_risk_only_3datasets_twocols.pdf

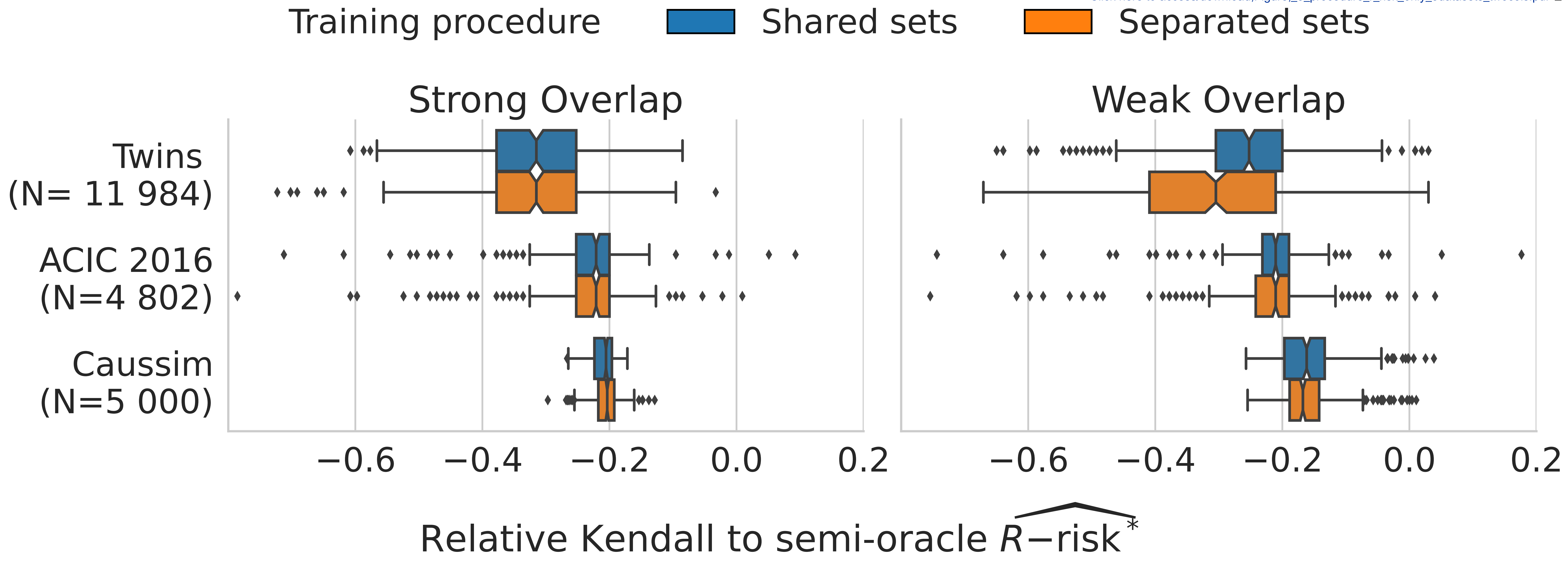

Click here to access/download;Figure;_1_r_risk_domination_mrisks_by_ds_feasible.pdf

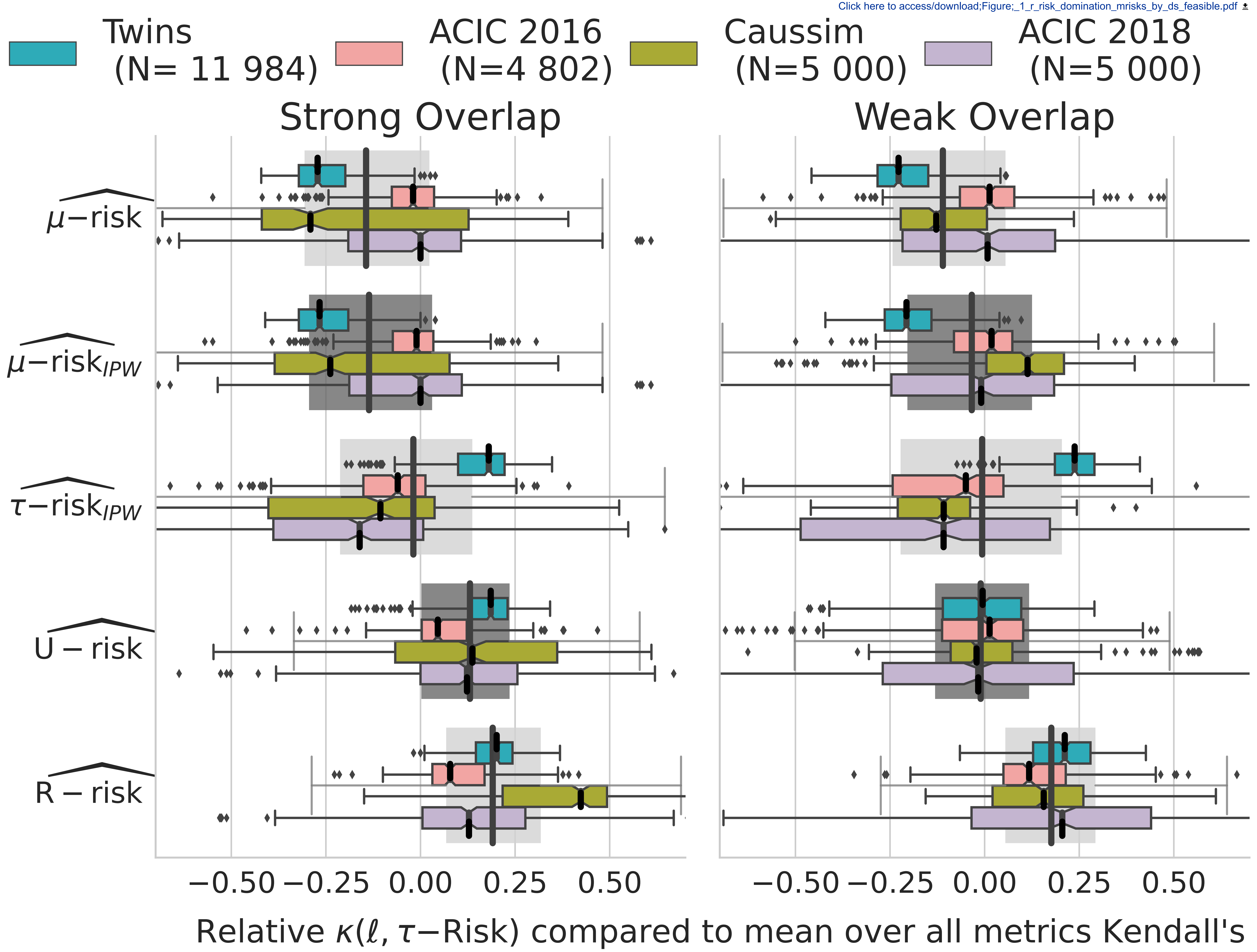

Click here to access/download;Figure;_4_nuisance_models_Twins_N11984__ref_oracle_r_risk.pdf

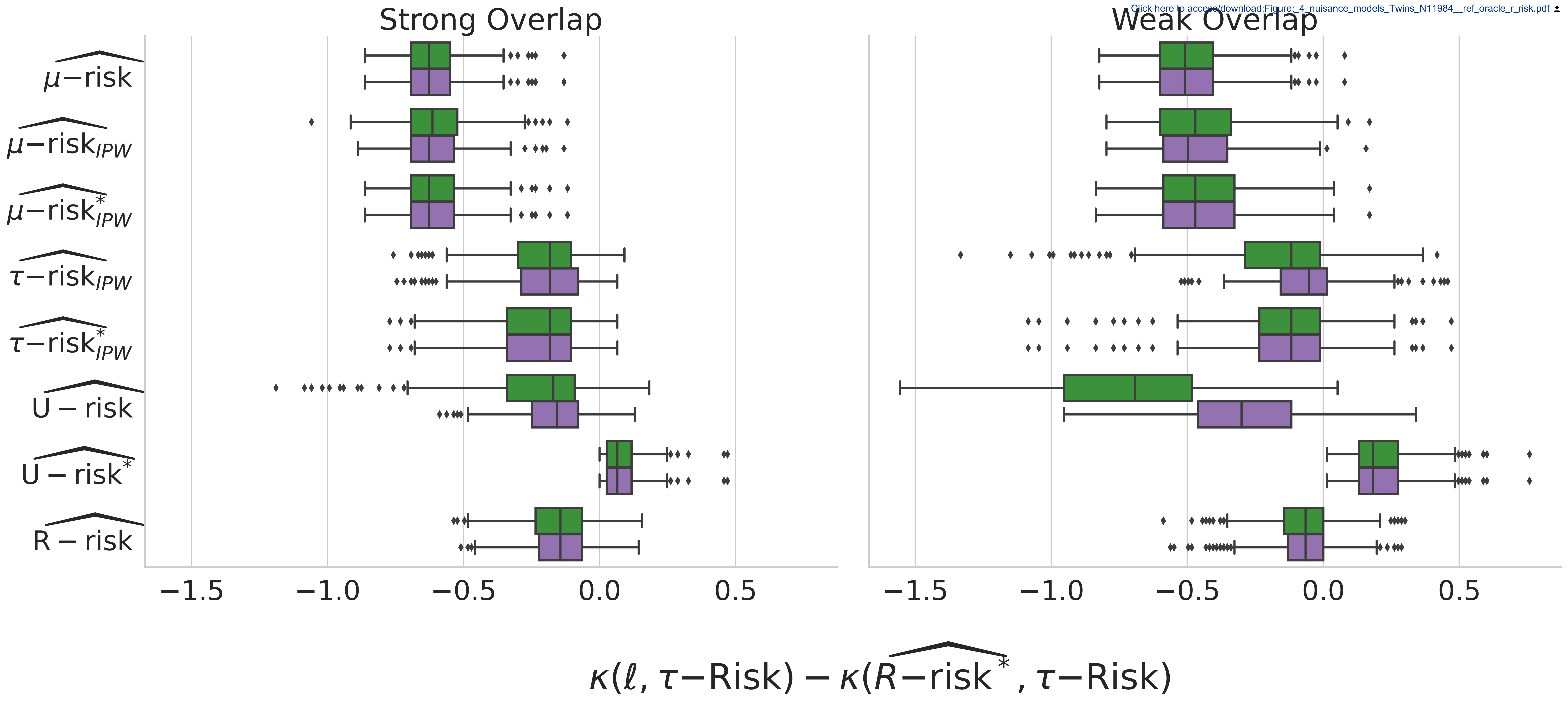

Click here to access/download;Figure;overlap_measure_diff_oracle_ntv_to_pred_ntv.pdf

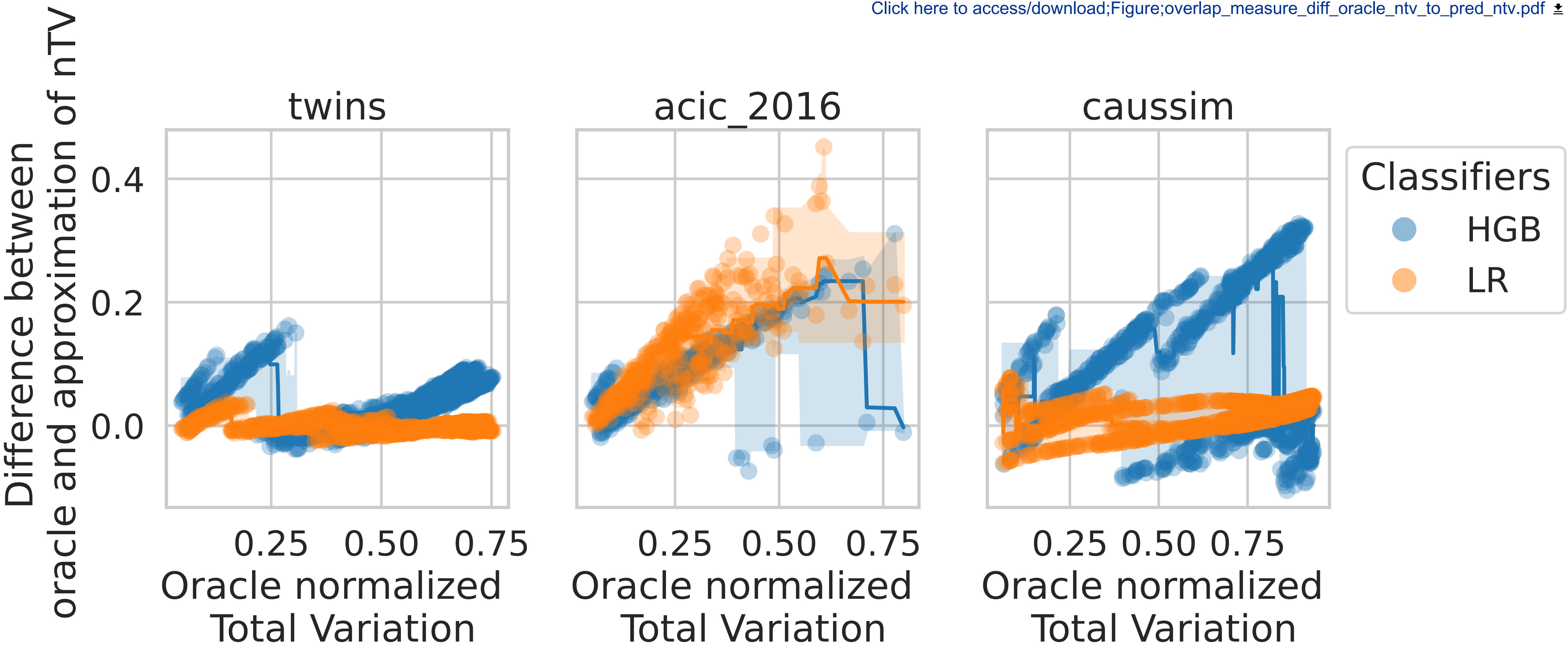

Click here to access/download;Figure;overlap_measure_caussim_max_ipw_vs_ntv.png.pdf

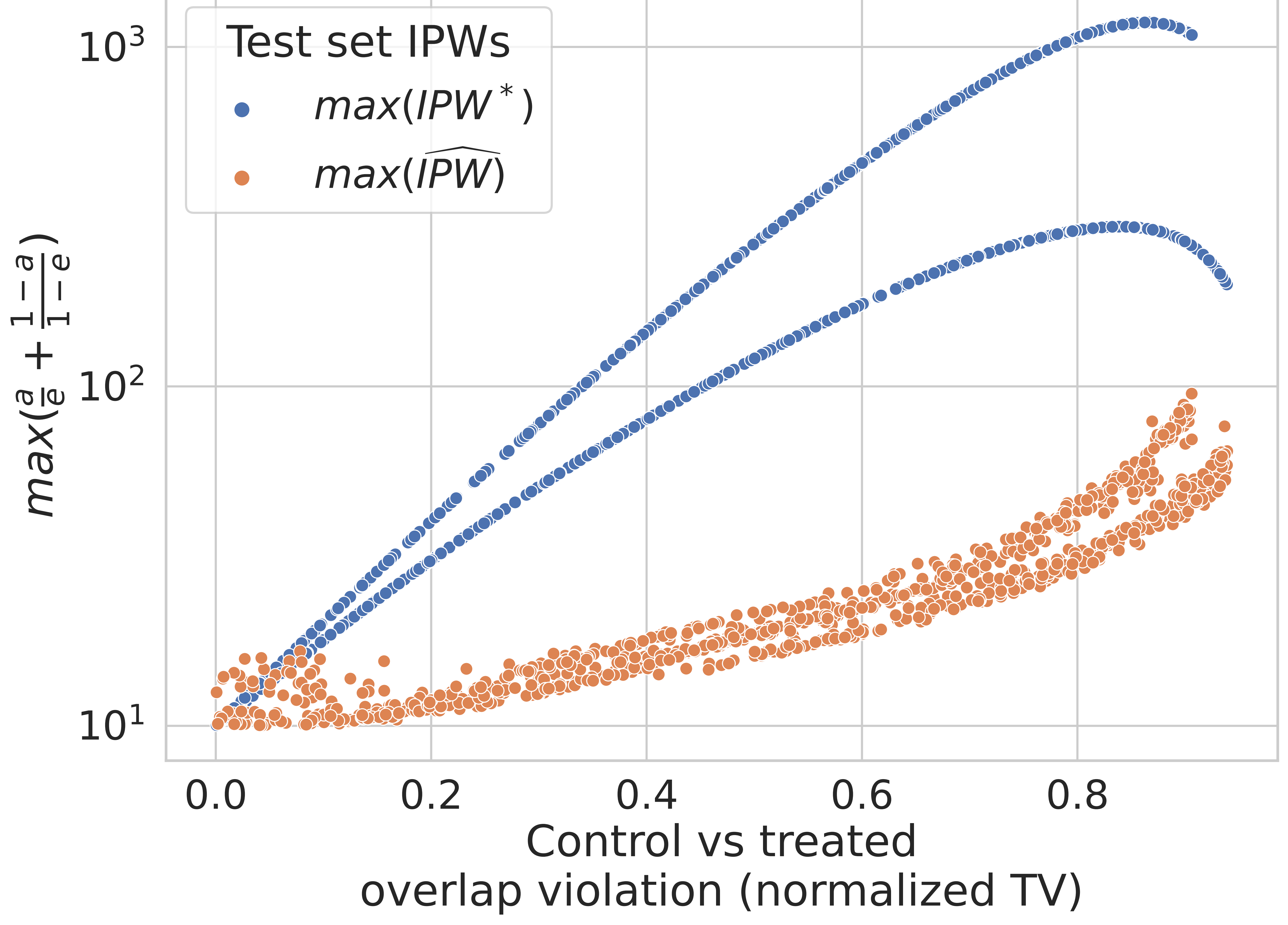

Click here to access/download;Figure;overlap_measure_diff_oracle_ntv_to_ntv_wcalibration.pdf

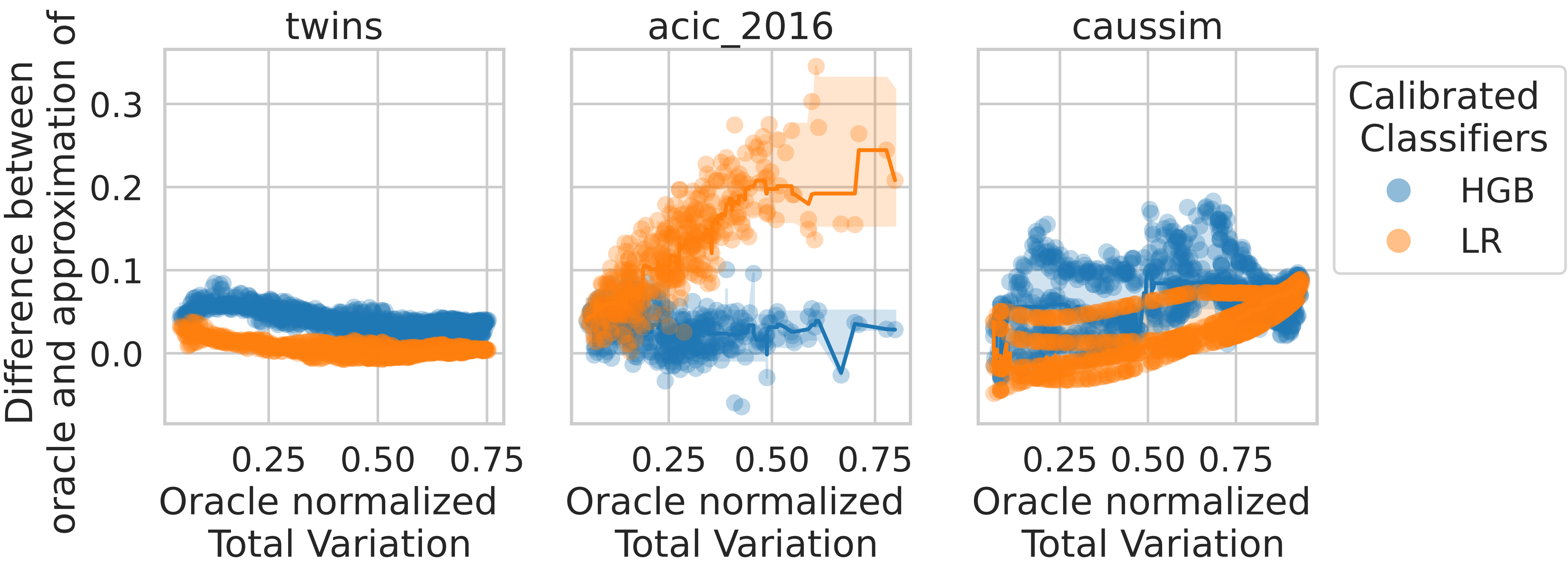

Click here to access/download;Figure;_3_procedure_ACIC2016_N4802__ref_oracle_r_risk.pdf

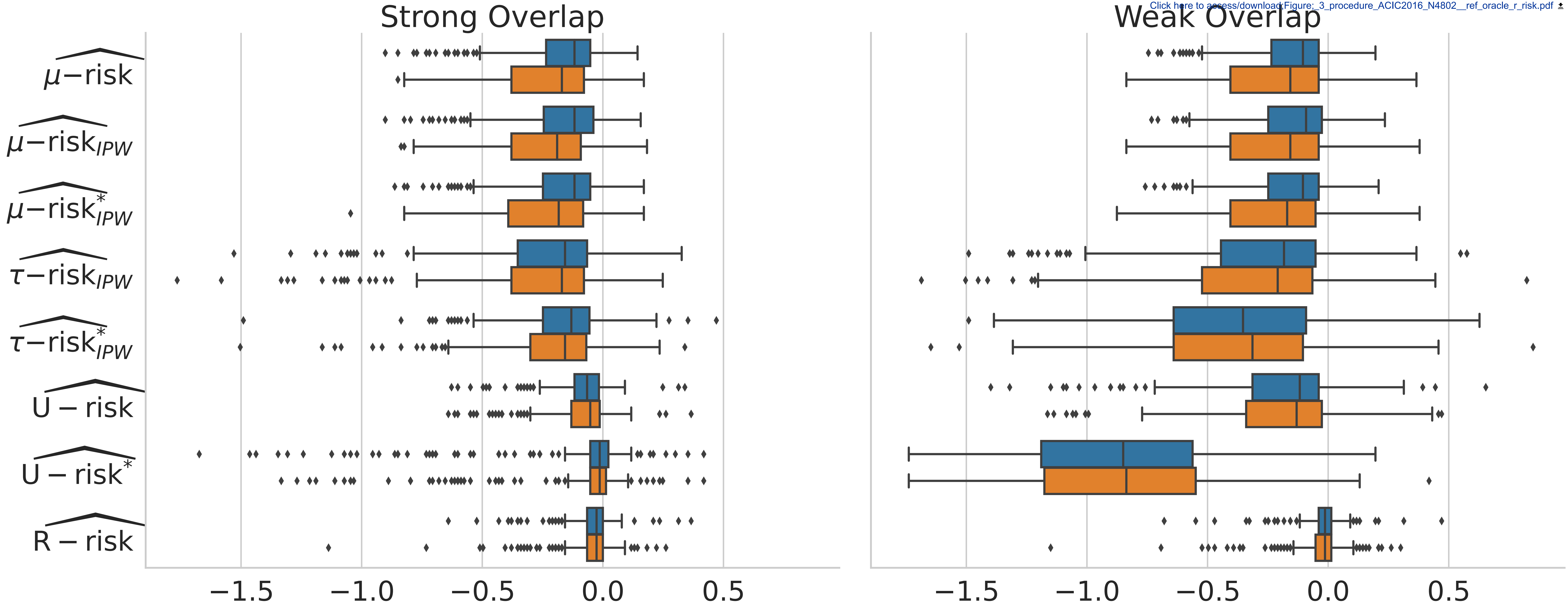

Click here to access/download;Figure;overlap_measure_caussim_transformed_mmd_vs_ntv.pdf

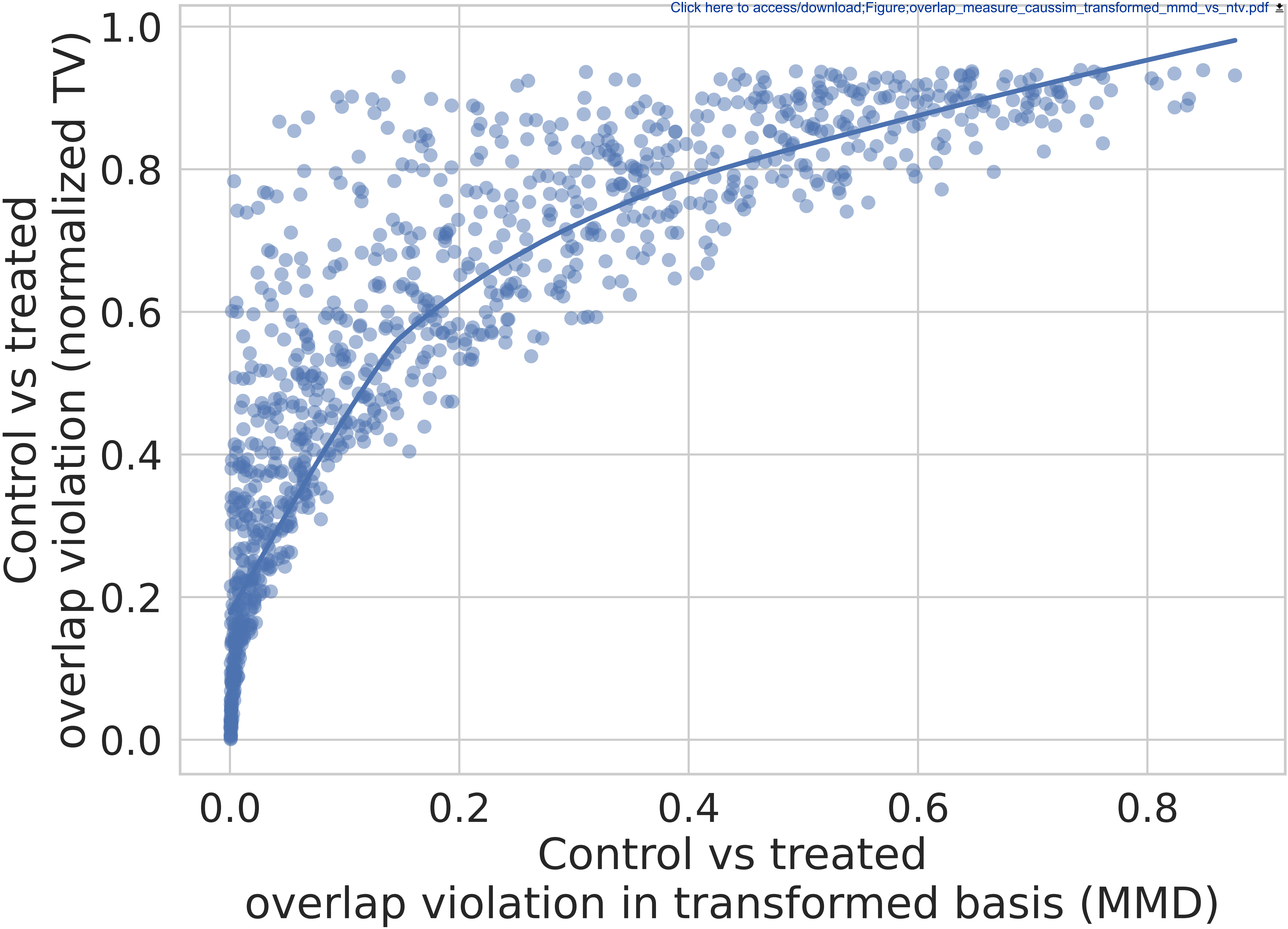

Click here to access/download;Figure;_4_nuisance_models_r_risk_only_twins_nuisances.pdf

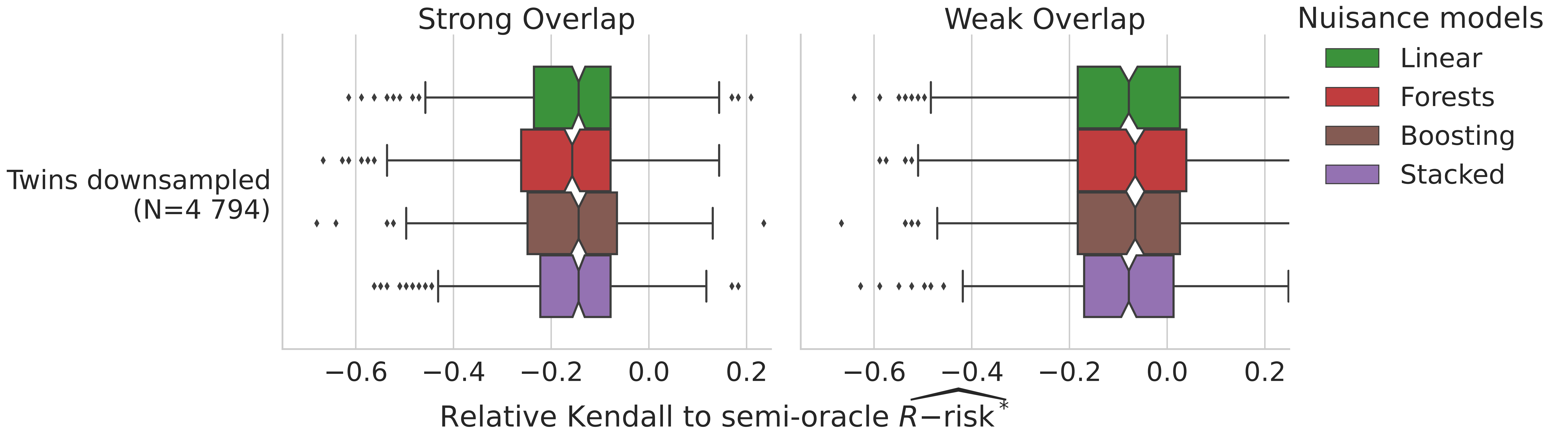

Click here to access/download;Figure;overlap_measure_acic_2016_recovery_overlap_setup.pdf

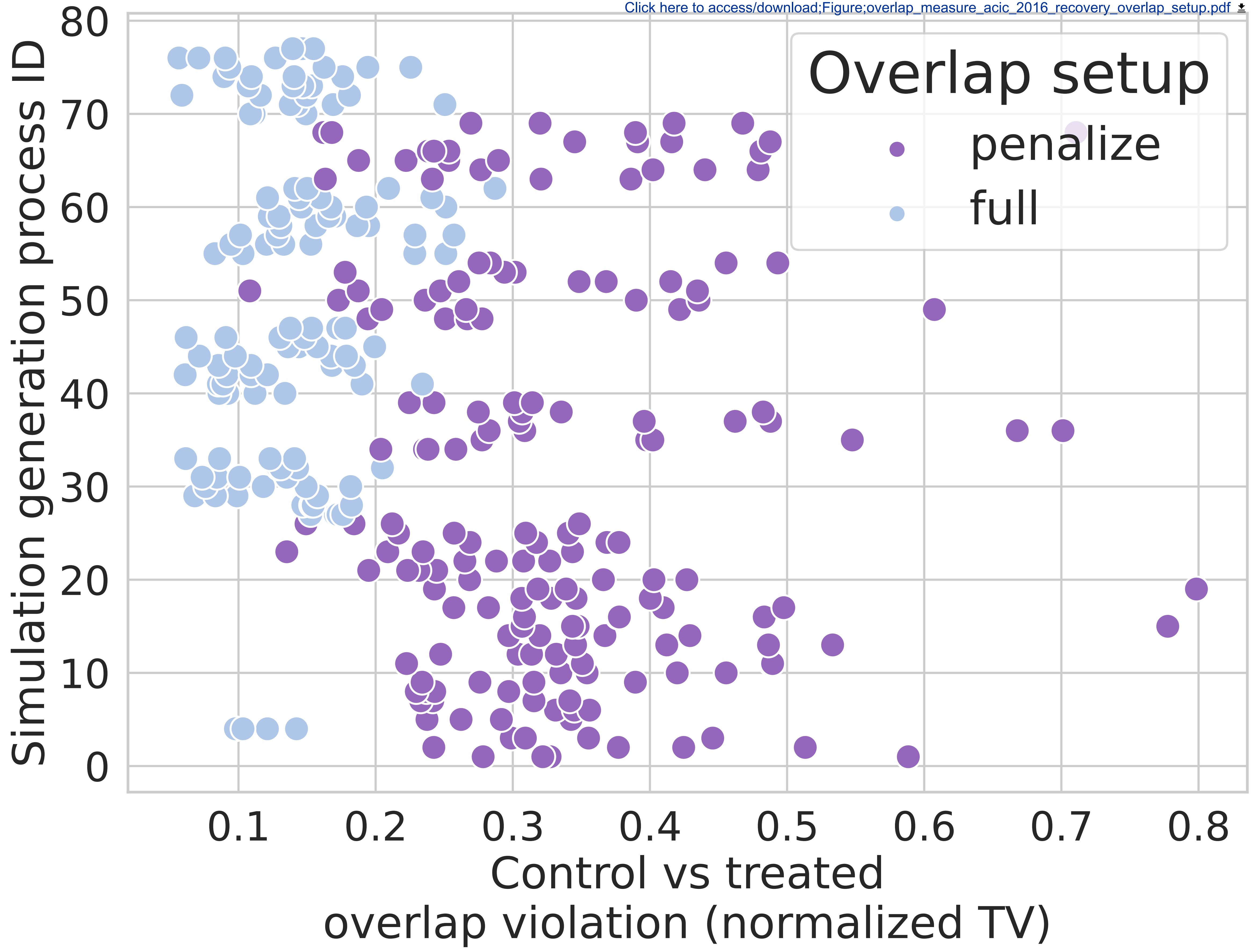

Click here to access/download;Figure;graphical_abstract.png

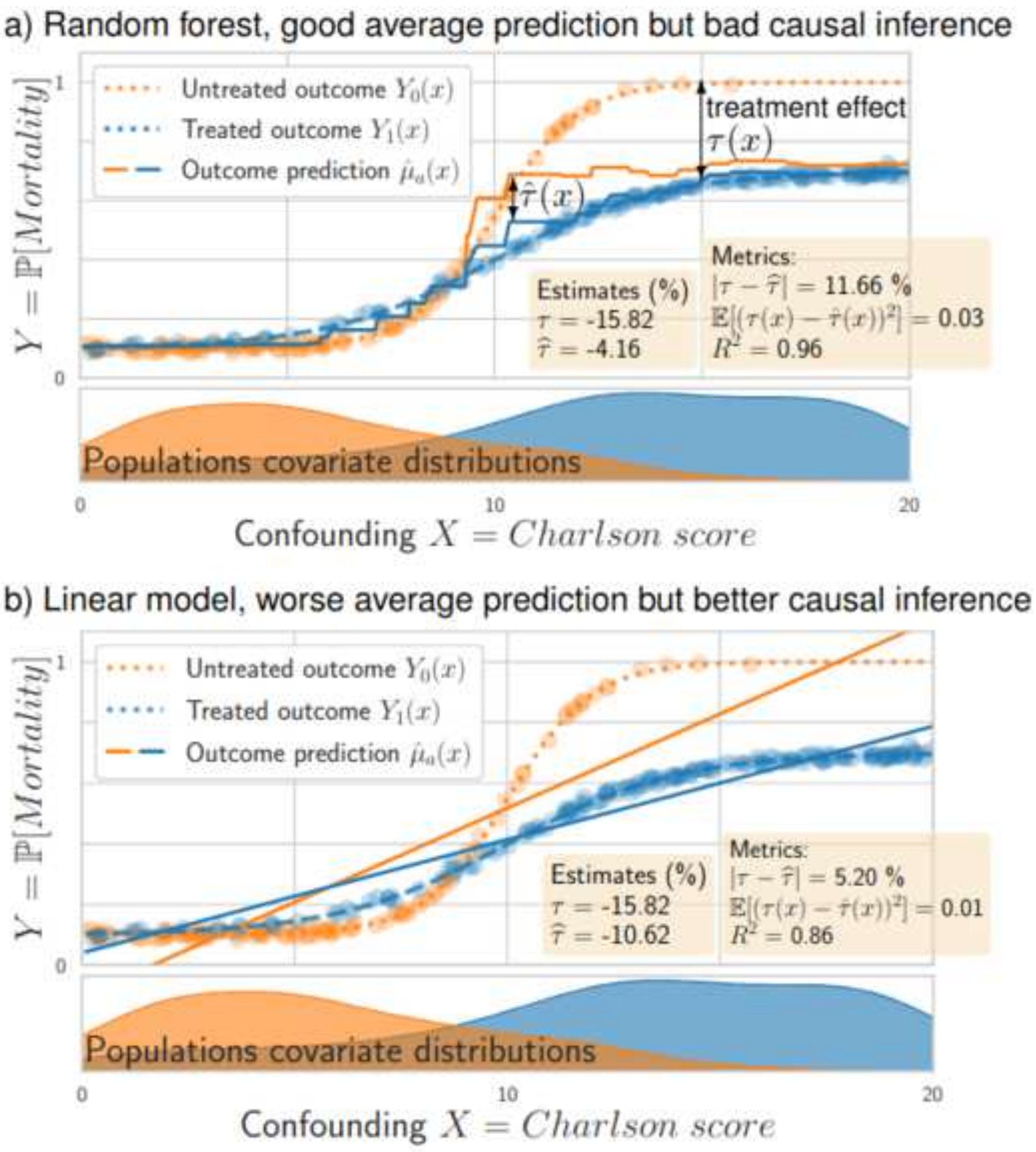

Click here to access/download;Figure;overlap_measure_twins_max_ipw_vs_ntv.pdf

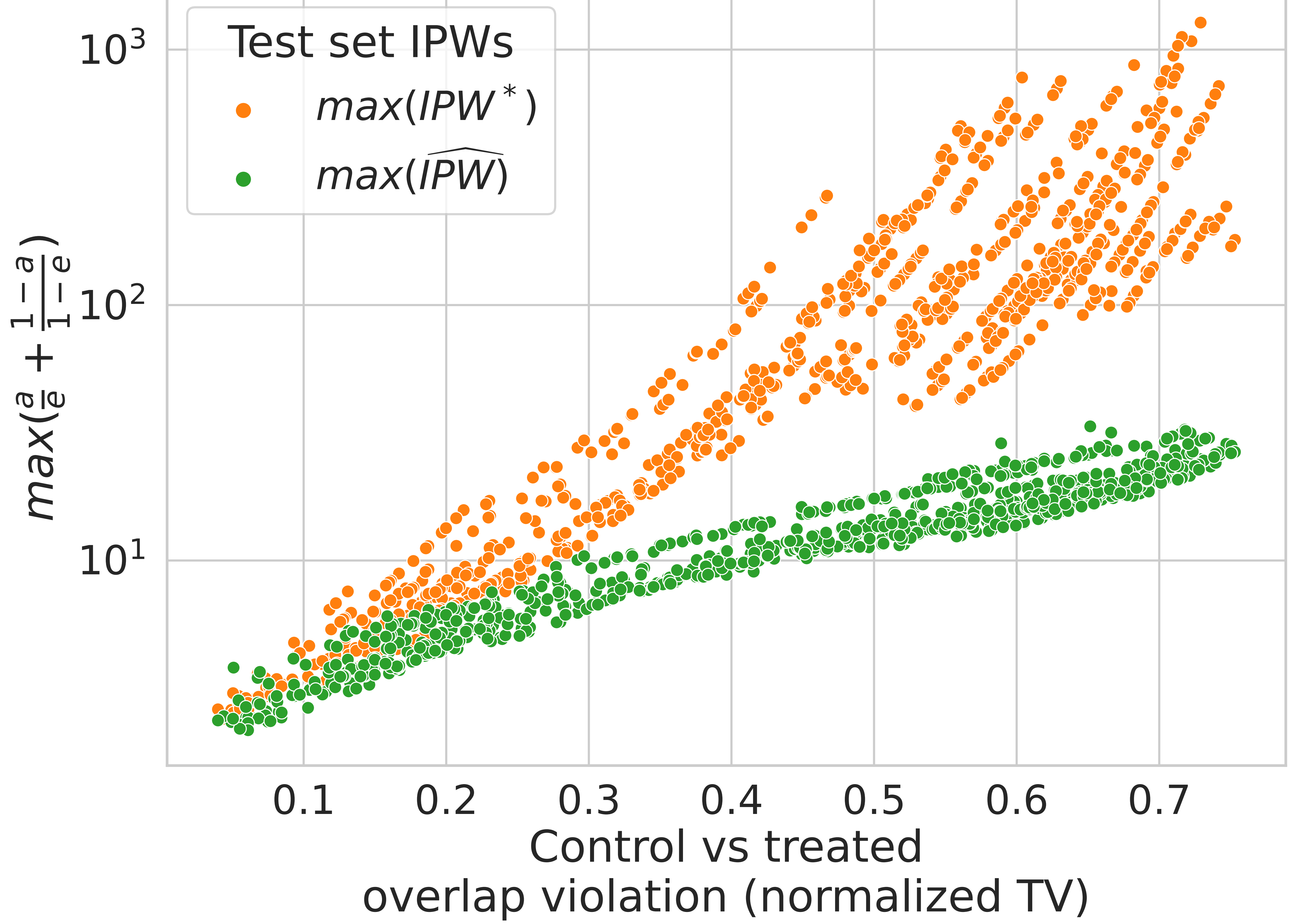

_6_effect_variance_by_bin_comparaison_kendall_by_Dataset
Click here to access/download;Figure;_6_effect_variance_by_bin_comparaison_kendall_by_Datas

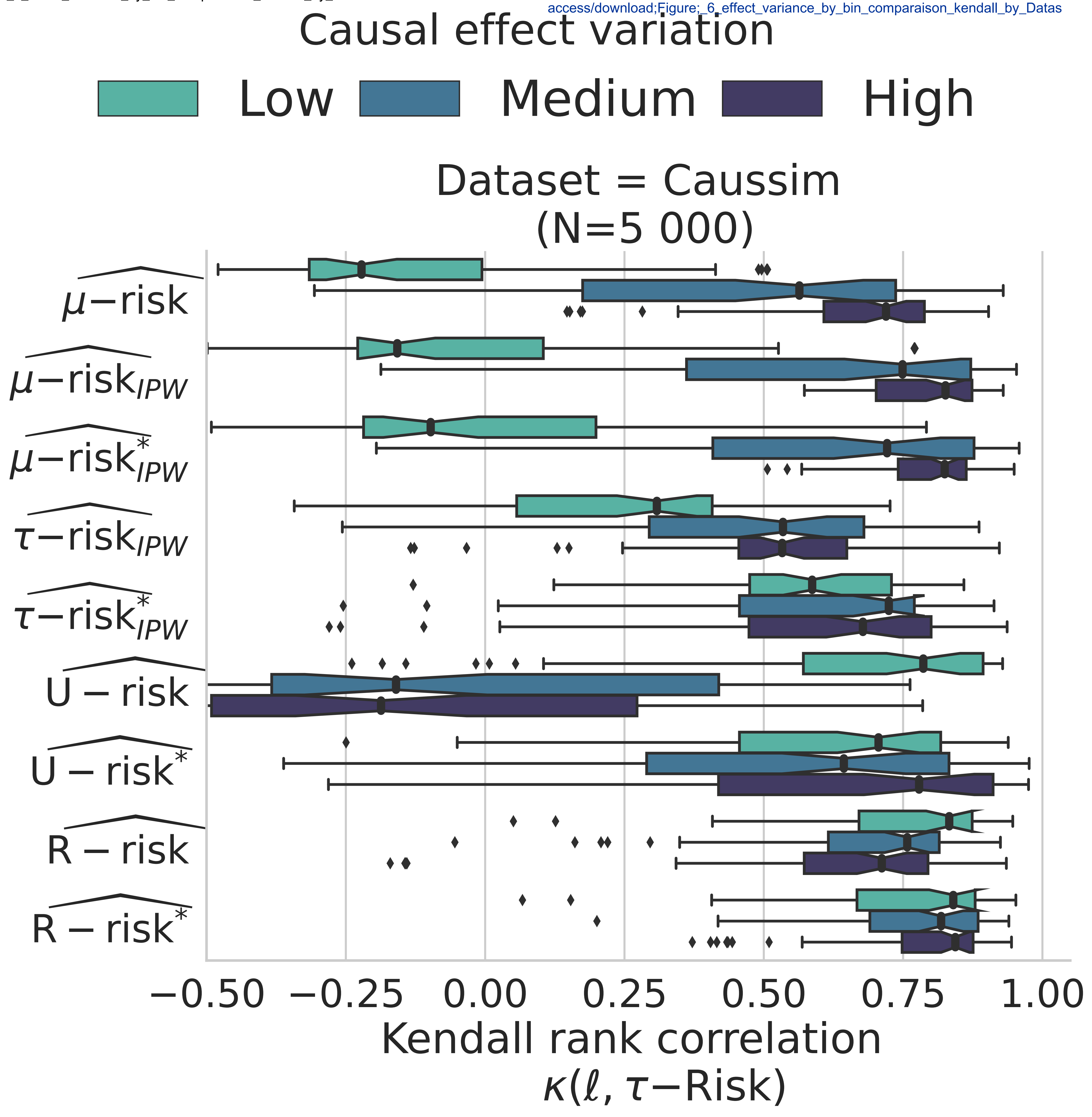

_6_effect_ratio_sym_by_bin_comparaison_kendall_by_Dataset Click here to access/download;Figure;_6_effect_ratio_sym_by_bin_comparaison_kendall_by_Datas

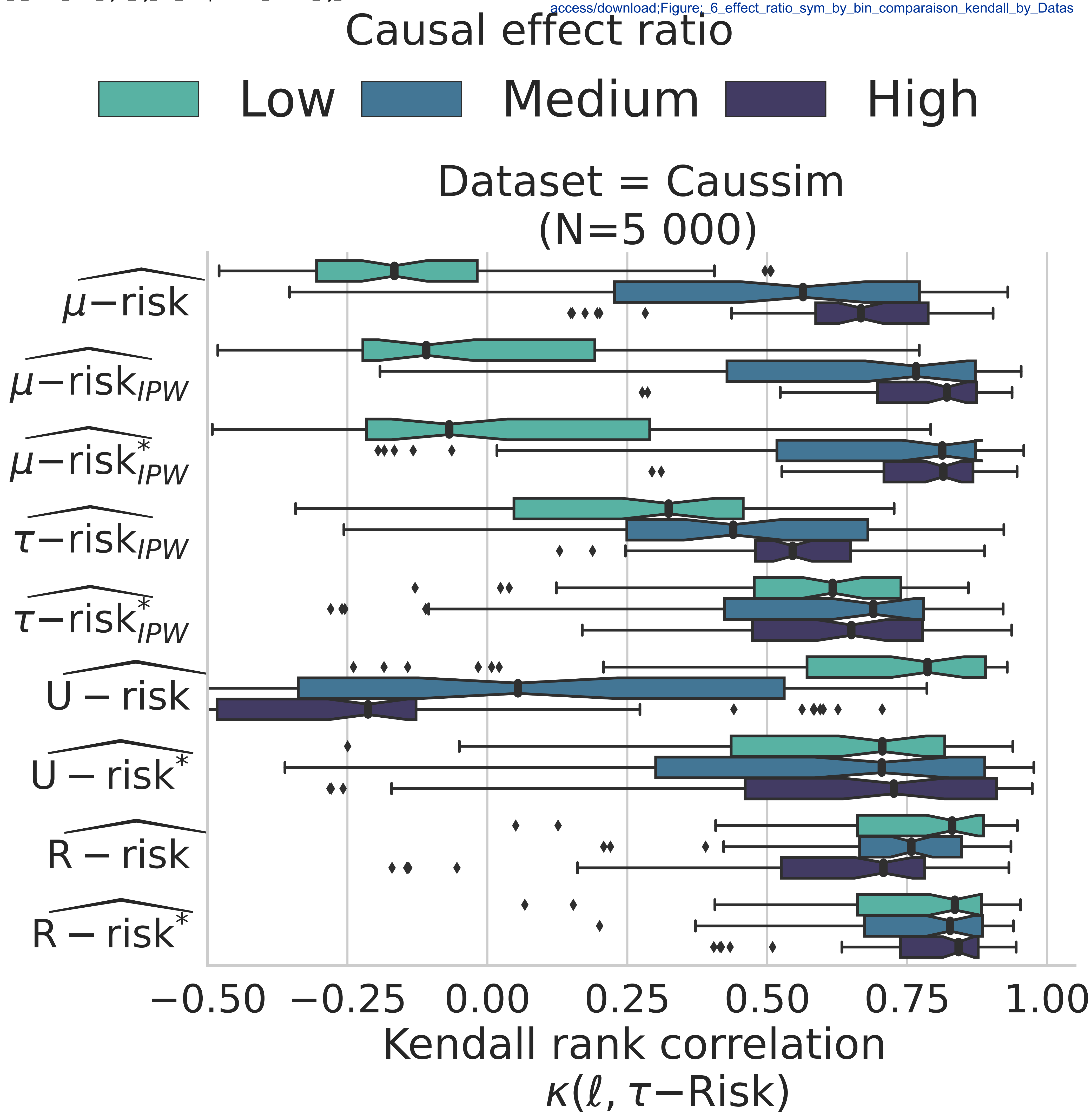

Click here to access/download
Supplementary Material
estimation_procedure_causal_selection_procedure.png.
svg

Main manuscript diff

Click here to access/download
Supplementary Material
diff.pdf

answers to reviewer

Click here to access/download
Supplementary Material
response_to_reviewer.md

Graphical Abstract

Click here to access/download;Graphical Abstract;how_to_select_graphical_abstract.png

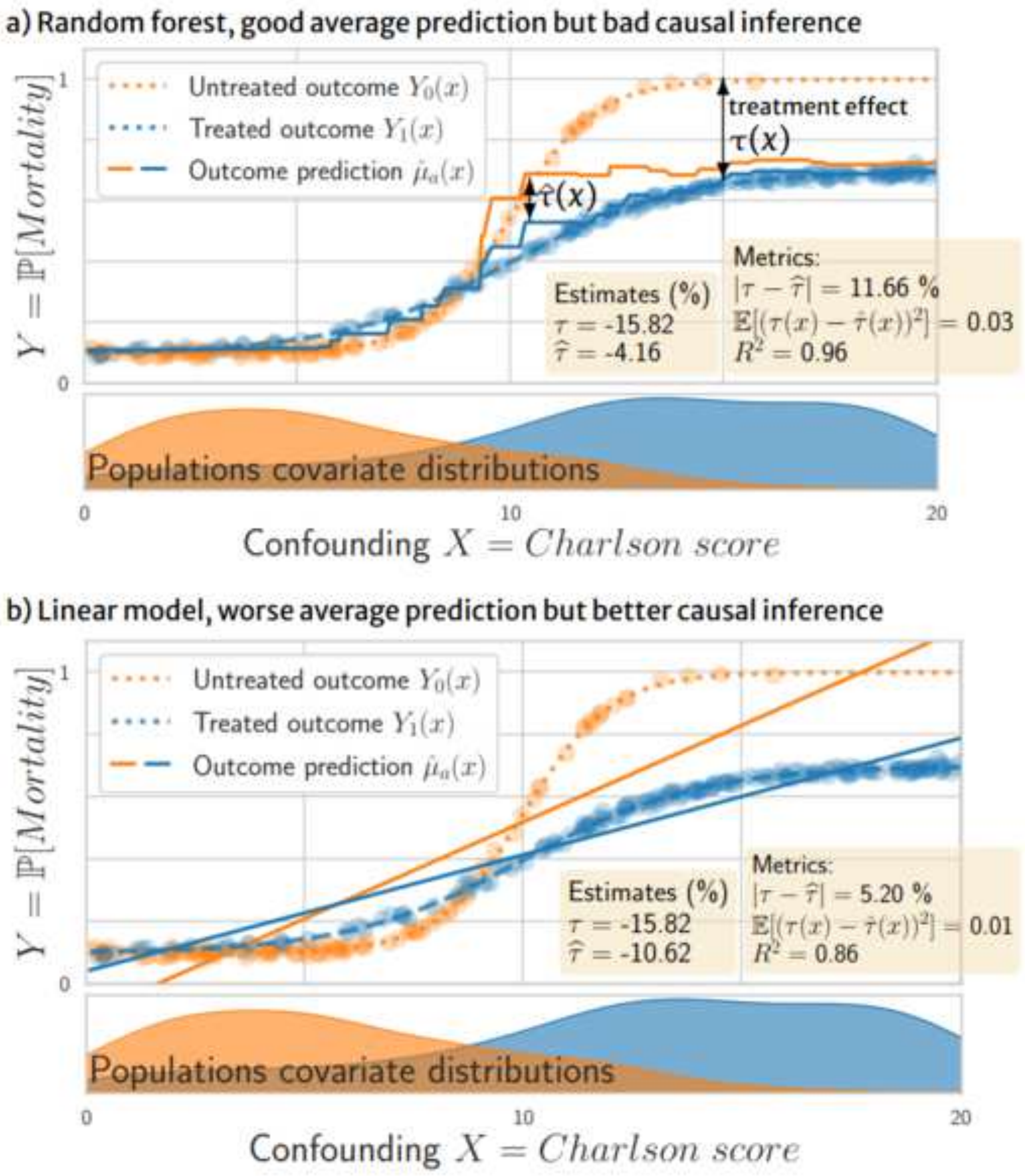

Supplement: giaf016_GIGA-D-24-00240_Revision_1 [file giaf016_giga-d-24-00240_revision_1.pdf]
