# Supplementary material for: How to select predictive models for decision-making or causal inference
Source: Gigascience. 2025 Mar 21;14:giaf016. doi: 10.1093/gigascience/giaf016 (PMC11927402; doi:10.1093/gigascience/giaf016)
Supplement: giaf016_GIGA-D-24-00240_Revision_2 [file giaf016_giga-d-24-00240_revision_2.pdf]

GigaScience

How to select predictive models for decision-making or causal inference?

--Manuscript Draft--

**Manuscript Number:**	GIGA-D-24-00240R2
**Full Title:**	How to select predictive models for decision-making or causal inference?
**Article Type:**	Research
**Funding Information:**	
**Abstract:**	Background: We investigate which procedure selects the predictive model most trustworthy to reason on the effect of an<br>intervention and support decision making.<br>Methods: We study a large variety of model selection procedures in practical settings: finite samples settings and without<br>theoretical assumption of well-specified models. Beyond standard cross-validation or internal validation procedures, we also study<br>elaborate causal risks. These build proxies of the causal error using “nuisance” re-weighting to compute it on the observed data.<br>We evaluate whether empirically estimated nuisances, which are necessarily noisy, add noise to model selection. We compare<br>different metrics for causal model selection in an extensive empirical study based on a simulation and three healthcare datasets<br>based on real covariates.<br>Results: Among all metrics, the mean squared error, classically used to evaluate predictive modes, is worse. Re-weighting it with<br>propensity score does not bring much improvements in most cases. On average, the R-risk, which uses as nuisances a model of<br>mean outcome and propensity scores, leads to the best performances. Nuisance corrections are best estimated with flexible<br>estimators such as a super learner.<br>Conclusions: When predictive models are used to reason on the effect of an intervention, they must be evaluated with different<br>procedures than standard predictive settings; using the R-risk from causal inference.
**Corresponding Author:**	Matthieu Doutreligne<br>Inria Saclay: Inria Centre de Recherche Saclay-Ile-de-France<br>Palaiseau, FRANCE
**Corresponding Author Secondary Information:**	
**Corresponding Author's Institution:**	Inria Saclay: Inria Centre de Recherche Saclay-Ile-de-France
**Corresponding Author's Secondary Institution:**	
**First Author:**	Matthieu Doutreligne
**First Author Secondary Information:**	
**Order of Authors:**	Matthieu Doutreligne
	Gaël Varoquaux
**Order of Authors Secondary Information:**	
**Response to Reviewers:**	1) Data Availability section added with references to items in the bibliography for the code and datasets<br><br>2) Removal of the footnotes and replacement with citation or in-text when approriate<br><br>3) ORCIDs added in the title page (without proper formatting)<br><br>4) additional file added as a df

Powered by Editorial Manager® and ProduXion Manager® from Aries Systems Corporation

	5) FIxed typo<br><br>6) LaTex source files added
**Additional Information:**	
**Question**	**Response**
Are you submitting this manuscript to a special series or article collection?	No
**Experimental design and statistics**<br><br>Full details of the experimental design and statistical methods used should be given in the Methods section, as detailed in our Minimum Standards Reporting Checklist. Information essential to interpreting the data presented should be made available in the figure legends.<br><br>Have you included all the information requested in your manuscript?	Yes
**Resources**<br><br>A description of all resources used, including antibodies, cell lines, animals and software tools, with enough information to allow them to be uniquely identified, should be included in the Methods section. Authors are strongly encouraged to cite Research Resource Identifiers (RRIDs) for antibodies, model organisms and tools, where possible.<br><br>Have you included the information requested as detailed in our Minimum Standards Reporting Checklist?	Yes
**Availability of data and materials**<br><br>All datasets and code on which the conclusions of the paper rely must be either included in your submission or deposited in publicly available repositories (where available and ethically appropriate), referencing such data using a unique identifier in the references and in	Yes

the “Availability of Data and Materials” section of your manuscript.<br><br>Have you have met the above requirement as detailed in our Minimum Standards Reporting Checklist?	

Powered by Editorial Manager® and ProduXion Manager® from Aries Systems Corporation

Manuscript
Click here to access/download;LaTeX - Main Document (TeX file);main_submission.tex

Placeholder for OUP logo oup.pdf

Placeholder for journal logo gigascience-logo.pdf

GigaScience, 2024, 1–10

doi: xx.xxxx/xxxx
Manuscript in Preparation
Paper

PAPER

How to select predictive models for decision-making or causal inference?

Matthieu Doutreligne (ORCID: 0000-0001-8072-9966)[1,*] and Gaël Varoquaux (ORCID: 0000-0003-1076-5122)[2]

[1]Soda, Inria Saclay, France and [2]Mission Data, Haute Autorité de Santé, France

*matt.dout@gmail.com; matthieu.doutreligne@inria.fr

Abstract

Background: We investigate which procedure selects the predictive model most trustworthy to reason on the effect of an intervention and support decision making.
Methods: We study a large variety of model selection procedures in practical settings: finite samples settings and without theoretical assumption of *well-specified* models. Beyond standard cross-validation or internal validation procedures, we also study elaborate causal risks. These build proxies of the causal error using "nuisance" re-weighting to compute it on the observed data. We evaluate whether empirically estimated nuisances, which are necessarily noisy, add noise to model selection. We compare different metrics for causal model selection in an extensive empirical study based on a simulation and three healthcare datasets based on real covariates.
Results: Among all metrics, the mean squared error, classically used to evaluate predictive modes, is worse. Re-weighting it with propensity score does not bring much improvements in most cases. On average, the R-risk, which uses as nuisances a model of mean outcome and propensity scores, leads to the best performances. Nuisance corrections are best estimated with flexible estimators such as a super learner.
Conclusions: When predictive models are used to reason on the effect of an intervention, they must be evaluated with different procedures than standard predictive settings; using the R-risk from causal inference.

Key words: Model Selection, Predictive model, Treatment Effect, G-computation, Machine Learning

Introduction

Extending prediction to prescription needs causality

Prediction models have long been used in biomedical settings, as with risk score or prognostic models [1, 2]. While these have historically been simple models on simple data, this is changing with progress in machine learning and richer medical data [3, 4]. Health predictions can now integrate medical images [5, 6, 7, 8, 9], patient records [10, 11, 12] or clinical notes [13, 14, 15]. Complex data is difficult to control and model, but these models are validated by verifying the accuracy of the prediction on left-out data [16, 17, 18]. Crucial to the clinical adoption of a model predicting a health outcome is that it "can support decisions about patient care" [19]. Precision medicine is about guiding decisions: *eg* will an individual benefit from an intervention such as surgery [20]? An estimate of the effect of the treatment can be obtained by contrasting model predictions with and without the treatment, but statistical validity requires causal inference [21, 22, 23].

Indeed, concluding on the effect of a treatment is a difficult causal-inference task, as it can be easily compromised by confounding: spurious associations between treatment allocation and baseline health, *e.g.* only prescribing a drug to mild cases [24, 25]. Predictive modeling is linked to causal inference theory by the concept of *outcome models* (or g-computation, g-estimation, g-formula [26], Q-model [21], conditional mean regression [27]). Medical statistics and epidemiology have mostly used other causal-inference methods, modeling treatment assignment with propensity scores [28, 29, 30, 31]. Outcome modeling brings the benefit of going beyond average effects, estimating individualized or

Compiled on: February 8, 2025.
Draft manuscript prepared by the author.

conditional average treatment effects (CATE), central to precision medicine. For this purpose, such methods are also invaluable for randomized trials [32, 33, 34].

Outcome-modeling methods, even when specifically designed for causal inference, are numerous: Bayesian Additive Regression Trees [35], Targeted Maximum Likelihood Estimation [36, 37], causal boosting [38], causal multivariate adaptive regression splines [38], random forests [39, 40], Meta-learners [41], R-learners [42], Doubly robust estimation [43]... The wide variety of methods raises the problem of selecting between different estimators based on the data at hand. Indeed, estimates of treatment effects can vary markedly across different predictive models [44, 45, 46, 47] (illustration in Appendix A.1).

Given complex health data, which predictive model is to be most trusted to yield valid causal estimates needed to motivate individual treatment decisions? As no single machine-learning method performs best across all datasets, there is a pressing need for clear guidelines to select outcome models for causal inference.

Objectives and structure of the paper. The intersection between machine learning and causal inference is growing rapidly [48, 49]. We focus on *model selection procedures* in practical settings, without theoretical assumptions often made in statistical literature such as *infinite* data or *well-specified* models (Appendix A.2). Asymptotic causal-inference theory recommends complex risks, but a practical question is whether model-selection procedures, that rely on data split, can estimate these risks reliably enough. Indeed, these risks come with more quantities to estimate, which may bring additional variance, leading to worse model selection.

We first illustrate the problem of causal model selection. Then we anchor causal model selection in the *potential outcome* framework and detail the causal risks and model-selection procedure. We then rewrite the so-called *R*-risk as a reweighted version of mean squared difference between the true and estimated individualized treatment effect. Finally, we conduct a thorough empirical study comparing the different metrics on diverse datasets, using a family of simulations and real health data, going beyond prior work limited to specific simulation settings [50, 51] (Appendix A.2).

Illustration: the best predictor may not estimate best causal effects

Using a predictor to reason on causal effects relies on contrasting the prediction of the outcome for a given individual with and without the treatment. Given various predictors of the outcome, which one should we use? Standard predictive modeling or machine-learning practice selects the predictor that minimizes the expected error on the outcome [17, 18]. However, this predictor may not be the best model to reason about causal effects of an intervention as Figure 1 illustrates. Consider the probability Y of an undesirable outcome (*e.g.*death), a binary treatment $A \in \{0, 1\}$, and a covariate $X \in \mathbb{R}$ summarizing the patient health status (*e.g.*the Charlson index [52]). We simulate a treatment beneficial (decreases mortality) for patients with high Charlson scores (bad health status) but with little effect for patients in good condition (low Charlson scores).

Figure 1a shows a random forest predictor with a counter-intuitive behavior: it predicts well on average the outcome (as measured by a regression R^2 score) but perform poorly to estimate causal quantities: the average treatment effect τ (as visible via the error $|\tau - \hat{\tau}|$) or the conditional average treatment effect (the error $\mathbb{E}[(\tau(x) - \hat{\tau}(x))^2]$, called CATE). On the contrary, Figure 1b shows a linear model with smaller R^2 score but better causal inference.

The problem is that causal estimation requires controlling an error on both treated and non-treated outcome for the same individual: the observed outcome, and the non-observed *counterfactual* one. The linear model is misspecified –the outcome functions are not linear–, leading to poor R^2; but it interpolates better to regions

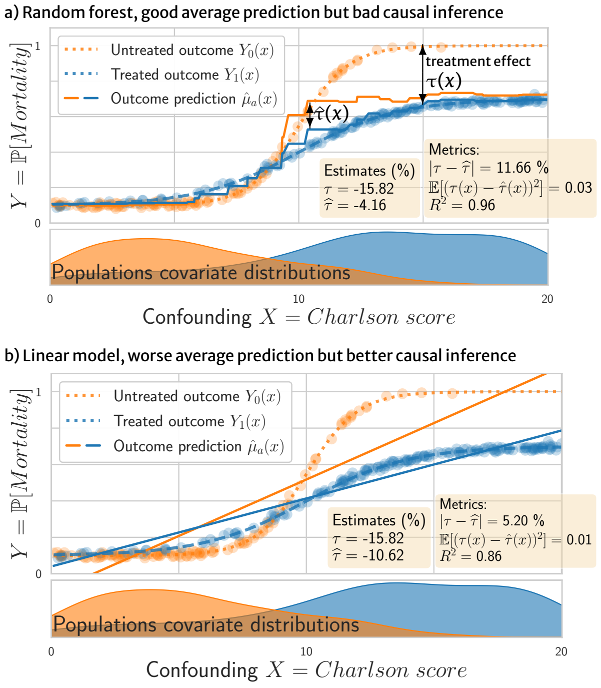

Figure 1. Illustration: a) a random-forest predictor with high performance for standard prediction (high R^2) but that yields poor causal estimates (large error between true effect τ and estimated $\hat{\tau}$), b) a linear predictor with smaller prediction performance leading to better causal estimation.

Selecting the predictor with the smallest error to the individual treatment effect $\mathbb{E}[(\tau(x) - \hat{\tau}(x))^2]$ –the τ-risk, eq. 10 – would lead to the best causal estimates; however computing this error is not feasible: it requires access to unknown quantities: $\tau(x)$.

While the random forest fits the data better than the linear model, it gives worse causal inference because its error is inhomogeneous between treated and untreated. The R^2 score does not capture this inhomogeneity.

where there are few untreated individuals –high Charlson score– and thus gives better causal estimates. Conversely, the random forest puts weaker assumptions on the data, thus has higher R^2 score but is biased by the treated population in the poor-overlap region, leading to bad causal estimates.

This toy example illustrates that the classic minimum mean squared error (MSE) criterion is not suited to choosing a model among candidate estimators for causal inference.

Methods

Neyman-Rubin Potential Outcomes framework

We first expose the classic construction of the outcome modeling (or G-computation) estimators of causal effect [53, 21, 24].

Settings. The Neyman-Rubin Potential Outcomes framework [54, 55] enables statistical reasoning on causal treatment effects: Given an outcome $Y \in \mathbb{R}$ (*e.g.*mortality risk or hospitalization length), function of a binary treatment $A \in \mathcal{A} = \{0, 1\}$ (*e.g.* a medical procedure), and baseline covariates $X \in \mathcal{X} \subset \mathbb{R}^d$, we observe the factual distribution, $O = (Y(A), X, A) \sim \mathcal{D} = \mathbb{P}(y, x, a)$. However, we want to model the existence of potential observations (unobserved ie. counterfactual) that correspond to a different treatment. Thus we want quantities on the counterfactual distribution $O^* = (Y(1), Y(0), X, A) \sim \mathcal{D}^* = \mathbb{P}(y(1), y(0), x, a)$.

Popular quantities of interest (estimands) are: at the population

level, the Average Treatment Effect

$$\text{ATE} \qquad \tau \stackrel{\text{def}}{=} \mathbb{E}_{Y(1),Y(0)\sim\mathcal{D}^*}[Y(1) - Y(0)];$$

at the individual level, to model heterogeneity, the Conditional Average Treatment Effect

$$\text{CATE} \qquad \tau(x) \stackrel{\text{def}}{=} \mathbb{E}_{Y(1),Y(0)\sim\mathcal{D}^\star}[Y(1) - Y(0)|X = x].$$

Causal assumptions. A given data needs to meet a few assumptions to enable identifying causal estimands [56]. *1)* an individual's outcome Y is solely governed by the corresponding potential outcome:

$$\textit{Consistency}\text{ assumption,} \qquad Y = A\,Y(1) + (1 - A)\,Y(0) \tag{1}$$

2) unconfoundedness $\{Y(0), Y(1)\} \perp\!\!\!\perp A|X$, *3) strong overlap* ie. every patient has a strictly positive probability to receive each treatment, and *4) generalization* –no covariate shift. These classic assumptions, called *strong ignorability*, are formally detailed in A.3.

Identifying treatment effects with outcome models – g-computation [53]. Should we know the two potential outcomes for a given X, we could compute the difference between them, which gives the causal effect of the treatment. These two potential outcomes can be estimated from observed data: the consistency and unconfoundedness A.3 assumptions imply the following equality, linking the target quantity to the observed data:

$$\mathbb{E}_{Y(a)\sim\mathcal{D}^\star}[Y(a)|X = x] = \mathbb{E}_{Y\sim\mathcal{D}}[Y|X = x, A = a] \tag{2}$$

On the left, the expectation is taken on the counterfactual unobserved distribution. On the right, the expectation is taken on the factual observed distribution conditionally on the treatment. For the rest of the paper, the expectations will always be taken on the factual observed distribution $\mathcal{D}$. This identification leads to outcome based estimators (ie. g-computation estimators [21]):

$$\begin{aligned} \tau = &\ \mathbb{E}_{Y\sim\mathcal{D}^\star}[Y(1) - Y(0)] \\ = &\ \mathbb{E}_{Y\sim\mathcal{D}}[Y|A = 1] - \mathbb{E}_{Y\sim\mathcal{D}}[Y|A = 0] \end{aligned} \tag{3}$$

This equation builds on the conditional expectation of the outcome given the treatment $\mathbb{E}_{\sim\mathcal{D}}[Y|A]$. Outcome based methods target this quantity conditionally on the covariates, called *response function*:

$$\text{Response function} \qquad \mu_a(x) \stackrel{\text{def}}{=} \mathbb{E}_{Y\sim\mathcal{D}}[Y|X = x, A = a]$$

Given a sample of data and the oracle response functions μ_0, μ_1, the finite sum version of Equation 3 leads to an unbiased estimator of the ATE written:

$$\hat{\tau} = \frac{1}{n}\left(\sum_{i=1}^{n} \mu_1(x_i) - \mu_0(x_i)\right) \tag{4}$$

This estimator is an oracle *finite sum* estimator by opposition to the population expression of τ, $\mathbb{E}[\mu_1(x_i) - \mu_0(x_i)]$, which involves an expectation taken on the full distribution $\mathcal{D}$, which is observable but requires infinite data. For each estimator ℓ taking an expectation over $\mathcal{D}$, we use the symbol $\hat{\ell}$ to note its finite sum version. The formulas in Eq. (2-4) are all partly oracle formulas: they rely on conditional expectations, the response functions, but give no specific procedures on how to compute or select them. This last point is the topic of our work, describe in the next section.

Similarly to the ATE, at the individual level Eq.2 links the CATE to statistical quantities:

$$\tau(x) = \mu_1(x) - \mu_0(x) \tag{5}$$

Robinson decomposition. G-computation is a choice of decomposition of the CATE estimation. Other choices of decomposition exist, such as the R-decomposition [57]. The latter introduces two new statistical estimates, the conditional mean outcome and the probability of being treated (known as propensity score [28]):

$$\text{Conditional mean outcome} \qquad m(x) \stackrel{\text{def}}{=} \mathbb{E}_{Y\sim\mathcal{D}}[Y|X = x] \tag{6}$$

$$\text{Propensity score} \qquad e(x) \stackrel{\text{def}}{=} \mathbb{P}[A = 1|X = x] \tag{7}$$

with these, the outcome (Eq. 1) can be written

$$\begin{aligned} \text{R-decomposition} \quad y(a) = m(x) + \big(a - e(x)\big)\tau(x) + \varepsilon(x; a) \\ \text{with} \quad \mathbb{E}[\,\varepsilon(X; A)|X, A] = 0 \end{aligned} \tag{8}$$

m and e are often called *nuisances* [43]. They are unknown and must be estimated from the data.

Both the in the ATE and CATE formula Eq. (4, 5), and the Robinson decomposition involve conditional expectations –the response functions $\mu_a(x)$ or the nuisances $m(x)$ and $e(x)$. In practice those are given by statistical models: linear models, random forests, etc [48, 49].

Model-selection, oracle and feasible risks

Causal model selection. We formalize model selection for causal estimation. Thanks to the outcome model identification (Equation 2), a given model $f : \mathcal{X} \times \mathcal{A} \to \mathcal{Y}$ –learned from data or built from domain knowledge– induces feasible estimates of the ATE and CATE (eqs 4 and 5), $\hat{\tau}_f$ and $\hat{\tau}_f(x)$. However, the g-computation framework presented above is written in terms of "perfect" conditional expectations (oracles), it does not control an error, *eg* on both populations as highlighted in Figure 1. Selection procedures are needed to find the best conditional-expectation models.

A selection procedure combines a risk ℓ, evaluating the quality of a model f with observed data O, and a splitting strategy of the data to estimate different regressions (nuisances) involved in the risk. Formally, let $\mathcal{F} = \{f : \mathcal{X} \times \mathcal{A} \to \mathcal{Y}\}$ be a family of such estimators. Our goal is to select the best candidate in this family for the observed dataset O using a risk ℓ:

$$f^*_\ell = \underset{f\in\mathcal{F}}{\text{argmin}}\ \ell(f, O) \tag{9}$$

We now detail possible risks ℓ, risks useful for causal model selection, and how to compute them.

The τ-risk: an oracle error risk. As we would like to target the CATE, the following evaluation risk is natural (also called PEHE [59, 35]):

$$\tau\text{-risk}(f) \stackrel{\text{def}}{=} \mathbb{E}_{X\sim p(X)}[(\tau(X) - \hat{\tau}_f(X))^2] \tag{10}$$

Given observed data from $p(X)$, the expectation is computed with a finite sum, as in eq. 4, to give an estimated value $\widehat{\tau\text{-risk}}(f)$. However this risk is not feasible as the oracles $\tau(x)$ are not accessible with the observed data $(Y, X, A) \sim \mathcal{D}$.

Feasible error risks. Table 1 lists *feasible* risks (Detailed in Appendix A.4), based on the prediction error of the outcome model and *observable* quantities. These observable, called nuisances are e – propensity score, eq 7– and m –conditional mean outcome, eq 6. We give the definitions as *semi-oracles*, function of the true unknown nuisances, but later instantiate them with estimated nuisances, noted $(\check{e}, \check{m})$. Semi-oracles risks are superscripted with the * symbol.

Table 1. Review of causal risks — The R-risk* is called τ-risk$_R$ in [50].

Risk	Equation	Reference
τ-risk = MSE($\tau(X), \tau_f(X)$)	$\mathbb{E}_{X\sim p(X)}[(\tau(X) - \hat{\tau}_f(X))^2]$	Eq. 10 [35]
μ-risk = MSE($Y, f(X)$)	$\mathbb{E}_{(Y,X,A)\sim\mathcal{D}}\left[(Y - f(X;A))^2\right]$	[50]
μ-risk$^*_{IPW}$	$\mathbb{E}_{(Y,X,A)\sim\mathcal{D}}\left[\left(\frac{A}{e(X)} + \frac{1-A}{1-e(X)}\right)(Y - f(X;A))^2\right]$	[58]
τ-risk$^\star_{IPW}$	$\mathbb{E}_{(Y,X,A)\sim\mathcal{D}}\left[\left(Y\left(\frac{A}{e(X)} - \frac{1-A}{1-e(X)}\right) - \hat{\tau}_f(X)\right)^2\right]$	[39]
U-risk*	$\mathbb{E}_{(Y,X,A)\sim\mathcal{D}}\left[\left(\frac{Y-m(X)}{A-e(X)} - \hat{\tau}_f(X)\right)^2\right]$	[42]
R-risk*	$\mathbb{E}_{(Y,X,A)\sim\mathcal{D}}\left[\left((Y - m(X)) - (A - e(X))\,\hat{\tau}_f(X)\right)^2\right]$	[42]

Model selection procedure

Causal model selection (eq 9) may involve estimating various quantities from the observed data: the outcome model f, its induced risk as introduce in the previous section, and possibly nuisances required by the risk. Given a dataset with N samples, we split out a train and a test sets $(\mathcal{T}, \mathcal{S})$. We fit each candidate estimator $f \in \mathcal{F}$ on $\mathcal{T}$. We also fit the nuisance models $(\check{e}, \check{m})$ on the train set $\mathcal{T}$, setting hyperparameters by a nested cross-validation before fitting the nuisance estimators with these parameters on the full train set. Causal quantities are then computed by applying the fitted candidates estimators $f \in \mathcal{F}$ on the test set $\mathcal{S}$. Finally, we compute the model-selection metrics for each candidate model on the test set. This procedure is described in Algorithm 1 and Figure 2.

Algorithm 1 Model selection procedure

Given train and test sets $(\mathcal{T}, \mathcal{S}) \sim \mathcal{D}$, a candidate estimator f, a causal metrics ℓ:

i. Prefit: Learn estimators for unknown nuisance quantities $(\check{e}, \check{m})$ on the training set $\mathcal{T}$
ii. Fit: learn $\hat{f}(\cdot, a)$ on $\mathcal{T}$
iii. Model selection: $\forall x \in \mathcal{S}$ predict $\big(\hat{f}(x, 1), \hat{f}(x, 0)\big)$ and evaluate the estimator storing the metric value: $\ell(f, \mathcal{S})$ – possibly function of $\check{e}$ and $\check{m}$

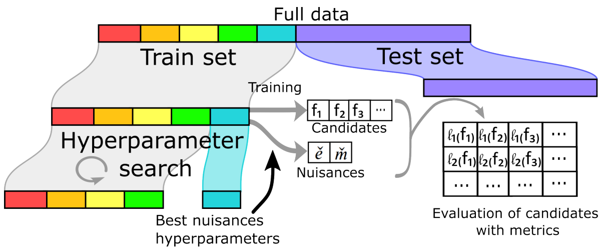

Figure 2. Estimation procedure for causal model selection.

R-risk as reweighted oracle metric

The R-risk can be rewritten as a rebalanced τ-risk.

This rewriting involves reweighted residuals: for each potential outcome, $a \in \{0;1\}$, the variance conditionally on x is [60]:

$$\sigma_y^2(x;a) \stackrel{\text{def}}{=} \int_y (y - \mu_a(x))^2\, p(y \mid x = x; A = a)\, dy$$

Integrating over the population, we get the Bayes squared error: $\sigma_B^2(a) = \int_{\mathcal{X}} \sigma_y^2(x;a) p(x) dx$ and its propensity weighted version: $\tilde{\sigma}_B^2(a) = \int_{\mathcal{X}} \sigma_y^2(x;a)\, p(x;a)\, dx$. In case of a purely deterministic link between the covariates, the treatment, and the outcome, these residual terms are null.

Proposition 1 **(R-risk as reweighted τ-risk)** *Given an outcome model f, its R-risk appears as weighted version of its τ-risk (Proof in Appendix A.5):*

$$\begin{aligned} R\text{-risk}^*(f) = &\int_x e(x)(1 - e(x))\,(\tau(x) - \tau_f(x))^2 p(x) dx \\ &+ \tilde{\sigma}_B^2(1) + \tilde{\sigma}_B^2(0) \end{aligned} \quad (11)$$

The R-risk targets the oracle at the cost of an overlap re-weighting and the addition of the reweighted Bayes residuals, which are independent of f. In good overlap regions the weights $e(x)(1 - e(x))$ are close to $\frac{1}{4}$, hence the R-risk is close to the desired gold-standard τ-risk. For randomized control trials, this weight is constant making the R-risk particularly suited for exploring heterogeneity (Appendix A.5)

Empirical Study

We evaluate the following causal metrics, oracle and feasible versions, presented in Table 1:
$\widehat{\mu\text{-risk}_{IPW}}^*$, $\widehat{R\text{-risk}}^*$, $\widehat{U\text{-risk}}^*$, $\widehat{\tau\text{-risk}_{IPW}}^*$, $\widehat{\mu\text{-risk}}$, $\widehat{\mu\text{-risk}_{IPW}}$, $\widehat{R\text{-risk}}$, $\widehat{U\text{-risk}}$, $\widehat{\tau\text{-risk}_{IPW}}$. We benchmark the metrics in a variety of settings: many different simulated data generation processes and three semi-simulated datasets.We provide scripts for the simulations [61] and the selection procedure [62].

The simulations, designed to evaluate the effect of the overlap parameter, also explore more diverse and noisy covariate distributions. They cover a diversity of causal settings such as different ratio of causal effect to background responses, and functional links between covariates, outcome and treatment.

Caussim: Extensive simulation settings

Data Generation. We use simulated data, on which the ground-truth causal effect is known. Going beyond prior empirical studies of causal model selection [50, 51], we use many generative processes, which is needed to reach general conclusions (Appendix A.7).

We generate the response functions using random bases extension, a common method in biostatistics, *e.g.*functional regression with splines [63, 64]. By allowing the function to vary at specific knots, we control the complexity of the non-linear outcome models. We use random approximation of Radial Basis Function (RBF) kernels [65] to generate the outcome and treatment functions. RBF use the same process as polynomial splines but replace polynomial by Gaussian kernels. Unlike polynomial, Gaussian kernels have decreasing influences in the input space. This avoids unrealistic divergences of the functions at the ends of the feature space. We generate 1 000 datasets based on these functions, with random overlap parameters. Example shown in Appendix A.7, Fig 13. and details in Appendix A.7.

Family of candidate estimators. We test model selection across different candidate estimators that approximate imperfectly the data-generating process. To build such estimators, we first use an RBF expansion similar to that used for data generation. We choose two random knots and transform the raw data features with a Gaussian kernel. This step is referred as the featurization. Then, we fit a linear regression on these transformed features. We consider two ways of combining these steps for outcome model; we use common nomenclature [41, 66] to refer to these different meta-learners that differ on how they model, jointly or not, the treated and the non treated:

- SLearner: A single learner for both populations, taking the treatment as a supplementary covariate.
- SftLearner: A single set of basis functions is sampled at random for both populations, leading to a given feature space used to model both the treated and controls, then two separate different regressors are fitted on this shared representation.
- TLearner: Two completely different learners for each population, hence separate feature representations and regressors.

For the regression step, we fit a Ridge regression on the transformed features with 6 different choices of the regularization parameter $\lambda \in [10^{-3}, 10^{-2}, 10^{-1}, 1, 10^{1}, 10^{2}]$, coupled with a TLearner or a SftLearner. We sample 10 different random basis for learning and featurization yielding a family $\mathcal{F}$ of 120 candidate estimators.

Semi-simulated datasets

Datasets. We also use three semi-simulated data adding a known synthetic causal effect to real –non synthetic– healthcare covariate. ACIC 2016 [45] is based on the Collaborative Perinatal Project [67], a RCT studying infants' developmental disorders containing 4,802 indivduals and 55 features. We used 770 dataset instances: 10 random seeds for each of the 77 simulated settings for the treatment and outcomes. ACIC 2018 [68] simulated treatment and outcomes for the Linked Births and Infant Deaths Database (LBIDD) [69] with $D = 177$ covariates. We used all 432 datasets of size $N = 5\,000$. Twins [70] is an augmentation of real data on twin births and mortality rates [71]. There are $N = 11\,984$ samples, and $D = 50$ covariates for which we simulated 1,000 different treatment allocations. Appendix A.7 gives datasets details.

Family of candidate estimators. For these three datasets, the family of candidate estimators are gradient boosting trees for both the response surfaces and the treatment (scikit-learn HistGradientBoostingRegressor HistGradientBoostingClassifier [72]) with S-learner, learning rate in {0.01, 0.1, 1}, and maximum number of leaf nodes in {25, 27, 30, 32, 35, 40} resulting in a family of size 18.

Nuisance estimators. Drawing from the TMLE literature that uses combination of flexible machine learning methods [37], we model the nuisances $\check{e}$ (respectivley $\check{m}$) with a meta-learner: a stacked estimator of ridge and boosting classifiers (respectively regressions) (hyperparameter selection in Appendix A.7).

Measuring overlap between treated and non treated

Good overlap between treated and control population is crucial for causal inference (Assumption A.3). We introduce the Normalized Total Variation (NTV), a divergence based on the propensity score summarizing the overlap between both populations (Appendix A.6).

Results: factors driving good model selection

The R-risk is the best metric on average. Figure 3 shows the agreement between the ideal ranking of outcome models given the oracle τ-risk and the different feasible causal metrics. We measure this agreement with relative Kendall tau κ (see Appendix A.7, eq. 20) [73]. To remove the variance across datasets (some datasets lead to easier model selection than others), we report values for one metric relative to the mean of all metrics for a given dataset instance: Relative $\kappa(\ell, \tau\text{-risk}) = \kappa(\ell, \tau\text{-risk}) - mean_{\ell}(\kappa(\ell, \tau\text{-risk}))$. Given the importance of overlap in how well metrics approximate the oracle τ-risk, we separate strong and weak overlap.

Among all metrics, the classical mean squared error (ie. factual μ-risk) is worse and reweighting it with propensity score ($\mu\text{-risk}_{IPW}$) does not bring much improvements. The R-risk, which includes a model of mean outcome and propensity scores, leads to the best performances. Interestingly, the U-risk, which uses the same nuisances, deteriorates in weak overlap, probably due to variance inflation when dividing by extreme propensity scores.

Beyond rankings, the differences in terms of absolute ability to select the best model are large: The R-risk selects a model with a τ-risk only 1% higher than the best possible candidate for strong overlap on Caussim, but selecting with the μ-risk or $\mu\text{-risk}_{IPW}$ –as per machine-learning practice– leads to 10% excess risk and using $\tau\text{-risk}_{IPW}$ –as in some causal-inference methods [74, 75]–leads to 100% excess risk (Appendix A.7, Figure 16). Across datasets, the R-risk consistently decreases the risk compared to the μ-risk: from 0.1% to 1% on ACIC2016, 1% from to 20% on ACIC2018, and 0.05% from to 1% on Twins.

Model selection is harder for low population overlap. Model selection for causal inference becomes more and more difficult with increasingly different treated and control populations (Figure 4). The absolute Kendall's coefficient correlation with τ-risk drops from 0.9 (excellent agreement with oracle selection) to 0.6 on both Caussim and ACIC 2018 (Appendix A.7, Figure 15).

Nuisances can be estimated on the same data as outcome models. Using the train set $\mathcal{T}$ both to fit the candidate estimator and the nuisance estimates is a form of double dipping which can lead errors in nuisances correlated to that of outcome models [42]. In theory, these correlations can bias model selection and, strictly speaking, push to split out a third separated dataset –a "nuisance set"– to

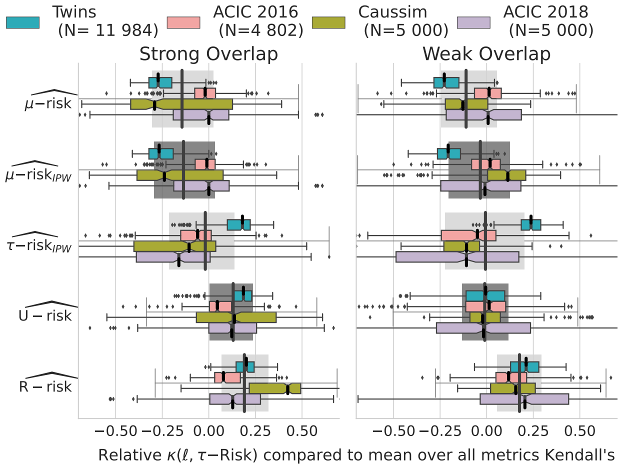

Figure 3. The *R*-risk is the best metric: Relative Kendall's τ agreement with τ-risk. Strong and Weak overlap correspond to the first and last tertiles of the overlap distribution measured with Normalized Total Variation (Appendix A.6). Appendix A.7 presents the same results by adding semi-oracle risks in Figure 14, measured with absolute Kendall's in Figure 15 and with τ−risk gains in Figure 16. Table 4 gives median and IQR of the relative Kendall.

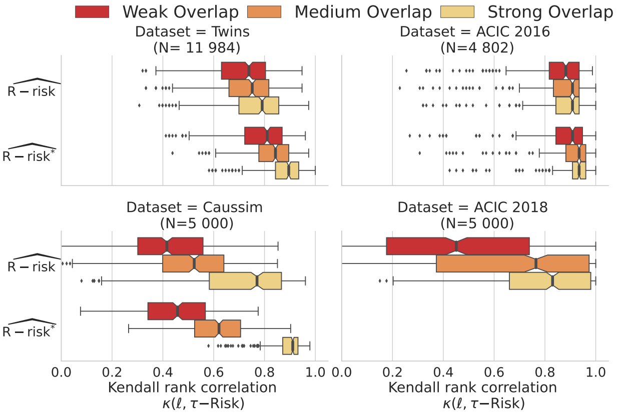

Figure 4. Model selection is harder for low population overlap: Kendall's τ agreement with τ-risk. Strong, medium and Weak overlap are the tertiles of the overlap measured with NTV (Appendix A.6, eq. 17). Appendix A.7 presents results for all metrics in Figure 18 in absolute Kendall's and continuous overlap values in Figure 15.

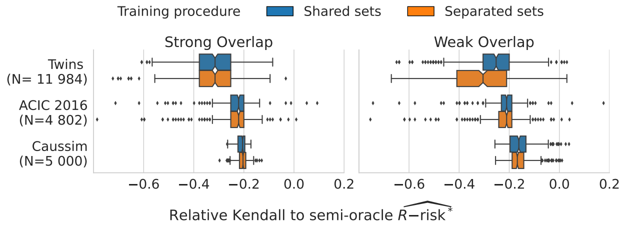

Figure 5. Nuisances can be estimated on the same data as outcome models: Results for the R-risk are similar between the shared nuisances/candidate set and the separated nuisances set procedures. Appendix A.7 Figure 17 details results for all metrics.

fit the nuisance models. The drawback is that it depletes the data available for model estimation and selection. However, Figure 5 shows no substantial difference between a procedure with a separated nuisance set and the simpler shared nuisance-candidate set procedure.

Empirically, the best split is 90 %/10 %: using 90 % of the data to estimate both the nuisances and candidates, then computing the risks on the remaining test set for model selection (experiments in Appendix A.8).

Stacked models are good overall estimators of nuisances. Stacked nuisances estimators (boosting and linear) lead to feasible metrics with close performances to the oracles ones: the corresponding estimators recover well-enough the true nuisances. One may wonder if simpler models for the nuisance could be useful, in particular in data-poor settings or when the true models are linear. Figure 6 compares causal model selection estimating nuisances with stacked estimators or linear model. It comprises the Twins data, where the true propensity model is linear, and a downsampled version of this data, to study a situation favorable to linear models. In these settings, stacked and linear estimations of the nuisances performs equivalently. Detailed analysis (Appendix A.7 Figure 20) confirms that using adaptive models –as built by stacking linear models and gradient-boosted trees– suffices to estimate nuisance.

R-risk is robust to a wide range of effect ratio values. Beyond overlap, we study for caussim simulations, the effect on model selection of different causal effect ratio to baseline. We vary the empirical mean absolute difference between the causal effect and the baseline, $\Delta_\mu = \frac{1}{N}\sum_{i=1}^{N}\left|\frac{\mu_1(x_i)-\mu_0(x_i)}{\mu_0(x_i)+\mu_1(x_i)-\frac{1}{N}\sum_{j=1}^{N}\mu_0(x_j)+\mu_1(x_j)}\right|$, covering a ratio range from 0.04 to 206 (median = 9.1). Appendix A.9 details this

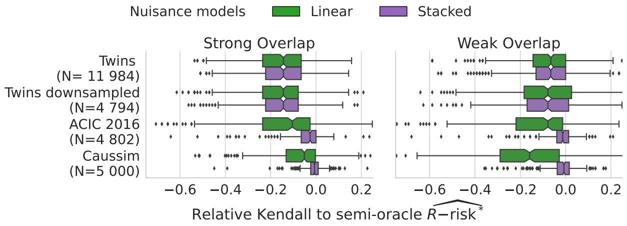

Figure 6. Stacked models are good overall estimators of the nuisances: Results are shown only for the R-risk; A.7 Figure 19 details every metrics. For Twins, where the true propensity model is linear, stacked and linear estimations of the nuisances performs equivalently, even for a downsampled version (N=4,794).

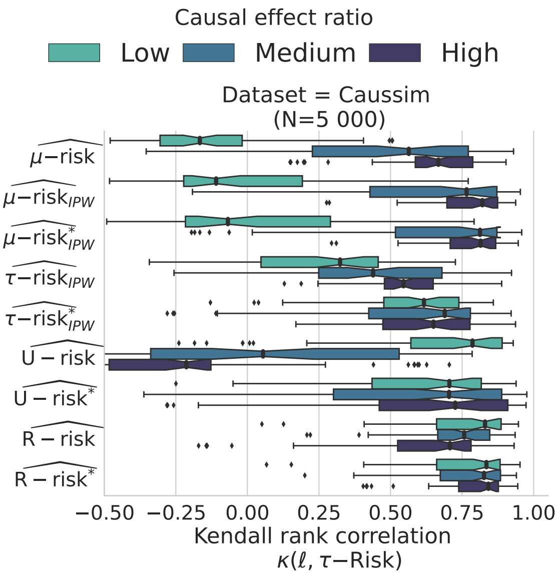

Figure 7. R-risk is robust to a wide range of effect ratio: Kendall's τ agreement with τ-risk. Strong, medium and Weak Causal effect ratio are the tertiles of the absolute ratio causal effect to baseline response, Δ_μ: Low [0.04; 2.86[, Medium [2.86; 16.65[, High [16.65; 206.53[. Appendix A.9 details this simulation.

setup as well as an alternive measure of effect ratio. Figure 7 shows that for high values of the ratio, $R-$ risk is outperformed by the μ-risk$_{IPW}$ and the τ-risk$_{IPW}$. However, on average, the $R-$ risk is still the better risk.

Discussion and conclusion

Nuisance models: more gain than pain. Predictive models are increasingly used to reason about treatment effects, for instance in precision medicine to drive individualized decision. Our results highlight that they should be selected, validated, and tuned using different procedures and error measures than those classically used to assess prediction. Rather, selecting the best outcome model according to the R-risk (Appendix A.4 eq. Definition 5) leads to more valid causal estimates on average. Estimating the R-risk requires a more complex procedure than standard cross-validation used *e.g.*in machine learning: it involves fitting nuisance models necessary for model evaluation. Our results show that these can be learned on the same set of data as the outcome models evaluated. The nuisance models must be well estimated (Figure 6). Our results show that using for nuisance models a flexible stacking-based family of estimator suffices for good model selection. To select propensity score models, we used the Brier score, minimized by the true individual probability. An easy mistake is to use calibration errors popular in machine learning [76, 77, 78, 79] as these select not for the individual posterior probability but for an aggregate error rate

[80].

More R-risk to select models driving decisions. Increasingly complex prediction models integrating richer medical data have flourished because their predictions can be easily demonstrated and validated on left-out data. But using them to underpin a decision on whether to treat or not requires more careful validation, using a metric accounting for the putative intervention, the R-risk. On average, the R-risk brings a sizeable benefit to select the most adequate model, even when model development is based on treated and untreated population with little differences, as in RCTs. Our conclusions are that without prior knowledge, the R-risk is a good default. However, there is much remaining variation, and the R-risk will not be optimal for every situation. We have identified one such specific situation: when the causal effect is large compared to the variation of the baseline effect, the μ-risk$_{IPW}$ performs slightly better.

To facilitate better model selection, we provide Python code [62]. This model-selection procedure puts no constraints on the models used to build predictive models: it opens the door to evaluating a wide range of models, from gradient boosting to convolutional neural network, or language models.

Availability of source code and requirements

Source code to replicate the experiments of the paper

The following repository is self-contained to allow anyone to replicate all experiments of the paper. It contains different codes to cover the full content of the paper. Thus it requires a bit of work to install and run the code.

- Project name: Caussim
- Project home page: [61]
- Operating system(s): Platform independent
- Programming language: Python
- License: BSD 3-Clause License

Standalone source code for the selection procedure

Code for cross-validation to select the best predictive model to guide decision on potential intervention. The procedure implemented here is described in details in Algorithm 1. It serves as a simple plug-and-play code for a user that would want to use our procedure.

- Project name: Causal Model Selection
- Project home page: [62]
- Operating system(s): Platform independent
- Programming language: Python
- License: BSD 2-Clause License

Data availability

Semi-simulated datasets for the experiments

The semi-simulated and simulated datasets used for the experiments are available at the following urls.

Detailed explanations on how to generate the datasets are available in the readme of the data section of our github [61].

- ACIC 2016: dataset provided through R package aciccomp2016 [45]
- ACIC 2018: scaling subset from the Synapse repository : [81]
- TWINS: dataset from [70], our version is on a zenodo repository [82].
- Caussim Dataset: generated dataset with the source code [61].

Experiments result data

The result data generated by the experiments are available at a dedicated zenodo repository [83]. We provide the data for the simulations and the semi-simulated datasets to allow an easy replication of the main graphic (Fig. 3) in the result section.

References

1. Karel GM Moons, Patrick Royston, Yvonne Vergouwe, Diederick E Grobbee, and Douglas G Altman. Prognosis and prognostic research: what, why, and how? *Bmj*, 338, 2009.
2. Ewout W Steyerberg. *Clinical prediction models*. Springer.
3. Andrew L Beam and Isaac S Kohane. Big data and machine learning in health care. *Jama*, 319(13):1317–1318, 2018.
4. Alvin Rajkomar, Jeffrey Dean, and Isaac Kohane. Machine learning in medicine. *New England Journal of Medicine*, 380 (14):1347–1358, 2019.
5. M Khojaste-Sarakhsi, Seyedhamidreza Shahabi Haghighi, SMT Fatemi Ghomi, and Elena Marchiori. Deep learning for alzheimer's disease diagnosis: A survey. *Artificial Intelligence in Medicine*, page 102332, 2022.
6. Zhenwei Zhang and Ervin Sejdić. Radiological images and machine learning: trends, perspectives, and prospects. *Computers in biology and medicine*, 108:354–370, 2019.
7. Adam Yala, Constance Lehman, Tal Schuster, Tally Portnoi, and Regina Barzilay. A deep learning mammography-based model for improved breast cancer risk prediction. *Radiology*, 292(1):60–66, 2019.
8. Li Shen, Laurie R Margolies, Joseph H Rothstein, Eugene Fluder, Russell McBride, and Weiva Sieh. Deep learning to improve breast cancer detection on screening mammography. *Scientific reports*, 9(1):12495, 2019.
9. Ali Bou Nassif, Manar Abu Talib, Qassim Nasir, Yaman Afadar, and Omar Elgendy. Breast cancer detection using artificial intelligence techniques: A systematic literature review. *Artificial Intelligence in Medicine*, page 102276, 2022.
10. Stephen J Mooney and Vikas Pejaver. Big data in public health: terminology, machine learning, and privacy. *Annual review of public health*, 39:95, 2018.
11. Rishi J Desai, Shirley V Wang, Muthiah Vaduganathan, Thomas Evers, and Sebastian Schneeweiss. Comparison of machine learning methods with traditional models for use of administrative claims with electronic medical records to predict heart failure outcomes. *JAMA network open*, 3(1):e1918962–e1918962, 2020.
12. Gregory E Simon, Eric Johnson, Jean M Lawrence, Rebecca C Rossom, Brian Ahmedani, Frances L Lynch, Arne Beck, Beth Waitzfelder, Rebecca Ziebell, Robert B Penfold, et al. Predicting suicide attempts and suicide deaths following outpatient visits using electronic health records. *American Journal of Psychiatry*, 175(10):951–960, 2018.
13. Steven Horng, David A Sontag, Yoni Halpern, Yacine Jernite, Nathan I Shapiro, and Larry A Nathanson. Creating an automated trigger for sepsis clinical decision support at emergency department triage using machine learning. *PloS one*, 12(4): e0174708, 2017.
14. Hanyin Wang, Yikuan Li, Seema A Khan, and Yuan Luo. Prediction of breast cancer distant recurrence using natural language processing and knowledge-guided convolutional neural network. *Artificial intelligence in medicine*, 110:101977, 2020.
15. Irena Spasic, Goran Nenadic, et al. Clinical text data in machine learning: systematic review. *JMIR medical informatics*, 8(3): e17984, 2020.
16. Douglas G Altman, Yvonne Vergouwe, Patrick Royston, and Karel GM Moons. Prognosis and prognostic research: validating a prognostic model. *Bmj*, 338, 2009.

17. Russell A Poldrack, Grace Huckins, and Gael Varoquaux. Establishment of best practices for evidence for prediction: a review. *JAMA psychiatry*, 77(5):534–540, 2020.
18. Gaël Varoquaux and Olivier Colliot. Evaluating machine learning models and their diagnostic value, 2022.
19. Jeremy C Wyatt and Douglas G Altman. Commentary: Prognostic models: clinically useful or quickly forgotten? *Bmj*, 311 (7019):1539–1541, 1995.
20. Mark Alan Fontana, Stephen Lyman, Gourab K Sarker, Douglas E Padgett, and Catherine H MacLean. Can machine learning algorithms predict which patients will achieve minimally clinically important differences from total joint arthroplasty? *Clinical orthopaedics and related research*, 477(6):1267, 2019.
21. Jonathan M. Snowden, Sherri Rose, and Kathleen M. Mortimer. Implementation of G-computation on a simulated data set: demonstration of a causal inference technique. *American Journal of Epidemiology*, 173(7):731–738, April 2011.
22. Matthew Sperrin, David Jenkins, Glen P Martin, and Niels Peek. Explicit causal reasoning is needed to prevent prognostic models being victims of their own success. *Journal of the American Medical Informatics Association*, 26(12):1675–1676, 2019.
23. Tony Blakely, John Lynch, Koen Simons, Rebecca Bentley, and Sherri Rose. Reflection on modern methods: when worlds collide–prediction, machine learning and causal inference. *International journal of epidemiology*, 49(6):2058–2064, 2020.
24. MA Hernán and JM Robins. *Causal Inference: What If.* CRC Boca Raton, FL.
25. Tyler J VanderWeele. Principles of confounder selection. *European journal of epidemiology*, 34:211–219, 2019.
26. James M. Robins and Sander Greenland. The role of model selection in causal inference from non experimental data. *American Journal of Epidemiology*, 123(3):392–402.
27. T. Wendling, K. Jung, A. Callahan, A. Schuler, N. H. Shah, and B. Gallego. Comparing methods for estimation of heterogeneous treatment effects using observational data from health care databases. *Statistics in Medicine*, (23):3309–3324.
28. Paul R Rosenbaum and Donald B Rubin. The central role of the propensity score in observational studies for causal effects. *Biometrika*, 70:41–55.
29. Peter C. Austin and Elizabeth A. Stuart. Moving towards best practice when using inverse probability of treatment weighting (IPTW) using the propensity score to estimate causal treatment effects in observational studies. *Statistics in Medicine*, 34(28): 3661–3679.
30. Sabrina Casucci, Li Lin, Sharon Hewner, and Alexander Nikolaev. Estimating the causal effects of chronic disease combinations on 30-day hospital readmissions based on observational medicaid data. *Journal of the American Medical Informatics Association*, 25(6):670–678, 2018.
31. Elysia Grose, Samuel Wilson, Jeffrey Barkun, Kimberly Bertens, Guillaume Martel, Fady Balaa, and Jad Abou Khalil. Use of propensity score methodology in contemporary high-impact surgical literature. *Journal of the American College of Surgeons*, 230(1):101–112.e2.
32. Xiaogang Su, Annette T Peña, Lei Liu, and Richard A Levine. Random forests of interaction trees for estimating individualized treatment effects in randomized trials. *Statistics in medicine*, 37(17):2547–2560, 2018.
33. Andrea Lamont, Michael D Lyons, Thomas Jaki, Elizabeth Stuart, Daniel J Feaster, Kukatharmini Tharmaratnam, Daniel Oberski, Hemant Ishwaran, Dawn K Wilson, and M Lee Van Horn. Identification of predicted individual treatment effects in randomized clinical trials. *Statistical methods in medical research*, 27(1):142–157, 2018.
34. Jeroen Hoogland, Joanna IntHout, Michail Belias, Maroeska M Rovers, Richard D Riley, Frank E. Harrell Jr, Karel GM Moons, Thomas PA Debray, and Johannes B Reitsma. A tutorial on individualized treatment effect prediction from randomized trials with a binary endpoint. *Statistics in medicine*, 40(26): 5961–5981, 2021.
35. Jennifer L. Hill. Bayesian nonparametric modeling for causal inference. *Journal of Computational and Graphical Statistics*, (1): 217–240.
36. Mark J. van der Laan and Sherri Rose. *Targeted Learning*. Springer Series in Statistics.
37. Megan S. Schuler and Sherri Rose. Targeted maximum likelihood estimation for causal inference in observational studies. *American Journal of Epidemiology*, 185(1):65–73.
38. Scott Powers, Junyang Qian, Kenneth Jung, Alejandro Schuler, Nigam H. Shah, Trevor Hastie, and Robert Tibshirani. Some methods for heterogeneous treatment effect estimation in high dimensions. *Statistics in Medicine*, 37(11):1767–1787.
39. Stefan Wager and Susan Athey. Estimation and inference of heterogeneous treatment effects using random forests. *Journal of the American Statistical Association*, 113(523):1228–1242.
40. Susan Athey, Julie Tibshirani, and Stefan Wager. Generalized random forests. *Annals of Statistics*, 47(2):1148–1178.
41. Sören R. Künzel, Jasjeet S. Sekhon, Peter J. Bickel, and Bin Yu. Metalearners for estimating heterogeneous treatment effects using machine learning. *Proceedings of the National Academy of Sciences*, 116(10):4156–4165.
42. Xinkun Nie and Stefan Wager. Quasi-oracle estimation of heterogeneous treatment effects. *Biometrika*, 108(2):299–319.
43. Victor Chernozhukov, Denis Chetverikov, Mert Demirer, Esther Duflo, Christian Hansen, Whitney Newey, and James Robins. Double/debiased machine learning for treatment and structural parameters. *The Econometrics Journal*, page 71.
44. Gang Fang, Izabela E Annis, Jennifer Elston-Lafata, and Samuel Cykert. Applying machine learning to predict real-world individual treatment effects: insights from a virtual patient cohort. *Journal of the American Medical Informatics Association*, 26(10):977–988, 2019.
45. Vincent Dorie, Jennifer Hill, Uri Shalit, Marc Scott, and Dan Cervone. Automated versus do-it-yourself methods for causal inference: Lessons learned from a data analysis competition. *Statistical Science*, 34(1):43–68, 2019.
46. Florent Le Borgne, Arthur Chatton, Maxime Léger, Rémi Lenain, and Yohann Foucher. G-computation and machine learning for estimating the causal effects of binary exposure statuses on binary outcomes. *Scientific reports*, 11(1):1435, 2021.
47. Jinma Ren, Paul Cislo, Joseph C Cappelleri, Patrick Hlavacek, and Marco DiBonaventura. Comparing g-computation, propensity score-based weighting, and targeted maximum likelihood estimation for analyzing externally controlled trials with both measured and unmeasured confounders: a simulation study. *BMC Medical Research Methodology*, 23(1):18, 2023.
48. Jean Kaddour, Aengus Lynch, Qi Liu, Matt J Kusner, and Ricardo Silva. Causal machine learning: A survey and open problems. arxiv 2022. *arXiv preprint arXiv:2206.15475*, 2022.
49. Victor Chernozhukov, Christian Hansen, Nathan Kallus, Martin Spindler, and Vasilis Syrgkanis. Applied causal inference powered by ml and ai. *arXiv preprint arXiv:2403.02467*, 2024.
50. Alejandro Schuler, Michael Baiocchi, Robert Tibshirani, and Nigam Shah. A comparison of methods for model selection when estimating individual treatment effects. *arXiv:1804.05146 [cs, stat]*.
51. Ahmed Alaa and Mihaela Van Der Schaar. Validating causal inference models via influence functions. *International Conference on Machine Learning*, pages 191–201.
52. Mary E. Charlson, Peter Pompei, Kathy L. Ales, and C.Ronald MacKenzie. A new method of classifying prognostic comorbidity in longitudinal studies: Development and validation. *Journal of Chronic Diseases*, 40(5):373–383.
53. James Robins. A new approach to causal inference in mortality studies with a sustained exposure period—application to control of the healthy worker survivor effect. *Mathematical*

Modelling, 7(9):1393–1512.
54. Ashley I Naimi and Brian W Whitcomb. Defining and identifying average treatment effects. *American Journal of Epidemiology*, 2023.
55. Guido W. Imbens and Donald B. Rubin. *Causal inference in statistics, social, and biomedical sciences*. Cambridge University Press.
56. Donald B Rubin. Causal inference using potential outcomes. *Journal of the American Statistical Association*, 100(469):322–331.
57. P. M. Robinson. Root-n-consistent semiparametric regression. *Econometrica*, (4):931–954.
58. Mark J van der Laan, MJ Laan, and JM Robins. *Unified methods for censored longitudinal data and causality*. Springer Science & Business Media.
59. Peter Schulam and Suchi Saria. Reliable decision support using counterfactual models. *Advances in neural information processing systems*, 30.
60. Uri Shalit, Fredrik D Johansson, and David Sontag. Estimating individual treatment effect: generalization bounds and algorithms. In *International Conference on Machine Learning*, pages 3076–3085. PMLR, 2017.
61. Matthieu Doutreligne and Gael Varoquaux. How to select predictive models for decision-making or causal inference? - caussim (version 1). [computer software]. https://archive.softwareheritage.org/swh:1:snp:d5621316ebbef4b5759ee54e4893a2954913134e;origin=https://github.com/soda-inria/caussim, 2025.
62. Matthieu Doutreligne and Gael Varoquaux. How to select predictive models for decision-making or causal inference? - causal model selection (version 1). [computer software]. https://archive.softwareheritage.org/swh:1:snp:36b289292192d9957b7b99f44654ab40fc49815d;origin=https://github.com/soda-inria/causal_model_selection, 2025.
63. Chanelle J. Howe, Stephen R. Cole, Daniel J. Westreich, Sander Greenland, Sonia Napravnik, and Joseph J. Eron. Splines for trend analysis and continuous confounder control. 22(6):874–875.
64. Aris Perperoglou, Willi Sauerbrei, Michal Abrahamowicz, and Matthias Schmid. A review of spline function procedures in r. 19(1):46.
65. Ali Rahimi and Benjamin Recht. Random features for large-scale kernel machines. In *Advances in Neural Information Processing Systems*, volume 20.
66. Lingjie Shen, Gijs Geleijnse, and Maurits Kaptein. Rctrep: An r package for the validation of estimates of average treatment effects. *Journal of Statistical Software*, 2023.
67. Kenneth R. Niswander and United States National Institute of Neurologiacal Diseases and Stroke. *The Women and Their Pregnancies: The Collaborative Perinatal Study of the National Institute of Neurological Diseases and Stroke*. National Institute of Health. Google-Books-ID: A0bdVhlhDQkC.
68. Yishai Shimoni, Chen Yanover, Ehud Karavani, and Yaara Goldschmnidt. Benchmarking framework for performance-evaluation of causal inference analysis. *arXiv:1802.05046 [cs, stat]*.
69. M. F. MacDorman and J. O. Atkinson. Infant mortality statistics from the linked birth/infant death data set–1995 period data. *Monthly Vital Statistics Report*, 46(6 Suppl 2):1–22, February 1998.
70. Christos Louizos, Uri Shalit, Joris Mooij, David Sontag, Richard Zemel, and Max Welling. Causal effect inference with deep latent-variable models. *Advances in neural information processing systems*.
71. Douglas Almond, Kenneth Y. Chay, and David S. Lee. The Costs of Low Birth Weight. *The Quarterly Journal of Economics*, 120(3), 2005.
72. Fabian Pedregosa, Gaël Varoquaux, Alexandre Gramfort, Vincent Michel, Bertrand Thirion, Olivier Grisel, Mathieu Blondel, Peter Prettenhofer, Ron Weiss, Vincent Dubourg, Jake Vanderplas, Alexandre Passos, David Cournapeau, Matthieu Brucher, Matthieu Perrot, and Édouard Duchesnay. Scikit-learn: Machine learning in python. *Journal of Machine Learning Research*, 12(85):2825–2830.
73. M. G. Kendall. A new measure of rank correlation. *Biometrika*, 30(1-2):81–93, June 1938.
74. Susan Athey and Guido Imbens. Recursive partitioning for heterogeneous causal effects. *Proceedings of the National Academy of Sciences*, 113(27):7353–7360, 2016.
75. Pierre Gutierrez and Jean-Yves Gerardy. Causal inference and uplift modeling a review of the literature. *Proceedings of The 3rd International Conference on Predictive Applications and APIs*, (67):14.
76. John C. Platt and John C. Platt. Probabilistic Outputs for Support Vector Machines and Comparisons to Regularized Likelihood Methods. *Advances in Large Margin Classifiers*, pages 61–74, 1999.
77. Bianca Zadrozny and Charles Elkan. Obtaining calibrated probability estimates from decision trees and naive Bayesian classifiers. page 8.
78. Alexandru Niculescu-Mizil and Rich Caruana. Predicting good probabilities with supervised learning. In *Proceedings of the 22nd international conference on Machine learning - ICML '05*, pages 625–632. ACM Press, 2005.
79. Matthias Minderer, Josip Djolonga, Rob Romijnders, Frances Hubis, Xiaohua Zhai, Neil Houlsby, Dustin Tran, and Mario Lucic. Revisiting the Calibration of Modern Neural Networks. *Advances in Neural Information Processing Systems*, 34:15682–15694, 2021.
80. Alexandre Perez-Lebel, Marine Le Morvan, and Gaël Varoquaux. Beyond calibration: estimating the grouping loss of modern neural networks. *arXiv preprint arXiv:2210.16315*, 2022.
81. Yishai Shimoni, Chen Yanover, Ehud Karavani, and Yaara Goldschmnidt. Ibm causal inference benchmarking data. https://www.synapse.org/Synapse:syn11738767, 2018.
82. Matthieu Doutreligne. Twins dataset used for experiment in the paper how to select predictive models for causal inference? [data set]. https://doi.org/10.5281/zenodo.14674618, 2025.
83. Matthieu Doutreligne. How to select predictive models for decision making or causal inference? experiments data [data set]. https://doi.org/10.5281/zenodo.13765465, 2024.

Declaration

Competing interests

No competing interest is declared.

Author contributions statement

M.D. conceived and conducted the experiments, M.D. and G.V. analyzed the results. M.D. and G.V. wrote and reviewed the manuscript.

Acknowledgments

We acknowledge fruitful discussions with Bénédicte Colnet.

A List of additional files

A.1 Variability of ATE estimation on ACIC 2016

A.2 Prior work : model selection for outcome modeling (g-computation)

A.3 Causal assumptions

A.4 Definitions of feasible risks

A.5 Proofs: Links between feasible and oracle risks

A.6 Measuring overlap

A.7 Experiments

A.8 Data split choices

A.9 Sensitivity of the results to different ratio of causal effect to baseline response

Manuscript
Click here to access/download;Manuscript;main_submission.pdf

Your PDF file "main_submission.pdf" cannot be opened and processed.
Please see the common list of problems, and suggested resolutions below.

Reason:

Other Common Problems When Creating a PDF from a PDF file

You will need to convert your PDF file to another format or fix the
current PDF file, then re-submit it.

Click here to access/download
Supplementary Material
additional_files_submission.pdf
