# Supplementary material for: How to select predictive models for decision-making or causal inference
Source: Gigascience. 2025 Mar 21;14:giaf016. doi: 10.1093/gigascience/giaf016 (PMC11927402; doi:10.1093/gigascience/giaf016)
Supplement: giaf016_Response_to_Reviewer_Comments_Original_Submission [file giaf016_response_to_reviewer_comments_original_submission.pdf]

Author's Response To Reviewer Comments

We thank the reviewers for their time, thoughts, and feedback on the manuscript. We have addresed the comments through additional work as well as clarifications in the revised manuscript.

To clarify the origin of the Robinson decomposition, as suggested by reviewer 1, we have added an equation. As a result the equation numbers in the revised manuscript are all shifted by one.

Reviewer 1

[...] The authors conceivably state that "The problem is that causal estimation requires controlling an error on both treated and non-treated outcome for the same individual.". Consequently, they report on estimator that attempt to consider this (in Section "Methods"). However, I cannot understand how this hint ("controlling error on both treated and non-treated sample (or patient?)") has been implemented by the procedure the authors describe in Equations (1), (2), and (3). If, I correctly understand", in Eq. (2) we see the g-computation estimators, a well-known concept introduced by [21] (and recently used also by https://www.nature.com/articles/s41598-020-65917-x), that splits the two configurations "treated" and "non-treated". [...] I understand that Eq. (2) and in Eq. (3) consider both treated and non-treated sample (or patient?), but those sums can be also equal to 0, even if their single terms are different from 0. Moreover, it is not clear if, and how Eq. (3) is related to Eq. (2): is it a variant? Is Eq. (3) derived from Eq. (2) under special assumptions?

> The reviewer is right that Eq. (2) is part of constructing the classic
> g-computation estimator, as well as Eq. (1) and Eq. (3). We added a
> sentence at the begining of the section to stress that it is a section
> summarizing a classic framework.

> Eq. (3) (now 4) is an expression of the estimator of the ATE based on a model of the outcome, sometimes called g-estimation. It is unbiased under consistency (Assumption. 3) and unconfoundedness (Assumption 1). To prove that, take the expectation of Eq. (3). Then apply Eq. (1) (now 2) which is true under consistency (Eq. 1 and Assumption 1) and unconfoundedness (Assumption 3). Then by the law of total expectancy, the conditionnality on X disappear and we recover Eq. (2) (now 3)

> G-estimation itself does not control error on both populations. The formulas in Eq. (1-3) (now 2-4) are all partly oracle formulas: they rely on conditional expectations but give no specific procedures on how to compute them. We added a clarification on this topic above Eq. (4) (now 5) and above Eq. (8) (now 9).

*Furthermore, the Robinson decomposition (R-decomposition) should be better discussed and its role in the context of causal inference should be better commented.
R-decomposition introduces two quantities, the conditional mean outcome and the probability to be treated (known as propensity score). What is the role of the propensity score? How is it calculated? Is it calculated simply by a frequentist approach (number of treated/total number of patients)?*

> G-computation relies on one choice of decomposition of the CATE
> estimation (Eq. (2)), which leads to into a sequence of regressions,
> where the only statistical estimate is the response function $\mu_a(x)$
> [Chernozhukov et al., 2024, chapter 15](https://causalml-book.org/). We
> have clarified that this decomposition is just a mathematical
> rewriting. The mean outcome from the treatment effect and the
> propensity score are two statistical quantity that enable this
> rewritting . Any statistical estimator can be chosen to model these
> quantities, in particular in the section were we introduce them, they
> are written in term of expectations, not detailing an estimation

> procedure, as this is discussed later. We have clarified this below
> formula 4 (formely 3) and in the begining of the section containing
> formula 9 (formely 8). A frequentist approach counting ratios of
> patients is one such estimator, but it will work only for a small
> number of discrete covariate. In practice, researchers often estimate
> the propensity score with a logistic regression, but other methods such
> as Boosted trees, Random Forests, or Neural Networks can be used. A
> good ps estimator should be well calibrated but this question is out of
> scope for this paper. In our discussion, we briefly mentioned this
> interesting research question on what properties should have a good
> estimator for the propensity score.

Finally, I note that the paper mentions inference methods specific to machine learning (random forest, etc.). In this regard, there is a great deal of study in the literature as to their actual usefulness in this field. I suggest the authors consider/review some of the following literature in the paper (those that the authors consider most appropriate to be included in their manuscript):

https://link.springer.com/book/10.1007/978-3-031-35051-1

https://www.causal-machine-learning.com/

https://www.frontiersin.org/journals/bioinformatics/articles/10.3389/fbinf.2021.746712/full

https://www.research-collection.ethz.ch/handle/20.500.11850/650703

https://aisel.aisnet.org/pacis2022/181/

or some other that can be easily found in literature.

> We thank the reviewer for the references. We have added several
> references for Causal ML into the manuscript, in particular [Kaddour et al.,
2022](https://arxiv.org/pdf/2206.15475), corresponding to
> https://www.causal-machine-learning.com/, and [Chernozhukov et al., 2024](https://causalml-book.org/) which both cover most of the
> subfields at the intersection of causal inference and machine learning.

Reviewer 2

Overall assessment:

*Let me begin with positive comments. I think the paper is very important because it describes a sophisticated methodology for experimental examination of causal models.
Despite some attempts this is still lacking in literature. I especially like the simulation scheme which uses different features for data generation and learning models. Moreover thorough experimental evaluations of causal models are not very frequent making the paper important. Code is available on Github which is a big plus.*

I am however not sure about the overall conclusions, which are that R-risk is always the best. Performance of risk functions may depend on several factors and the overall best performance might be a result of bias in available data. The only factor taken into account by the authors is overlap. However, only one type of lack of overlap is tested in experiments, based on shifted Gaussians. This will likely lead to violation of the positivity assumption. How about different scenarios, e.g.: perfect overlap but different treatment/control distributions? Confounding need not necessarily lead to lack of overlap. Another possible factor is the ratio of causal effect to background responses. It is known that it may affect relative performance of HTE estimators.

*Overall: I am no fully convinced that R-risk is always the best. The answer could be dependent on other

factors and I hope the authors would explore more scenarios.*

> We thank the reviewer for the thoughful comments and the call for
> nuance. Our first response is that our thoughts did not come out right
> and that it is not clear, indeed, that R-risk is always the best.
> Rather, that it is a good default solution. We have reformulate parts of
> the paper to convey this nuance better and added the nuance in the
> conclusion.

> In addition, the reviewer is correct that factors beyond overlap
> probably affect which risk is best. We have followed the suggestion to
> explore more diverse simulation scenarii, as detailing the sensitivity
> of our result on the R-risk makes a more nuanced and stronger analysis.
> Thus, we ran a dedicated experiment focusing on the ratio of causal
> effect to background response. We measure the ratio by the absolute
> mean difference between the causal effect and the baseline response,
> measured empirically on each dataset instance $\Delta_{\mu} =
> \frac{1}{N} \sum_{i=1}^N \big | \frac{\mu_{1}(x_i) -
> \mu_{0}(x_i)}{\mu_{0}(x_i) + \mu_{1}(x_i) - \frac{1}{N} \sum_{j=1}^N \mu_{0}(x_i) +
\mu_{1}(x_i)} \big|$. We vary a dedicated parameter
> (ω in Algorithm 2) in the simulation to force this ratio to
> cover a wide range of values. In 90 dataset instance, it ranges several
> orders of magnitude from 0.04 to 206. Figure 7 gives the main results
> of this experiment. We see that R-risk is still the best risk on
> average, but when the effect ratio is high (tertile between 9 and
> 9675), the reweighted mu-risk and the tau-risk_IPW are better than
> the R-risk. However, for low value of the ratio, the R-risk largely
> outperforms all other metrics. We added these new results to the main
> paper. We also studied another characteristic of the simulation: the variance of the causal effect
measured as the empirical version of $\frac{var(\mu_1 - \mu_0)}{var(\mu_1+\mu_0)}$. For this last
experiment, we found similar results to the causal effect experiment. We include it in the supp mat for
completeness about our reflexion.

> We did not explore more sophisticated treatment/control distributions for our simulation. Building these
new data generation processes would imply a big modification to our setup. We rely on the easy inversion
of the distribution functions of the gaussians (p(X|A=a)) to analytically compute the true propensity score.
In addition, we think that the semi-simulated datasets already provide some interesting argument in favor
of r-risk with more more diverse treatment/control covariate distributions.

*Another question: do the differences between risks in experiments follow known theoretical results? For
example, it is well known (e.g.
https://www.semanticscholar.org/reader/112aba9c62f98b9684693f4c20c30274bc9eb1dc or [Rudas ans
jaroszewisz, 2017](https://link.springer.com/content/pdf/10.1007/s10618-018-0576-8.pdf)) that the tau
risk performs poorly.*

> The high variability of the U-learner has already been noted by [Nie and Wager,
2021](https://arxiv.org/pdf/1712.04912). This advantage of the R-loss that does not have a propensity
score to the denominator is also discussed in [Chernozhukov et al. 2024, chapter 15.](https://causalml-
book.org/assets/chapters/CausalML_chap_15.pdf). The convergence rate of the R-loss has been proven in
[Foster and Syrgkanis, 2023](https://arxiv.org/pdf/1901.09036). We are not aware of theoretical results
comparing simple robust losses (such as mu-risk or mu-risk IPW) to orthogonal losses such as R-loss, U-
loss or DR-Loss.
> Empirically, the differences between risks have been highlighted in other papers such as [Schuler, et al.,
2018](https://arxiv.org/pdf/1804.05146) for quadratic and stepwise data generation processes or [Curth
et Van der Schaar, 2023](https://proceedings.mlr.press/v202/curth23b/curth23b.pdf) exploring more
diverse outcome generation processes focusing on CATE sparsity (as a proxy of its complexity) based on a
semi-simulated dataset for the covariate (ACIC2016). They both underline good performances for the R-

risk (see Table.1 for Schuler and Fig. 3 for Curth). A remark that we made in our manuscript is that the results of Schuler are convincing for randomization setups but not for observational (non-randomized) setups. Curth's results are more convincing for observational setups but did not explore the overlap assumption.

> The second paper is from the uplift regression literature. It focus on full randomization problems and a linear specification with gaussian predictors. It shows theoretically that a linear T-learner (called double model approach in their approach) has smaller asymptotical variance than a treatment ratio reweighted outcome S learner. However, they note that in non normal cases the T-learner can be ill-defined or with high variance. They propose a corrected reweighted S-learner approach to reduce this variance. They show empirically that it behaves better than the non corrected version on a simulation and two real world problems.
> I am not sure to see the link with the tau-risk. The paper does not compare risks but corrected or uncorrected vs T-learners. In addition, they only consider full randomization and linear problems. In our case, we consider a more general setting with a variety of data generation procedures and we focus on non-randomized situations where the distribution of the treatment is conditioned by the predictors.

Another problem is that the paper is hard to follow. The authors rapidly switch between topics: risk, selection, nuisance parameter estimation so the reader quickly gets lost. The sections in supplementary materials contain important information but not all are referenced in main text. The paper gives an impression of several experiments put together in some order without guiding the reader through the maze. I think organization should be improved before publication.

> We tried to better guide the reader through the method section and to clarify the motivation behind the different experiments.
> We checked the supplementary materials but we did not find any appendix that was not referenced at least once in the main text (several are referred in figure captions). Did you have a specific appendix in mind that would require a reference in the main text?

Other comments:

In the Methods section g-computation is described. It seems indeed the first name under which the technique appeared in literature, but other names, most importantly S-learner are being used frequently nowadays. Interestingly those names are mentioned in Empirical study on page 4, however, the close relationship (equivalence?) between S-learner and g-computation is not mentioned.

> G-computation is larger than S-learner. The first mention of S-learner that we are aware of is [Künzel, 2019](https://arxiv.org/pdf/1706.03461). In this paper, they distinguish T-learners from S-learners (or more elaborated ones). T-learners model $\mu(0)$ and $\mu(1)$ separately and train separately on each population. On the contrary S learner use only one model for both population and add the treatment as a covariate. A good example to understand the difference is to note that a linear T-learner is the same thing as a linear S-learner where we add an interaction term between the treatment and the covariates. Ultimately, both methods are G-computation in the sense that they rely on the identification of eq. (1) making the link between potential outcomes and observed quantities. To improve the manuscript, we added just after the eq. (3) that one can choose to model the response function with either a T-learner or a S-learner.

Equation 2: should the expectation be over X not Y?

> Thanks for the remark. We corrected a typo in the equation. There should not have any mention of X in the conditional expectation.

page 17: proposition number is missing

> Thanks for this remark. We fixed it.

A.7: the data generation procedure needs better explanations. Why not call (7) P(d|A) since that's how its used later? On page 17 you say '[b1,bD]~P(x)' and later 'x~P(x)' which causes confusion. Also, how are beta vectors generated?

> We assumed that you talked about the equation 17 rather than 7. Could you point where we use the P(d|A) notation? We did not find it in the manuscript.
> The notation b1, ..bD ~P(x) was meant to be understood as the beta vectors are drawn from a distribution P(x). The later mention of 'x~P(x)' was meant to be understood as the definition if the RBF function. We removed the second mention which causes confusion.
> The beta vectors are drawn from two normal distributions with mean 0 and unit variances. We added this information in the manuscript.
> We added the Algorithm 2, hopping that it would help the reader to understand exactly how we generate the data for our simulation.

page 4: TLearner uses different feature representation for each treatment arm. Does this really happen in practice? Usually all subgroups are described with the same set of features.

> It is indeed true that in usual practice all subgroups are described with the same set of features. However, as soon as we are using a machine learning (necessary to model CATE) such as a random forest or boosted trees, the inner representations of the features inside the model is different for both population. This idea has been exploited to build representation aware neural net models for cate estimation by [Johansson et al., 2022](https://www.jmlr.org/papers/volume23/19-511/19-511.pdf). It is also the case for simpler models such as a linear model with treatment interaction and lasso regularization.

page 4/5: Family of candidate estimators: why are only linear models used in some dataset and forests for other? The leaf sizes of forests are chosen from a rather small range, why not use some percentage of the size of the training set?

> For the simulations we chose estimators that are out of the functional class of the true data generation process (a candidate estimator cannot model exactly the data generation process) but that are quite similar to it. They are out of the functional class because the representers are different from the one of the true data (they are two randomly sampled data points). They are close because they have the same functional form : a linear model on top of two data representers. We used the simulations to explore the wider range of data generation processes, focusing on a gradient of overlap. Because of the sheer number of generated dataset, we did not include computationally costly boosted trees in the candidate family.

> The choice of the candidate family of estimators was guided by the idea to compare estimators that are not too different from each other, making the model selection task more challenging. The important point is that we are not focusing on the best possible estimator but on the best possible model selection procedure. To identify the optimal procedure, it is more convincing to compare close candidate estimators than a wide variety of candidate estimators. In the later case, one estimator could dominate all other estimators for every selection procedure. This explains why we are choosing a narrow range of leaf sizes for the forests.

Language comments:
----

*It seems that English might need improvement in some cases, for
example:*

Abstract: "We study such a variety of model selection procedures in practical settings": a "large variety" seems more appropriate.

On page 1, the sentence "Predictive modeling bridges to causal inference theory under the name of outcome models ..." contains "bridges under the name" which also sounds strange.

On page 2: In the sentence "Then we anchor causal model selection in the potential outcome framework and details the causal risks and model-selection procedure." I believe 'details' should be 'detail'.

Algorithm 1: 'a candidate estimators f' should be 'a candidate estimator f'

throughout: "an RBF expansion" is more commonly used than "a RBF expansion"

page 4: 'for both population' should be 'for both populations'

> Thanks for the remarks. We corrected the typos and improved the language as suggested.
