# Supplementary material for: How to select predictive models for decision-making or causal inference
Source: Gigascience. 2025 Mar 21;14:giaf016. doi: 10.1093/gigascience/giaf016 (PMC11927402; doi:10.1093/gigascience/giaf016)
Supplement: giaf016_Response_to_Reviewer_Comments_Revision_1 [file giaf016_response_to_reviewer_comments_revision_1.pdf]

Author's Response To Reviewer Comments

1) Data Availability section added with references to items in the bibliography for the code and datasets

2) Removal of the footnotes and replacement with citation or in-text when approriate

3) ORCIDs added in the title page (without proper formatting)

4) additional file added as a df

5) FIxed typo

6) LaTex source files added
