# Supplementary material for: How to select predictive models for decision-making or causal inference
Source: Gigascience. 2025 Mar 21;14:giaf016. doi: 10.1093/gigascience/giaf016 (PMC11927402; doi:10.1093/gigascience/giaf016)
Supplement: giaf016_Reviewer_1_Report_Original_Submission — Paola Lecca -- 8/12/2024 [file giaf016_reviewer_1_report_original_submission.pdf]

Reviewer Report

Title: How to select predictive models for decision-making or causal inference?

Version: Original Submission **Date:** 8/12/2024

Reviewer name: Paola Lecca

Reviewer Comments to Author:

I appreciate the approch and the level of detail that this paper offers on an issue that is, to say the least, a hot topic in bioinformatics and data science in general: the evaluation of the best 'error' model when choosing a causal model for predictive purposes. Indeed, there is much confusion on the subject and very often error models are applied without any real awareness of their potential and limitations.
That is why I think the paper can be of great educational value as well as research.
Of particular interest is the statement "the best predictor may not estimate best causal effects".
Central to the understanding of the paper is Figure 1 and the concept it seeks to explain. The figure clearly shows that if we rely on R^2, a predictor is preferred which on average is efficient but not on estimating causal quantities. The authors conceivably state that "The problem is that causal estimation requires controlling an error on both treated and non-treated outcome for the same individual.".
Consequently, they report on estimator that attempt to consider this (in Section "Methods"). However, I cannot understand how this hint ("controlling error on both treated and non-treated sample (or patient?)") has been implemented by the procedure the authors describe in Equations (1), (2), and (3).
If, I correctly understand", in Eq. (2) we see the g-computation estimators, a well-known concept introduced by [21] (and recently used also by https://www.nature.com/articles/s41598-020-65917-x), that splits the two configurations "treated" and "non-treated".
Other reference on g-computation to consider in the study can be for example:
- https://www.nature.com/articles/s41598-021-81110-0
- https://www.sciencedirect.com/science/article/pii/S0167947306002076
- https://bmcmedresmethodol.biomedcentral.com/articles/10.1186/s12874-023-01835-6
- https://pubmed.ncbi.nlm.nih.gov/34861799/

I understand that Eq. (2) and in Eq. (3) consider both treated and non-treated sample (or patient?), but those sums can be also equal to 0, even if their single terms are different from 0. Moreover, it is not clear if, and how Eq. (3) is related to Eq. (2): is it a variant? Is Eq. (3) derived from Eq. (2) under special assumptions?
Furthermore, the Robinson decomposition (R-decomposition) should be better discussed and its role in the context of causal inference should be better commented.
R-decomposition introduces two quantities, the conditional mean outcome and the probability to be treated (known as propensity score). What is the role of the propensity score? How is it calculated? Is it calculated simply by a frequentist approach (number fo treated/total number of patients?
Finally, I note that the paper mentions inference methods specific to machine learning (random forest, etc.). In this regard, there is a great deal of study in the literature as to their actual usefulness in this field. I suggest the authors consider/review some of the following literature in the paper (those that the

authors consider most appropriate to be included in their manuscript):
https://link.springer.com/book/10.1007/978-3-031-35051-1
https://www.causal-machine-learning.com/
https://www.frontiersin.org/journals/bioinformatics/articles/10.3389/fbinf.2021.746712/full
https://www.research-collection.ethz.ch/handle/20.500.11850/650703
https://aisel.aisnet.org/pacis2022/181/
or some other that can be easily found in literature.

Methods

Are the methods appropriate to the aims of the study, are they well described, and are necessary controls included? Choose an item.

Conclusions

Are the conclusions adequately supported by the data shown? Choose an item.

Reporting Standards

Does the manuscript adhere to the journal's guidelines on minimum standards of reporting? Choose an item.

Choose an item.

Statistics

Are you able to assess all statistics in the manuscript, including the appropriateness of statistical tests used? Choose an item.

Quality of Written English

Please indicate the quality of language in the manuscript: Choose an item.

Declaration of Competing Interests

Please complete a declaration of competing interests, considering the following questions:

- Have you in the past five years received reimbursements, fees, funding, or salary from an organisation that may in any way gain or lose financially from the publication of this manuscript, either now or in the future?
- Do you hold any stocks or shares in an organisation that may in any way gain or lose financially from the publication of this manuscript, either now or in the future?
- Do you hold or are you currently applying for any patents relating to the content of the manuscript?

- Have you received reimbursements, fees, funding, or salary from an organization that holds or has applied for patents relating to the content of the manuscript?
- Do you have any other financial competing interests?
- Do you have any non-financial competing interests in relation to this paper?

If you can answer no to all of the above, write 'I declare that I have no competing interests' below. If your reply is yes to any, please give details below.

I declare that I have no competing interests.

I agree to the open peer review policy of the journal. I understand that my name will be included on my report to the authors and, if the manuscript is accepted for publication, my named report including any attachments I upload will be posted on the website along with the authors' responses. I agree for my report to be made available under an Open Access Creative Commons CC-BY license (http://creativecommons.org/licenses/by/4.0/). I understand that any comments which I do not wish to be included in my named report can be included as confidential comments to the editors, which will not be published.

Choose an item.

To further support our reviewers, we have joined with Publons, where you can gain additional credit to further highlight your hard work (see: https://publons.com/journal/530/gigascience). On publication of this paper, your review will be automatically added to Publons, you can then choose whether or not to claim your Publons credit. I understand this statement.

Yes Choose an item.
