# Supplementary material for: How to select predictive models for decision-making or causal inference
Source: Gigascience. 2025 Mar 21;14:giaf016. doi: 10.1093/gigascience/giaf016 (PMC11927402; doi:10.1093/gigascience/giaf016)
Supplement: giaf016_Reviewer_2_Report_Original_Submission — Szymon Jaroszewicz -- 8/16/2024 [file giaf016_reviewer_2_report_original_submission.pdf]

Reviewer Report

Title: How to select predictive models for decision-making or causal inference?

Version: Original Submission **Date:** 8/16/2024

Reviewer name: Szymon Jaroszewicz

Reviewer Comments to Author:

Overall assessment:

Let me begin with positive comments. I think the paper is very important because it describes a sophisticated methodology for experimental examination of causal models.
Despite some attempts this is still lacking in literature. I especially like the simulation scheme which uses different features for data generation and learning models. Moreover thorough experimental evaluations of causal models are not very frequent making the paper important. Code is available on Github which is a big plus.
I am however not sure about the overall conclusions, which are that R-risk is always the best. Performance of risk functions may depend on several factors and the overall best performance might be a result of bias in available data. The only factor taken into account by the authors is overlap. However, only one type of lack of overlap is tested in experiments, based on shifted Gaussians. This will likely lead to violation of the positivity assumption. How about different scenarios, e.g.: perfect overlap but different treatment/control distributions? Confounding need not necessarily lead to lack of overlap.
Another possible factor is the ratio of causal effect to background responses. It is known that it may affect relative performance of HTE estimators.
Overall: I am no fully convinced that R-risk is always the best. The answer could be dependent on other factors and I hope the authors would explore more scenarios.
Another question: do the differences between risks in experiments follow known theoretical results? For example, it is well known (e.g.
https://www.semanticscholar.org/reader/112aba9c62f98b9684693f4c20c30274bc9eb1dc or
https://link.springer.com/content/pdf/10.1007/s10618-018-0576-8.pdf) that the tau risk performs poorly.
Another problem is that the paper is hard to follow. The authors rapidly switch between topics: risk, selection, nuisance parameter estimation so the reader quickly gets lost. The sections in supplementary materials contain important information but not all are referenced in main text. The paper gives an impression of several experiments put together in some order without guiding the reader through the maze. I think organization should be improved before publication.
Other comments:

In the Methods section g-computation is described. It seems indeed the first name under which the technique appeared in literature, but other names, most importantly S-learner are being used frequently nowadays. Interestingly those names are mentioned in Empirical study on page 4, however,

the close relationship (equivalence?) between S-learner and g-computation is not mentioned.
Equation 2: should the expectation be over X not Y?
page 17: proposition number is missing
A.7: the data generation procedure needs better explanations. Why not call (7) P(d|A) since that's how its used later? On page 17 you say '[b1,bD]~P(x)' and later 'x~P(x)' which causes confusion. Also, how are beta vectors generated?
page 4: TLearner uses different feature representation for each treatment arm. Does this really happen in practice? Usually all subgroups are described with the same set of features.
page 4/5: Family of candidate estimators: why are only linear models used in some dataset and forests for other? The leaf sizes of forests are chosen from a rather small range, why not use some percentage of the size of the training set?
Language comments:

It seems that English might need improvement in some cases, for example:
Abstract: "We study such a variety of model selection procedures in practical settings": a "large variety" seems more appropriate.
On page 1, the sentence "Predictive modeling bridges to causal inference theory under the name of outcome models ..." contains "bridges under the name" which also sounds strange.
On page 2: In the sentence "Then we anchor causal model selection in the potential outcome framework and details the causal risks and model-selection procedure." I believe 'details' should be 'detail'.
Algorithm 1: 'a candidate estimators f' should be 'a candidate estimator f'
throughout: "an RBF expansion" is more commonly used than "a RBF expansion"
page 4: 'for both population' should be 'for both populations'
etc.

Methods

Are the methods appropriate to the aims of the study, are they well described, and are necessary controls included? Choose an item.

Conclusions

Are the conclusions adequately supported by the data shown? Choose an item.

Reporting Standards

Does the manuscript adhere to the journal's guidelines on [minimum standards of reporting?](#) Choose an item.

Choose an item.

Statistics

Are you able to assess all statistics in the manuscript, including the appropriateness of statistical tests used? Choose an item.

Quality of Written English

Please indicate the quality of language in the manuscript: Choose an item.

Declaration of Competing Interests

Please complete a declaration of competing interests, considering the following questions:

- Have you in the past five years received reimbursements, fees, funding, or salary from an organisation that may in any way gain or lose financially from the publication of this manuscript, either now or in the future?
- Do you hold any stocks or shares in an organisation that may in any way gain or lose financially from the publication of this manuscript, either now or in the future?
- Do you hold or are you currently applying for any patents relating to the content of the manuscript?
- Have you received reimbursements, fees, funding, or salary from an organization that holds or has applied for patents relating to the content of the manuscript?
- Do you have any other financial competing interests?
- Do you have any non-financial competing interests in relation to this paper?

If you can answer no to all of the above, write 'I declare that I have no competing interests' below. If your reply is yes to any, please give details below.

'I declare that I have no competing interests'

I agree to the open peer review policy of the journal. I understand that my name will be included on my report to the authors and, if the manuscript is accepted for publication, my named report including any attachments I upload will be posted on the website along with the authors' responses. I agree for my report to be made available under an Open Access Creative Commons CC-BY license (http://creativecommons.org/licenses/by/4.0/). I understand that any comments which I do not wish to be included in my named report can be included as confidential comments to the editors, which will not be published.

Choose an item.

To further support our reviewers, we have joined with Publons, where you can gain additional credit to further highlight your hard work (see: https://publons.com/journal/530/gigascience). On publication of this paper, your review will be automatically added to Publons, you can then choose whether or not to claim your Publons credit. I understand this statement.

Yes Choose an item.
